# Design and Synthesis of New Quinazolin-4-one Derivatives with Negative mGlu_7_ Receptor Modulation Activity and Antipsychotic-Like Properties

**DOI:** 10.3390/ijms24031981

**Published:** 2023-01-19

**Authors:** Katarzyna Kaczorowska, Anna Stankiewicz, Ryszard Bugno, Maria H. Paluchowska, Grzegorz Burnat, Piotr Brański, Paulina Cieślik, Joanna M. Wierońska, Mariusz Milik, Mateusz Nowak, Agnieszka Przybyłowicz, Aneta Kozioł, Agata Hogendorf, Adam S. Hogendorf, Justyna Kalinowska-Tłuścik, Beata Duszyńska, Andrzej Pilc, Andrzej J. Bojarski

**Affiliations:** 1 Department of Medicinal Chemistry, Maj Institute of Pharmacology, Polish Academy of Sciences, 12 Smętna Street, 30-343 Kraków, Poland; 2 Department of Neurobiology, Maj Institute of Pharmacology, Polish Academy of Sciences, 12 Smętna Street, 30-343 Kraków, Poland; 3 SELVITA S.A., Bobrzyńskiego 14, 30-348 Kraków, Poland; 4 Department of Crystal Chemistry and Crystal Physic, Faculty of Chemistry, Jagiellonian University, Gronostajowa 2, 30-387 Kraków, Poland

**Keywords:** mGlu_7_ metabotropic glutamate receptor 7, negative allosteric modulators (NAMs), antipsychotic

## Abstract

Following the glutamatergic theory of schizophrenia and based on our previous study regarding the antipsychotic-like activity of mGlu_7_ NAMs, we synthesized a new compound library containing 103 members, which were examined for NAM mGlu_7_ activity in the T-REx 293 cell line expressing a recombinant human mGlu_7_ receptor. Out of the twenty-two scaffolds examined, active compounds were found only within the quinazolinone chemotype. 2-(2-Chlorophenyl)-6-(2,3-dimethoxyphenyl)-3-methylquinazolin-4(3*H*)-one (**A9-7**, **ALX-171**, mGlu_7_ IC_50_ = 6.14 µM) was selective over other group III mGlu receptors (mGlu_4_ and mGlu_8_), exhibited satisfactory drug-like properties in preliminary DMPK profiling, and was further tested in animal models of antipsychotic-like activity, assessing the positive, negative, and cognitive symptoms. **ALX-171** reversed DOI-induced head twitches and MK-801-induced disruptions of social interactions or cognition in the novel object recognition test and spatial delayed alternation test. On the other hand, the efficacy of the compound was not observed in the MK-801-induced hyperactivity test or prepulse inhibition. In summary, the observed antipsychotic activity profile of **ALX-171** justifies the further development of the group of quinazolin-4-one derivatives in the search for a new drug candidate for schizophrenia treatment.

## 1. Introduction

Metabotropic glutamate (mGlu) receptors are a family of class C G protein-coupled receptors (GPCRs) comprising eight subtypes classified into three groups (group I, mGlu_1,5_; group II, mGlu_2,3_; group III, mGlu_4,6,7,8_) [1]. Among them, the mGlu_7_ subtype is widely expressed in the brain regions associated with reward, cognition, and emotion, i.e., the cerebral cortex [2,3,4], hippocampus [2,3,5], amygdala [2,3], and basal ganglia [2,3,6]. In line with this location, as well as based on pharmacological studies, the mGlu_7_ receptor is postulated to be an attractive therapeutic target for numerous neurological and psychiatric disorders, including schizophrenia [7], depression [8,9], anxiety [10,11,12,13,14,15], post-traumatic stress disorders [14,16], Rett syndrome [17,18,19], epilepsy [20], neurodevelopmental disorders [21], autism [22], ADHD [23], and addiction [24,25,26].

Out of the group III mGlu receptors, the mGlu_7_ receptor has a low affinity for endogenous agonists [27,28], which is several orders of magnitude lower than that for mGlu_4_ and mGlu_8_ receptors, requiring a high glutamate concentration (almost 10 mM) for a full receptor activation.

**Figure 1:**
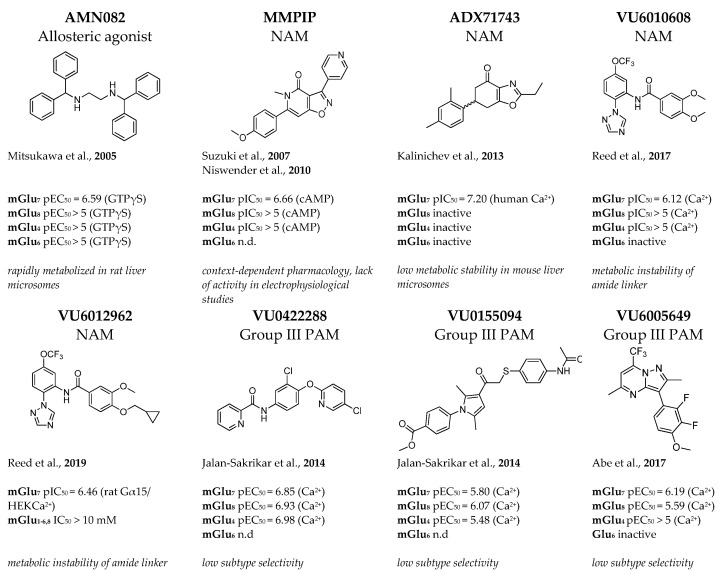
Summary of the first chemical structures of reported mGlu_7_ receptor tool compounds (NAM—negative allosteric modulator; PAM—positive allosteric modulator) [13,17,18,20,21,29,36,38,39]. The activity is shown as the pEC_50_/pIC_50_ value; assay type is indicated in parentheses: cAMP, cAMP accumulation; Ca^2+^, calcium mobilization; GTPγS, GTPγS binding; n.d.—no data.

Due to there only being a small number of selective ligands with good bioavailability, the mGlu_7_ receptor is one of the least-studied members of the family. Most of the described compounds (AMN082 [26,29,30,31], MMPIP [32,33,34], ADX71743 [6,7,33,35], VU6010608 [36] VU6012962 [37], VU0155094 [38], VU0422288 [38], and VU6005649 [17]) have not demonstrated either a high selectivity or desired pharmacokinetic properties, and/or high potency, as shown in Figure 1. All of these compounds regulate the activity of the mGlu_7_ receptor through allosteric modulation by enhancing (positive allosteric modulator—PAM) or weakening (negative allosteric modulator—NAM) its function.

**Figure 2:**
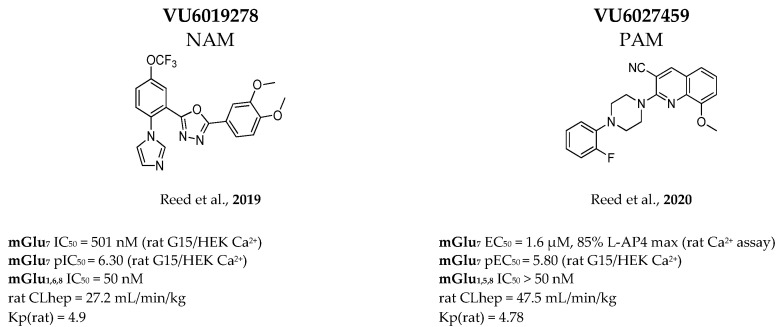
Chemical structures of the last reported mGlu_7_ receptor tool compounds [37,40] (NAM—negative allosteric modulator; PAM—positive allosteric modulator). EC_50_—effective concentration 50; IC_50_—inhibitory concentration 50; assay type indicated in parenthesis; rat CL hep—predicted hepatic clearance in rat; Kp(rat)—rat brain/plasma ratio.

Recent discoveries of potent, highly selective, orally bioavailable, CNS penetrant, and metabolically stable NAM and PAM mGlu_7_ modulators, VU6019278 [37] and VU6027459 [40], respectively, as shown in Figure 2, have provided new opportunities for essential research on mGlu_7_ receptor pharmacology.

Regarding the importance of mGlu_7_ receptor modulation for potential therapeutic applications, our previous study explored the role of negative mGlu_7_ receptor modulation in animal models of schizophrenia [7]. The promising results of this study showed that the new mGlu_7_ ligands could accelerate the development of a unique drug candidate in this class of molecular targets.

With this in mind, we now focus on designing and discovering new structural scaffolds and potential mGlu_7_ NAMs, and assessing their therapeutic potential based on behavioral tests commonly used in antipsychotic drug development.

## 2. Results

### 2.1. Scaffold Design

**Figure 3:**
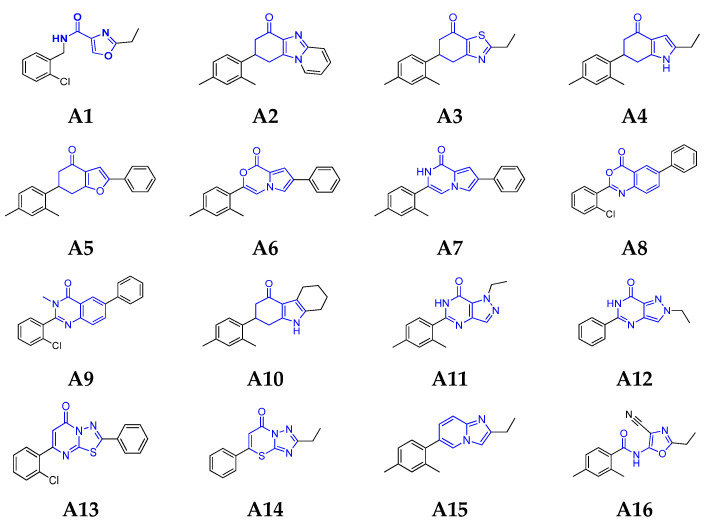

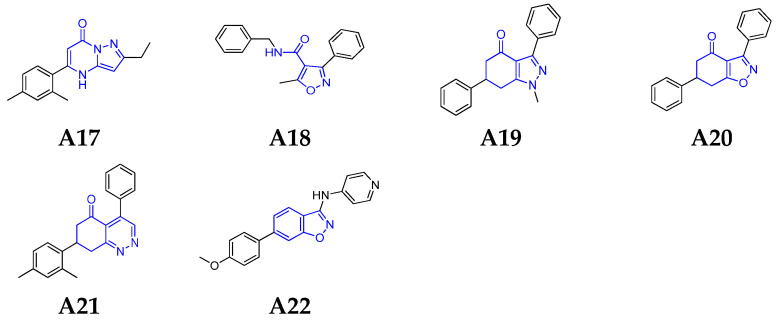
Examples from the designed compound library (all compounds are presented in the Supplementary Materials) consisting of 22 chemotypes derived from ADX71743 or MMPIP tool compounds (cores outlined in blue) (**A1**–**A22**). Structures **A14**–**A17** generated via Cresset field methodology.

Starting the search for new scaffolds, the structures of the primary tool compounds available at that time (MMPIP and ADX71743) were applied. In addition to a bioisosteric replacement, which was the primary strategy to design the **A1**–**A13** and **A18**–**A22** chemotypes, the Cresset field methodology was used to develop the **A14**–**A17** scaffolds, as shown in Figure 3.

**Figure 4:**
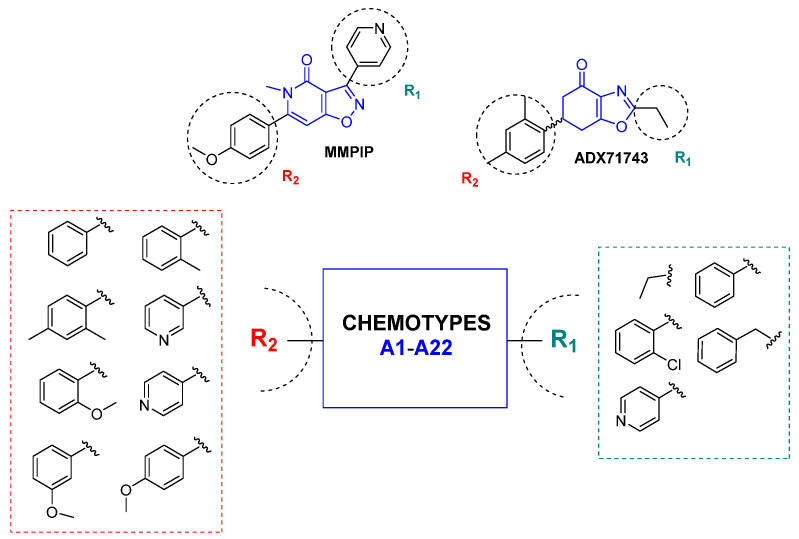
Terminal moieties (R_1_ and R_2_) introduced to designed chemotypes **A1**–**A22** (shown in the Supplementary Materials, summarized in Tables S1 and S2) via analogy to reference tool compounds MMPIP and ADX71743.

Initially, the terminal groups present in the reference structures were used to substitute the designed cores (Figure 3), i.e., 4-methoxyphenyl and 4-pyridyl in MMPIP and 2,4-dimethylphenyl and ethyl in ADX71743. Additionally, selected scaffolds were decorated with other R_1_ and R_2_ substituents (Figure 4), which were previously used in SAR studies of ADX compounds [35].

**Figure 5:**
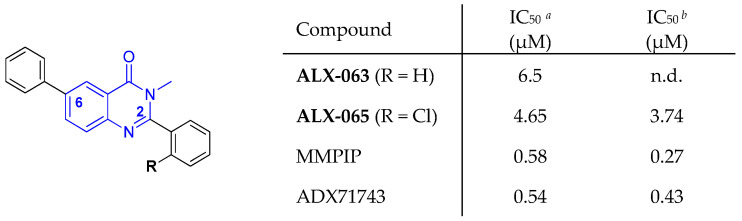
Structures of hit and tool compounds and mGlu_7_ NAM activities determined with *^a^* T-REx 293 cell line [41] and *^b^* CHO-K1 cell line methods reported in the Supplementary Materials—Sections 2.1 and 2.2; Figures S2—S4; n.d.—not determined.

The compound’s activity was examined on recombinant-human-mGlu_7_-receptor-expressing cells by detecting the level of cyclic AMP in the presence of forskolin, an adenylyl cyclase activator. Among all the derivatives (Tables S1 and S2 in the Supplementary Materials), only two that belonged to chemotype **A9** exhibited negative allosteric modulation activity toward the mGlu_7_ receptor, i.e., 3-methyl-2,6-diphenylquinazolin-4(*3H*)-one (**A9-1**, **ALX-063**, IC_50_ = 6.5 µM) and 2-(2-chlorophenyl)-3-methyl-6-phenylquinazolin-4(*3H*)-one (**A9-3**, **ALX-065**, IC_50_ = 4.65 µM) (Figure 5). This new 4(*3H*)-quinazolinone scaffold, structurally different from the previously known mGlu_7_ NAM chemotypes, was subjected to further optimization.

### 2.2. Chemistry of 2,6-Disubstituted (3H)-quinazolin-4-ones

**Scheme 1:**
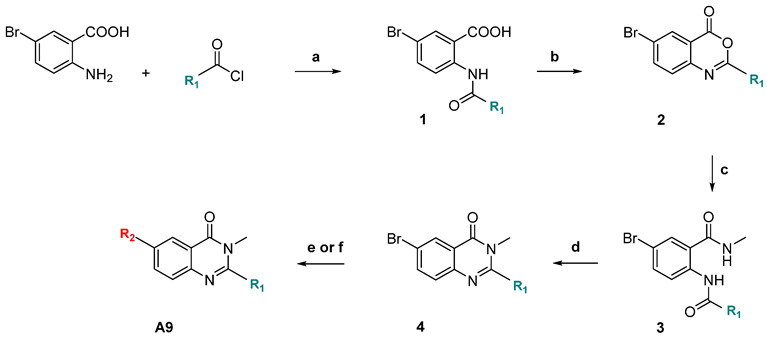
Synthetic route to 2,6-disubstituted(3H)-quinazolin-4-ones (chemotype **A9**); R_1_ and R_2_ substituents are listed in Figure 6. Reagents and conditions: (**a**) TEA, THF (or DCM), RT, overnight; (**b**) acetic anhydride, DCM (or neat), reflux, 3–20 h; (**c**) MeNH_2_ in THF, DME (or EtOAc), RT, 1–16 h; (**d**) KOH (or NaOH), EtOH, reflux, 1 h–5 days; (**e**) boronic acid/ester, Pd(dppf)Cl_2_ · DCM, K_2_CO_3_, 2N Na_2_CO_3_, 80 °C, 1,4-dioxane, toluene; or boronic acid/ester, Pd(dppf)Cl_2_ · DCM, K_3_PO_4_, DMF, water, 80 °C; (**f**) Pd_2_(dba)_3_, DavePhos, 1,4-dioxane, toluene, K_3_PO_4_ or Cs_2_CO_3_, 80 °C.

2,6-disubstituted(*3H*)-quinazolin-4-ones (**A9**) were synthesized according to the synthetic path outlined in Scheme 1. The synthetic method involved the acylation of commercially available 6-bromoanthranilic acid with corresponding acid chlorides to afford amide **1,** followed by dehydration under heating with acetic anhydride to benzoxazinone **2**. The subsequent condensation of **2** with an appropriate amine and intramolecular dehydrative cyclization of diamide derivative **3** yielded key 2-substituted 6-bromo(*3H*)-quinazolin-4-ones (**4**). The final 6-susbstituted-quinazolin-4(*3H*)-ones (**A9**) were synthesized using C–C coupling methods, such as Pd-catalyzed Suzuki–Miyaura cross-coupling reactions, or applying the Buchwald coupling procedure (Scheme 1, steps e and f, respectively).

### 2.3. Structural Modification of Hit Compounds

**Figure 6:**
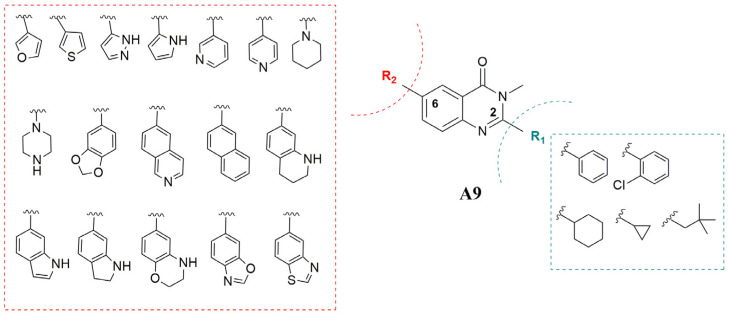
Structural modifications explored for the **A9** chemotype at the C-2 and C-6 positions. All analogs (depicted in Table S1, Supplementary Materials) were inactive as mGlu_7_ NAMs (IC_50s_ > 10 µM).

The synthesis of derivatives of the hit compounds **ALX-063** and **ALX-065** started from the tolerability exploration of various substituents at the C-2 (R_1_) and C-6 (R_2_) positions (Figure 6). The incorporation of both aliphatic and aromatic substituents, i.e., cyclopropyl, neopentyl, cyclohexyl, and 2-pyridyl, at the C-2 position, when the phenyl moiety at the C-6 position was unchanged, resulted in inactive analogs (mGlu_7_ IC_50_ > 10 μM, e.g., Table S2, Supplementary Materials). Similarly, analogs of **ALX-065** with diverse heterocyclic moieties at the C-6 position (furan, thiophene, pyrazole, pyrrole, oxazole, or bulkier fused systems, such as naphthalene, benzothiazole, isoquinoline, 1*H*-indole, 2,3-dihydro-1*H*-indole, and 1,2,3,4-tetrahydroquinoline) were found to be inactive (mGlu_7_ IC_50_ values >10 µM, Table S2, Supplementary Materials, entries **A9-13**–**A9-32**).

Among all of the tested substituents at the C-2 and C-6 positions, only the terminal groups applied in the structures of the hit compounds influenced the mGlu_7_ NAM activity of the quinazoline-4(3*H*)-one derivatives. Thus, in the next step of structural modifications, due to the slightly better activity of **ALX-065** vs. **ALX-063**, the 2-chlorophenyl moiety at the C-2 position was used, whereas the second terminal phenyl, by analogy to MMPIP, was replaced with a 4-methoxyl group (**A9-6**, Table 1). Although the desired activity was not observed, a series of **A9-6** derivatives with methoxyl group/s in different positions of the phenyl moiety was synthesized. Generally, except for 2-(2-chlorophenyl)-6-(2,3-dimethoxyphenyl)-3-methylquinazolin-4(3*H*)-one (**A9-7**, **ALX-171)**, containing the 2,3-dimethoxyphenyl moiety (Table 1), the other mono/dimethoxyphenyl analogs (**A9-8**–**A9-12**) displayed a complete lack of potency.

In the next step, selectivity tests of both **ALX-065** and **ALX-171** at other group III mGlu receptors (mGlu_4_ and mGlu_8_) were conducted, revealing only mGlu_4_ NAM activity of **ALX-065** (19.6% at 10 µM) (Supplementary Materials, Section 2.3, Figures S5 and S6). Finally, the crystal structures were determined for these two modulators, and initial DMPK profiling and in vivo behavioral tests were performed.

### 2.4. Crystal Structure Analysis

The molecular structures of both most active compounds, **ALX-065** and **ALX-171,** were confirmed with the use of a single-crystal X-ray diffraction experiment. The asymmetric unit of the crystal **ALX-065** consisted of two independent molecules, which represented two alternative conformations. This solid-state observation indicated the significant rotational freedom of the benzene substituent in the solution. This was not observed for the structure of **ALX-171**, where, for both aromatic fragments at C-2 and C-6, there were spatial substituents in the *ortho* positions, leading to a stabilization and reduction in rotation due to steric hindrance.

**Figure 7:**
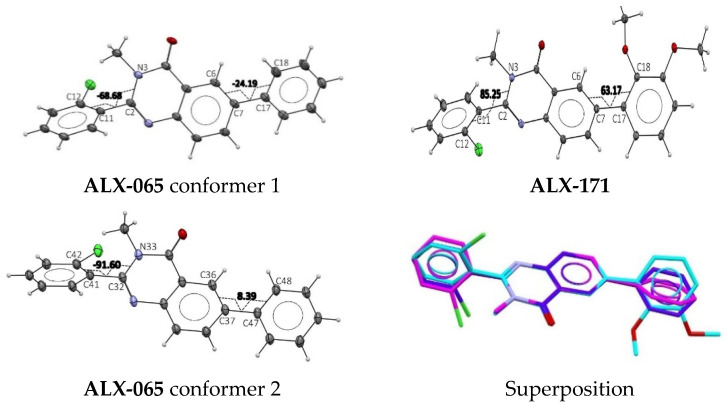
The molecular geometries observed in the investigated crystal structures of **ALX-065** and **ALX-171** with superposition of all presented conformers (the RMSD for atoms of the 3-methyl-quinazolin-4-one moiety was 0.03; **ALX-065** conformer 1, conformer 2, and **ALX-171** are shown with C-atoms in magenta, violet, and light blue, respectively). Additional information is depicted in Section 1.3, Figure S1, Table S3, Supplementary Materials.

The orientation of both aromatic fragments at C-2 and C-6 to the 3-methyl-quinazolin-4-one moiety was a result of two effects. First, it was strictly dependent on the *ortho* substituent. Second, both mentioned rings influenced their mutual arrangement, which was observed in structure **ALX-065**. The angular position of *o*-Cl-benzene with respect to the 3-methyl-quinazolin-4-one ring system (approx. 65°) favored the angular orientation of benzene at C-6 (approx. 25°), whereas the perpendicular orientation of *o*-Cl-benzene observed for conformer two led to a nearly flat arrangement of the remaining molecular fragment (*see* Figure 7; conformers one and two of **ALX-065**). Despite *o*-Cl-benzene being in proximity to the perpendicular orientation (approx. 85°) in structure **ALX-171,** the angular orientation of the *o*,*m*-dimethoxy benzene was preferential.

### 2.5. Preliminary DMPK Profile

#### 2.5.1. Pharmacokinetic

The pharmacokinetic properties and brain uptake of the new 2,6-disubstituted (3*H*)-quinazolin-4-one derivatives (**ALX-065** and **ALX-171**) and tool compounds (ADX71743 and MMPIP) were determined in mice after an *i.p*. administration of a dose of 10 mg/kg in 3% DMSO + 20% Captisol in water. The results shown in Table 2 confirmed that all analyzed compounds entered the circulation system and reached the brain quickly (T_max_ (plasma and brain): 15 min for ADX71743, **ALX-065,** and **ALX-171**, and 30 min for MMPIP). Of these compounds, **ALX-171** exhibited the longest half-life (T_1/2_ = 5.56 h) and the highest brain exposure (AUC_0-t_ = 13.85 μM × h/L).

As shown in Table 3, **ALX-171** also exhibited a good oral bioavailability (F = 58%) after the administration of a 5 mg/kg dose.

#### 2.5.2. In Vitro Metabolic Stability and CYP450 Inhibition

The data presented in Table 4 show that **ALX-171** exhibited a significantly higher microsomal stability with the lowest intrinsic clearance compared to the other compounds tested. Moreover, this compound was also characterized by relatively good kinetic solubility. Regarding the cytochrome P450 inhibition profile, all compounds weakly inhibited (IC_50_ > 10 μM) isoforms 2B6 and 2D6, while strong inhibition (IC_50_ < 1.1) was observed only for MMPIP for isoforms 3A4 and 2C19.

### 2.6. In Vivo Behavioral Tests

Based on our previous study regarding the putative antipsychotic-like activity of mGlu_7_ NAMs [7], we aimed to assess the efficacy of newly synthesized compounds to reverse schizophrenia-like symptoms in several behavioral tests. Since **ALX-171** had better drug-like properties (kinetic solubility and metabolic stability) than **ALX-065,** and relatively good bioavailability, this compound was selected for the in vivo profiling. The antipsychotic-like activity was evaluated, assessing each group of symptoms (positive, negative, and cognitive) that may manifest in schizophrenic patients.

Positive symptoms include hallucinations, delusions and disorganized behavior, which, in animals, are modeled using tests such as the DOI (2,5-dimethoxy-4-iodoamphetamine)-induced head twitch response (HTR) and MK-801 (dizocilpine)-induced hyperactivity. Negative symptoms are associated with anhedonia, a blunted affect, or social withdrawal. In animals, social disturbances can be measured with social interaction tests. Cognitive symptoms (memory and attention deficits) can be assessed with the novel object recognition test, spatial delayed alternation test, and prepulse inhibition test.

#### 2.6.1. Tests Predictive of Positive Symptoms

##### DOI-Induced HTR

**Figure 8:**
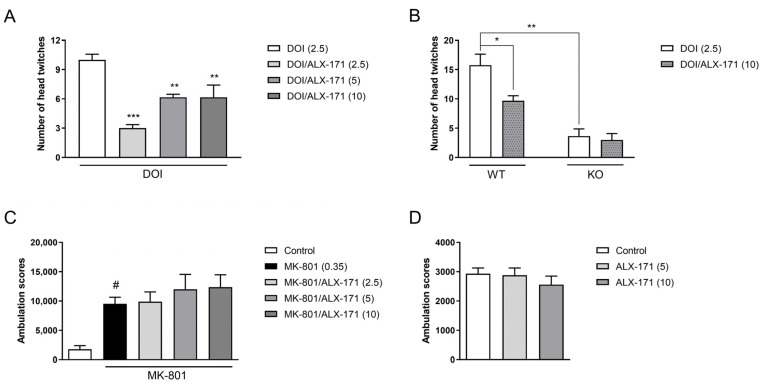
The effect of **ALX-171** on the DOI-induced HTR test (**A**,**B**) and MK-801-induced hyperactivity (**C**). The effect of **ALX-171** on the locomotor activity of naïve mice (**D**). Doses are indicated in parentheses as mg/kg. The data are presented as means ± SEM. (**A**) One-way ANOVA: F_(3.20)_ = 15.46, *p* < 0.0001; ** *p* < 0.01; *** *p* < 0.001 vs. DOI-treated group. (**B**) Two-way ANOVA: KO (F_(1.11)_ = 51.33, *p* < 0.0001); ALX-171 administration (F_(1.11)_ = 6.432, *p* < 0.03); * *p* < 0.05, ** *p* < 0.01 vs. DOI-treated WT mice. (**C**) # *p* < 0.0001 vs. control group.

The administration of DOI induced characteristic head shakes in mice. **ALX-171** significantly attenuated the number of head shakes at all tested doses (2.5, 5, and 10 mg/kg), compared with the control group (Figure 8A). Additionally, mGlu_7_ knockout (KO) animals were used to confirm that the observed effects are mGlu_7_-receptor-dependent. The deletion of the mGlu_7_ receptor resulted in a marked reduction in DOI-induced head twitches, and **ALX-171** had no further effect on head twitches in KO mice, which suggested that the effect of **ALX-171** was mGlu_7_ receptor-specific (Figure 8B).

##### MK-801-Induced Hyperactivity

The administration of MK-801 resulted in an increase in locomotor activity in mice. **ALX-171** was not able to reverse the MK-801-induced increase in locomotor activity at any of the administered doses (2.5, 5, or 10 mg/kg) (Figure 8C). The administration of **ALX-171** had no effect on spontaneous locomotor activity in naïve mice (Figure 8D).

#### 2.6.2. Test Predictive of Negative Symptoms

##### Social Interaction Test

**Figure 9:**
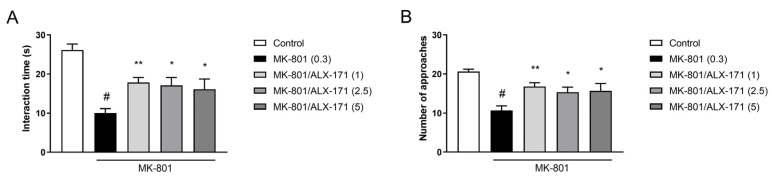
The effect of **ALX-171** on MK-801-induced deficits in the social interaction test. Doses are indicated in parentheses as mg/kg. The data are presented as means ± SEM. One-way ANOVA: (**A**) interaction time F_(3.38)_ = 5.346, *p* < 0.02; (**B**) number of approaches F_(3.38)_ = 4.871, *p* < 0.006; * *p* < 0.05; ** *p* < 0.01 vs. MK-801-treated group; (**A**) # *p* < 0.01, (**B**) # *p* < 0.0001 vs. control group.

The administration of MK-801 resulted in significant disruptions in both the number of social episodes and the total time of interactions between mice. **ALX-171** reversed the MK-801-induced disruptions in the interaction time and number of episodes at all tested doses (Figure 9A,B).

#### 2.6.3. Tests Predictive of Cognitive Symptoms

##### Novel Object Recognition Test

**Figure 10:**
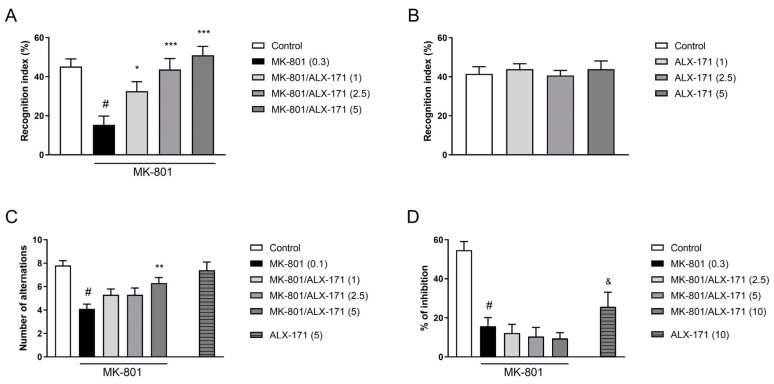
The effect of **ALX-171** on MK-801-induced deficits (**A**) or in naïve mice (**B**) in the NOR test. The effect of **ALX-171** on MK-801-induced deficits in the spatial delayed alternation test (**C**) and prepulse inhibition (**D**). Doses are indicated in parentheses as mg/kg. The data are presented as means ± SEM. (**A**) One-way ANOVA: F_(3.36)_ = 10.08, *p* < 0.0001; * *p* < 0.05; *** *p* < 0.001 vs. MK-801-treated group; # *p* < 0.01 vs. control group. (**C**) One-way ANOVA: F(3.36) = 3.344, *p* < 0.05; ** *p* < 0.01 vs. MK-801-treated group; # *p* < 0.0001 vs. control group. (**D**) Student’s *t*-test: *t* = 3.459, df = 26, & *p* < 0.01 vs. control group; *t* = 6.272, df =40, # *p* < 0.0001 vs. control group.

The administration of MK-801 induced a profound decrease in the recognition index in the NOR test in mice. **ALX-171** ameliorated the effects of the MK-801 administration at all tested doses (1, 2.5, and 5 mg/kg) (Figure 10A). It was observed that the compound had no significant impact on the recognition index after the administration of the compound alone (Figure 10B).

##### Spatial Delayed Alternation Test

The administration of MK-801 decreased the choice accuracy in the spatial delayed alternation test in rats. **AXL-171** at the highest dose tested (5 mg/kg) reversed the MK-801-induced disruptions in the number of alternations (Figure 8C). The compound, when administered alone, had no negative effect on choice accuracy in this test (Figure 8C).

##### Prepulse Inhibition Test

The administration of MK-801 decreased the prepulse inhibition in rats. **ALX-171** had no effect on the MK-801-induced effects at any of the investigated doses (Figure 8D). The compound disturbed the PPI response (~50%) when given alone at the highest investigated dose of 10 mg/kg.

## 3. Discussion

Although the efficacy of currently available antipsychotics toward positive symptoms of schizophrenia is satisfactory, the reversal and treatment of negative and cognitive symptoms are debatable. The latter two groups of symptoms persist chronically, have the most significant negative impact on daily functioning, and remain treatment-resistant [42,43]. Therefore, the search for new antipsychotic drugs focuses on providing relief to these groups of symptoms, and the determination to find new targets for the drug is well-founded. Metabotropic glutamate receptors have been widely studied in this field [42,44], and activators of mGlu_2_ or mGlu_4_ receptors have been indicated as promising targets. The mGlu_7_ subtype has been the least investigated thus far. According to the glutamatergic theory of schizophrenia, an increased glutamate efflux is responsible for schizophrenia arousal [42,45,46,47]. Therefore, the inhibition of this increased activity of glutamatergic neurons presumably constitutes the most appropriate way to reverse schizophrenia symptoms. The first choice is to use activators of receptors mGlu_2_ or mGlu_4_, which serve as autoreceptors.

In contrast to receptors mGlu_2_ and mGlu_4_, which inhibit the glutamate release when activated, the mGlu_7_ receptor acts rather as a heteroreceptor expressed on GABAergic terminals that regulate the GABA release. NAMs of mGlu_7_, including **ALX-171**, inhibit receptors expressed on the terminals of GABAergic neurons, thus, facilitating the GABA release. This neurotransmitter is a principal inhibitory amino acid in the brain that controls and inhibits excitation, including the glutamatergic network. Consequently, increasing its activity with mGlu_7_ inhibition may counteract an abnormal glutamatergic excitation.

Although new subtype-selective negative allosteric mGlu_7_ receptor modulators have recently been identified, only a few have been shown to have acceptable DMPK profiles allowing for a robust validation of their therapeutic potential. Here, we reported on an early-lead selection in the search for new mGlu_7_ NAMs and their preliminary in vitro and in vivo preclinical characterization. The efforts to identify **ALX-171** were not straightforward, and confirmed complexities in designing allosteric modulators of mGlu receptors. Indeed, steep and flat SAR and subtle structural changes that affect CNS penetration and DMPK properties are frequently reported problems in numerous studies devoted to developing ligands with an allosteric mechanism of action [36,46,48,49].

The synthesized compound library, containing 103 chemicals representing 22 chemotypes (**A1-A17** and **M1-M5),** included only three compounds (quinazoline analogs, chemotype **A9**) that exhibited NAM mGlu_7_ activity in the T-REx 293 cell line. Furthermore, the structural modification around the active 4(*3H*)-quinazolinone basic skeleton at the C-2 and C-6 positions showed that the incorporation of a wide range of substituents was not tolerated; the phenyl moieties were found to be important for interactions with the mGlu_7_ receptor on both sides of the basic nucleus. The incorporation of a 2,3-dimethoxyphenyl substituent at the C-6 position of the basic molecule did not significantly change the NAM activity of the mGlu_7_ receptor; however, **ALX-171** showed an improved selectivity toward the mGlu_4_-receptor-enhanced stability and increased solubility, compared to the unsubstituted derivative **ALX-065**. Interestingly, despite the comparable activity and slight differences in chemical structure between **ALX-065** and **ALX-171**, the crystal structure analysis showed some diversity in the examined crystals, e.g., the presence of two conformers in **ALX-065** crystals, and various angular orientations at the C6 position for both molecules. The differences observed in the analyzed crystals suggested that the mutual orientation of terminal aromatic substituents may be important for interactions inside the allosteric binding site. This hypothesis is currently under investigation.

An in vivo pharmacokinetic characterization of **ALX-171** revealed that this compound possessed good exposure in the brain and oral bioavailability. As reported in our previous studies, a positive allosteric modulator of the mGlu_7_ receptor, AMN082, exerted propsychotic activity in animal models [50], while the modulation of the mGlu_7_ receptor through NAMs, e.g., ADX71743 or MMPIP, exerted antipsychotic activity in animal models of schizophrenia [7]. ADX71743 was active in all the tests used, including the DOI-induced head twitches, MK-801-induced disruptions in social interaction, cognitive impairment in novel object recognition, and prepulse inhibition, while the activity of MMPIP was not observed in MK-801-induced disruptions of social interaction or in PPI.

In the present study, **ALX-171** was screened for its potential antipsychotic-like activity. The compound reversed DOI-induced head twitches and MK-801-induced disruptions of social interactions or cognition in the novel object recognition test and spatial delayed alternation test. The efficacy of the compound was not observed in the MK-801-induced hyperactivity test or prepulse inhibition at any dose tested.

Despite some differences observed in vivo between **ALX-171** and the reference compounds ADX71743 and MMPIP, the antipsychotic activity profile of **ALX-171** justified the further development of the group of quinazolin-4-one derivatives in the search for a new drug candidate. At the same time, our results confirmed, once again, the involvement of the inhibition of the mGlu_7_ receptor in the therapeutic effects on schizophrenia.

## 4. Materials and Methods

### 4.1. Chemistry

#### 4.1.1. Materials

All the chemicals employed in the syntheses were purchased from commercial vendors such as Merck (Darmstadt, Germany), Fluorochem (Hadfield, UK), Apollo (Bredbury, UK), and Combi-Blocks (San Diego, USA) and were used without purification. Solvents and inorganic reagents were acquired from Chempur (Piekary Śląskie, Poland). Reaction progress was monitored with TLC on Merck Silica Gel 60 F 254 on aluminum plates. Column chromatography was performed on Merck Silica Gel 60 (0.063–0.200 mm; 70–230 mesh ASTM).

#### 4.1.2. Analytical Methods

A UPLC/MS analysis was performed on a Waters TQD spectrometer combined with a UPLC Acquity H-Class with PDA eLambda detector. A Waters Acquity UPLC BEH C18 1.7 μm 2.1 × 50 mm chromatographic column was used at 40 °C, a 0.3 mL/min flow rate, and 1.0 μL injection volume (the samples were dissolved in LC–MS-grade acetonitrile, typically used at a concentration of 0.1–1 mg/mL prior to injection). All mass spectra were recorded under electrospray ionization in the positive mode (ESI+) and chromatograms were recorded with UV detection in the range of 190–300 nm. The gradient conditions used were: 80% phase A (water + 0.1% formic acid) and 20% phase B (acetonitrile + 0.1% formic acid) to 100% phase B (acetonitrile + 0.1% formic acid) at 3.0 min, kept for 3.5 min, and then to initial conditions for 4.0 min and kept for an additional 2.0 min. Total time of analysis—6.0 min.

#### 4.1.3. Purity Analysis

The ^1^H NMR spectra were measured at 300 MHz or 500 MHz, and the ^13^C NMR spectra at 75 MHz or 126 MHz on a Varian Mercury-VX (300 MHz/500 MHz) spectrometer in CDCl_3_ or d_6_-DMSO solutions with TMS as an internal standard. The spectral data of new compounds refer to their free bases. Chemical shifts are expressed in (ppm). Splitting patterns describe apparent multiplicities and were designated as s (singlet), d (doublet), t (triplet), q (quartet), m (multiplet), and br s (broad singlet). Coupling constants are given in units of hertz (Hz). In cases where it was possible, multiplets in ^13^C NMR were identified and denoted in the experimental part. Unless otherwise noted, presented compounds were of at least 95% purity as determined with LC–MS. Syntheses and characterization details for intermediate products and final compounds, as well as the spectral data for all compounds, are included in the Supplementary Materials.

#### 4.1.4. Software

ChemDraw Prime (v. 20.0.0.41, PerkinElmer, Waltham, USA) was used for drawing chemical structures, substructures and reactions; Marvin (v19.8.0, Chemaxon, Budapest, Hungary) and Instant JChem (v20.20.0, Chemaxon, Budapest, Hungary) were used for displaying structures, calculating physical–chemical properties and chemical data management. MestReNova (v11.0.1-17801, Mestrelab Research S.L., Santiago de Compostela, Spain) was used to visualize, process, analyze, and report on ^1^H and ^13^C NMR spectra.

#### 4.1.5. Synthesis

Syntheses and characterization for intermediate products and final compounds, as well as the spectral data for all compounds, are included in the Supplementary Materials. The final step of the procedure for preparing compounds of the A9 chemotype (**ALX-171** analogs) was presented below.

##### General Procedure for the Synthesis of 2,6-Disubstituted(3H)-quinazolin-4-ones (**A9-1**–**A9-36**)

Method A. A mixture of 6-bromo(3*H*)-quinazolin-4-ones (**4**, Scheme 1) (0.48 mmol), appropriate phenylboronic acid (0.72 mmol, 1.5 eq), potassium carbonate (1.44 mmol, 3 eq), and 1 mL of 2N Na_2_CO_3_ aq solution in 4 mL of toluene and 1 mL of 1,4-dioxane was degassed with argon, and a Pd(dppf)Cl_2_ complex in DCM (0.024 mmol, 0.05 eq) was added. The reaction was run under microwave irradiation at 120 °C for 20 min, cooled to room temperature (RT), and diluted with chloroform (50 mL) and water (30 mL). The layers were separated and the aqueous layer was extracted with chloroform (3×). Combined organic layers were washed with brine, dried over MgSO_4_, and evaporated to give a dark oily product. The crude product was purified with silica gel column chromatography followed by slurring with 2-PrOH/hexane (1:3) to yield the desired products.

Method B. A mixture of 6-bromo(3*H*)-quinazolin-4-ones (**4**, Scheme 1) (2.0 mmol), appropriate phenylboronic acid (2.58 mmol, 1.3 eq), and K_2_CO_3_ (6 mmol, 3 eq) was suspended in 1,4-dioxane (14 mL) and toluene (7 mL) and degassed with argon. Then, the Pd(dppf)Cl_2_ complex in DCM (0.2 mmol) was added. The reaction was run in a sealed tube at 80 °C for 1 h, cooled to RT, and diluted with chloroform (50 mL) and water (30 mL). The layers were separated and the aqueous layer was extracted with chloroform (3 × 20 mL). The combined organic layers were washed with brine, dried over MgSO_4_, and evaporated. The product was purified with silica gel column chromatography using hexane/EtOAc as an eluent, followed by triturating with 2-PrOH/hexane (1:3) at RT to yield the desired compounds.

Method C. We started with 6-bromo(3*H*)-quinazolin-4-ones (**4**, Scheme 1) (0.54 mmol), corresponding phenylboronic acid (0.70 mmol, 1.3 eq), potassium phosphate tribasic (1.36 mmol, 2.5 eq), a Pd(dppf)Cl_2_ complex in DCM (0.05 mmol) in 4 mL of DMF, and 3 mL of water. The reaction was run for 1 h in a sealed tube at 80 °C, cooled to RT, and diluted with ethyl acetate (60 mL) and water (20 mL). The layers were separated and the aqueous layer was extracted with ethyl acetate (3×). The combined organic layers were washed with brine, dried over MgSO_4_, and evaporated, and the crude product was purified with silica gel column chromatography using hexane/EtOAc, followed by slurring with 2-PrOH/hexane (1:3) to yield **A-9** derivatives.

#### 4.1.6. Crystal Structure Determination

Compounds **ALX-065** and **ALX-171** were crystallized using the slow evaporation method from solution under ambient conditions. For the crystallization process, a mixture of solvents was applied—water:2-propanol:acetone—in a ratio of 45%:45%:10%. X-ray diffraction data for selected monocrystals were collected using an XtalLAB Synergy-S four circle diffractometer with a mirror monochromator and a microfocus CuKα radiation source (λ = 1.5418 Å). Additionally, the diffractometer was equipped with the CryoStream cryostat system, allowing for low-temperature experiments performed at 100(2) K. The obtained datasets were processed with CrysAlisPro software [51]. The phase problem was solved with direct methods using SIR2014 [43]. Parameters of the obtained models were refined with full-matrix least-squares on F^2^ using SHELXL-2014/6 [52]. Calculations were performed using the WinGX integrated system (v2014.1) [53]. Figures were prepared with Mercury 4.0 software [54]. All nonhydrogen atoms were refined anisotropically. All hydrogen atoms attached were positioned with the idealized geometry and refined using the riding model with the isotropic displacement parameter U_iso_[H] = 1.2 U_eq_[C] for all but the methyl groups, for which U_iso_[H] = 1.5 U_eq_[C] was applied. Crystal data and refinement results are shown in Table S6. Asymmetric units, presenting an atom numbering scheme, are shown in Figure S1. The crystallographic data were deposited with the Cambridge Crystallographic Data Centre as supplementary publication nos.: CCDC2130726(**ALX-065**), CCDC2130727(**ALX-171**). Copies of the data can be obtained, free of charge, on application to CCDC, 12 Union Road, Cambridge, CB2 1EZ, UK (e-mail: deposit@ccdc.cam.ac.uk).

### 4.2. In Vitro

#### 4.2.1. cDNA Constructs and Cell Lines

The cell lines with the tetracycline-inducible expression of human metabotropic receptors 4, 7, and 8 were described in detail by Chruścicka et al. [41] (mGlu_4_ receptor NM_000841, mGlu_7_ receptor NM_000844.2, and mGlu_8_ receptor NM_000845). Cells were grown under standard cell culture conditions (37 °C, 5% CO_2_) in DMEM supplemented with 10% tetracycline-free FBS. The expression was induced through the addition of tetracycline to the culture medium at 0.75 μg/mL.

#### 4.2.2. Forskolin-Induced cAMP Production Assay

The determination of the intracellular cAMP through homogeneous time-resolved fluorescence (HTRF) was described in detail previously [41]. Briefly, 48 h before the experiments, the expression of the receptor was induced through the addition of tetracycline to the culture medium; the next day, FBS and L-Glu were removed from the medium. Just before the cAMP measurement, the cells were detached and centrifuged. Then, the cell suspension in Hanks-HEPES (130 mM NaCl, 5.4 mM KCl, 1.8 mM CaCl_2_, 0.8 mM MgSO_4_, 0.9 mM NaH_2_PO_4_, 20 mM HEPES, and 3.25 mM glucose; pH 7.4) was incubated in the presence of 10 µM forskolin (mGlu_7_ receptor, 3 µM forskolin), the agonist LSP4-2022 or L-Glu (for mGlu_4_ and mGlu_8_), and a compound for 5 min. The intracellular concentration of cyclic AMP was measured by means of a cAMP Gi HTRF kit from Cisbio, according to the manufacturer’s instructions. After 1 h of incubation at room temperature, the fluorescence at 620 nm and 665 nm was read (Tecan Infinite M1000). The results were calculated as the 665 nm/620 nm ratio multiplied by 10^4^. Each sample was prepared in triplicate. Data were analyzed using GraphPad Prism version 5.04 for Windows (GraphPad Software, San Diego, USA).

#### 4.2.3. In Vitro Activity

The activity of the compounds was examined in the T-REx 293-cell-line-expressing recombinant human mGlu_7_ receptor by detecting the level of cyclic AMP in the presence of 5 µM forskolin [7,41]. The cell line was verified using the reference negative allosteric modulators ADX71743 and MMPIP. The substances were incubated with 5 µM LSP4-2022 (EC_80_). Both ADX71743 and MMPIP dose-dependently antagonized LSP4-2022 in the presence of forskolin, with IC_50_ values of 0.58 µM (±0.27, n = 5) and 0.54 µM (±0.470, n = 3), respectively (Figure S2, Supplementary Materials).

Firstly, all compounds were screened at 10 μM using three cell lines expressing the mGlu_4/7/8_ receptor to evaluate selectivity. For the mGlu_4_ and mGlu_8_ receptors, glutamic acid was used as an agonist and had good potency. For the mGlu_7_ receptor, we used LSP4-2022 as an agonist due to its homogeneity and better potency, compared to L-Glu (~1 mM). VU0155041 and AZ12216052 were used as reference compounds for the mGlu_4_ and mGlu_8_ receptors, respectively. The parameter described as “% of inhibition” was introduced to compare the bioactivity of the new compounds to the reference NAM at a 10 μM concentration in the presence of 5 μM LSP4-2022 (0%) and 3 μM forskolin (100%). For ADX71743, the percent inhibition was 42.61% (±7.56; n = 10). For compounds that passed the screening procedure, the dose–response curve in the presence of LSP4-2022 in 5 μM was evaluated compared to reference NAM ADX71743. Only compounds **ALX-063**, **ALX-065,** and **ALX-171** (Figure S3, Supplementary Materials) met our conditions regarding bioactivity. All of them were further investigated to determine the EC_50_ and receptor selectivity.

### 4.3. Pharmacokinetic Studies

The method described below was successfully applied to a pharmacokinetic study of all tested compounds (**ALX-065**, **ALX-171**, ADX71743, and MMPIP) in mice (Albino Swiss) after an *i.p.* injection. Compounds were administered to mice at a dose of 10 mg/kg *i.p.* Plasma and tissue samples were collected at 0.25, 0.50, 1.0, 2.0, 4.0, 6.0, and 24 h. Plasma and tissue samples from all drug-treated animals were thawed at room temperature prior to use. The standard protocol for sample preparation was used: 200 μL of acetonitrile was added to the Eppendorf tubes with 50 μL of studied plasma samples or tissue homogenate. Samples were mixed for 5 min on a mixer at 25 °C and 1400 rpm. The tubes were then centrifuged at 2000× *g* for 15 min at 4 °C. A total of 180 μL of each supernatant was transferred to a plate well. Finally, each sample was injected into the LC–MS column. Calibration curve serial dilution method: Plasma was spiked with a standard at different concentrations. Acetonitrile was added. After mixing and centrifugation, the supernatant was collected.

#### LC–MS Analysis

Chromatographic conditions. Plasma and tissue samples from all drug-treated animals at selected time points were analyzed using a previously developed nonvalidated LC–MS/MS method. A sensitive and highly selective liquid chromatography–tandem mass spectrometry (LC–MS) method was used to determine the drug concentration in mouse plasma samples or tissue homogenates. The LC–MS analysis was carried out on a Bruker amaZon SL mass spectrometer using the positive/negative ion ESI mode. Chromatographic separation was achieved on an Ascentis Express C18 column (5 cm × 2.1 mm, 2.7 μM, Supelco Technologies) at room temperature with a thermostatted column oven. A gradient elution of eluents A (acetonitrile (LiChrosolv, Reag. Ph Eur) + 0.1% formic acid (Sigma Aldrich, 98–100%)) and B (water + 0.1% formic acid) were used for separation. The flow rate was set at 1 mL/min. The injection volume was 20 μL, and the time of injection was 4 min.

Mass spectrometric conditions. An ion trap mass spectrometer (Bruker amaZon SL, Bruker, Bremen, Germany) was equipped with an electrospray source operating in the positive/negative ion mode. Data were collected and processed using Bruker Quant Analysis (Bruker, Bremen, Germany) software. A quantification of the analytes was performed in the SIM mode.

### 4.4. CYP Inhibition Assay

CYP inhibition was performed with the recombinant enzymes CYP1A2, CYP2B6, CYP2C9, CYP2C19, CYP2D6, and CYP3A4 obtained from BD Biosciences. Compounds for CYP inhibition testing were prepared as 10 mM stock solutions in DMSO. Isoform-specific substrates were incubated at 37 °C individually with P450 enzymes (Table 5) and a range of test compound concentrations (1.1, 3.3 and 10 μM) in duplicate. Each isoform was tested separately with one reference compound, a known positive control inhibitor. During preincubation, the 96-well black plates were scanned with a fluorescence plate reader to eliminate false results originating from the autofluorescence of the test compounds. At the end of the incubation, product formation was monitored with fluorescence detection. A decrease in the formation of the metabolite compared to the “no inhibition” control samples was used to calculate the IC_50_ value.

### 4.5. Kinetic Solubility Assay

The kinetic solubility assay was performed to determine the solubility of the compounds in the in vitro assay conditions. The assay investigated the solubility based on the precipitation process in the HBSS buffer. For the preparation of the assay buffer, the following compounds were dissolved in 800 mL purified water:

− 130 mM sodium chloride, NaCl (7.600 g);
− 5.4 mM potassium chloride, KCl (0.403 g);
− 0.8 mM magnesium sulfate, MgSO_4_ × 7H_2_O (0.197 g);
− 0.9 mM sodium phosphate monobasic, NaH_2_PO_4_ × 2H_2_O (0.140 g);
− 25 mM D-glucose (4.500 g);
− 20 mM HEPES sodium salt (5.210 g).

The buffer was adjusted to pH 7.4, and the following compound was added to the mixture:

− 1.8 mM calcium chloride, CaCl_2_ × 2H_2_O (0.265 g).

The total volume was adjusted to 1000 mL.

Compounds for the kinetic solubility testing were prepared as 10 mM stock solutions in DMSO. The investigated compounds were diluted (in triplicate) with the HBSS buffer at pH 7.4 to a final concentration of 500 μM.

The mixture was shaken on a filter plate at 500 rpm for 1.5 h at RT. After incubation, the filter plate was placed inside a vacuum manifold and filtrated. Samples were collected and the concentration of the filtrate was determined spectrophotometrically by measuring the UV–VIS absorption spectrum. Concentrations were calculated based on equations resulting from the calibration curves (in duplicates, 8 calibration points, dilution Factor ×2, 500 µM → 3.91 µM, including blank sample).

### 4.6. Metabolic Stability

A metabolic stability assay was performed using a mouse microsomal fraction (phase I of metabolism) obtained from XenoTech (Kansas City, MO, USA). Compounds for metabolic stability testing were prepared as 10 mM stock solutions in DMSO. Compounds were incubated in triplicate at a 1 µM initial concentration with the microsomal fraction in the presence of NRS (NADPH-regenerating system, 1.3 mM). The final microsomal incubation (0.3 mg/mL) contained the components mentioned below in a pH 7.4 phosphate buffer. Control incubations were conducted without cofactors. The reference compound verapamil was used as a positive control. Incubations were carried out for 1 h on 96-well plates using a heated (+37 °C) orbital shaker (350 rpm). After incubation, the reaction was quenched through the addition of cold acetonitrile. The plate containing the samples was centrifuged at 2000 rpm for 15 min at 4 °C. The supernatants were analyzed using LC–MS.

#### Conditions of the Liquid Chromatography–Mass Spectrometry

The HPLC–MS consisted of a Dionex, Ultimate 3000 HPLC, and a Bruker Daltonics amaZon SL mass spectrometer (ESI-IT). The conditions were as follows: Column HPLC Ascentis Express C18 (5 cm × 2.1 mm, 2.7 µm) and an injection volume of 20 µL. An acetonitrile gradient was used with 0.1% formic acid in water at a constant flow rate of 1 mL/min. The programming was as follows: 0–0.5 min 5% ACN, 0.5–3.0 increase of 5–95%, 3.0–3.3 remaining at 95% ACN, 3.3–3.4 min decrease of 95–5% ACN, and 3.4–4.0 min 5% ACN. Samples were analyzed in a single-ion monitoring scan mode in positive mode. The conditions of the main parameters were as follows: a trap drive of 48.2, capillary exit voltage of 140.0 V, dry temperature (set) of 300 °C, nebulizer pressure (set) of 40.00 psi, dry gas flow (set) of 9.00 L/min HV capillary voltage of −4500 V, HV end-plate offset voltage of −500 V.

The percent loss of compounds was calculated through the normalization of the peak area against time 0, where the peak area at T_0_ was 100%. The elimination rate constant (h) was determined from the plot of ln (percent loss of compound) as a time function and equal to the minor value of the linear curve slope. The half-life time was calculated from the following equation:

$$T_{1/2}=\frac{0.693}{k}$$

The calculated half-life time was used for the intrinsic clearance calculation:

$$CL_{int}=\frac{V_{d}\times 0.693}{T_{1/2}}$$

where *V_d_*, the volume of distribution, equals:

$$V_{d}=\frac{1}{protein\;concentration\;\left(\frac{mg}{mL}\right)}$$

### 4.7. Behavioral Tests

#### 4.7.1. Animals

Male CD1 mice (Charles River, Sulzfeld, Germany) weighing 20–25 g at the time of arrival were used in the behavioral experiments. Animals were kept under standard laboratory conditions (12:12 light:dark cycle, 22 ± 2 °C) with free access to food and water. Animal welfare was regularly controlled by a veterinarian and animal welfare committee. After 2 weeks of acclimatization and handling, the experiments began. Experimental groups consisted of 4 to 10 animals, depending on the procedure. Drugs were administered intraperitoneally (*i.p.*) at a volume of 10 mL/kg. Experimental assessments were performed by an observer who was blinded to the treatment conditions. All procedures were conducted in accordance with the European Communities Council Directive of 22 September 2010 (2010/63/EU) and Polish legislation acts concerning animal experimentation, and were approved by the II Local Ethics Committee of the Maj Institute of Pharmacology, Polish Academy of Sciences, in Krakow (272/2019).

#### 4.7.2. Drugs

MK-801 was purchased from Tocris Bioscience, Bristol, UK. MK-801 was dissolved in 0.9% NaCl. Compounds 15 and 18 were dissolved in 0.9% NaCl. All compounds were administered intraperitoneally (*i.p.*) in a volume of 10 mL/kg. Vehicle-treated animals received appropriate solvents. The vehicle was administered to animals in any case when drug administration was omitted (e.g., control or MK-801-treated groups).

#### 4.7.3. DOI-Induced Head Twitch Test

The experiments were performed according to the procedure described in our previous studies [7,53,54]. Briefly, to habituate mice to the experimental environment, each animal was transferred to a 12 cm (diameter) × 20 cm (height) glass cage lined with sawdust 30 min before treatment. Test compounds were administered intraperitoneally (*i.p.*) at doses of 2.5, 5, and 10 mg/kg body weight 60 min before the test was performed. The head twitch response (HTR) in mice was induced using DOI (2.5 mg/kg, *i.p.*). Immediately after treatment, the number of head twitches was counted during a 20 min session.

#### 4.7.4. Novel Object Recognition Test

This procedure was adapted from [55] and performed as described in a previous paper [7]. Habituation, training and test trials were performed in a black plastic rectangular arena (40 × 30 × 35 cm) illuminated with a light intensity of 335 lux. During the habituation trial (2 consecutive days), each animal was allowed to explore the arena for 10 min. The next day, during the training trial (T1), mice were placed in the arena and were presented with two identical objects (red glass cylinders; 6.5 cm in diameter and 4.5 cm high) for 5 min. After 1 h, animals were placed back into the arena for a 5 min test trial, during which one of the previously presented familiar objects was replaced with a novel object (a transparent glass elongated sphere-like object with an orange cap; 5.5 cm in diameter and 8.5 cm high). Time spent exploring (i.e., sniffing or touching) the familiar (Tfamiliar) and novel (Tnovel) objects was measured by a trained observer, and the recognition index (%) was calculated for each mouse [(Tnovel − Tfamiliar)/(Tfamiliar + Tnovel)] “×” 100.

#### 4.7.5. MK-801-Induced Hyperactivity

The locomotor activity was recorded individually for each animal in locomotor activity cages [55,56] with modifications [50]. The mice were placed individually into activity cages (13 × 23 × 15 cm; Opto-M3; Columbus Instruments) for an acclimatization period of 30 min; then, they were injected *i.p.* with compound 15 (0.5, 1, 3 mg/kg) or compound 18 (0.05, 0.1, 0.5, 1, 3 mg/kg) and placed again in the same cages. After 30 min, all of the mice were injected *i.p.* with MK-801 at 0.35 mg/kg and once again placed in the same cage. From then on, the ambulation scores were counted for 60 min. All of the groups were compared with the MK-801 control group. The experiment also included a control group treated with NaCl only.

#### 4.7.6. Social Interaction Test

The method was adapted from de Moura Linck et al., 2008 [57], and Woźniak et al., 2016 [58]. After the 2-day habituation trial (10 min/day), a pair of mice was placed in the open field for 5 min. The social interactions between the two mice were determined based on the total time spent participating in social behavior, such as genital investigation, sniffing, chasing, and fighting each other. The total number of social episodes was also measured. The test was video-recorded and viewed by a trained observer. MMPIP (5, 10, and 20 mg/kg, *i.p*.) or ADX71743 (1, 5, and 15 mg/kg, *i.p.*) were administered 30 min before MK-801 (0.3 mg/kg, *i.p.*), which was administered 30 min before the test.

#### 4.7.7. Statistics

The statistical analysis was performed using GraphPad Prism v.9.1.0. The results of behavioral studies were analyzed using Student’s t-test (comparison vs control group), one-way ANOVA followed by Dunnett’s post hoc comparison or two-way ANOVA followed by Tukey’s post hoc comparison. Data are presented as mean ± SEM.

## Figures and Tables

**Table 1:** Structures and in vitro mGlu_7_ receptor NAM activity of selected 2,6-disubstituted(3*H*)-quinazolin-4-ones *^a^*.

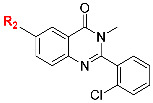			
ID	**R_2_**	Inhibition *^b^* (%)	mGlu_7_ IC_50_ *^c^* (µM)
**ALX-065**	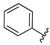	26.5 ± 11.8	4.65 ± 3.46
**A9-4**	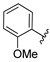	Inactive	
**A9-5**	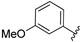	Inactive	
**A9-6**	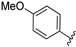	Inactive	
**ALX-171**	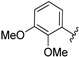	30.8 ± 6.44	6.14 ± 3.42
**A9-8**	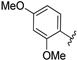	Inactive	
**A9-9**	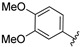	Inactive	
**A9-10**	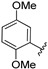	Inactive	
**A9-11**	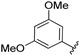	Inactive	
**A9-12**	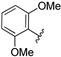	Inactive	

*^a^* All derivatives of the A9 chemotype depicted in Table S2, Supplementary Materials; *^b^* inhibition of the forskolin-stimulated (5 µM) cAMP response using the EC_80_ concentration of LSP4-2022, according to methodology described in previous papers [7,41]; *^c^* T-REx 293 cells expressing the human mGlu_7_ receptor (depicted in Section 2.1 of the Supplementary Materials); data are the mean of three independent experiments. The substances were incubated with 5 µM LSP4-2022 (EC_80_).

**Table 2:** Plasma (A) and brain (B) concentrations of ADX71743, MMPIP, **ALX-065,** and **ALX-171** after *i.p.* administration of 10 mg/kg (mouse, Albino Swiss).

	Parameters	ADX71743	MMPIP	**ALX-065**	**ALX-171**
(A)	T_max_ *^a^*	0.25	0.25	0.25	0.50
	T_1/2_ *^b^*	0.90	1.16	1.31	5.20
	C_max_ *^c^*	3.73	9.85	2.28	4.46
	AUC_0-t_ *^d^*	1.90	13.52	1.99	13.41
(B)	T_max_ *^a^*	0.25	0.50	0.25	0.25
	T_1/2_ *^b^*	0.34	1.75	0.49	5.56
	C_max_ *^c^*	3.38	5.42	1.81	2.52
	AUC_0-t_ *^d^*	2.27	8.98	1.32	13.85

*^a^* Time at maximum observed concentration (h); *^b^* terminal elimination half-life (h); *^c^* maximum drug concentration (µM/L); *^d^* area under the concentration–time curve from zero to the last measurable plasma concentration point (μM × h/L).

**Table 3:** Pharmacokinetic parameters for **ALX-171**. Plasma (A) and brain (B) concentrations of **ALX-171** in female BALB/c mice following intravenous (*i.v.*, 3 mg/kg) and peroral (*p.o.*, 5 mg/kg) administration.

	Administration	T_max_ *^a^*	T_1/2_ *^b^*	C_max_ *^c^*	AUC_0-t_ *^d^*	Vd *^e^*	F *^f^*
(A)	*i.v.*	0.25	2.26	1.59	174.75	7630	58
	*p.o.*	0.50	3.08	1.09	157.54	–	
(B)	*i.v.*	0.25	3.41	1.85	196.38	–	–
	*p.o*.	0.50	2.95	1.36	177.94	–	

*^a^* Time at maximum observed concentration (h); *^b^* terminal elimination half-life (h); *^c^* maximum drug concentration (µM/L); *^d^* area under the concentration–time curve from zero to the last measurable plasma concentration point (μM × h/L); *^e^* volume of distribution (mL/kg); *^f^* peroral bioavailability (%).

**Table 4:** Preliminary ADME results.

Parameters	ADX71743	MMPIP	**ALX-065**	**ALX-171**
M.W. *^a^*	269.14	333.35	346.81	406.87
clogP *^b^*	3.64	1.79	5.10	4.79
TPSA *^c^*	43.10	68.46	32.67	51.13
KS *^d^*	509.87	5.6	6.88	15.23
MML *^e^*	0.01	49.78	0.81	54.01
Clint *^f^*	500.14	38.74	267.64	34.22
Cytochrome P450 (IC_50_, μM) *^g^*				
1A2	>10	>10	1.1 < IC_50_ < 3.3	3.3 < IC_50_ < 10
3A4	>10	<1.1	>10	3.3 < IC_50_ < 10
2B6	>10	>10	>10	>10
2C19	3.3 < IC_50_ < 10	<1.1	1.1<IC_50_ < 3.3	3.3 < IC_50_ < 10
2D6	>10	>10	>10	>10

Physicochemical descriptors: *^a^* molecular weight; *^b^* calculated partition coefficient; *^c^* topological surface area calculated using ChemAxon software; *^d^* kinetic solubility in Hanks-HEPES (HBSS buffer) (µM); *^e^* metabolic stability liver microsome mouse (% remaining after 60 min); *^f^* ability of the body to eliminate a drug, expressed as a volume per unit of time (µL/min/mg); *^g^* inhibitor concentration which results in 50% inhibition of activity of cytochrome P450 isoenzymes; IC_50_ > 10 µM weak inhibition; 1.1 < IC_50_ < 3.3 µM moderate inhibition; 3.3 < IC_50_ < 10 µM mild inhibition; IC_50_ < 1.1 strong inhibition.

**Table 5:** Experimental conditions summary—cytochrome P450 IC_50_ inhibition determination assay.

P450 Isoform	Positive Control Highest Concentration (µM)	Substrate Concentration (µM)	Enzyme Concentration (nM)	Incubation Length (min)
CYP1A2	Furafylline 5	CEC *^a^* 5	5	15
CYP3A4	Ketoconazole 0.05	BFC *^b^* 50	5	30
CYP2B6	Tranylcypromine 50	EFC *^c^* 2.5	10	30
CYP2C19	Tranylcypromine 10	CEC *^a^* 25	10	30
CYP2D6	Quinidine 0.05	AMMC *^d^* 1.5	15	30

*^a^* cyano-7-ethoxycoumarin; *^b^* 7-benzyloxy-4-(trifluoromethyl) coumarin; *^c^* 7-ethoxy-4-(trifluoromethyl) coumarin; *^d^* 3-[2-(*N,N*-diethyl-*N*-methylamino)ethyl]-7-methoxy-4-methylcoumarin.

## Data Availability

Data is contained within the article or Supplementary Material.

## Supplementary Materials

The following supporting information can be downloaded at: https://www.mdpi.com/article/10.3390/ijms24031981/s1, syntheses [59,60,61,62,63,64,65,66,67,68,69,70,71] and characterization details for final products,^1^H and ^13^C NMR spectra, LC–MS purity, crystal data and structure results, in vitro methodology (T-REx 293, CHO-K1) [7,41], mGlu_4,8_ receptors selectivity.
