# Supplementary material for: Design and Synthesis of New Quinazolin-4-one Derivatives with Negative mGlu7 Receptor Modulation Activity and Antipsychotic-Like Properties
Source: Int J Mol Sci. 2023 Jan 19;24(3):1981. doi: 10.3390/ijms24031981 (PMC9916658; doi:10.3390/ijms24031981)
Supplement: Supplementary file 1 [file ijms-24-01981-s001.zip › ijms-2078472-supplementary.pdf]

Supplementary materials

Design and Synthesis of New Quinazolin-4-one Derivatives with Negative $mGlu_7$ Receptor Modulation Activity and Antipsychotic-Like Properties

1. CHEMISTRY ... **3**
1.1. OVERVIEW OF ALL THE STRUCTURES OF SYNTHESIZED FINAL COMPOUNDS ... 3
1.1.1. Table S1. Compounds of active chemotype A9 ... *3*
1.1.2. Table S2. Compounds of chemotypes: A1-A22 (except A9) ... *4*
1.2 SYNTHETIC PROCEDURES ... 7
1.2.1 CHEMOTYPE A9 ... 7
1.2.2. Chemotype A1 ... *37*
1.2.3. Chemotype A2 ... *38*
1.2.4. Chemotype A3 ... *42*
1.2.5. Chemotype A4 ... *45*
1.2.6. Chemotype A5 ... *47*
1.2.7. Chemotype A6 ... *49*
1.2.8. Chemotype A7 ... *51*
1.2.9. Chemotype A8 ... *52*
1.2.10. Chemotype A10 ... *54*
1.2.11. Chemotypes A11 and A12 ... *55*
1.2.12. Chemotypes A13 ... *60*
1.2.13. Chemotypes A14 ... *62*
1.2.14. Chemotypes A15 ... *64*
1.2.15. Chemotypes A16 ... *67*
1.2.16. Chemotypes A17 ... *69*
1.2.17. Chemotypes A18 ... *75*
1.2.18. Chemotypes A19 ... *77*
1.2.19. Chemotypes A20 ... *81*
1.2.20. Chemotypes A21 ... *83*
1.2.21. Chemotypes A22 ... *87*
1.4. CRYSTAL STRUCTURE DETERMINATION ... 92
2. IN VITRO PHARMACOLOGY ... **93**
2.1. NAM MGLU7 RECEPTOR ACTIVITY ON T-REX 293 CELL LINE ... 93
2.2. NAM MGLU7 RECEPTOR ACTIVITY ON CHO-K1 CELL LINE ACCORDING TO EUROSCREENFAST PROTOCOL 94
2.3. MGLU4 AND MGLU8 RECEPTOR SELECTIVITY ... 95
3. REFERENCES ... **97**

1. Chemistry

1.1. Overview of all the structures of synthesized final compounds

1.1.1. Table S1. Compounds of active chemotype A9

A9-1	A9-2	A9-3	A9-4
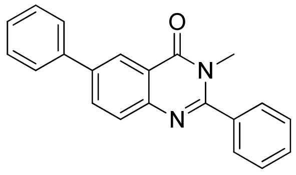	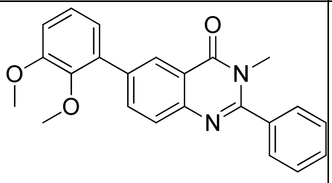	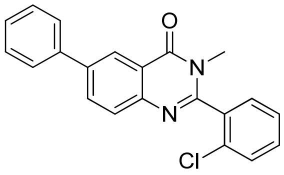	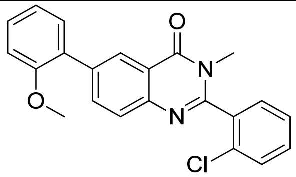
A9-5	A9-6	A9-7	A9-8
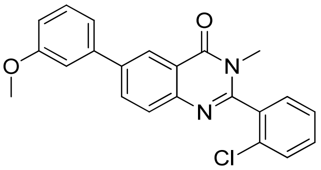	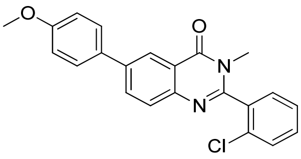	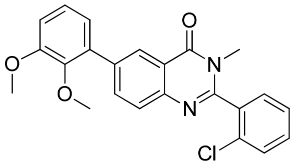	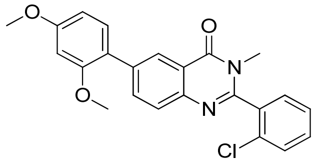
A9-9	A9-10	A9-11	A9-12
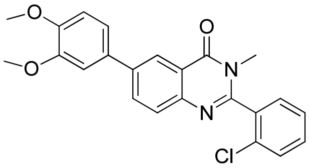	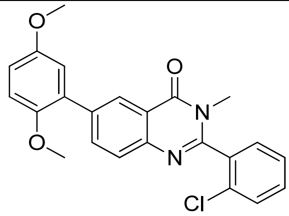	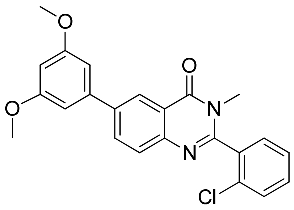	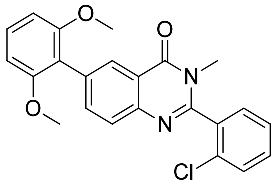
A9-13	A9-14	A9-15	A9-16
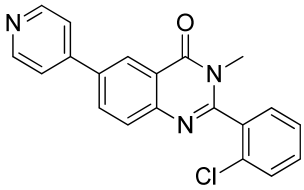	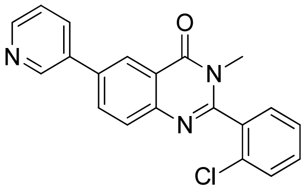	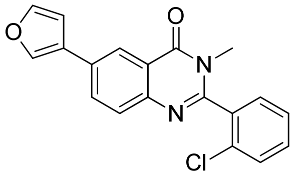	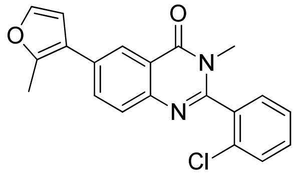
A9-17	A9-18	A9-19	A9-20
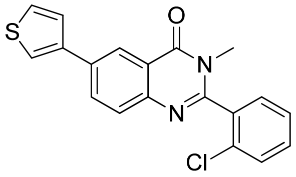	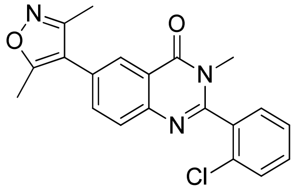	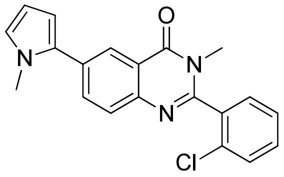	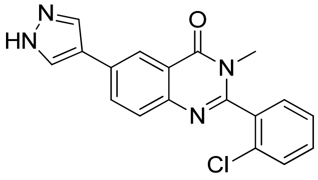
A9-21	A9-22	A9-23	A9-24
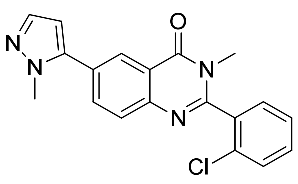	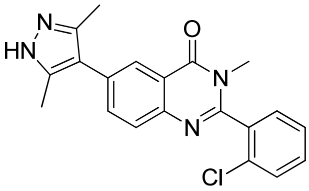	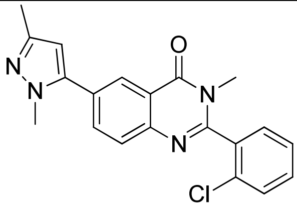	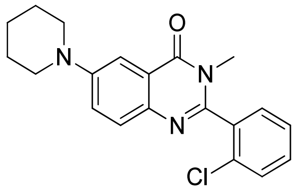

A9-25	A9-26	A9-27	A9-28
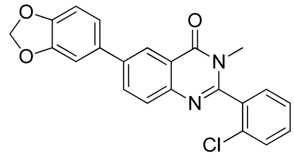	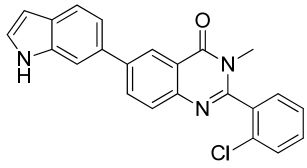	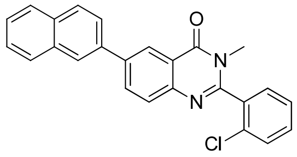	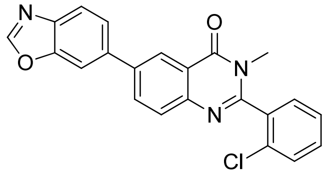
A9-29	A9-30	A9-31	A9-32
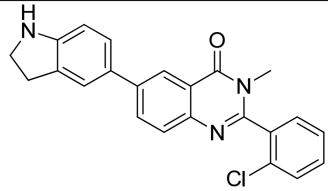	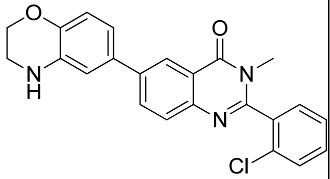	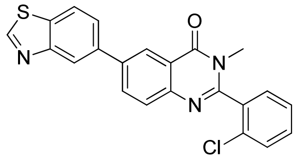	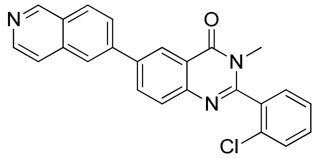
A9-33	A9-34	A9-35	A9-36
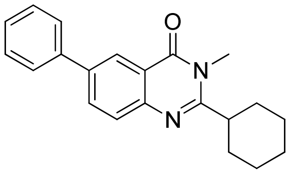	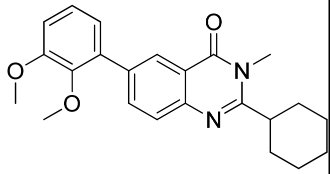	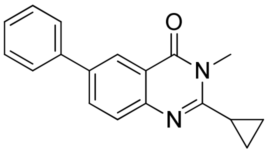	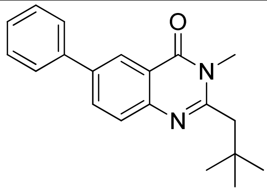

1.1.2. Table S2. Compounds of chemotypes: A1-A22 (except A9)

A1-1	A1-2	A2-1	A2-2
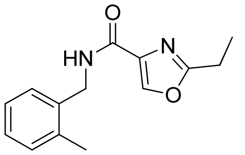	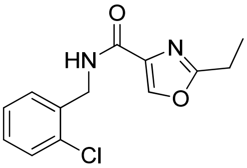	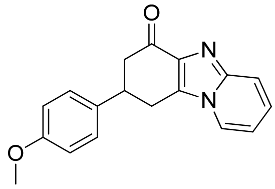	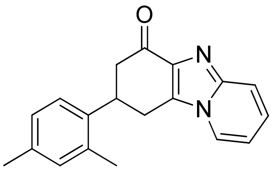
A3-1	A3-2	A3-3	A3-4
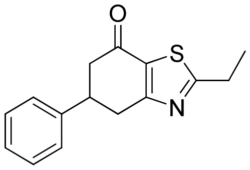	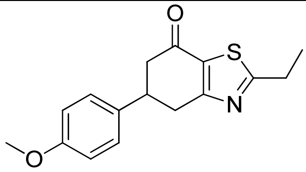	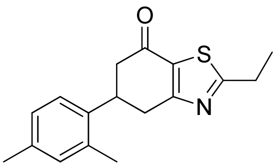	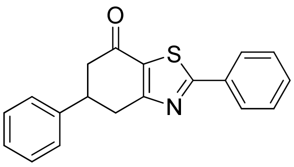
A3-5	A4-1	A4-2	A4-3
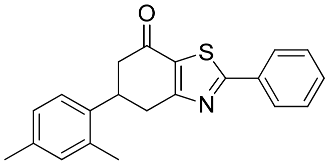	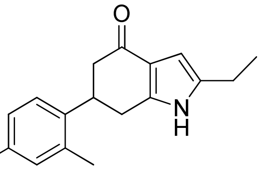	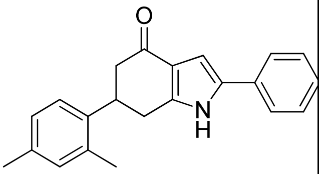	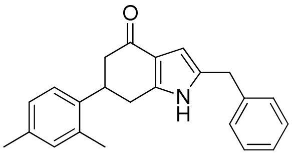
A5-1	A5-2	A6-1	A6-2

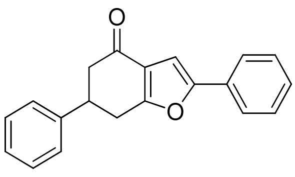	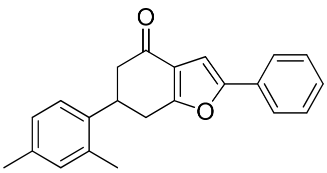	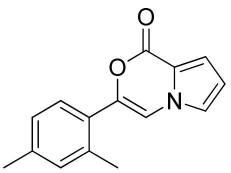	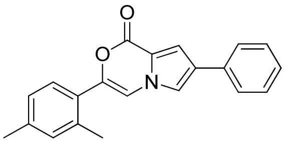
A6-3	A7-1	A8-1	A8-2
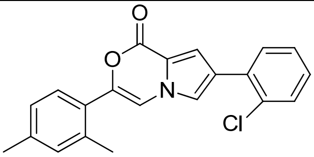	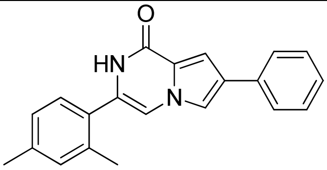	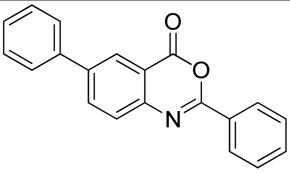	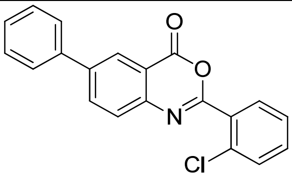
A8-3	A10-1	A11-1	A11-2
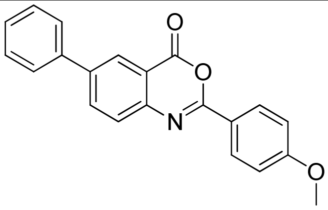	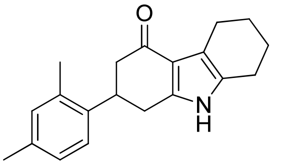	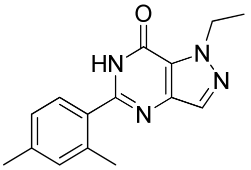	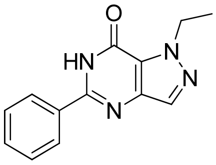
A11-3	A12-1	A12-2	A13-1
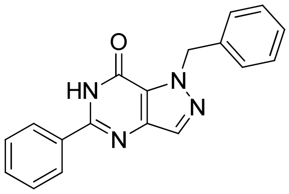	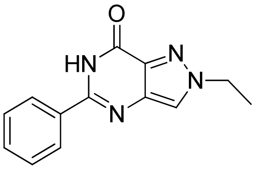	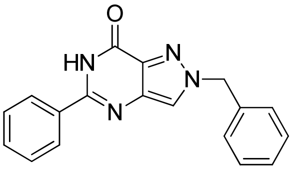	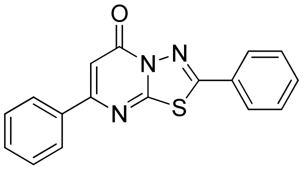
A13-2	A13-3	A14-1	A14-2
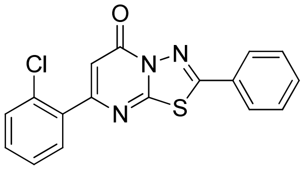	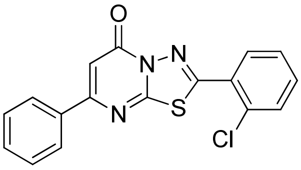	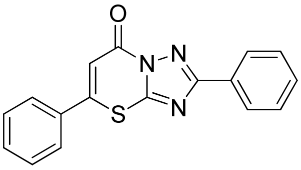	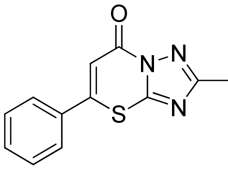
A14-3	A15-1	A15-2	A15-3
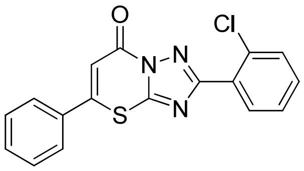	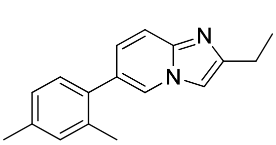	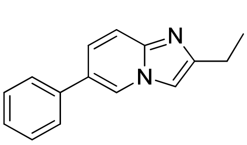	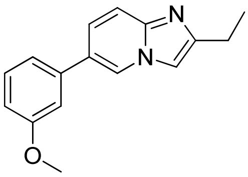
A15-4	A15-5	A16-1	A16-2
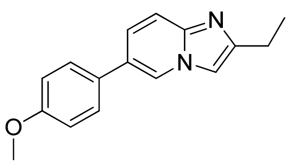	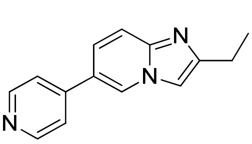	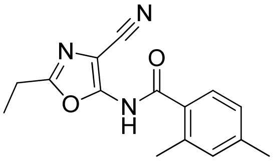	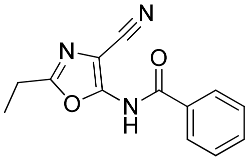

A17-1	A17-2	A17-3	A17-4
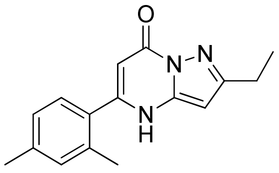	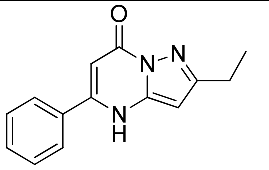	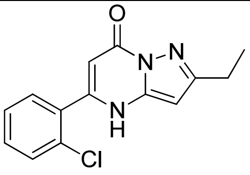	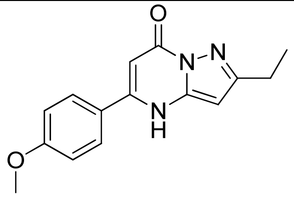
A17-5	A17-6	A17-7	A17-8
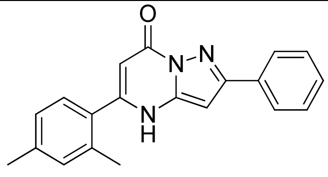	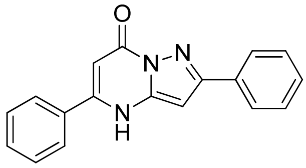	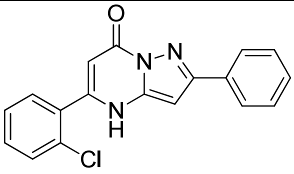	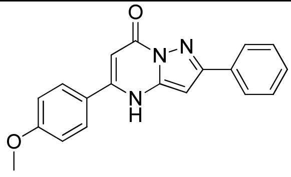
A18-1	A18-2	A18-3	A18-4
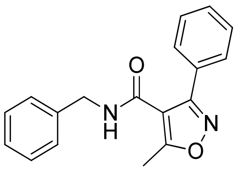	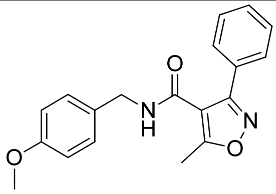	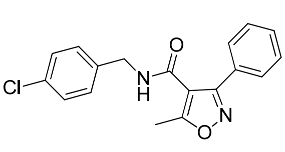	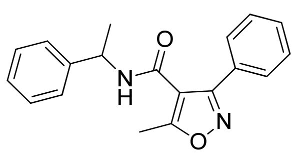
A19-1	A19-2	A19-3	A19-4
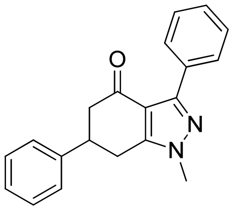	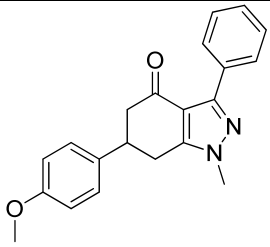	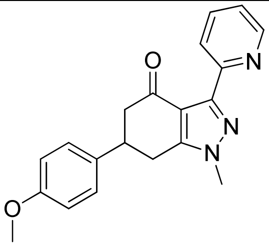	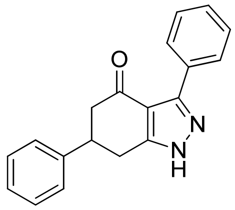
A20-1	A20-2	A21-1	A21-2
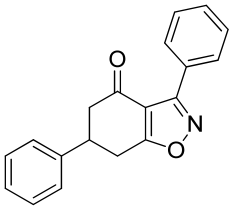	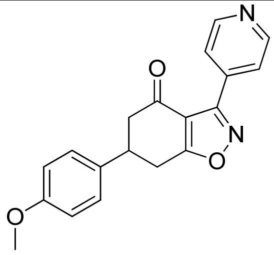	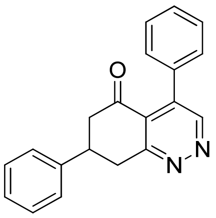	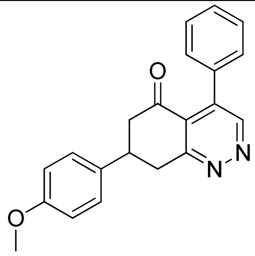
A21-3	A21-4	A21-5	A22-1
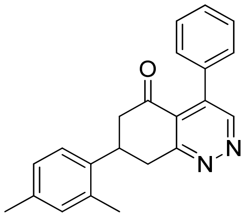	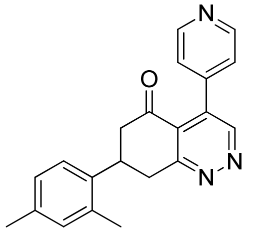	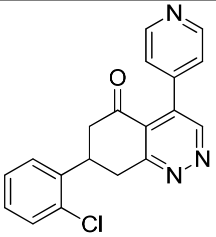	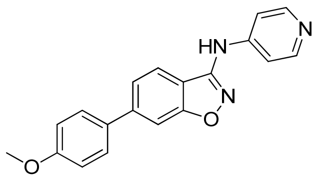

A22-2	A22-3	A22-4	
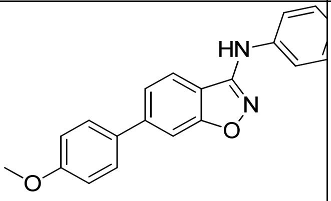	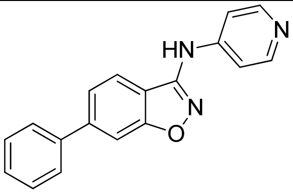	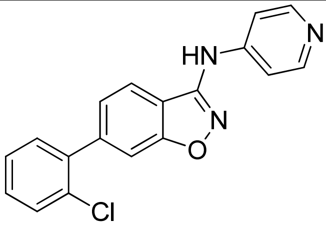	

1.2 Synthetic procedures

1.2.1. Chemotype A9

General procedure for the synthesis of A9 derivatives

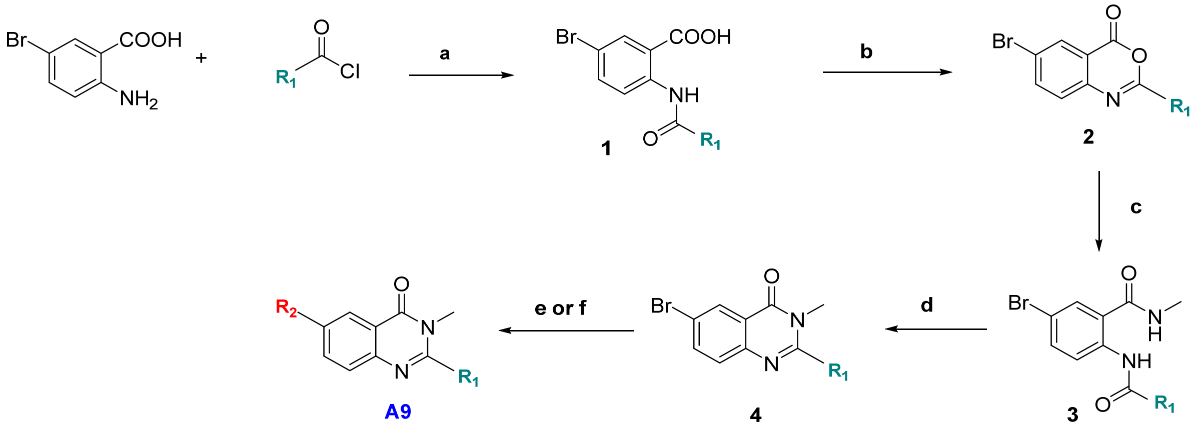

Procedure for the synthesis of intermediates **1** (step a)

Triethylamine (1.5 eq.) was slowly dropped to a stirred suspension of 2-amino-5-bromobenzoic acid (1.0 eq.) with appropriate acid chloride (0.9 eq.) in tetrahydrofuran (2 mL per 1g of compound 16) at 0–5 °C. Then the ice bath was removed and the reaction mixture was stirred at RT overnight. Then the mixture was diluted with ethyl acetate and quenched with water. The product was extracted thrice with ethyl acetate and the combined organic phases were dried over $MgSO_4$ and concentrated. The products **1** were sufficiently pure for the next step

Procedure for the synthesis of intermediates **2** (step b)

Compound **1** (1.0 eq) was suspended in acetic anhydride (20 mL per 1g of compound **1**) and heated under reflux for 3 h. After the reaction was completed, the mixture was cooled to RT, treated with 2-PrOH/hexane a 1:3 (v/v) mixture and stirred overnight at room temperature. The precipitate was suction filtered and rinsed with 2-PrOH/hexane a 1:3 (v/v) mixture and dried at 40 °C to give crude compound **2** which was used in next step without further purification.

Procedure for the synthesis of intermediates **3** (step c)

Compound **2** (6.62 mmol, 1.0 eq) was suspended in 15 mL of EtOAc, treated with 2M $MeNH_2$ in THF (7.28 mmol, 1.1 eq) and stirred at RT for 2 h. Next 10 mL of n-hexane was dropped and the resulting

suspension was stirred overnight at RT. The solid was filtratied and washed with hexane to yield pure compund **3** as a white solid.

Procedure for the synthesis of intermediates **4** (step d)

10 mL of 10% NaOH aq was slowly dropped into a stirring suspension of compound **3** (4.20 mmol), water (20 mL) and EtOH (5 mL). The reaction mixture was refluxed for 1 h, cooled down and left at RT overnight. The solid was filtered off, washed with hexane and purified *via* slurring with 2-PrOH/hexane a ratio of 1:3 (v/v) to provide the title compound **4** as a white solid.

Procedures for the synthesis **A9** derivatives (step e)

General procedure 1 - Suzuki coupling.

A mixture of compound **4** (1.0 eq), corresponding boronic acid (1.5 eq), potassium carbonate (3 eq.) and 2N Na_2CO_3 aq solution (2.0 eq) in 1,4-dioxane/toluene a ratio of 1:4 (v/v) (15 vol/1 g of compound 20) was degassed with argon and Pd(dppf)Cl_2 complex in DCM (0.05 eq.) was added. Reaction was run **under microwave irradiation** at 120 °C (20-40 min, 250 Watts), cooled down to RT, quenched with water and extracted with chloroform thrice. The combined organic layers were washed with brine, dried over $MgSO_4$ and evaporated. Crude product was purified by silica gel column chromatography using preferably chloroform: methanol a ratio of 99:1=>9:1 (v/v) or hexane/ethyl acetate a ratio of 2:1=>1:1 (v/v) as the eluates. Product usually was additionally triturated with 2-PrOH/hexane a 1:3 (v/v) mixture to yield the desirable **A9** product as a solid.

General procedure 2 - Suzuki coupling.

Compound **4** (1.0 eq), appropriate boronic acid (1.3 eq), K_2CO_3 (3.0 eq) and 2N Na_2CO_3 aq solution (2.0 eq) were suspended in 1,4-dioxane/toluene a ratio of 1:4 (v/v) (15 vol/1 g of compound **4**), degassed with argon and next Pd(dppf)Cl_2 complex in DCM (0.05 eq.) was added. Reaction was stirred in a sealed tube at 80 °C until all the substrate was converted (monitored with LC-MS). Then the reaction mixture was cooled down to RT, quenched with water and extracted thrice with chloroform. The combined layers were washed with brine, dried over $MgSO_4$ and evaporated. Product was purified by silica gel column chromatography using hexane/EtOAc a ratio of 3:1 => 2:1 (v/v) as an eluent, followed by trituration with 2-PrOH/hexane a 1:3 mixture to yield **A9** derivative.

General procedure 3 - Suzuki coupling.

Compound **4** (1.0 eq), appropriate boronic acid (1.3 eq), potassium phosphate tribasic (2.5 eq) were suspended in DMF/water a ratio of 4:3 (v/v) (10 vol/1 g of compound **4**), degassed with argon, and Pd(dppf)Cl_2 complex in DCM (0.10 eq) was added. Reaction was stirred in a sealed tube at 80 °C for 1-3 h, cooled down to RT, quenched with water and extracted with ethyl acetate (Note: to remove DMF it is necessary to apply into extraction a ratio of 3:1 (v/v) water/ethyl acetate). The combined organic layers were washed with brine, dried over $MgSO_4$ and evaporated. The crude product was purified via silica gel column chromatography eluted with hexane/ethyl acetate a ratio of 2:1=> 1:1 (v/v), followed by slurring with 2-PrOH/hexane a 1:3 (v/v) mixture to yield final **A9** derivative as a solid.

Procedure for the synthesis A9 derivatives (step f)

General procedure - Buchwald-Hartwig C-N bond formation.

Starting compound **4** (1.0 eq), corresponding amine (1.7 eq.) and K_3PO_4 (1.6 eq.) were suspended in dry toluene under inert Ar atmosphere (the reaction mixture was degassed with argon). Then DavePhos (0.05 eq) and $Pd_2(dba)_3$ (0.05 eq) were added. Reaction was run in a sealed tube for 4 days at 80 °C. Then the reaction mixture was cooled down and evaporated to dryness. The crude mixture was purified by silica gel column chromatography eluted with hexane/EtOAc a ratio of 2:1=> 1:1=> 1:2 (v/v), followed by trituration with 2-PrOH/hexane a 1:3 (v/v) mixture to yield A9 derivative as a solid.

Preparation of key intermediates 4

6-Bromo-3-methyl-2-phenylquinazolin-4(3*H*)-one (**4a**)

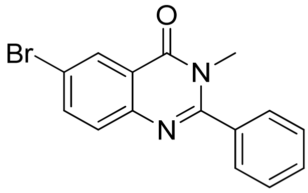

2-Benzamido-5-bromobenzoic acid (**1a**)

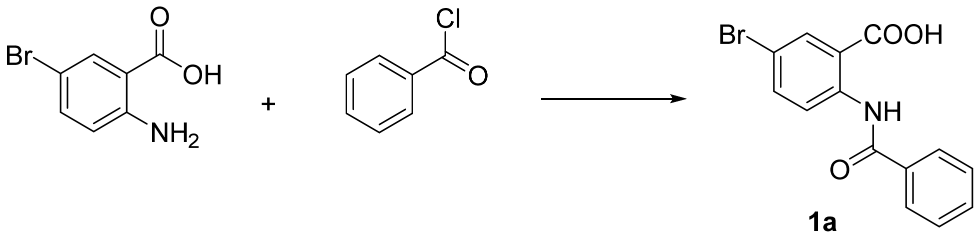

Triethylamine (3.51 g, 34.71 mmol, 1.5 eq.) was slowly dropped to a stirred suspension of 2-amino-5-bromobenzoic acid (5.0 g, 23.14 mmol, 1.0 eq.) and benzoyl chloride (2.93 g, 20.83 mmol, 0.9 eq.) in THF (100 mL) at 5 °C. The ice bath was removed and the reaction mixture was left to stir at RT overnight. The mixture was diluted with 100 mL of EtOAc and 100 mL of water. Layers were separated, the aqueous layer was extracted with EtOAc. Combined organic layers were washed with brine, dried over $MgSO_4$ and concentrated to yield crude 2-benzamido-5-bromobenzoic acid **1a** which was used without further purification (7.2 g, 97.0%).

LC-MS: 89.0% (Rt = 3.28), ESI(+) *m/z* found: 321.0 $[M+H]^+$. Molecular Weight calc'd for $C_{14}H_{10}BrNO_3$ = 320.14.

6-Bromo-2-phenyl-4*H*-benzo[*d*][1,3]oxazin-4-one (**2a**)

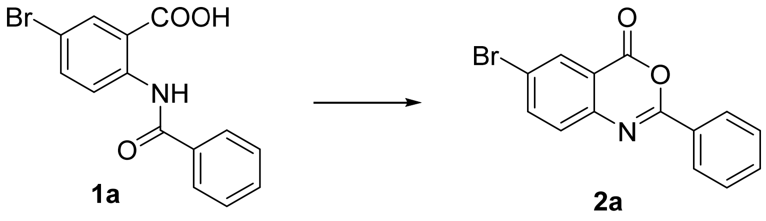

2-Benzamido-5-bromobenzoic acid **1a** (7.2 g, 24.5 mmol) was suspended in acetic anhydride (21.6 g, 0.21 mol) and heated under reflux for 3 h and then cooled to RT. The mixture was diluted with 30 mL of 2-PrOH/hexane a 1:3 (v/v) mixture and stirred overnight at room temperature. The solid was filtrated, washed with a 1:3 (v/v) and dried to give 6-bromo-2-phenyl-4H-benzo[*d*][1,3]oxazin-4-one (6.5 g, 95.6%). Crude compound **2a** was used in next step without further purification.

LC-MS: 100% (Rt = 3.77), ESI(+) *m/z* found: 303.0 $[M+H]^+$. Molecular Weight calc'd for $C_{14}H_8BrNO_2$ = 302.12.

^{1}H NMR (300 MHz, DMSO-d_6) δ ppm: 8.25 (dd, *J* = 2.3, 0.4 Hz, 1H), 8.21-8.19 (m, 2H), 8.10 (dd, *J* = 8.4, 1.3 Hz, 1H), 7.69-7.67 (m, 2H), 7.63-7.60 (m, 2H).

^{13}C NMR (75 MHz, DMSO-d_6) δ ppm: 157.4, 156.7, 145.3, 139.4, 132.9, 129.9, 129.8, 129.1, 127.8, 120.4, 118.9.

2-Benzamido-5-bromo-*N*-methylbenzamide (**3a**)

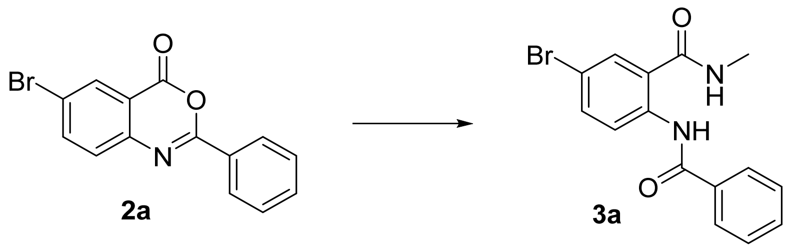

6-Bromo-2-phenyl-4*H*-benzo[*d*][1,3]oxazin-4-one **2a** (2.0 g, 6.62 mmol) was suspended in 15 mL of EtOAc, treated with 2M $MeNH_2$ in THF (0.226 g, 7.28 mmol) and stirred at RT for 2 h. 10 mL of n-hexane was dropped and the resulting suspension was stirred overnight at RT. The solid was filtratied and washed with hexane to yield pure 2-benzamido-5-bromo-*N*-methylbenzamide **3a** as a white solid (2.15 g, 97.7%).

LC-MS: 98.6% (Rt = 3.26), ESI(+) *m/z* found: 332.77 $[M+H]^+$. Molecular Weight calc'd for $C_{15}H_{13}BrN_2O_2$ = 333.18.

^{1}H NMR (300 MHz, $CDCl_3$) δ ppm: 11.99 (s br., 1H, NHCO), 8.60 (d, *J* = 8.9 Hz, 1H), 8.00-7.98 (m, 2H), 7.60 (d, *J* = 2.3 Hz, 1H), 7.55-7.48 (m, 4H), 6.85 (d br, 1H, NHCO), 2.99 (d, *J* = 4.8 Hz, 3H, CH_3).

^{13}C NMR (75 MHz, $CDCl_3$) δ ppm: 168.5, 165.7, 138.5, 135.0, 134.2, 132.1, 129.6, 128.8, 127.3, 123.1, 122.4, 115.3, 26.9.

6-bromo-3-methyl-2-phenylquinazolin-4(3*H*)-one (**4a**)

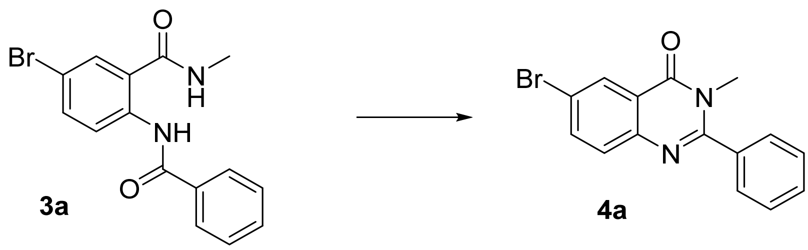

10 mL of 10% NaOH aq was slowly dropped into a stirring suspension of 2-benzamido-5-bromo-*N*-methylbenzamide (**3a**) (1.4 g, 4.20 mmol), water (20 mL) and EtOH (5 mL). The reaction mixture was refluxed for 1 h, cooled down and left at room temperature overnight. The solid was filtered off, washed with hexane and purified *via* slurring with 2-PrOH/hexane a 1:3 (v/v) mixture to provide the title compound 6-bromo-3-methyl-2-phenylquinazolin-4(3*H*)-one (**4a**) as a white solid (1.08 g, 81.8%).

LC-MS: 100% (Rt = 3.26), ESI(+) *m/z* found: 316.80 $[M+H]^+$. Molecular Weight calc'd for $C_{15}H_{11}BrN_2O$ = 315.16.

^{1}**H NMR**(300 MHz, $CDCl_3$) δ ppm: 8.46 (d, *J* = 2.2 Hz, 1H), 7.83 (dd, *J* = 8.7, 2.3 Hz, 1H), 7.61 (d, *J* = 8.7 Hz, 1H), 7.57-7.53 (m, 5H), 3.50 (s, 3H, CH_3).

^{13}**C NMR** (75 MHz, $CDCl_3$) δ ppm: 161.6, 156.5, 146.2, 137.5, 135.1, 130.3, 129.4, 129.2, 128.9, 128.0, 121.9, 120.5, 34.5.

6-Bromo-2-(2-chlorophenl)-3-methylquinazolin-4(3*H*)-one (**4b**)

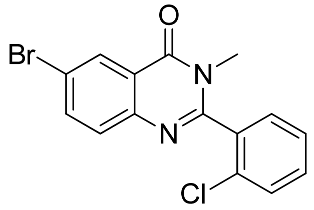

5-Bromo-2-(2-chlorobenzamido)benzoic acid (**1b**)

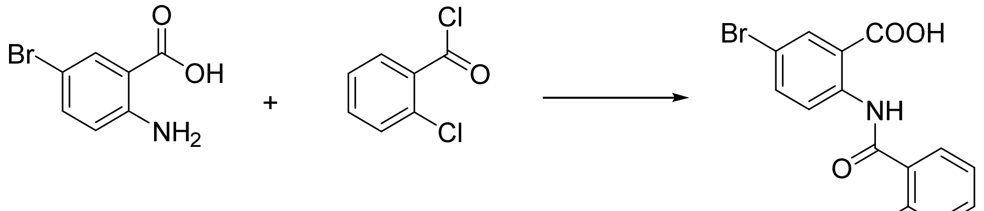

1b

Triethylamine (4.21 g, 41.65 mmol) was slowly dropped to a stirred suspension of 2-amino-5-bromobenzoic acid (6.0 g, 27.77 mmol) and 2-chlorobenzoyl chloride (4.42 g, 25.26 mmol) in THF (300 mL) at 5 °C. The ice bath was removed and the reaction mixture was left to stir at RT overnight. The mixture was diluted with 100 mL of EtOAc and 100 mL of water. Layers were separated, the aqueous layer was extracted with EtOAc. Combined organic layers were washed with brine, dried over $MgSO_4$ and concentrated to yield 5-bromo-2-(2-chlorobenzamido)benzoic acid (**1b**) as a light yellow solid (8.0 g, 81.2%) which was used in next step without further purification.

LC-MS: 83.5% (Rt = 3.29), ESI(+) *m/z* found: 355.94 $[M+H]^+$. Molecular Weight calc'd for $C_{14}H_9BrClNO_3$ = 354.58.

6-Bromo-2-(2-chlorophenyl)-4*H*-benzo[*d*][1,3]oxazin-4-one (**2b**)

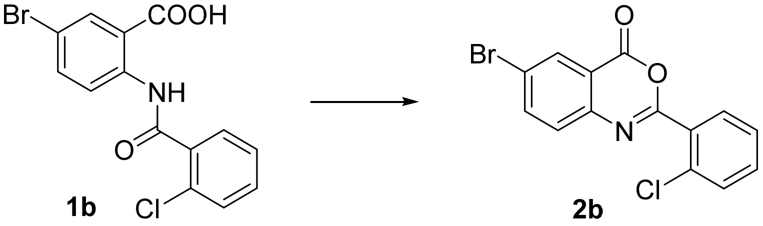

A suspension of 5-bromo-2-(2-chlorobenzamido)benzoic acid (**1b**) (8.0 g, 22.56 mmol) and acetic anhydride (21.6 g, 0.21 mol) was heated under reflux for 3 h and cooled down to RT. The mixture was diluted with 20 mL of the mixture 2-PrOH/hexane (1:3) and stirred overnight at RT. The solid was filtered, washed with 2-PrOH/hexane a 1:3 (v/v) mixture and dried to give 6-bromo-2-(2-chlorophenyl)-4*H*-benzo[*d*][1,3]oxazin-4-one **2b** as a white solid (6.35 g, 83.7%).

LC-MS: 100% (Rt = 3.74), ESI(+) *m/z* found: 337.87 $[M+H]^+$. Molecular Weight calc'd for $C_{14}H_7BrClNO_2$ = 336.57.

^{1}**H NMR** (300 MHz, DMSO-d_6) δ ppm: 8.27 (dd, *J* = 2.4, 0.3 Hz, 1H), 8.13 (dd, *J* = 8.6, 2.4 Hz, 1H), 7.93 (ddd, *J* = 7.8, 1.6, 0.5 Hz, 1H), 7.68 (dd, *J* = 8.6, 0.4 Hz, 1H), 7.68-7.62 (m, 2H), 7.55 (ddd, *J* = 7.7, 7.0, 1.6 Hz, 1H).

^{13}**C NMR** (75 MHz, DMSO-d_6) δ ppm: 157.6, 156.1, 144.9, 139.7, 133.1, 131.9, 130.8, 130.0, 129.8, 129.3, 127.6, 121.4, 118.8.

5-Bromo-2-(2-chlorobenzamido)-*N*-methylbenzamide (**3b**)

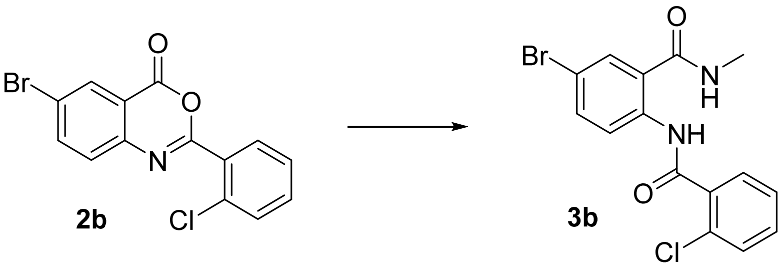

6-Bromo-2-(2-chlorophenyl)-4*H*-benzo[*d*][1,3]oxazin-4-one **2b** (5.85 g, 17.38 mmol) was suspended in 100 mL of EtOAc, treated with 2M $MeNH_2$ in THF (2.48 g, 80.0 mmol) and stirred overnight at RT. The solvent was evaporated and product was precipitated from 2-PrOH/hexane a 1:3 (v/v) mixture to yield the desired 5-bromo-2-(2-chlorobenzamido)-*N*-methylbenzamide **3b** as a white solid (5.95 g, 93.1%) which was used in next step without further purification.

LC-MS: 100% (Rt = 3.23), ESI(+) *m/z* found: 368.93 $[M+H]^+$. Molecular Weight calc'd for $C_{15}H_{12}BrClN_2O_2$ = 367.62.

^{1}**H NMR** (300 MHz, $CDCl_3$) δ ppm: 11.45 (s br, 1H), 8.66 (d, *J* = 8.9 Hz, 1H), 7.63-7.61 (m, 2H), 7.59 (dd, *J* = 8.9, 2.3 Hz, 1H), 7.44 (dd, *J* = 7.8, 1.4 Hz, 1H), 7.38 (dt, *J* = 7.8, 1.8 Hz, 1H), 7.34 (dt, *J* = 7.4, 1.4 Hz, 1H), 6.59 (d br, 1H), 2.91 (d, *J* = 4.8 Hz, 3H).

^{13}**C NMR** (75 MHz, $CDCl_3$) δ ppm: 168.1, 165.5, 138.1, 135.7, 135.2, 131.6, 131.3, 130.7, 129.5, 129.3, 127.2, 123.3, 122.7, 115.8, 26.9.

6-Bromo-2-(2-chlorophenyl)-3-methylquinazolin-4(3*H*)-one (**4b**)

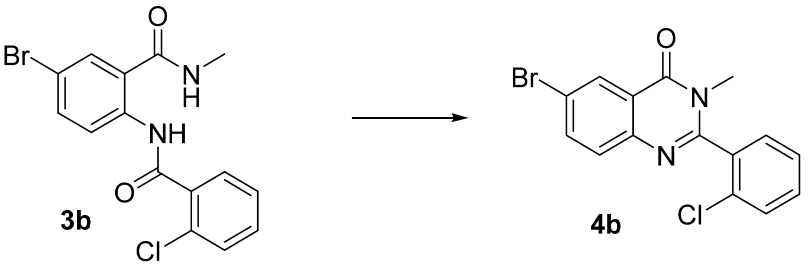

3b **4b**

To a stirred suspension of 5-bromo-2-(2-chlorobenzamido)-*N*-methylbenzamide (**3b**) (5.95 g, 16.27 mmol) in 100 mL of EtOH, KOH (2.72 g, 48.48 mmol) was added in portions. The resulting mixture was heated under reflux for 12 h, cooled to RT and concentrated. The residue was treated with a mixture of EtOH/water a 1:1 (v/v) mixture, the solid was filtered and dried yielded 6-bromo-2-(2-chlorophenyl)-3-methylquinazolin-4(3*H*)-one (**4b**) as a white solid (5.09 g 90.0%).

LC-MS: 98.9% (Rt = 3.39), ESI(+) *m/z* found: 351.0 $[M+H]^+$. Molecular Weight calc'd for $C_{15}H_{10}BrClN_2O$ = 349.61.

^{1}**H NMR** (300 Hz, $CDCl_3$) δ ppm: 8.48 (dd, *J* = 2.3, 0.4 Hz, 1H), 7.84 (dd, *J* = 8.7, 2.3 Hz, 1H), 7.62 (dd, *J* = 8.7, 0.4 Hz, 1H), 7.54-7.52 (m, 1H), 7.51-7.46 (m, 3H), 3.41 (s, 3H).

^{13}**C NMR** (75 MHz, $CDCl_3$) δ ppm: 161.0, 154.1, 146.1, 137.6, 134.4, 132.2, 131.4, 130.0, 129.4, 129.3, 127.7, 122.2, 120.9, 32.6.

6-Bromo-2-cyclohexyl-3-methylquinazolin-4(3*H*)-one (**4c**)

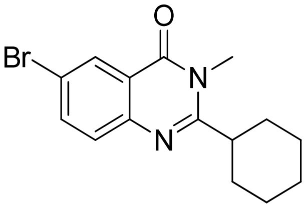

6-bromo-2-cyclohexyl-3-methylquinazolin-4(3*H*)-one (**4c**) was prepared according to procedures described for intermediate **4b**. Yields: **1c** (quantitative), **2c** (88%), **3c** (77%), **4c** (47%).

LC-MS: 100% (Rt = 3.83), ESI(+) *m/z* found: 322.88 $[M+H]^+$. Molecular Weight calc'd for $C_{15}H_{17}BrN_2O$ = 321.21.

^{1}**H NMR** (300 Hz, $CDCl_3$) δ ppm: 8.36 (dd, *J* = 2.3, 0.4, 1H, ArH), 7.76 (dd, *J* = 8.7, 2.3, 1H, ArH), 7.62 (dd, *J* = 8.7, 0.4, 1H, ArH), 3.35 (s, 3H, NCH_3), 2.82-2.78 (m, 1H, ArCH), 1.95-1.90 (m, 4H, CH_2), 1.78-1.72 (m, 3H, CH_2), 1.42-1.36 (m, 3H, CH_2).

^{13}**C NMR** (75 Hz, $CDCl_3$) δ ppm: 161.7, 160.8, 146.3, 137.0, 129.1, 129.0, 121.6, 119.5, 42.4, 31.0, 30.2, 26.1, 25.8.

6-Bromo-2-cyclopropyl-3-methylquinazolin-4(3*H*)-one (**4d**)

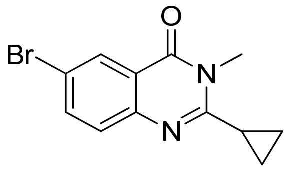

6-bromo-2-cyclopropyl-3-methylquinazolin-4(3*H*)-one (**4d**) was prepared according to procedures described for intermediate **4b.** Yields: **1d** (quantitative), **2d** (80%), **3d** (90%), **4d** (58%).

LC-MS: 100% (Rt = 3.05) ESI(+) *m/z* found: 280.02 $[M+H]^+$. Molecular Weight calc'd for $C_{12}H_{11}BrN_2O$ = 279.13.

^{1}**H NMR** (300 Hz, $CDCl_3$) δ ppm: 8.36 (d, *J* = 2.3, 1H, ArH), 7.74 (dd, *J* = 8.7, 2.3, 1H, ArH), 7.43 (d, *J* = 8.7, 1H, ArH), 3.78 (s, 3H, NCH_3), 2.03-2.00 (m, 1H, ArCH), 1.28-1.25 (m, 2H, CH_2), 1.13-1.10 (m, 2H, CH_2).

^{13}**C NMR** (75 Hz, $CDCl_3$) δ ppm: 161.4, 158.2, 146.3, 137.1, 129.2, 128.7, 121.6, 119.2, 30.5, 14.6, 8.6.

6-Bromo-3-methyl-2-neopenthylquinazolin-4(3*H*)-one (**4e**)

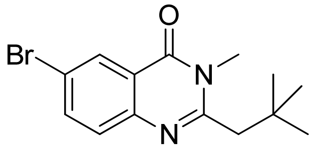

6-bromo-3-methyl-2-neopenthylquinazolin-4(3*H*)-one (**4e**) was prepared according to procedures described for intermediate **4b.** Yields: **1e** (85%), **2e** (73%), **3e** (42%), **4e** (76%).

LC-MS: 96.0% (Rt = 3.57), ESI(+) *m/z* found: 310.01 $[M+H]^+$. Molecular Weight calc'd for $C_{14}H_{17}BrN_2O$ = 309.20.

^{1}**H NMR** (300 Hz, $CDCl_3$) δ ppm: 8.38 (d, *J* = 2.3, 1H, ArH), 7.78 (dd, *J* = 8.7, 2.3, 1H, ArH), 7.52 (d, *J* = 8.7, 1H, ArH), 3.65 (s, 3H, NCH3), 2.78 (s, 2H, ArCH2), 1.13 (s, 9H, $(CH3)_3$).

^{13}**C NMR** (75 Hz, $CDCl_3$) δ ppm: 161.6, 156.2, 145.7, 137.2, 129.2, 128.9, 121.5, 119.7, 46.8, 33.2, 32.2, 30.1.

Preparation of **A9** derivative

3-Methyl-2,6-diphenylquinazolin-4(3*H*)-one **A9-1** (ALX-063)

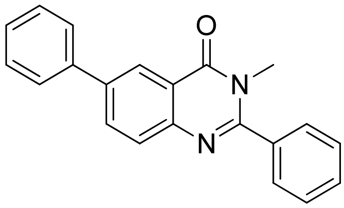

A mixture of 6-bromo-3-methyl-2-phenylquinazolin-4(3*H*)-one (**4a**) (150 mg, 0.48 mmol), phenylboronic acid (90.0 mg, 0.72 mmol), potassium carbonate (200 mg, 1.44 mmol, 3 eq.) and 1 mL of 2N Na_2CO_3 aq solution in 4 mL of toluene and 1 mL of 1,4-dioxane was degassed with argon and $Pd(dppf)Cl_2$ complex in DCM (20 mg, 0.024 mmol, 0.05 eq.) was added. Reaction was run under

microwave irradiation at 120 °C for 20 min, cooled down to RT and diluted with $CHCl_3$ (50 mL) and water (30 mL). The layers were separated, the aqueous layer was extracted with $CHCl_3$ (3x). Combined organic layers were washed with brine, dried over $MgSO_4$ and evaporated to give 3-methyl-2,6-diphenylquinazolin-4(3*H*)-one as an dark oily product. Crude product was purified by silica gel column chromatography ($CHCl_3$), followed by slurring with 2-PrOH/hexane a 1:3 (v/v) mixture to yield the desirable product as an off-white solid (98 mg, 65.8%).

LC-MS: 95.5% (RT = 3.34), ESI(+) *m/z* found: 313.21 $[M+H]^+$. Molecular Weight calc'd for $C_{21}H_{16}N_2O$ = 312.36.

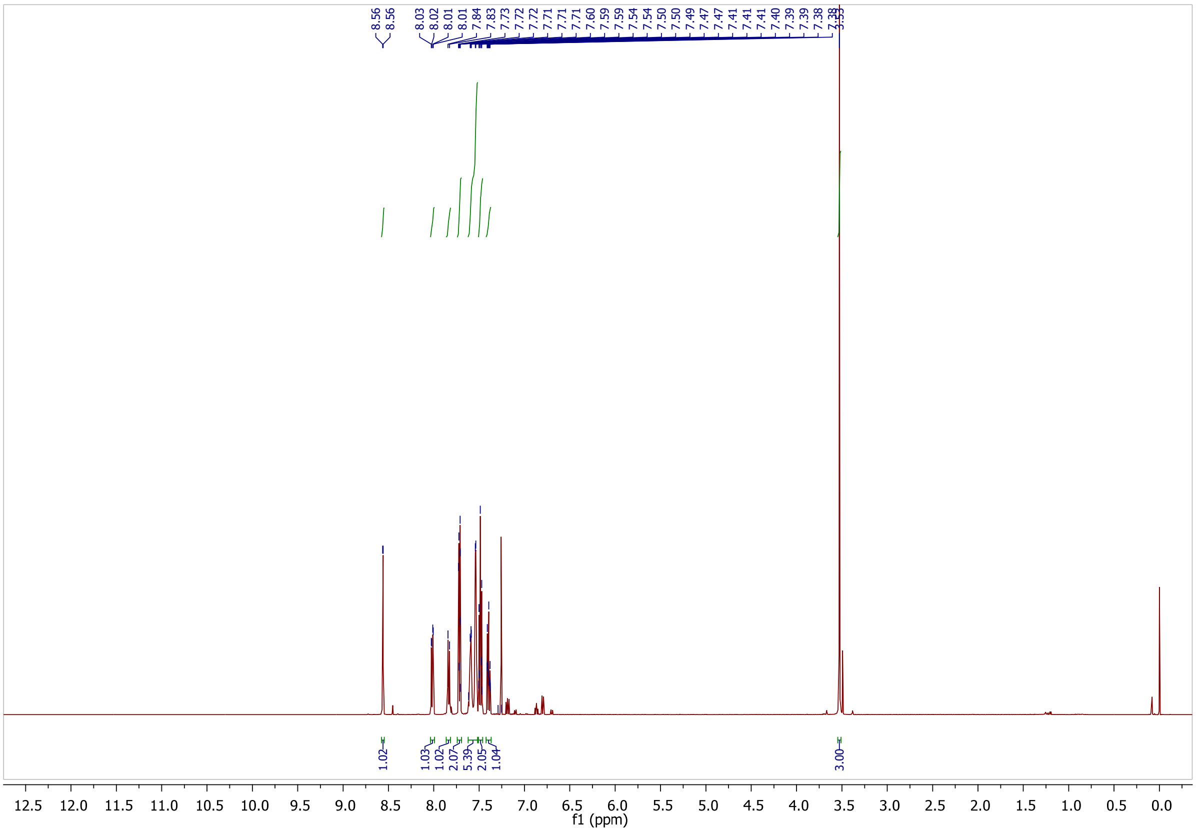

^{1}H NMR (300 Hz, $CDCl_3$) δ ppm: 8.56 (d, *J* = 2.1 Hz, 1H), 8.02 (dd, *J* = 8.4, 2.2 Hz, 1H), 7.83 (d, *J* = 8.5 Hz, 1H), 7.73-7.71 (m, 2H), 7.60-7.54 (m, 5H), 7.50-7.47 (m, 2H), 7.41-7.38 (m, 1H), 3.53 (s, 3H). *Traces of impurities visible on the spectrum.*

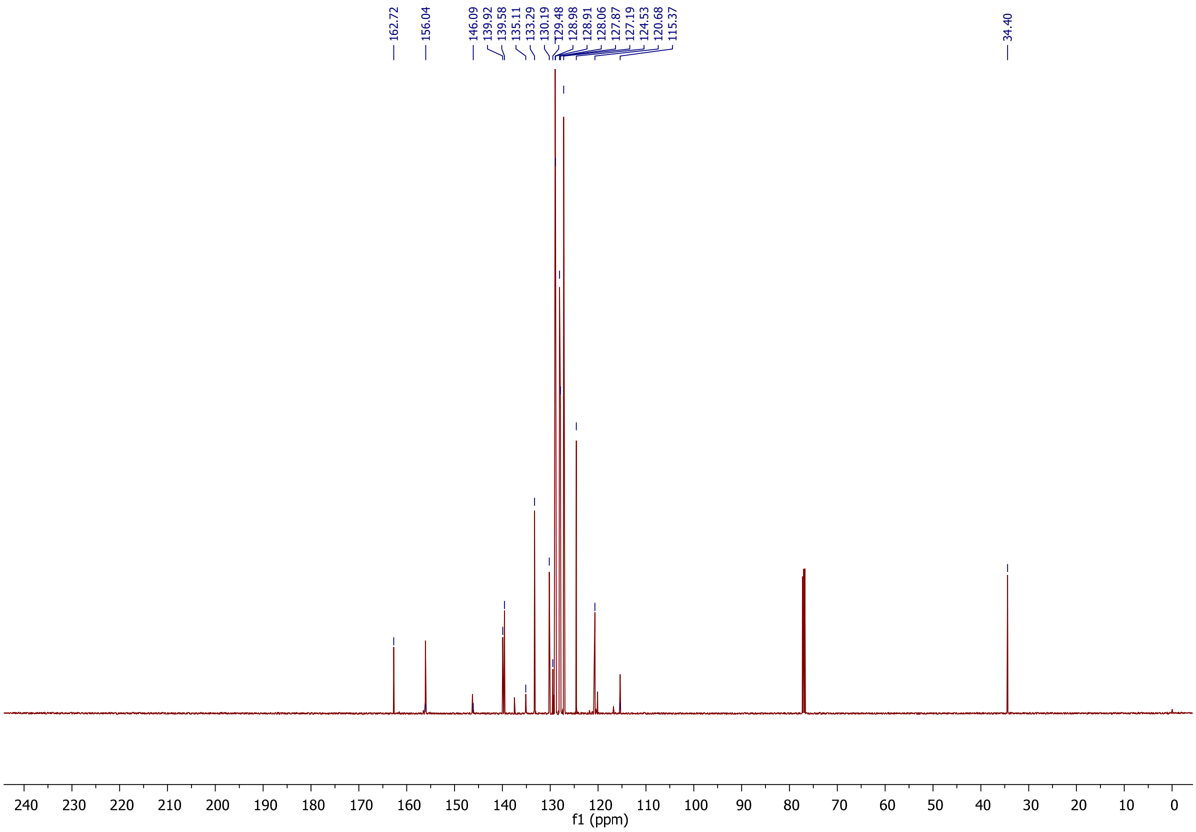

^{13}C NMR (75 Hz, $CDCl_3$) δ ppm: 162.7, 156. 0, 146.1, 139.9, 139.6, 135.1, 133.3, 130.2, 129.5, 129.0, 128.9, 128.1, 127.9, 127.2, 124.5, 120.7, 115.4, 34.4.

6-(2,3-Dimethoxyphenyl)-3-methyl-2-phenylquinazolin-4(3*H*)-one **A9-2**

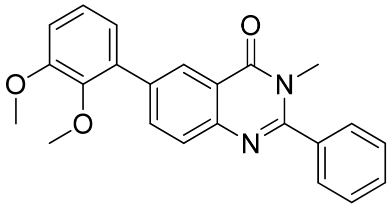

Prepared from 6-bromo-3-methyl-2-phenylquinazolin-4(3*H*)-one (**4a**) (170 mg, 0.54 mmol), 2,3-dimethoxyphenylboronic acid (128 mg, 0.70 mmol), potassium phosphate tribasic (290 mg, 1.36 mmol), Pd(dppf)Cl_2 complex in DCM (40 mg, 0.05 mmol) in 4 mL of DMF and 3 mL of water. Reaction was run in a sealed tube at 80 °C/1 h, cooled down to RT and diluted with EtOAc (60mL) and water (20mL). The layers were separated, the aqueous layer was extracted with EtOAc (3x). Combined organic layers were washed with brine, dried over $MgSO_4$, evaporated and crude product was purified by silica gel column chromatography (hexane-EtOAc, a ratio of 2:1=> 1:1), followed by slurring with 2-PrOH/hexane a 1:3 (v/v) mixture to yield 6-(2,3-dimethoxyphenyl)-3-methyl-2-phenylquinazolin-4(3*H*)-one **A9-2** as an off-white solid (118 mg, 59.0%).

LC-MS: 100% (Rt = 3.39), ESI(+) *m/z* found: 373.00 [M+H]+. Molecular Weight calc'd for $C_{23}H_{20}N_2O_3$ = 372.15.

^{1}**H NMR** (300 Hz, $CDCl_3$) δ ppm: 8.48 (d, *J* = 1.8, 1H), 8.03 (dd, *J* = 8.4, 2.1, 1H), 7.78 (d, *J* = 8.4, 1H), 7.60-7.53 (m, 5H), 7.16 (t, *J* = 7.9, 1H), 7.06 (dd, *J* = 7.8, 1.5, 1H), 6.98 (dd, *J* = 8.2, 1.5, 1H), 3.93 (s, 3H), 3.63 (s, 3H), 3.52 (s, 3H).

^{13}**C NMR** (75 Hz, $CDCl_3$) δ ppm: 162.8, 156.1, 153.2, 146.7, 146.4, 137.4, 135.9, 135.5, 134.6, 130.1, 128.9, 128.0, 127.1, 126.8, 124.3, 122.6, 120.4, 112.2, 60.7, 56.0, 34.3.

2-(2-Chlorophenyl)-3-methyl-6-phenylquinazolin-4(3*H*)-one **A9-3** (ALX-065)

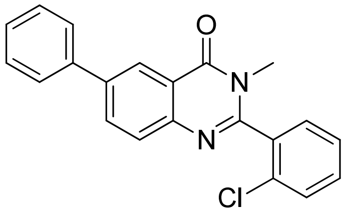

Prepared from 6-bromo-2-(2-chlorophenyl)-3-methylquinazolin-4(3*H*)-one (**4b**) (150 mg, 0.48 mmol), phenylboronic acid (150 mg, 0.429 mmol), K_2CO_3 (180 mg, 1.30 mmol) and 1 mL of 2N Na_2CO_3 aq solution in 4 mL of toluene and 1 mL of 1,4-dioxane in the presence of Pd(dppf)Cl_2 complex in DCM (18 mg, 0.022 mmol) according to general procedure 1. Crude product was purified by silica gel column chromatography eluted with hexane/ethyl acetate a ratio of 3:1=>2:1 (v/v), followed by trituration with with 2-PrOH/hexane a 1:3 (v/v) mixture to yield 2-(2-chlorophenyl)-3-methyl-6-phenylquinazolin-4(3H)-one **A9-3** as an off-white solid (98 mg, 65.8%).

LC-MS: 100% (Rt = 3.53), ESI(+) *m*/*z* found: 346.70$[M+H]^+$. Molecular Weight calc'd for $C_{21}H_{15}ClN_2O$ = 346.81.

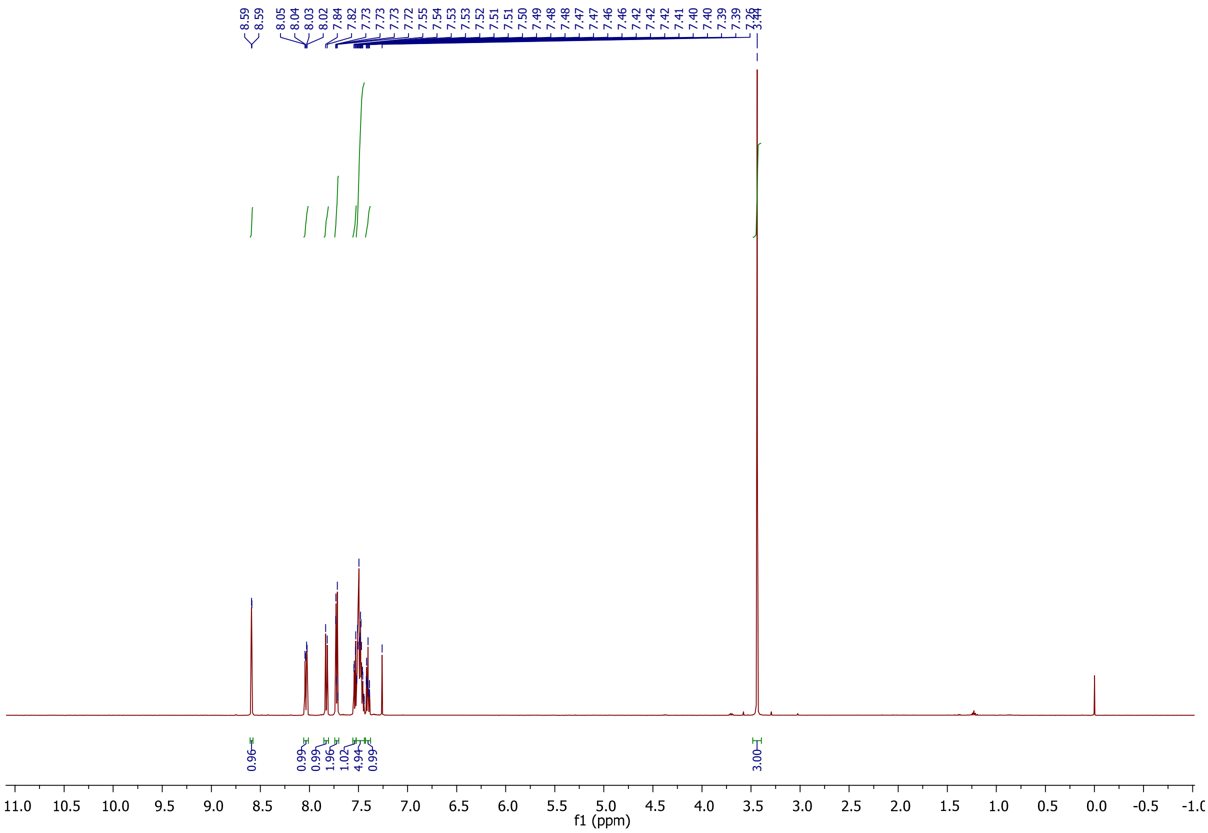

^{1}H NMR (300 Hz, $CDCl_3$) δ ppm: 8.59 (*d*, J = 2.1 Hz, 1H), 8.04 (*dd*, J = 8.4, 2.2 Hz, 1H), 7.83 (*d*, J = 8.4 Hz, 1H), 7.73-7.72 (m, 2H), 7.54 (*dd*, J = 7.4, 1.8 Hz, 1H), 7.52-7.44 (m, 5H), 7.42-7.39 (m, 1H), 3.44 (s, 3H).

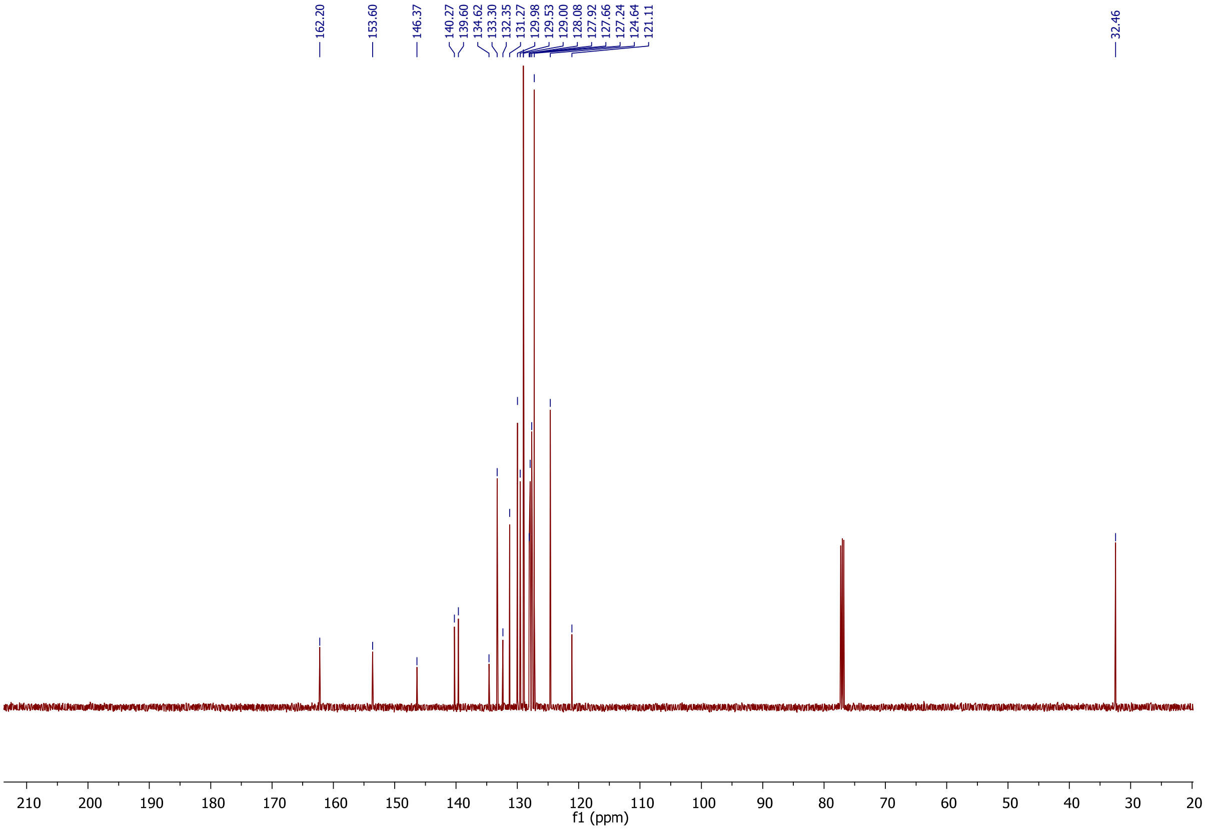

^{13}C NMR (75 Hz, $CDCl_3$) δ ppm: 162.2, 153.6, 146.4, 140.3, 139.6, 134.6, 133.3, 132.4, 131.3, 130.0, 129.5, 129.0, 128.1, 127.9, 127.7, 127.2, 124.6, 121.1, 32.5.

2-(2-Chlorophenyl)-6-(2-methoxyphenyl)-3-methylquinazolin-4(3*H*)-one **A9-4**

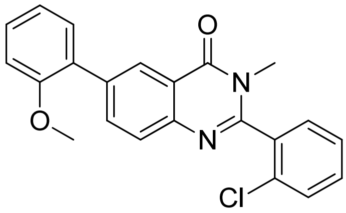

Prepared from 6-bromo-2-(2-chlorophenyl)-3-methylquinazolin-4(3*H*)-one (**4b**) (150 mg, 0.429 mmol) and 2-methoxybenzeneboronic acid (98 mg, 0.645 mmol) according to general procedure 1. Product was purified by silica gel column chromatography eluted with hexane/EtOAc a ratio of 4:1=> 2:1 (v/v), followed by trituration with 2-PrOH/ hexane a 1:3 (v/v) mixture to yield 2-(2-chlorophenyl)-6-(3-methoxyphenyl)-3-methylquinazolin-4(3*H*)-one **A9-4** as an off-white solid (54 mg, 33.54%).

LC-MS: 96.8% (Rt = 3.52), ESI(+) *m/z* found: 376.93 $[M+H]^+$. Molecular Weight calc'd for $C_{22}H_{17}ClN_2O_2$ = 376.10.

^{1}H NMR (300 Hz, $CDCl_3$) δ ppm: δ 8.51 (d, *J* = 1.8 Hz, 1H), 7.99 (dd, *J* = 8.4, 2.1 Hz, 1H), 7.78 (d, *J* = 8.4 Hz, 1H), 7.54-7.52 (m, 1H), 7.51-7.44 (m, 3H), 7.42 (dd, *J* = 7.5, 1.7 Hz, 1H), 7.38 (ddd, *J* = 8.2, 7.5, 1.7 Hz, 1H), 7.08 (td, *J* = 7.5, 1.0 Hz, 1H), 7.03 (d, *J* = 8.3 Hz, 1H), 3.85 (s, 3H), 3.43 (s, 3H).

2-(2-Chlorophenyl)-6-(3-methoxyphenyl)-3-methylquinazolin-4(3*H*)-one **A9-5**

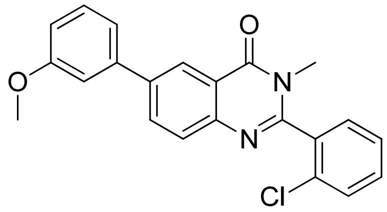

Prepared from 6-bromo-2-(2-chlorophenyl)-3-methylquinazolin-4(3*H*)-one (**4b**) (150 mg, 0.429 mmol) and 3-methoxybenzeneboronic acid (130 mg, 0.645 mmol) according to general procedure 1. Product was purified by silica gel column chromatography eluted with EtOAc/hexane a ratio of 1:4=> 1:2 (v/v), followed by trituration with 2-PrOH/hexane a 1:3 (v/v) mixture to yield 2-(2-chlorophenyl)-6-(3-methoxyphenyl)-3-methylquinazolin-4(3*H*)-one **A9-5** as an off-white solid (90 mg, 55.9%).

LC-MS: 93.2% (Rt = 3.53), ESI(+) *m/z* found: 377.0 $[M+H]^+$. Molecular Weight calc'd for $C_{22}H_{17}ClN_2O_2$ = 376.10.

^{1}**H NMR** (300 Hz, $CDCl_3$) δ ppm: 8.58 (d, *J* = 1.9 Hz, 1H), 8.02 (dd, *J* = 8.5, 2.2 Hz, 1H), 7.81 (d, *J* = 8.5 Hz, 1H), 7.54-7.44 (m, 4H), 7.40 (t, *J* = 7.9 Hz, 1H), 7.35 (ddd, *J* = 7.6, 1.6, 0.9 Hz, 1H), 7.24 (dd, *J* = 2.4, 1.8 Hz, 1H), 6.95 (ddd, *J* = 8.2, 2.6, 0.9 Hz, 1H), 3.89 (s, 3H), 3.43 (s, 3H).

^{13}**C NMR** (75 Hz, $CDCl_3$) δ ppm: 162.4, 160.3, 153.8, 146.7, 141.2, 140.3, 134.8, 133.5, 132.5, 131.4, 130.2, 130.1, 129.7, 128.2, 127.8, 124.8, 121.2, 119.9, 113.6, 112.9, 55.5, 32.6.

2-(2-Chlorophenyl)-6-(4-methoxyphenyl)-3-methylquinazolin-4(3*H*)-one **A9-6**

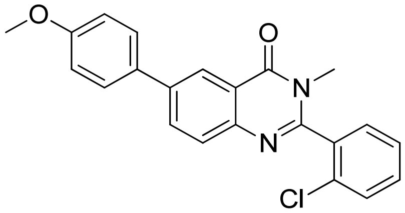

Prepared from 6-bromo-2-(2-chlorophenyl)-3-methylquinazolin-4(3*H*)-one (**4b**) (200 mg, 0.572 mmol) and 4-methoxybenzeneboronic acid (130 mg, 0.855 mmol) according to general procedure 1. Product was purified by silica gel column chromatography eluted with chloroform, followed by trituration with 2-PrOH/hexane a 1:3 (v/v) mixture to yield 2-(2-chlorophenyl)-6-(4-methoxyphenyl)-3-methylquinazolin-4(3*H*)-one **A9-6** as an off-white solid (144 mg, 66.7%).

LC-MS: 94.3% (Rt = 3.77), ESI(+) *m/z* found: 376.83 $[M+H]^+$. Molecular Weight calc'd for $C_{22}H_{17}ClN_2O2$ = 376.10.

^{1}**H NMR** (300 Hz, $CDCl_3$) δ ppm: 8.53 (dd, *J* = 2.2, 0.5 Hz, 1H), 8.00 (dd, *J* = 8.5, 2.2 Hz, 1H), 7.79 (dd, *J* = 8.5, 0.5 Hz, 1H), 7.68-7.65 (m, 2H), 7.55-7.44 (m, 4H), 7.04-7.01 (m, 2H), 3.88 (s, 3H), 3.43 (s, 3H).

^{13}**C NMR** (75 Hz, $CDCl_3$) δ ppm: 162.3, 159.7, 153.3, 146.0, 139.9, 134.7, 132.9, 132.4, 132.1, 131.2, 130.0, 129.6, 128.3, 128.1, 127.7, 123.9, 121.2, 114.5, 55.4, 32.5.

2-(2-Chlorophenyl)-6-(2,3-dimethoxyphenyl)-3-methylquinazolin-4(3*H*)-one **A9-7**

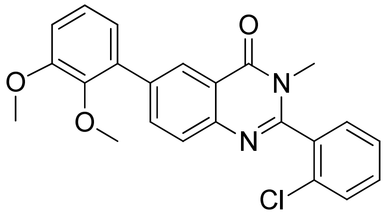

Prepared from 6-bromo-2-(2-chlorophenyl)-3-methylquinazolin-4(3H)-one (**4b**) (700 mg, 2.0 mmol) and 2,3-dimethoxyphenylboronic acid (470 mg, 2.58 mmol) according to general procedure 2. Crude product was purified by silica gel column chromatography eluted with hexane/EtOAc a ratio of 2:1 (v/v), followed by trituration with 2-PrOH/hexane a 1:3 (v/v) to yield 2-(2-chlorophenyl)-6-(2,3-dimethoxyphenyl)-3-methylquinazolin-4(3*H*)-one **A9-7** as an off-white solid (0.60 g, 74.1%).

LC-MS: 100% (Rt = 3.49), ESI(+) *m/z* found: 406.93 $[M+H]^+$. Molecular Weight calc'd for $C_{23}H_{19}ClN_2O_3$ = 406.11.

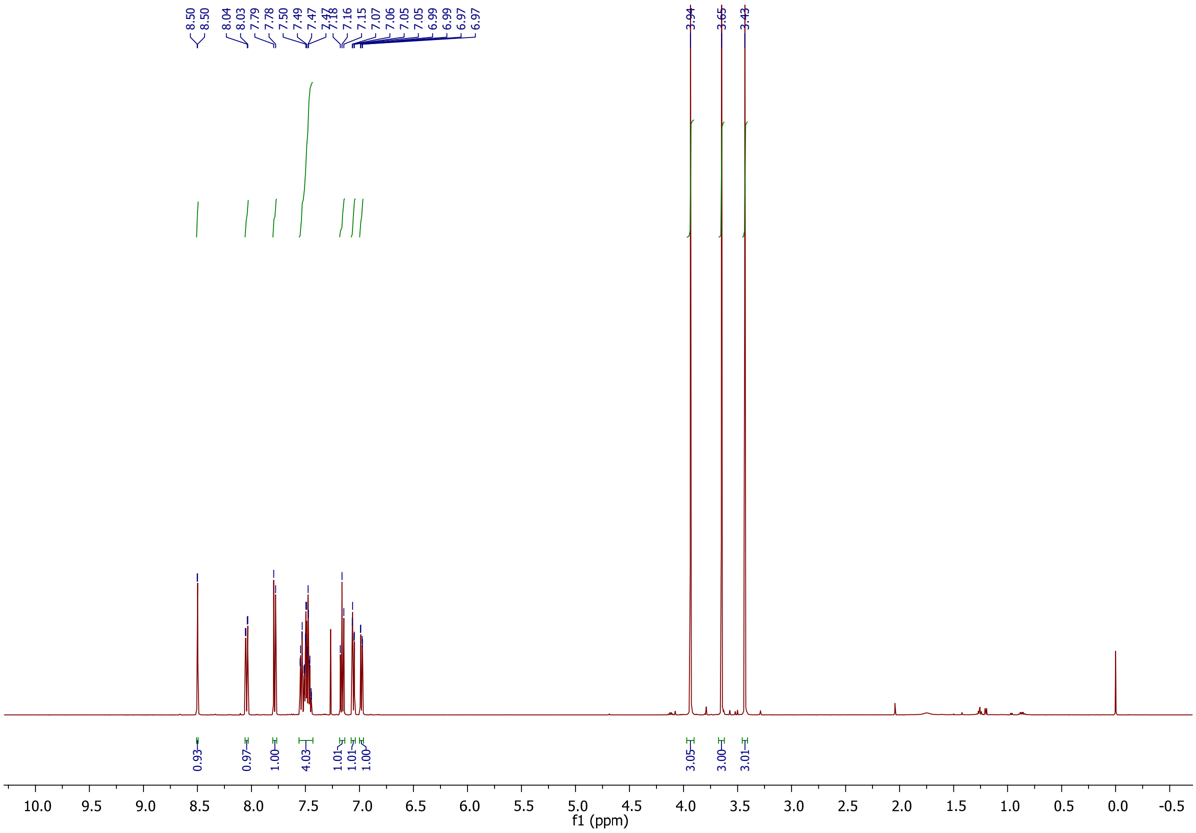

^{1}H NMR (300 Hz, $CDCl_3$) δ ppm: 8.50 (d, *J* = 2.2, 1H), 8.04 (dd, *J* = 8.4, 2.1, 1H), 7.78 (d, *J* = 8.4, 1H), 7.55-7.46 (m, 4H), 7.16 (t, *J* = 8.0, 1H), 7.06 (dd, *J* = 7.8, 1.5, 1H), 6.98 (dd, *J* = 8.2, 1.4, 1H), 3.94 (s, 3H), 3.65 (s, 3H), 3.43 (s, 3H).

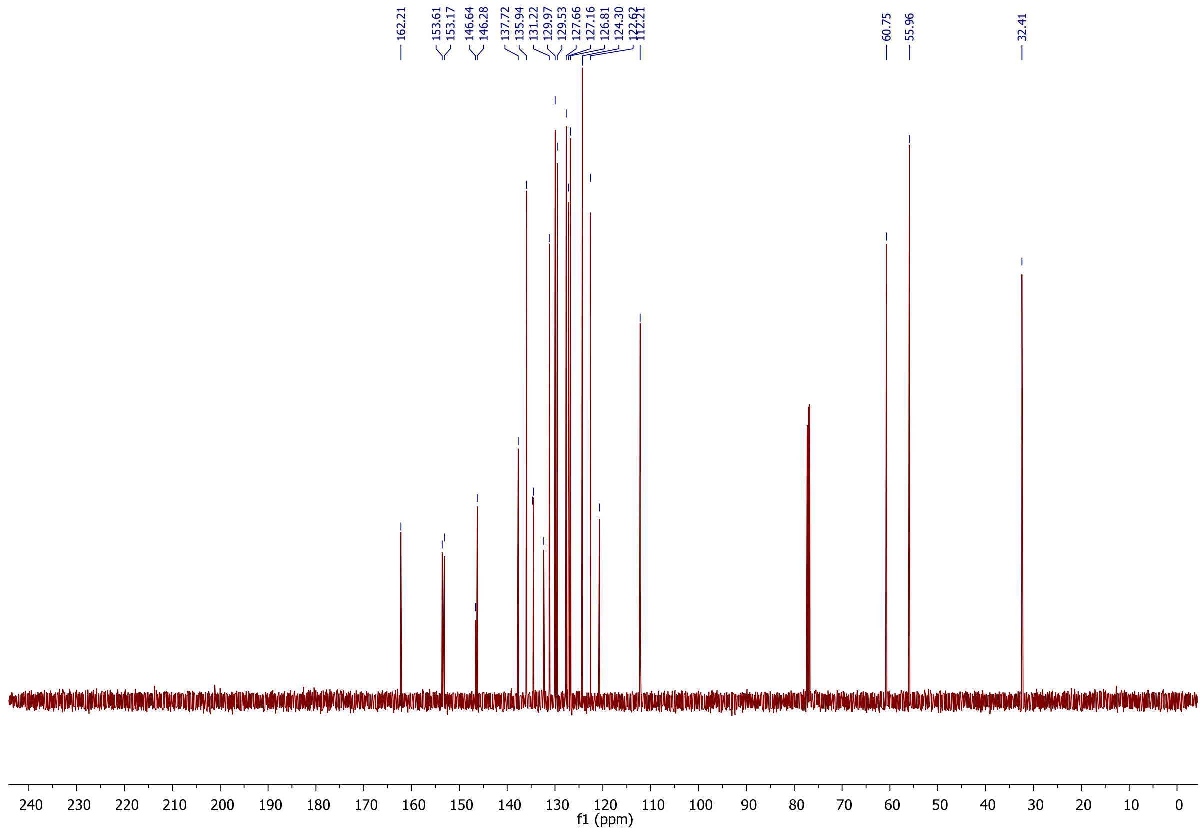

^{13}C NMR (75 Hz, $CDCl_3$) δ ppm: 162.2, 153.6, 153.2, 146.6, 146.3, 137.7, 136.0, 134.7, 134.5, 132.4, 131.2, 130.0, 129.5, 127.7, 127.2, 126.8, 124.3, 122.6, 120.8, 112.2, 60.8, 56.0, 32.4.

2-(2-Chlorophenyl)-6-(2,4-dimethoxyphenyl)-3-methylquinazolin-4(3*H*)-one **A9-8**

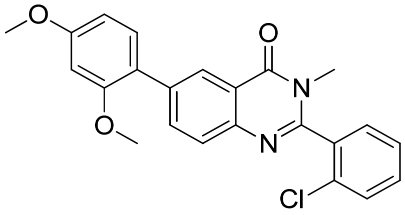

Prepared from 6-bromo-2-(2-chlorophenyl)-3-methylquinazolin-4(3*H*)-one (**4b**) (180 mg, 0.51 mmol) and 2,4-dimethoxyphenylboronic acid (141 mg, 0.77 mmol) according to general procedure 1. Crude product was purified by silica gel column chromatography eluted with hexane/EtOAc a ratio of 3:1=>1:1 (v/v), followed by trituration with 2-PrOH/hexane a 1:3 (v/v) mixture to yield 2-(2-chlorophenyl)-6-(2,4-dimethoxyphenyl)-3-methylquinazolin-4(3*H*)-one **A9-8** as an off-white solid (63 mg, 30.4%).

LC-MS: 98.8% (Rt = 3.49), ESI(+) *m/z* found: 406.68 $[M+H]^+$. Molecular Weight calc'd for $C_{23}H_{19}ClN_2O_3$ = 406.11.

^{1}H NMR (300 Hz, $CDCl_3$) δ ppm: 8.49 (d, *J* = 2.1, 1H), 7.99 (dd, *J* = 8.5, 2.1, 1H), 7.78 (d, *J* = 8.5, 1H), 7.56-7.45 (m, 4H), 7.38 (d, *J* = 8.2, 1H), 6.65-6.61 (m, 2H)), 3.89 (s, 3H), 3.86 (s, 3H), 3.44 (s, 3H).

^{13}C NMR (75 Hz, $CDCl_3$) δ ppm: 162.4, 161.0, 157.7, 153.4, 145.9, 138.0, 136.2, 134.9, 132.5, 131.6, 131.3, 130.1, 129.7, 127.8, 127.1, 126.9, 122.2, 120.8, 105.0, 99.2, 55.7, 55.6, 32.5.

2-(2-Chlorophenyl)-6-(3,4-dimethoxyphenyl)-3-methylquinazolin-4(3*H*)-one **A9-9**

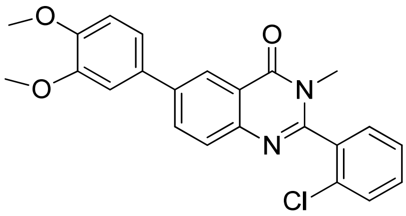

Prepared from 6-bromo-2-(2-chlorophenyl)-3-methylquinazolin-4(3*H*)-one (**4b**) (180 mg, 0.51 mmol) and 3,4-dimethoxyphenylboronic acid (141 mg, 0.77 mmol) according to general procedure 2. Crude product was purified by silica gel column chromatography eluted with hexane/EtOAc a ratio of 2:1 (v/v), followed by trituration with 2-PrOH/hexane a 1:3 (v/v) mixture to yield 2-(2-chlorophenyl)-6-(3,4-dimethoxyphenyl)-3-methylquinazolin-4(3*H*)-one **A9-9** as an off-white solid (0.124 g, 94.9%).

LC-MS: 94.9% (Rt = 3.27), ESI(+) *m/z* found: 407.0 $[M+H]^+$. Molecular Weight calc'd for $C_{23}H_{19}ClN_2O_3$ = 406.11.

^{1}**H NMR** (300 Hz, $CDCl_3$) δ ppm: 8.54 (d, *J* = 2.2, 1H), 8.01 (dd, *J* = 8.5, 2.2, 1H), 7.80 (d, *J* = 8.5, 1H), 7.55-7.45 (m, 4H), 7.28 (dd, *J* = 8.3, 2.1, 1H), 7.23 (d, *J* = 2.0, 1H), 7.00 (d, *J* = 8.3, 1H), 4.00 (s, 3H), 3.95 (s, 3H), 3.44 (s, 3H).

^{13}**C NMR** (75 Hz, $CDCl_3$) δ ppm: 162.3, 153.4, 149.4, 149.2, 146.1, 140.1, 134.7, 133.1, 132.5, 132.4, 131.3, 130.0, 129.6, 128.1, 127.7, 124.0, 121.1, 119.7, 111.6, 110.3, 56.1, 56.0, 32.5.

2-(2-Chlorophenyl)-6-(2,5-dimethoxyphenyl)-3-methylquinazolin-4(3*H*)-one **A9-10**

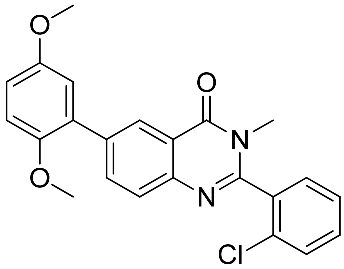

Prepared from 6-bromo-2-(2-chlorophenyl)-3-methylquinazolin-4(3*H*)-one (**4b**) (180 mg, 0.51 mmol) and 2,5-dimethoxyphenylboronic acid (141 mg, 0.77 mmol) according to general procedure 2. Crude product was purified by silica gel column chromatography eluted with hexane/EtOAc a ratio of 2:1 (v/v), followed by trituration with 2-PrOH/hexane a 1:3 (v/v) mixture to yield 2-(2-chlorophenyl)-6-(2,5-dimethoxyphenyl)-3-methylquinazolin-4(3*H*)-one **A9-10** as an off-white solid (0.130 g, 62.2%).

LC-MS: 98.6% (Rt = 3.54), ESI(+) *m/z* found: 407.08 $[M+H]^+$. Molecular Weight calc'd for $C_{23}H_{19}ClN_2O_3$ = 406.11.

^{1}**H NMR** (300 Hz, $CDCl_3$) δ ppm: 8.50 (d, *J* = 2.1, 1H), 8.00 (dd, *J* = 8.4, 2.1, 1H), 7.78 (d, *J* = 8.4, 1H), 7.54-7.52 (m, 1H), 7.51-7.44 (m, 3H), 7.00 (d, *J* = 3.0, 1H), 6.96 (d, *J* = 8.9, 1H), 6.90 (d, *J* = 8.9, 3.0, 1H), 3.83 (s, 3H), 3.79 (s, 3H), 3.43 (s, 3H).

^{13}**C NMR** (75 Hz, $CDCl_3$) δ ppm: 162.2, 153.9, 153.6, 150.8, 146.2, 137.8, 136.1, 134.8, 132.4, 131.2, 130.1, 130.0, 129.5, 127.7, 127.1 (2C), 120.7, 116.6, 114.0, 112.7, 56.3, 55.9, 32.4.

2-(2-Chlorophenyl)-6-(3,5-dimethoxyphenyl)-3-methylquinazolin-4(3*H*)-one **A9-11**

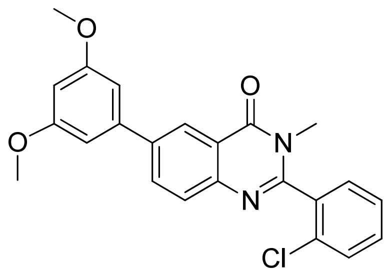

Prepared from 6-bromo-2-(2-chlorophenyl)-3-methylquinazolin-4(3*H*)-one (**4b**) (180 mg, 0.51 mmol) and 3,5-dimethoxyphenylboronic acid (141 mg, 0.77 mmol) according to general procedure 2. Crude product was purified by silica gel column chromatography eluted with hexane/EtOAc a ratio of 3:1=>1:1 (v/v), followed by trituration with 2-PrOH/hexane a 1:3 (v/v) mixture to yield 2-(2-chlorophenyl)-6-(3,5-dimethoxyphenyl)-3-methylquinazolin-4(3*H*)-one **A9-11** as an off-white solid (96 mg, 46.4%).

LC-MS: 96.8% (Rt = 3.55), ESI(+) *m/z* found: 406.68 $[M+H]^+$. Molecular Weight calc'd for $C_{23}H_{19}ClN_2O_3$ = 406.11.

^{1}H NMR (300 Hz, $CDCl_3$) δ ppm: 8.57 (dd, *J* = 2.2, 0.4, 1H), 8.01 (dd, *J* = 8.5, 2.2, 1H), 7.81 (dd, *J* = 8.5, 0.4, 1H), 7.55-7.45 (m, 4H), 6.84 (d, *J* = 2.2, 2H), 6.52 (t, *J* = 2.2, 1H), 3.88 (s, 6H), 3.44 (s, 3H).

^{13}C NMR (75 Hz, $CDCl_3$) δ ppm: 162.2, 161.3, 153.7, 146.6, 141.8, 140.2, 134.7, 133.4, 132.4, 131.3, 130.0, 129.5, 128.1, 127.7, 124.7, 121.0, 105.5, 100.1, 55.5, 32.5.

2-(2-Chlorophenyl)-6-(2,6-dimethoxyphenyl)-3-methylquinazolin-4(3*H*)-one **A9-12**

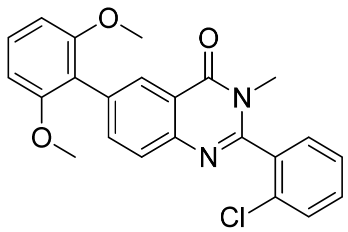

Prepared from 6-bromo-2-(2-chlorophenyl)-3-methylquinazolin-4(3*H*)-one (**4b**) (200 mg, 0.57 mmol) and 2,6-dimethoxyphenylboronic acid (140 mg, 0.77 mmol) according to general procedure 2. Crude product was purified by silica gel column chromatography eluted with hexane/EtOAc a ratio of 2:1 (v/v), followed by trituration with 2-PrOH/hexane a 1:3 (v/v) mixture to yield 2-(2-chlorophenyl)-6-(2,6-dimethoxyphenyl)-3-methylquinazolin-4(3*H*)-one **A9-12** as an off-white solid (0.050 g, 21.7%).

LC-MS: 97.9% (Rt = 3.49), ESI(+) *m/z* found: 407.08 $[M+H]^+$. Molecular Weight calc'd for $C_{23}H_{19}ClN_2O_3$ = 406.11.

^{1}H NMR (300 Hz, $CDCl_3$) δ ppm: 8.37 (t, *J* = 1.3, 1H), 7.78 (d, *J* = 1.3, 2H), 7.54-7.52 (m, 1H), 7.49-7.43 (m, 3H), 7.32 (t, *J* = 8.4, 1H), 8.68 (d, *J* = 8.4, 2H), 3.76 (s, 6H), 3.41 (s, 3H).

^{13}C NMR (75 Hz, $CDCl_3$) δ ppm: 162.3, 157.6 (2C), 153.3, 146.0, 137.7, 134.9, 133.7, 132.4, 131.2, 130.0, 129.6, 129.3, 129.0, 127.6, 126.8, 120.5, 118.0, 104.1 (2C), 55.9 (2C), 32.3.

2-(2-Chlorophenyl)-3-methyl-6-(pyridin-4-yl)quinazolin-4(3*H*)-one **A9-13**

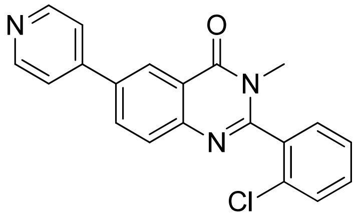

Prepared from 6-bromo-2-(2-chlorophenyl)-3-methylquinazolin-4(3*H*)-one (**4b**) (200 mg, 0.57 mmol) and 4-pyridylboronic acid (110 mg, 0.86 mmol) according to general procedure 1. Crude product was purified by silica gel column chromatography eluted with hexane/EtOAc a ratio of 2:1 (v/v), followed by trituration with 2-PrOH/hexane a 1:3 (v/v) mixture to yield 2-(2-chlorophenyl)-3-methyl-6-(pyridin-4-yl)quinazolin-4(3*H*)-one **A9-12** as an off-white solid (50 mg, 21.7%).

LC-MS: 97.3% (Rt = 1.94), ESI(+) m/z found: 348.07 $[M+H]^+$ Molecular Weight calc'd for $C_{20}H_{14}ClN_3O$ = 347.08.

^{1}H NMR (300 Hz, $CDCl_3$) δ ppm: 8.76 (dd, *J* = 4.8, 1.4, 2H), 8.68 (d, *J* = 2.2, 0.5, 1H), 8.08 (dd, *J* = 8.5, 2.3, 1H), 7.89 (dd, *J* = 8.5, 0.5, 1H), 7.77 (dd, *J* = 4.6, 1.6, 2H), 7.57-7.47 (m, 4H), 3.46 (s, 3H).

^{13}C NMR (75 Hz, $CDCl_3$) δ ppm: 161.9, 154.8, 148.9 (2C), 148.3, 148.0, 136.3, 134.4, 132.7, 132.2, 131.5, 130.1, 129.4, 128.8, 127.8, 125.5, 122.0 (2C), 121.4, 32.6.

2-(2-Chlorophenyl)-3-methyl-6-(pyridin-3-yl)quinazolin-4(3*H*)-one **A9-14**

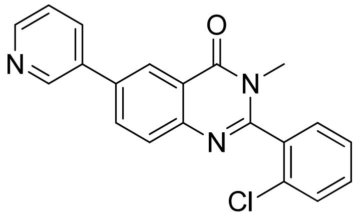

Prepared from 6-bromo-2-(2-chlorophenyl)-3-methylquinazolin-4(3*H*)-one (**4b**) (200 mg, 0.57 mmol) and 3-pyridylboronic acid (110 mg, 0.86 mmol) according to general procedure 1. Crude product was purified by silica gel column chromatography eluted with hexane/EtOAc a ratio of 2:1 (v/v), followed by trituration with 2-PrOH/hexane a 1:3 (v/v) mixture to yield 2-(2-chlorophenyl)-3-methyl-6-(pyridin-3-yl)quinazolin-4(3*H*)-one **A9-14** as an off-white solid (50 mg, 21.7%).

LC-MS: 93.8% (Rt = 2.36), ESI(+) m/z found: 348.10 $[M+H]^+$ Molecular Weight calc'd for $C_{20}H_{14}ClN_3O$ = 347.08.

^{1}H NMR (300 Hz, $CDCl_3$) δ ppm: 8.98 (d, *J* = 1.7, 1H), 8.66 (d, *J* = 4.8, 1.6, 1H), 8.59 (dd, *J* = 2.2, 0.5, 1H), 8.04-8.00 (m, 2H), 7.87 (dd, *J* = 8.4, 0.5, 1H), 7.56-7.46 (m, 4H), 7.43 (ddd, *J* = 7.9, 4.8, 0.8, 1H), 3.45 (s, 3H).

^{13}C NMR (75 Hz, $CDCl_3$) δ ppm: 162.1, 154.1, 149.1, 148.4, 147.0, 136.9, 135.2, 134.6, 134.5, 133.0, 132.3, 131.4, 130.0, 129.5, 128.6, 127.7, 125.0, 123.7, 121.4, 32.6.

2-(2-Chlorophenyl)-6-(furan-3-yl)-3-methylquinazolin-4(3*H*)-one **A9-15**

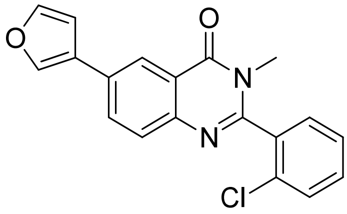

Prepared from 6-bromo-2-(2-chlorophenyl)-3-methylquinazolin-4(3*H*)-one (**4b**) (200 mg, 0.57 mmol), 3-furanboronic acid (96 mg, 0.85 mmol), according to general procedure 1.

Crude product was purified by silica gel column chromatography using hexane/EtOAc (3:1=>2:1) as an eluent followed by triturating with 2-PrOH/hexane (1:3) to yield 2-(2-chlorophenyl)-6-(furan-3-yl)-3-methylquinazolin-4(3*H*)-one **A9-15** as an off-white solid (25 mg, 14.0%).

LC-MS: 94.2% (Rt = 3.27), ESI(+) m/z found: 337.21 $[M+H]^+$. Molecular Weight calc'd for $C_{19}H_{13}ClN_2O_2$ = 336.07.

^{1}H NMR (300 Hz $CDCl_3$) δ ppm: 8.44 (dd, *J* = 2.1, 0.5, 1H), 7.91 (dd, *J* = 8.4, 2.1, 1H), 7.88 (dd, *J* = 1.5, 0.9, 1H), 7.76 (dd, *J* = 8.5, 0.5, 1H), 7.55-7.44 (m, 5H), 6.85 (dd, *J* = 1.9, 0.9, 1H), 3.43 (s, 3H).

^{13}C NMR (75 Hz $CDCl_3$) δ ppm: 162.0, 153.4, 145.7, 144.1, 139.3, 134.3, 132.4, 132.1, 131.9, 131.4, 130.0, 129.6, 127.9, 127.7, 125.5, 123.0, 121.1, 108.8, 32.5.

2-(2-Chlorophenyl)-3-methyl-6-(2-methylfuran-3-yl)quinazolin-4(3*H*)-one **A9-16**

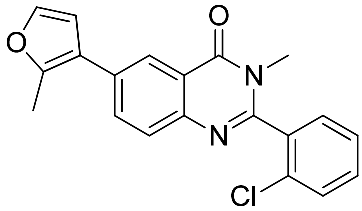

Prepared from 6-bromo-2-(2-chlorophenyl)-3-methylquinazolin-4(3*H*)-one **(4b)** (200 mg, 0.57 mmol) and 2-methylfuran-3-boronic acid pinacol ester (190 mg, 0.953 mmol) according to general procedure 3. Crude product was purified by silica gel column chromatography eluted with hexane/EtOAc a ratio of 1:3=>1:1 (v/v), followed by trituration with 2-PrOH/hexane a 1:3 (v/v) mixture to yield 2-(2-chlorophenyl)-3-methyl-6-(2-methylfuran-3-yl)quinazolin-4(3*H*)-one **A9-16** as a solid (31 mg, 15.5%).

LC-MS: 97.4% (Rt = 3.43), ESI(+) *m/z* found: 351.14 $[M+H]^+$. Molecular Weight calc'd for $C_{20}H_{15}ClN_2O_2$ = 350.80.

^{1}H NMR (300 Hz $CDCl_3$) δ ppm: 8.36 (d, *J* = 2.0, 1H), 7.83 (dd, *J* = 8.4, 2.1, 1H), 7.78 (d, *J* = 8.4, 1H), 7.55-7.46 (m, 4H), 7.38 (d, *J* = 1.9, 1H), 6.64 (d, *J* = 1.9, 1H), 3.43 (s, 3H), 2.54 (s, 3H).

^{13}C NMR (75 Hz $CDCl_3$) δ ppm: 162.2, 153.3, 148.7, 145.6, 140.7, 134.7, 133.7, 133.6, 132.4, 131.2, 130.0, 129.6, 127.9, 127.7, 124.6, 121.1, 120.0, 111.0, 32.4, 13.3.

2-(2-Chlorophenyl)-3-methyl-6-(thiophen-3-yl)quinazolin-4(3*H*)-one **A9-17**

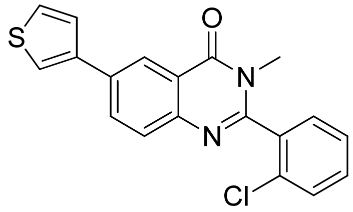

Prepared from 6-bromo-2-(2-chlorophenyl)-3-methylquinazolin-4(3*H*)-one (**4b**) (200 mg, 0.57 mmol) and 3-thienylboronic acid (110 mg, 0.85 mmol) according to general procedure 1. Product was purified by silica gel column chromatography eluted with $CHCl_3$/hexane a ratio of 3:1 (v/v), followed by trituration with 2-PrOH/hexane a 1:3 (v/v) mixture to yield 2-(2-chlorophenyl)-3-methyl-6-(thiophen-3-yl)quinazolin-4(3*H*)-one **A9-17** as a beige solid (84 mg, 41.6%).

LC-MS: 100% (Rt = 3.42), ESI(+) m/z found: 353.14 $[M+H]^+$. Molecular Weight calc'd for $C_{19}H_{13}ClN_2OS$ = 352.84.

^{1}H NMR (300 Hz $CDCl_3$) δ ppm: 8.56 (dd, *J* = 2.2, 0.4, 1H), 8.03 (dd, *J* = 8.5, 2.2, 1H), 7.77 (dd, *J* = 8.5, 0.5, 1H), 7.62 (dd, *J* = 3.0, 1.4, 1H), 7.54-7.44 (m, 6H), 3.43 (s, 3H).

^{13}C NMR (75 Hz $CDCl_3$) δ ppm: 161.7, 153.0, 145.8, 140.4, 134.5, 134.2, 132.2, 131.9, 130.8, 129.5, 129.1, 127.7, 127.2, 126.3, 125.8, 123.2, 121.0, 120.7, 32.0.

2-(2-Chlorophenyl)-6-(3,5-dimethylisoxazol-4-yl)-3-methylquinazolin-4(3*H*)-one **A9-18**

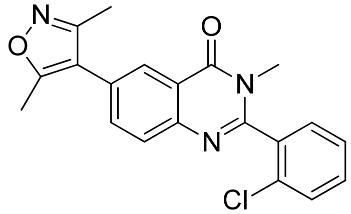

Prepared from 6-bromo-2-(2-chlorophenyl)-3-methylquinazolin-4(3*H*)-one (**4b**) (200 mg, 0.57 mmol) and 3,5-dimethylisoxazole-4-boronic acid (120 mg, 0.86 mmol) according to general procedure 3. Reaction was run run at 90 °C for 3 h. Product was purified by silica gel column chromatography eluted with EtOAc/hexane a ratio of 2:1=>1:1 (v/v), followed by trituration with 2-PrOH/hexane a 1:3 (v/v) mixture to yield 2-(2-chlorophenyl)-6-(3,5-dimethylisoxazol-4-yl)-3-methylquinazolin-4(3*H*)-one **A9-18** as a beige solid (9 mg, 4.3%).

LC-MS: 93.2% (Rt = 3.11), ESI(+) m/z found: 366.07 $[M+H]^+$. Molecular Weight calc'd for $C_{20}H_{16}ClN_3O_2$ = 365.81.

^{1}H NMR (300 Hz $CDCl_3$) δ ppm: 8.25 (dd, *J* = 2.1, 0.5, 1H), 7.83 (dd, *J* = 8.4, 0.5, 1H), 7.67 (dd, *J* = 8.4, 2.1, 1H), 7.56-7.54 (m, 1H), 7.52-7.46 (m, 3H), 3.44 (s, 3H), 2.48 (s, 3H), 2.34 (s, 3H).

2-(2-Chlorophenyl)-3-methyl-6-(1-methyl-1*H*-pyrrol-2-yl)quinazolin-4(3*H*)-one **A9-19**

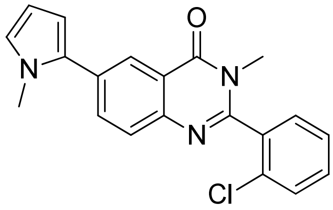

Prepared from 6-bromo-2-(2-chlorophenyl)-3-methylquinazolin-4(3*H*)-one (**4b**) (200 mg, 0.57 mmol) and *N*-methylpyrrole-2-boronic acid pinacol ester (180 mg, 0.86 mmol) according to general procedure 3. Reaction was run at 90 °C for 3 h. Product was purified by silica gel column chromatography eluted with EtOAc/ hexane a ratio of 2:1 (v/v), followed by trituration with 2-PrOH/hexane a 1:3 (v/v) mixture to yield 2-(2-chlorophenyl)-3-methyl-6-(1-methyl-1H-pyrrol-2-yl)quinazolin-4(3*H*)-one **A9-19** as a beige solid (104 mg, 52.0%).

LC-MS: 94.5% (Rt = 3.32), ESI(+) *m/z* found: 350.14 [M+H]+. Molecular Weight calc'd for $C_{20}H_{16}ClN_3O$ = 349.81.

^{1}H NMR (300 Hz CDCl$_3$) δ ppm: 8.36 (d, *J* = 1.8, 1H), 7.85 (dd, *J* = 8.4, 2.1, 1H), 7.77 (dd, *J* = 8.4, 0.4, 1H), 7.54-7.53 (m, 1H), 7.51-7.44 (m, 3H), 6.78 (dd, *J* = 2.5, 2.0, 1H), 6.38 (dd, *J* = 3.6, 1.8, 1H), 6.25 (dd, *J* = 3.6, 2.7, 1H), 3.77 (s, 3H), 3.42 (s, 3H).

^{13}C NMR (75 Hz CDCl$_3$) δ ppm: 162.1, 153.4, 145.7, 134.7, 134.7, 133.2, 132.5, 132.4, 131.3, 130.0, 129.6, 127.8, 127.7, 125.2, 124.8, 120.9, 109.9, 108.2, 35.4, 32.5.

2-(2-Chlorophenyl)-3-methyl-6-(1*H*-pyrazol-4-yl)quinazolin-4(3*H*)-one **A9-20**

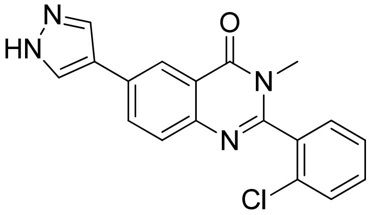

Prepared from 6-bromo-2-(2-chlorophenyl)-3-methylquinazolin-4(3*H*)-one (**4b**) (200 mg, 0.57 mmol) and 4-pyrazoleboronic acid pinacol ester (166 mg, 0.85 mmol) according to general procedure 3. Reaction was run in standard conditions for 6 h. Crude product was purified by silica gel column chromatography eluted with CHCl$_3$/MeOH a ratio of 19:1 (v/v), followed by trituration with 2-PrOH/hexane a 1:3 (v/v) mixture to yield 2-(2-chlorophenyl)-3-methyl-6-(1*H*-pyrazol-4-yl)quinazolin-4(3*H*)-one **A9-19** as a beige solid (120 mg, 62.5%).

LC-MS: 100% (Rt = 2.79), ESI(+) *m/z* found: 337.04 [M+H]$^+$. Molecular Weight calc'd for $C_{18}H_{13}ClN_4O$ = 336.78.

^{1}H NMR (500 MHz, CDCl$_3$) δ ppm: 8.47 (s, 1H), 8.02 (s, 2H), 7.94 (dd, *J* = 8.4, 1.7 Hz, 1H), 7.77 (d, *J* = 8.4 Hz, 1H), 7.56-7.42 (m, 5H), 3.41 (s, 3H).

2-(2-Chlorophenyl)-3-methyl-6-(1-methyl-1*H*-pyrazol-4-yl)quinazolin-4(3*H*)-one **A9-21**

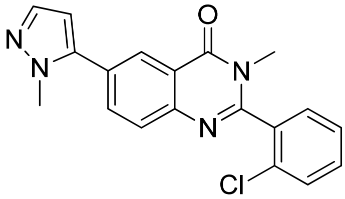

Prepared from 6-bromo-2-(2-chlorophenyl)-3-methylquinazolin-4(3*H*)-one (**4b**) (200 mg, 0.57 mmol) and 1-methyl-1*H*-pyrazole-4-boronic acid (79 mg, 0.63 mmol) according to general procedure 3. Product was purified by silica gel column chromatography eluted with $CHCl_3$/MeOH a ratio of 19:1 (v/v), followed by trituration with 2-PrOH/hexane a 1:3 (v/v) mixture to yield 2-(2-chlorophenyl)-3-methyl-6-(1-methyl-1H-pyrazol-4-yl)quinazolin-4(3*H*)-one **A9-21** as a beige solid (91 mg, 45.7%).

LC-MS: 96.3% (Rt = 2.83), ESI(+) *m/z* found: 351.00 [M+H]$^+$. Molecular Weight calc'd for $C_{19}H_{15}ClN_4O$ = 350.80.

^{1}H NMR (300 Hz $CDCl_3$) δ ppm: 8.42 (s, 1H), 7.86-7.82 (m, 2H), 7.57-7.46 (m, 5H), 6.44 (d, J = 1.8, 1H), 3.99 (s, 3H), 3.44 (s, 3H).

^{13}C NMR (75 Hz $CDCl_3$) δ ppm: 161.8, 154.4, 146.9, 142.2, 138.7, 134.5, 134.4, 132.2, 131.4, 130.0, 129.7, 129.4, 128.2, 127.7, 126.5, 121.0, 106.7, 37.8, 32.5.

2-(2-Chlorophenyl)-6-(3,5-dimethyl-1*H*-pyrazol-4-yl)-3-methylquinazolin-4(3*H*)-one **A9-22**

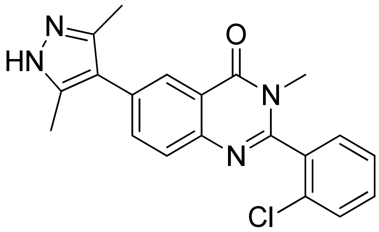

Prepared from 6-bromo-2-(2-chlorophenyl)-3-methylquinazolin-4(3*H*)-one (**4b**) (200 mg, 0.57 mmol) and 3,5-Dimethylpyrazole-4-boronic acid pinacol ester (160 mg, 0.86 mmol) according to general procedure 3. Reaction was run in standard conditions for 6 h. Crude product was purified by silica gel column chromatography eluted with $CHCl_3$/MeOH a ratio of 19:1 (v/v), followed by trituration with 2-PrOH/hexane a 1:3 (v/v) mixture to yield 2-(2-chlorophenyl)-6-(3,5-dimethyl-1H-pyrazol-4-yl)-3-methylquinazolin-4(3*H*)-one **A9-22** as a beige solid (54 mg, 25.8%).

LC-MS: 100% (Rt = 2.62), ESI(+) *m/z* found: 365.00 [M+H]$^+$. Molecular Weight calc'd for $C_{20}H_{17}ClN_4O$ = 364.83.

^{1}H NMR (300 Hz $CDCl_3$) δ ppm: 8.27 (brs, 1H), 7.83-7.80 (m, 2H), 7.56 -7.46 (m, 5H), 3.44 (s, 3H), 2.39 (s, 6H).

^{13}C NMR (75 Hz $CDCl_3$) δ ppm: 162.2, 153.4, 145.5, 142.0 (2C), 135.6, 134.7, 133.5, 132.4, 131.3, 130.0, 129.6, 127.7, 127.7, 126.5, 121.0, 117.4, 32.4, 25.4, 11.7.

2-(2-Chlorophenyl)-6-(1,3-dimethyl-1*H*-pyrazol-5-yl)-3-methylquinazolin-4(3*H*)-one **A9-23**

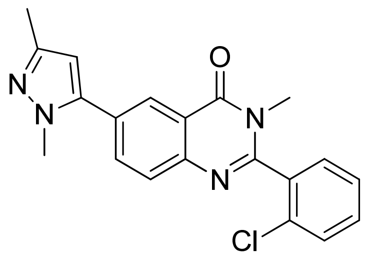

Prepared from 6-bromo-2-(2-chlorophenyl)-3-methylquinazolin-4(3*H*)-one (**4b**) (200 mg, 0.57 mmol) and 1,3-Dimethylpyrazole-5-boronic acid pinacol ester (150 mg, 0.67 mmol) according to general procedure 3. Reaction was run in standard conditions for 3 h. Crude product was purified by silica gel column chromatography eluted with hexane/EtOAc a ratio of 2:1 (v/v), followed by trituration with 2-PrOH/hexane a 1:3 (v/v) mixture to yield 2-(2-chlorophenyl)-6-(1,3-dimethyl-1H-pyrazol-5-yl)-3-methylquinazolin-4(3*H*)-one **A9-23** as a beige solid (68 mg, 32.7%).

LC-MS: 98.2% (Rt = 2.94), ESI(+) *m/z* found: 365.00 $[M+H]^+$. Molecular Weight calc'd for $C_{20}H_{17}ClN_4O$ = 364.83.

^{1}H NMR (300 Hz CDCl$_3$) δ ppm: 8.40 (t, *J* = 1.2, 1H), 7.82 (d, *J* = 1.2, 2H), 7.53-7.46 (m, 1H), 7.53-7.46 (m, 3H), 6.22 (s, 1H), 3.91 (s, 3H), 3.44 (s, 3H), 2.33 (s, 3H).

^{13}C NMR (75 Hz CDCl$_3$) δ ppm: 161.9, 154.3, 147.8, 146.9, 143.0, 134.5, 134.5, 132.3, 131.4, 130.0, 129.9, 129.4, 128.1, 127.7, 126.4, 121.0, 106.2, 37.4, 32.5, 13.5.

2-(2-Chlorophenyl)-3-methyl-6-(piperidin-1-yl)quinazolin-4(3*H*)-one **A9-24**

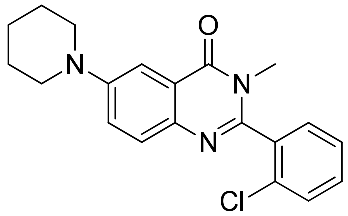

A mixture of 6-bromo-3-methyl-2-phenylquinazolin-4(3*H*)-one (**4b**) (200 mg, 0.57 mmol), piperidine (81 mg, 0.95 mmol), cesium carbonate (520 mg, 1.6 mmol) in 5 mL of 1,4-dioxane was degassed with argon and next $Pd_2(dba)_3$(6 mg, 0.01 mmol) and DavePhos (5 mg, 0.01 mmol) were added. Reaction was carried out according to general procedure 4, the mixture was stirred in a sealed tube at 80°C for 8 h, cooled down to RT and diluted with EtOAc (60 mL) and water (20 mL). The layers were separated, the aqueous layer was extracted with EtOAc (3x). Combined organic layers were washed with brine, dried over $MgSO_4$, evaporated and crude product was purified by silica gel column chromatography eluted with hexane/EtOAc, a ratio of 3:1=> 2:1=> 1:1=> 1:2), followed by trituration with 2-PrOH/hexane a 1:3 (v/v) mixture to yield 2-(2-chlorophenyl)-3-methyl-6-(piperidin-1-yl)quinazolin-4(3*H*)-one **A9-24** as a solid (85 mg, 42.1%).

LC-MS: 92.1% (Rt = 3.29), ESI(+) *m/z* found: 354.00 $[M+H]^+$. Molecular Weight calc'd for $C_{20}H_{20}ClN_3O$ = 353.85.

^{1}H NMR (500 MHz, CDCl$_3$) δ ppm: 7.68 (d, *J* = 2.9 Hz, 1H), 7.63 (d, *J* = 9.0 Hz, 1H), 7.52-7.40 (m, 5H), 3.39 (s, 3H), 3.35-3.30 (m, 4H), 1.74 (dt, *J* = 11.2, 5.7 Hz, 4H), 1.66-1.60 (m, 2H).

^{13}C NMR (126 MHz, $CDCl_3$) δ ppm: 162.23, 151.05, 150.44, 140.05, 134.94, 132.65, 130.97, 129.86, 129.80, 128.36, 127.54, 124.39, 121.58, 109.45, 76.77, 50.15, 32.35, 25.52, 24.23.

6-(Benzo[*d*][1,3]dioxol-5-yl)-2-(2-chlorophenyl)-3-methylquinazolin-4(3*H*)-one **A9-25**

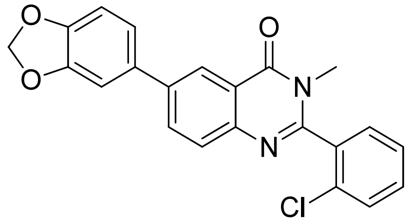

Prepared from 6-bromo-2-(2-chlorophenyl)-3-methylquinazolin-4(3*H*)-one (**4b**) (200 mg, 0.57 mmol) and 3,4-methylenedioxyphenylboronic acid (137 mg, 0.83 mmol) according to general procedure 1. Product was purified by silica gel column chromatography eluted with EtOAc/hexane, a ratio of 1:2 (v/v), by trituration with 2-PrOH/hexane a 1:3 (v/v) mixture to yield 6-(benzo[d][1,3]dioxol-5-yl)-2-(2-chlorophenyl)-3-methylquinazolin-4(3*H*)-one **A9-25** as a beige solid (167 mg, 75.0%).

LC-MS: 96.0% (Rt = 3.54), ESI(+) m/z found: 391.09 $[M+H]^+$. Molecular Weight calc'd for $C_{22}H_{15}ClN_2O_3$ = 390.08.

^{1}H NMR (500 MHz, $CDCl_3$) δ ppm: 8.51 (d, *J* = 2.0 Hz, 1H), 7.96 (dd, *J* = 8.5, 2.2 Hz, 1H), 7.80 (d, *J* = 8.5 Hz, 1H), 7.51 (tdt, *J* = 13.9, 8.8, 4.5 Hz, 4H), 7.24-7.17 (m, 2H), 6.97-6.91 (m, 1H), 6.05 (s, 2H), 3.45 (s, 3H).

^{13}C NMR (126 MHz, $CDCl_3$) ppm: 162.18, 153.41, 148.39, 147.63, 146.13, 139.97, 134.66, 133.92, 133.03, 132.36, 131.23, 129.97, 129.53, 128.05, 127.65, 124.13, 121.08, 120.97, 108.76, 107.63, 101.32, 32.44.

2-(2-Chlorophenyl)-6-(1*H*-indol-6-yl)-3-methylquinazolin-4(3*H*)-one **A9-26**

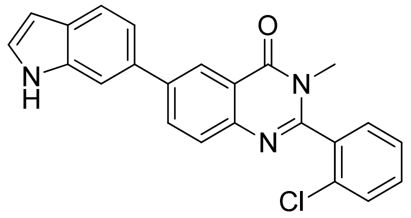

Prepared from 6-bromo-2-(2-chlorophenyl)-3-methylquinazolin-4(3*H*)-one (**4b**) (Scheme I, 200 mg, 0.57 mmol) and indole-6-boronic acid (120 mg, 0.74 mmol) according to general procedure 2. Reaction was run in standard conditions for 1 h. Product was purified by silica gel column chromatography eluted with EtOAc/hexane a ratio of 2:1 (v/v), followed by trituration with EtOAc/hexane a 1:3 (v/v) mixture to yield 2-(2-chlorophenyl)-6-(1*H*-indol-6-yl)-3-methylquinazolin-4(3*H*)-one **A9-26** as a white solid (178 mg, 80.9%).

LC-MS: 95.5% (Rt = 3.36), ESI(+) m/z found: 385.69 $[M+H]^+$. Molecular Weight calc'd for $C_{23}H_{16}ClN_3O$ = 385.85.

^{1}H NMR (400 Hz DMSO-d_6) δ ppm: 11.3 (s, 1H), 8.45 (d, *J* = 2.2, 1H), 8.22 (dd, *J* = 8.5, 2.2, 1H), 7.80 (s, 1H), 7.78 (d, *J* = 8.7, 1H), 7.72-7.68 (m, 3H), 7.65-7.56 (m, 2H), 7.46-7.43 (m, 2H), 6.50 (t, *J* = 2.0, 1H), 3.29 (s, 3H).

^{13}**C NMR** (101 Hz DMSO-d$_6$) δ ppm: 161.8, 153.5, 146.1, 141.1, 137.1, 134.8, 133.6, 132.2, 132.0, 131.5, 130.6, 130.0, 128.4, 128.3, 128.1, 127.2, 123.4, 121.2 (2C), 118.7, 110.2, 101.6, 32.6.

2-(2-Chlorophenyl)-3-methyl-6-(naphthalen-2-yl)quinazolin-4(3*H*)-one **A9-27**

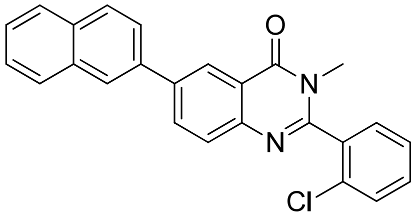

Prepared from 6-bromo-2-(2-chlorophenyl)-3-methylquinazolin-4(3*H*)-one (**4b**) (200 mg, 0.57 mmol), naphthalene-2-boronic acid (130 mg, 0.74 mmol), according to general procedure 2. Product was purified by silica gel column chromatography using EtOAc/hexane a ratio of 3:1=>2:1 (v/v) as an eluent followed by triturating with 2% 2-PrOH in Et$_2$O (1:3) to yield 2-(2-chlorophenyl)-3-methyl-6-(naphthalen-2-yl)quinazolin-4(3*H*)-one **A9-27** as an off-white solid (120 mg, 52.9%).

LC-MS: 94.3% (Rt = 4.14), ESI(+) m/z found: 396.68 [M+H]$^+$. Molecular Weight calc'd for $C_{25}H_{17}ClN_2O$ = 396.87.

^{1}**H NMR** (400 Hz CDCl$_3$) δ ppm: 8.75 (d, *J* = 2.1, 1H), 8.22 (s, 1H), 8.20 (dd, *J* = 8.5, 2.2, 1H), 8.01-7.88 (m, 5H), 7.59-7.47 (m, 6H), 3.48 (s, 3H).

^{13}**C NMR** (101 Hz CDCl$_3$) δ ppm: 162.3, 153.7, 146.5, 140.2, 136.9, 134.7, 133.7, 133.5, 132.9, 132.4, 131.3, 130.0, 129.6, 128.8, 128.4, 128.3, 127.7 (2C), 126.6, 126.4, 126.3, 125.2, 124.9, 121.3, 32.5.

6-(Benzo[*d*]oxazol-6-yl)-2-(2-chlorophenyl)-3-methylquinazolin-4(3*H*)-one **A9-28**

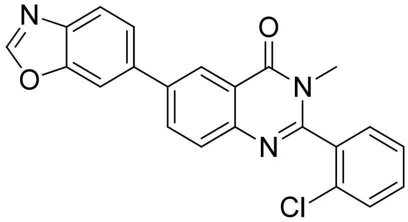

6-(benzo[*d*]oxazol-6-yl)-2-(2-chlorophenyl)-3-methylquinazolin-4(3*H*)-one **A9-28** was prepared according to general procedure 2. A mixture of 6-bromo-2-(2-chlorophenyl)-3-methylquinazolin-4(3*H*)-one (**4b**) (150 mg, 0.43 mmol), benzooxazole-5-boronic acid pinacol ester (137 mg, 0.56 mmol), K_2CO_3 (180 mg, 1.30 mmol) and 1 mL of 2N Na_2CO_3 aq solution in 1.5 mL of dry toluene and 3 mL of dry 1,4-dioxane was degassed with argon and Pd(dppf)Cl_2 complex in DCM (35 mg, 0.043 mmol) was added. Reaction was run in a sealed tube at 80 °C for 1 h. Product was purified by silica gel column chromatography eluted with hexane/EtOAC a ratio of 3:1=>2:1 (v/v) followed by trituration with 2% 2-PrOH in Et$_2$O to yield 6-(benzo[*d*]oxazol-6-yl)-2-(2-chlorophenyl)-3-methylquinazolin-4(3*H*)-one **A9-28** as an off-white solid (120 mg, 75.9%).

LC-MS: 96.3% (Rt = 3.37), ESI(+) *m/z* found: 387.69 [M+H]$^+$. Molecular Weight calc'd for $C_{22}H_{14}ClN_3O_2$ = 387.82.

^{1}H NMR (400 Hz $CDCl_3$) δ ppm: 8.62 (d, *J* = 2.0, 1H), 8.18 (s, 1H), 8.12 (d, *J* = 1.3, 1H), 8.08 (dd, *J* = 8.5, 2.2, 1H), 7.87 (d, *J* = 8.5, 1H), 7.78 (dd, *J* = 8.5, 1.7, 1H), 7.72 (d, *J* = 8.5, 1H), 7.58-7.48 (m, 4H), 3.47 (s, 3H).

^{13}C NMR (101 Hz $CDCl_3$) δ ppm: 162.2, 153.8, 153.3, 149.9, 146.5, 140.9, 140.0, 137.0, 134.7, 133.6, 132.4, 131.3, 130.0, 129.5, 128.3, 127.7, 125.4, 125.1, 121.2, 119.3, 111.4, 32.5.

2-(2-Chlorophenyl)-6-(indolin-5-yl)-3-methylquinazolin-4(3*H*)-one **A9-29**

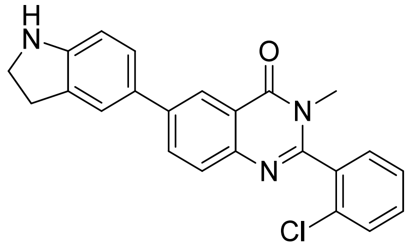

Prepared from 6-bromo-2-(2-chlorophenyl)-3-methylquinazolin-4(3*H*)-one (**4b**) (200 mg, 0.57 mmol) and indolin-6-yl boronic acid (121 mg, 0.74 mmol) according to general procedure 2. Reaction was run in standard conditions for 1 h. Product was purified by silica gel column chromatography eluted with EtOAc/hexane a ratio of 2:1 (v/v), followed by trituration with 2-PrOH/hexane a 1:3 (v/v) mixture to yield 2-(2-chlorophenyl)-6-(indolin-5-yl)-3-methylquinazolin-4(3*H*)-one **A9-29** as a beige solid (117 mg, 53.0%).

LC-MS: 99.4% (Rt = 2.60), ESI(+) m/z found: 387.75 $[M+H]^+$. Molecular Weight calc'd for $C_{23}H_{18}ClN_3O$ = 387.86.

^{1}H NMR (400 Hz CDCl3) δ ppm: 8.56 (d, *J* = 2.1, 1H), 8.01 (dd, *J* = 8.5, 2.2, 1H), 7.80 (d, *J* = 8.5, 1H), 7.56-7.45 (m, 4H), 7.24 (d, *J* = 7.6, 1H), 7.08 (dd, *J* = 7.6, 1.6, 1H), 7.00 (d, *J* = 1.4, 1H), 3.65 (t, *J* = 8.4, 2H), 3.45 (s, 3H), 3.11 (t, *J* = 8.4, 2H).

^{13}C NMR (101 Hz CDCl3) δ ppm: 162.3, 153.3, 152.5, 146.2, 141.0, 139.1, 134.8, 133.4, 132.4, 131.2, 130.0, 129.6, 129.5, 127.9, 127.7, 125.0, 124.4, 121.0, 118.0, 108.0, 47.6, 32.5, 29.6.

2-(2-Chlorophenyl)-6-(3,4-dihydro-2*H*-benzo[*b*][1,4]oxazin-6-yl)-3-methylquinazolin-4(3*H*)-one **A9-30**

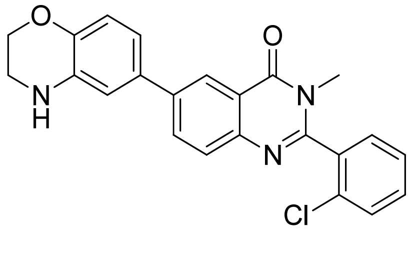

Prepared from 6-bromo-2-(2-chlorophenyl)-3-methylquinazolin-4(3*H*)-one (**4b**) (150 mg, 0.43 mmol) and 3,4-dihydro-2*H*-1,4-benoxazine-6-boronic acid (121 mg, 0.56 mmol) according to general procedure 2. Reaction was run in standard conditions for 2.5 h. Product was purified by silica gel column chromatography eluted with EtOAc/hexane a ratio of 2:1 (v/v), followed by trituration with 2-PrOH/hexane a 1:3 (v/v) mixture to yield 2-(2-chlorophenyl)-6-(3,4-dihydro-2*H*-benzo[*b*][1,4]oxazin-6-yl)-3-methylquinazolin-4(3*H*)-one **A9-30** as a beige solid (160 mg, 92.3%).

LC-MS: 99.0% (Rt = 3.28), ESI(+) *m/z* found: 403.68 $[M+H]^+$. Molecular Weight calc'd for $C_{23}H_{18}ClN_3O_2$ = 403.86.

^{1}H NMR (300 Hz $CDCl_3$) δ ppm: 8.51 (d, *J* = 2.1, 1H), 7.98 (dd, *J* = 8.5, 2.2, 1H), 7.78 (d, *J* = 8.5, 1H), 7.56-7.45 (m, 4H), 7.04 (dd, *J* = 8.3, 2.2, 1H), 6.99 (d, *J* = 2.1, 1H), 6.91 (d, *J* = 8.3, 1H), 4.34-4.32 (m, 2H), 3.51-3.48 (m, 2H), 3.44 (s, 3H).

^{13}C NMR (75 Hz $CDCl_3$) δ ppm: 162.3, 153.2, 146.0, 144.4, 140.3, 134.8, 134.1, 133.0, 132.9, 132.4, 131.2, 130.0, 129.6, 127.9, 127.7, 123.8, 121.1, 117.8, 117.2, 114.2, 65.4, 41.0, 32.4.

6-(Benzo[*d*]thiazol-5-yl)-2-(2-chlorophenyl)-3-methylquinazolin-4(3*H*)-one **A9-31**

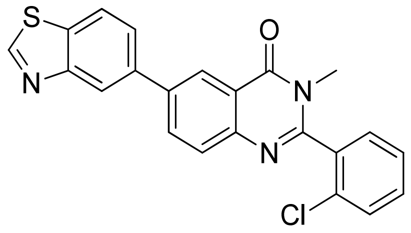

Prepared from 6-bromo-2-(2-chlorophenyl)-3-methylquinazolin-4(3*H*)-one (**4b**) (200 mg, 0.57 mmol) and benzothiazole-5-boronic acid pinacol ester (194 mg, 0.74 mmol) according to according to general procedure 2. Reaction was run in standard conditions for 1 h. Product was purified by silica gel column chromatography eluted with EtOAc/hexane a ratio of 2:1 (v/v), followed by trituration with 2-PrOH/hexane a 1:3 (v/v) mixture to yield 6-(benzo[*d*]thiazol-5-yl)-2-(2-chlorophenyl)-3-methylquinazolin-4(3*H*)-one **A9-31** as a beige solid (44 mg, 19.0%).

LC-MS: 100% (Rt =3.20), ESI(+) *m*/*z* found: 403.68 $[M+H]^+$. Molecular Weight calc'd for $C_{22}H_{14}ClN_3OS$ = 403.88.

^{1}H NMR (400 Hz $CDCl_3$) δ ppm: 9.08 (s, 1H), 8.69 (d, *J* = 2.1, 1H), 8.48 (d, *J* = 1.3, 1H), 8.14 (dd, *J* = 8.5, 2.2, 1H), 8.09 (d, *J* = 8.4, 1H), 7.88 (d, *J* = 8.4, 1H), 7.84 (d, *J* = 8.4, 1.6, 1H), 7.57-7.46 (m, 4H), 3.47 (s, 3H).

^{13}C NMR (101 Hz $CDCl_3$) δ ppm: 162.2, 154.9, 154.1, 153.8, 146.7, 139.7, 138.3, 134.7, 133.5, 133.4, 132.4, 131.3, 130.0, 129.5, 128.4, 127.7, 125.1 (2C), 122.4, 122.1, 121.3, 32.5.

2-(2-Chlorophenyl)-6-(isoquinolin-6-yl)-3-methylquinazolin-4(3*H*)-one **A9-32**

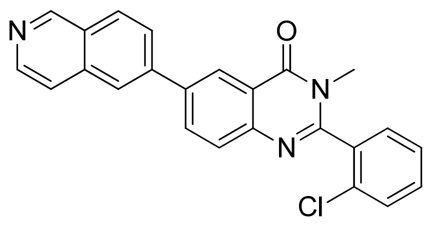

Prepared from 6-bromo-2-(2-chlorophenyl)-3-methylquinazolin-4(3*H*)-one (**4b**) (150 mg, 0.43 mmol) and isoquinolin-6-yl-boronic acid (96 mg, 0.56 mmol) according to according to general procedure 2. Product was purified by silica gel column chromatography eluted with EtOAc/hexane a ratio of 1:2 (v/v), followed by trituration with EtOAc/hexane a 1:2 (v/v) mixture to yield 2-(2-chlorophenyl)-6-(isoquinolin-6-yl)-3-methylquinazolin-4(3*H*)-one **A9-32** as a white solid (78 mg, 45.6%).

LC-MS: 99.0% (Rt = 2.43), ESI(+) *m*/*z* found: 397.68 $[M+H]^+$. Molecular Weight calc'd for $C_{24}H_{16}ClN_3O$ = 397.86.

^{1}H NMR (400 MHz, $CDCl_3$) δ ppm: 9.34 (s, 1H), 8.74 (d, *J* = 2.1 Hz, 1H), 8.61 (d, *J* = 5.6 Hz, 1H), 8.22-8.10 (m, 3H), 8.01 (dd, *J* = 8.5, 1.7 Hz, 1H), 7.91 (d, *J* = 8.5 Hz, 1H), 7.77 (d, *J* = 5.7 Hz, 1H), 7.61-7.46 (m, 4H), 3.48 (s, 3H).

^{13}C NMR (101 MHz, $CDCl_3$) δ ppm: 162.15, 154.14, 152.35, 147.05, 143.71, 141.40, 139.13, 136.12, 134.58, 133.45, 132.33, 131.37, 130.04, 129.48, 128.53, 128.49, 127.94, 127.71, 126.80, 125.46, 124.65, 121.31, 120.75, 32.56.

2-Cyclohexyl-3-methyl-6-phenylquinazolin-4(3*H*)-one **A9-33**

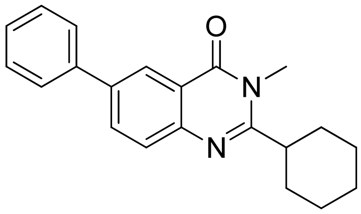

Prepared from 6-bromo-2-cyclohexyl-3-methylquinazolin-4(3*H*)-one (**4c**) (200 mg, 0.62 mmol) and phenylboronic acid (111 mg, 0.90 mmol) according to general procedure 1. Product was purified by silica gel column chromatography eluted with EtOAc/hexane a ratio of 1:2 (v/v), followed by trituration with EtOAc/hexane a 1:2 (v/v) mixture to yield 2-cyclohexyl-3-methyl-6-phenylquinazolin-4(3*H*)-one **A9-33,** as a white solid (106 mg, 53.8%).

LC-MS: 92.9% (Rt = 4.11), ESI(+) *m/z* found: 319.18 $[M+H]^+$. Molecular Weight calc'd for $C_{21}H_{22}N_2O$ = 318.41.

^{1}H NMR (500 MHz, $CDCl_3$) δ ppm: 8.49 (dd, *J* = 2.2, 0.5 Hz, 1H), 7.96 (dd, *J* = 8.5, 2.2 Hz, 1H), 7.72-7.67 (m, 3H), 7.49-7.44 (m, 2H), 7.40-7.34 (m, 1H), 3.69 (s, 3H), 2.83 (tt, *J* = 11.6, 3.1 Hz, 1H), 2.00-1.89 (m, 4H), 1.84-1.72 (m, 3H), 1.48-1.32 (m, 3H).

^{13}C NMR (126 MHz, $CDCl_3$) δ ppm: 162.91, 160.21, 146.67, 139.87, 138.99, 132.85, 128.91, 127.63, 127.62, 127.15, 124.54, 120.42, 76.76, 42.41, 31.06, 30.12, 26.18, 25.80.

2-Cyclohexyl-6-(2,3-dimethoxyphenyl)-3-methylquinazolin-4(3*H*)-one **A9-34**

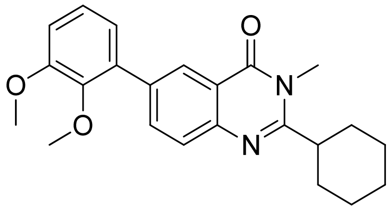

Prepared from 6-bromo-2-cyclohexyl-3-methylquinazolin-4(3*H*)-one (**4c**) (150 mg, 0.47 mmol) and 2,3-dimethoxyphenylboronic acid (90 mg, 0.51 mmol) according to general procedure 2. Product was purified by silica gel column chromatography eluted with EtOAc/hexane, a ratio of 1:2 (v/v), followed by slurring with 2-PrOH/hexane (1:3) to 2-cyclohexyl-6-(2,3-dimethoxyphenyl)-3-methylquinazolin-4(3*H*)-one **A9-34,** as a white solid (122 mg, 68.9%).

LC-MS: 100% (Rt = 3.88), ESI(+) *m/z* found: 379.37 $[M+H]^+$. Molecular Weight calc'd for $C_{23}H_{26}N_2O_3$ = 378.19.

^{1}H NMR (DMSO-d6) δ ppm: 8.17 (d, *J* = 2.1, 1H), 7.87 (dd, *J* = 8.4, 2.2, 1H), 7.62 (d, *J* = 8.5, 1H), 7.16 (t, *J* = 8.0, 1H), 7.10 (dd, *J* = 8.3, 1.5, 1H), 6.98 (dd, *J* = 7.6, 1.6, 1H), 3.86 (s, 3H), 3.60 (s, 3H), 3.52 (s, 3H), 2.99-2.93 (m, 1H), 1.93 (d, *J* = 12.3, 2H), 1.81-1.79 (m, 2H), 1.71 (d, *J* = 12.5, 1H), 1.60 (qd, *J* = 13.0, 3.0, 2H), 1.41 (qt, *J* = 13.0, 3.0, 2H), 1.26 (qt, *J* = 12.8, 3.4, 1H).

^{13}C NMR (DMSO-d6) δ ppm: 161.6, 161.0, 152.9, 146.0, 146.0, 135.6, 135.0, 133.9, 126.6, 126.0, 124.4, 121.9, 119.5, 112.7, 60.2, 55.8, 41.3, 30.7 (2C), 29.8, 25.6, 25.5 (2C).

2-Cyclopropyl-3-methyl-6-phenylquinazolin-4(3*H*)-one **A9-35**

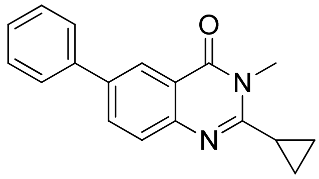

Prepared from 6-bromo-2-cyclopropyl-3-methylquinazolin-4(3*H*)-one (**4d**) (200 mg, 0.72 mmol) and phenylboronic acid (96 mg, 0.79 mmol) according to general procedure 1. Product was purified via silica gel column chromatography eluted with EtOAc/hexane a ratio of 1:2 (v/v), followed by trituration with EtOAc/hexane a 1:2 (v/v) mixture to yield 2-cyclopropyl-3-methyl-6-phenylquinazolin-4(3*H*)-one **A9-35** as a white solid (107 mg, 53.8%).

LC-MS: 95.7% (Rt = 3.29), ESI(+) *m/z* found: 277.22 $[M+H]^+$. Molecular Weight calc'd for $C_{18}H_{16}N_2O$ = 276.33.

^{1}H NMR (500 MHz, $CDCl_3$) δ ppm: 8.48 (dd, *J* = 2.2, 0.5 Hz, 1H), 7.94 (dd, *J* = 8.5, 2.2 Hz, 1H), 7.71-7.66 (m, 2H), 7.63 (dd, *J* = 8.5, 0.5 Hz, 1H), 7.49-7.43 (m, 2H), 7.40-7.34 (m, 1H), 3.82 (s, 3H), 2.05 (tt, *J* = 8.1, 5.0 Hz, 1H), 1.31-1.27 (m, 2H), 1.14-1.09 (m, 2H).

^{13}C NMR (126 MHz, $CDCl_3$) δ ppm: 162.63, 157.51, 146.70 , 139.83, 138.82, 132.87, 128.91, 127.63, 127.38, 127.12, 124.60, 120.42, 76.76, 30.36, 14.64, 8.36.

3-Methyl-2-neopentyl-6-phenylquinazolin-4(3*H*)-one **A9-36**

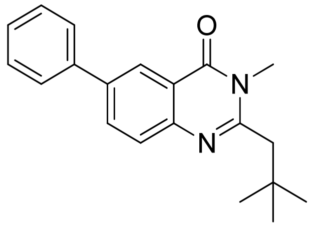

Prepared from 6-bromo-3-methyl-2-neopentylquinazolin-4(3*H*)-one (**4e**) (200 mg, 0.65 mmol) and phenylboronic acid (120 mg, 0.97 mmol) according to general procedure 1. Product was purified *via* silica gel column chromatography eluted with EtOAc/hexane a ratio of 1:2 (v/v), followed by trituration with EtOAc/hexane a 1:2 (v/v) mixture to yield 3-methyl-2-neopentyl-6-phenylquinazolin-4(3*H*)-one **A9-36** as a white solid (116 mg, 58.3%).

LC-MS: 97.2% (Rt = 3.70), ESI(+) *m/z* found: 307.15 $[M+H]^+$. Molecular Weight calc'd for $C_{20}H_{22}N_2O$ = 306.40.

^{1}**H NMR** (500 MHz, $CDCl_3$) δ ppm: 8.49 (dd, *J* = 2.2, 0.5 Hz, 1H), 7.97 (dd, *J* = 8.5, 2.2 Hz, 1H), 7.74-7.65 (m, 3H), 7.50-7.43 (m, 2H), 7.41-7.33 (m, 1H), 3.68 (s, 3H), 2.83 (s, 2H), 1.15 (s, 9H).

^{13}**C NMR** (126 MHz, $CDCl_3$) δ ppm: 162.87, 155.65, 146.15, 139.84, 139.21, 133.00, 128.93, 127.68, 127.56, 127.17, 124.56, 120.35, 76.76, 46.77, 33.29, 32.19, 30.03.

1.2.2. *Chemotype* A1

General procedure for the synthesis of *N*-benzyl-1,3-oxazole-4-carboxamide **A1** derivatives

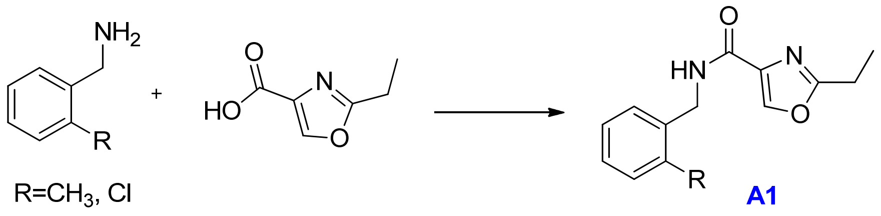

The derivatives of 1,3-oxazole-4-carboxylic acid or 1,3-thiazole-4-carboxylic acid (0.57 mmol) were dissolved in DCM (2 mL) and treated with 1.2 eq. of appropriate benzylamine (0.67 mmol), 2.2 eq. of *N,N*-diisopropylethylamine (1.24 mmol) and 1 eq. of BOP (0.57 mmol). The mixture was stirred at RT 0.5 h. The solvent was evaporated, and the crude product was purified *via* silica gel flash column chromatography eluting with $CHCl_3$ and using centrifugal preparative thin layer chromatography (CPTLC, chromatotron, SiO_2) eluting with hexane/$CHCl_3$/acetone a ratio of 40:30:1 (v/v).

2-Ethyl-*N*-(2-methylbenzyl)oxazole-4-carboxamide **A1-1**

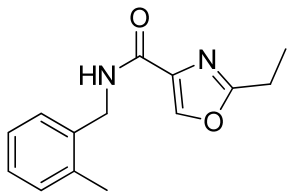

The compound was synthesized from 2-ethyl-1,3-oxazole-4-carboxylic acid and 1-(2-methylphenyl)methanamine according to the general procedure. There was obtained: 89 mg of a colorless solid product (yield: 79%).

LC-MS: 100% (Rt = 2.77), ESI(+) m/z found: 245.09 $[M+H]^+$. Molecular Weight calc'd for $C_{14}H_{16}N_2O_2$ = 244.29.

^{1}H NMR (500 MHz, DMSO-d_6) δ ppm: 8.56 (t, *J* = 6.2 Hz, 1H), 8.50 (s, 1H), 7.24-7.17 (m, 1H), 7.18-7.10 (m, 3H), 4.40 (d, *J* = 6.2 Hz, 2H), 2.80 (q, *J* = 7.6 Hz, 2H), 2.30 (s, 3H), 1.26 (t, *J* = 7.6 Hz, 3H).

^{13}C NMR (126 MHz, DMSO-d_6) δ ppm: 165.29, 160.05, 141.38, 137.05, 135.82, 135.27, 129.81, 127.16, 126.67, 125.67, 39.72, 20.89, 18.69, 10.88.

N-(2-chlorobenzyl)-2-ethyloxazole-4-carboxamide **A1-2**

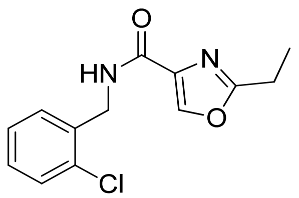

The compound was synthesized from 2-ethyl-1,3-oxazole-4-carboxylic acid and 2-chlorobenzylamine according to the general procedure. Obtained: 105 mg of a colorless solid product (yield: 87%).

LC-MS: 100% (Rt = 2.84), ESI(+) m/z found: 265.09 $[M+H]^+$. Molecular Weight calc'd for $C_{13}H_{13}ClN_2O_2$ = 264.71.

^{1}H NMR (500 MHz, DMSO-d_6) δ ppm: 8.74 (t, *J* = 6.2 Hz, 1H), 8.53 (s, 1H), 7.46-7.40 (m, 1H), 7.34-7.24 (m, 3H), 4.49 (d, *J* = 6.3 Hz, 2H), 2.82 (q, *J* = 7.6 Hz, 2H), 1.27 (t, *J* = 7.6 Hz, 3H).

^{13}C NMR (126 MHz, DMSO-d_6) δ ppm: 165.36, 160.36, 141.60, 136.21, 135.63, 131.74, 129.02, 128.43, 128.26, 127.09, 39.79, 20.90, 10.88.

1.2.3. Chemotype A2

General procedure for the synthesis of A2 derivatives

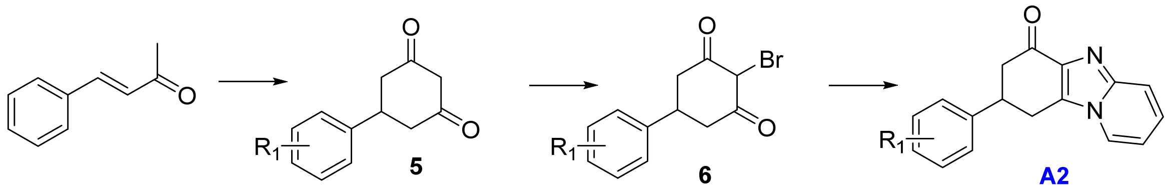

5 6 A2

General procedure for synthesis of intermediates 5a-d

The starting 5-phenylcyclohexane-1,3-dione derivatives **5** were synthesized according to the procedure reported by Kesten et al. [1]. To 3.2 g (0.14 mol) of sodium dissolved in 90 mL of anhydrous EtOH was added 23.0 g (0.14 mol) of diethyl malonate followed by 0.14 mol of 4-phenylbut-3-en-2-one. The solution was heated under reflux for 6 h, allowed to cool to room temperature overnight. The reaction mixture was concentrated in vacuo, suspended in water and washed with $CHCl_3$ to remove unchanged starting material and other organic-soluble impurities. The aqueous layer was concentrated in vacuo to a crude solid. To obtained solid 100 mL of 2 N NaOH was added, and the resulting solution was heated under reflux for 2 h. To the cooled, basic reaction mixture was added 100 mL of 5 N sulfuric acid. The resulted suspension was next heated under reflux for 4.5 h, allowed to cool to room temperature, and filtered, triturated with toluene and crystallized in acetone.

Procedure for synthesis of intermediates **6a-c**

Reaction was run according to previously reported methods [2,3]. To the vigorously stirred suspension of intermediate **5** (5 mmol) in acetic acid (7 mL) bromine (0.26 mL, 5 mmol) was added dropwise and stirred at RT for 2 h. The reaction mixture was filtered off, and the precipitate was washed with acetic acid and crystalized (water/EtOH).

Procedure for synthesis of A2 derivatives

A mixture of compound **6** (1 mmol) and 2-aminopyridine (2.5 mmol) in 10 mL acetonitrile was heated under reflux for 5 h, allowed to cool to room temperature, and the solvent was evaporated. MeOH was added to the residue, followed by slow addition of water until a precipitate formed. Next, the solid was filtered, dried, and purified *via* alumina (Al_2O_3) flash column chromatography eluting with hexane/EtOAc (2:1) to provide, after trituration with acetone/hexane and drying desired product.

5-phenylcyclohexane-1,3-dione (**5a**)

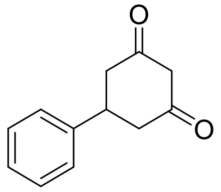

The compound was synthesized from 4-phenylbut-3-en-2-one according to the general procedure. There was obtained: 13.7 g of a colorless solid product (yield: 52%).

LC-MS: 100% (Rt = 2.19), ESI(+) m/z found: 189.08 [M+H]+. Molecular Weight calc'd for $C_{12}H_{12}O_2$ = 188.23.

5-(4-methoxyphenyl)cyclohexane-1,3-dione (**5b**)

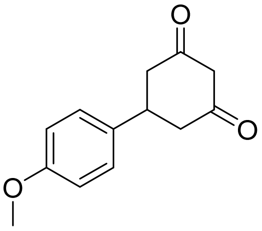

The compound was synthesized from 4-(4-methoxyphenyl)but-3-en-2-one according to the general procedure. There was obtained: 5.9 g of a colorless solid product (yield: 79.5%).

LC-MS: 100% (Rt = 2.26), ESI(+) m/z found: 219.17 [M+H]+. Molecular Weight calc'd for $C_{13}H_{14}O_3$ = 218.25.

5-(2,4-dimethylphenyl)cyclohexane-1,3-dione (**5c**)

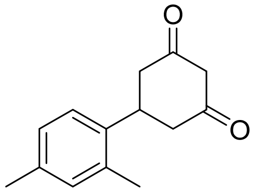

The compound was synthesized from 4-(2,4-dimethylphenyl)but-3-en-2-one according to the general procedure. There was obtained: 19.3 g of a colorless solid product (yield: 48%).

LC-MS: 97.2% (Rt = 2.65), ESI(+) m/z found: 216.97 [M+H]+. Molecular Weight calc'd for $C_{14}H_{16}O_2$ = 216.28.

5-(2-chlorophenyl)cyclohexane-1,3-dione (**5d**)

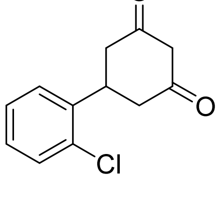

The compound was synthesized from 4-(2-chlorophenyl)but-3-en-2-one according to the general procedure. There was obtained: 3.2 g of a colorless solid product (yield: 15%).

LC-MS: 100% (Rt = 2.51), ESI(+) m/z found: 222.73 [M+H]+. Molecular Weight calc'd for $C_{12}H_{11}ClO_2$ = 222.67.

2-bromo-5-phenylcyclohexane-1,3-dione (**6a**)

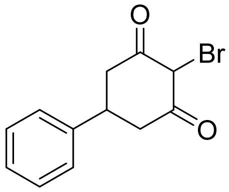

The compound was synthesized from intermediate **5a** according to the general procedure. There was obtained: 1.2 g of a yellow solid product (yield: 84%).

LC-MS: 73.0% (Rt = 2.37), ESI(+) m/z found: 267.09 $[M+H]^+$. Molecular Weight calc'd for $C_{12}H_{11}BrO_2$ = 267.12.

2-bromo-5-(4-methoxyphenyl)cyclohexane-1,3-dione (**6b**)

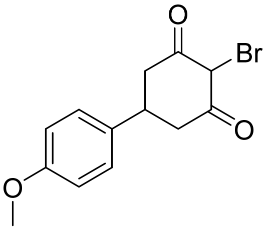

The compound was synthesized from intermediate **5b** according to the general procedure. There was obtained: 1.1 g of a yellow solid product (yield: 75%).

LC-MS: 100% (Rt = 2.71), ESI(+) m/z found: 297.08 $[M+H]^+$. Molecular Weight calc'd for $C_{13}H_{13}BrO_3$ = 297.15.

2-bromo-5-(2,4-dimethylphenyl)cyclohexane-1,3-dione (**6c**)

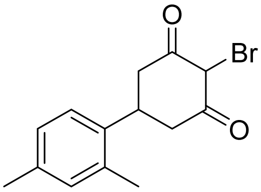

The compound was synthesized from intermediate 5c according to the general procedure. There was obtained: 0.89 g of a yellow solid product (yield: 60%).

LC-MS: 97.6% (Rt = 2.79), ESI(+) m/z found: 295.15 $[M+H]^+$. Molecular Weight calc'd for $C_{14}H_{15}BrO_2$ = 295.18.

8-(4-Methoxyphenyl)-8,9-dihydrobenzo[4,5]imidazo[1,2-*a*]pyridin-6(7*H*)-one **A2-1**

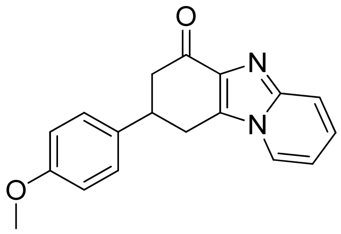

The compound was synthesized from 2-bromo-5-(4-methoxyphenyl)cyclohexane-1,3-dione (**6b**) according to the general procedure. Obtained: 8 mg of solid product (yield: 2.7%).

LC-MS: 97.5% (Rt = 2.46), ESI(+) m/z found: 293.34 $[M+H]^+$. Molecular Weight calc'd for $C_{18}H_{16}N_2O_2$ = 292.12.

^{1}H NMR (500 MHz, DMSO-d$_6$) δ ppm: 9.21 (dt, *J* = 6.7, 1.2 Hz, 1H), 7.77 (dt, *J* = 9.0, 1.1 Hz, 1H), 7.65 (ddd, *J* = 9.0, 6.9, 1.4 Hz, 1H), 7.39-7.29 (m, 2H), 7.25 (td, *J* = 6.9, 1.2 Hz, 1H), 6.94-6.85 (m, 2H), 3.65-3.55 (m, 1H), 3.32 (s, 3H), 3.23 (dd, *J* = 16.5, 10.7 Hz, 1H), 3.14 (ddd, *J* = 16.3, 4.9, 1.1 Hz, 1H), 3.01 (dd, *J* = 16.3, 12.0 Hz, 1H), 2.64 (ddd, *J* = 16.2, 4.0, 1.0 Hz, 1H).

8-(2,4-Dimethylphenyl)-8,9-dihydrobenzo[4,5]imidazo[1,2-*a*]pyridin-6(7*H*)-one **A2-2**

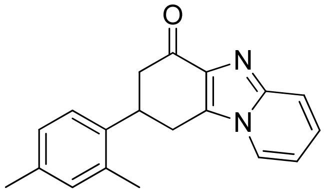

The compound was synthesized from 2-bromo-5-(2,4-dimethylphenyl)cyclohexane-1,3-dione (**6c**) according to the general procedure. Obtained: 9 mg of solid product (yield: 3%).

LC-MS: 100% (Rt = 2.91), ESI(+) m/z found: 291.04 $[M+H]^+$. Molecular Weight calc'd for $C_{19}H_{18}N_2O$ = 290.37.

^{1}H NMR (500 MHz, DMSO-d6) ppm: 9.31 (dt, *J* = 6.7, 1.2 Hz, 1H), 7.90 (dt, *J* = 9.0, 1.2 Hz, 1H), 7.83 (ddd, *J* = 8.8, 7.0, 1.3 Hz, 1H), 7.42 (td, *J* = 6.9, 1.2 Hz, 1H), 7.35 (d, *J* = 8.3 Hz, 1H), 7.05-6.99 (m, 2H), 3.85 (tt, *J* = 11.7, 4.2 Hz, 1H), 3.27 (dd, *J* = 16.6, 11.3 Hz, 1H), 3.13 (ddd, *J* = 16.6, 4.6, 1.1 Hz, 1H), 3.06 (dd, *J* = 16.4, 12.4 Hz, 1H), 2.58 (ddd, *J* = 16.3, 3.8, 1.2 Hz, 1H), 2.30 (s, 3H), 2.25 (s, 3H).

1.2.4. Chemotype A3

General procedure for the synthesis of A3 derivatives

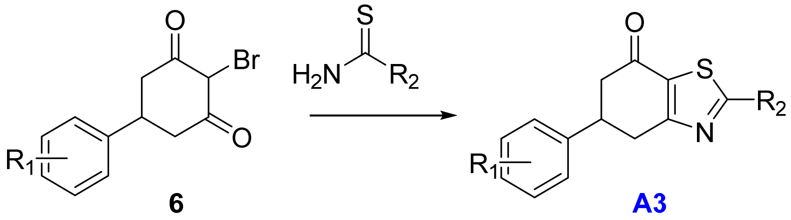

The starting compound **6** was synthesized according to method described for preparation of **A2** derivatives. A mixture of corresponding compound **6** (1 eq.) and propanethioamide or thiobenzamide (2.5 eq.) in 10 mL DMF was stirred for 1h. Water was added to the reaction mixture and the product was extracted with $CHCl_3$. The organic phase was washed with brine, dried over $MgSO_4$ and the solvent was evaporated under vacuum. Next, the residue was purified *via* alumina (Al_2O_3) flash column chromatography eluting with hexane/EtOAc a ratio of 6:1 (v/v).

2-Ethyl-5-phenyl-5,6-dihydrobenzo[d]thiazol-7(4*H*)-one A3-1

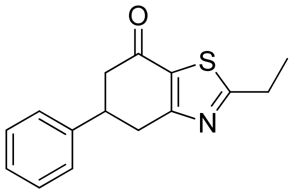

The compound was synthesized from 2-bromo-5-phenylcyclohexane-1,3-dione (**6a**) according to the general procedure. Obtained: 217 mg of yellow oil (yield: 53%).

LC-MS: 97.3% (Rt = 3.20), ESI(+) m/z found: 258.08 $[M+H]^+$. Molecular Weight calc'd for $C_{15}H_{15}NOS$ = 257.35.

^{1}**H NMR** (500 MHz, DMSO-d_6) δ ppm: 7.43-7.36 (m, 2H), 7.35 (ddt, *J* = 7.8, 6.4, 1.1 Hz, 2H), 7.29-7.22 (m, 1H), 3.66-3.55 (m, 1H), 3.27-3.13 (m, 2H), 3.06 (d, *J* = 7.5 Hz, 1H), 3.06-2.96 (m, 2H), 2.66 (ddd, *J* = 16.2, 3.9, 1.0 Hz, 1H), 1.31 (t, *J* = 7.5 Hz, 3H).

^{13}**C NMR** (126 MHz, DMSO-d_6) δ ppm: 190.87, 179.71, 165.90, 143.13, 129.37, 128.53(2C), 127.00(2C), 126.77, 44.36, 40.64, 34.23, 27.06, 13.59.

2-Ethyl-5-(4-methoxyphenyl)-5,6-dihydrobenzo[d]thiazol-7(4*H*)-one A3-2

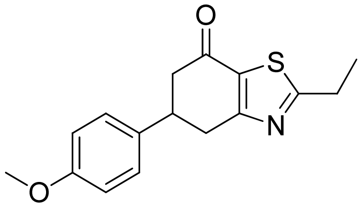

The compound was synthesized from 2-bromo-5-(4-methoxyphenyl)cyclohexane-1,3-dione (**6b**) according to the general procedure. Obtained: 233 mg of solid product (yield: 41%).

LC-MS: 97.8% (Rt = 3.16), ESI(+) m/z found: 288.06 $[M+H]^+$. Molecular Weight calc'd for $C_{16}H_{17}NO_2S$ = 287.38.

^{1}H NMR (500 MHz, DMSO-d$_6$) δ ppm: 7.34-7.27 (m, 2H), 6.93-6.86 (m, 2H), 3.73 (s, 3H), 3.59-3.49 (m, 1H), 3.23-3.08 (m, 2H), 3.04 (q, *J* = 7.5 Hz, 2H), 2.96 (dd, *J* = 16.3, 12.5 Hz, 1H), 2.63 (ddd, *J* = 16.3, 3.9, 1.1 Hz, 1H), 1.35-1.28 (m, 3H).

^{13}C NMR (126 MHz, DMSO-d$_6$) δ ppm: 191.02, 179.64, 165.98, 158.03, 135.12, 129.35, 127.97(2C), 113.88(2C), 55.04, 44.65, 39.90, 34.51, 27.04, 13.59.

5-(2,4-Dimethylphenyl)-2-ethyl-5,6-dihydrobenzo[*d*]thiazol-7(4*H*)-one **A3-3**

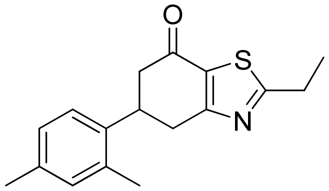

The compound was synthesized from 2-bromo-5-(2,4-dimethylphenyl)cyclohexane-1,3-dione (**6c**) according to the general procedure. Obtained: 109 mg of solid product (yield: 35%).

LC-MS: 100% (Rt = 3.57), ESI(+) m/z found: 286.31 $[M+H]^+$. Molecular Weight calc'd for $C_{17}H_{19}NOS$ = 285.41.

^{1}H NMR (500 MHz, DMSO-d$_6$) δ ppm: 7.32-7.26 (m, 1H), 7.03-6.97 (m, 2H), 3.74 (dtd, *J* = 13.0, 6.1, 3.0 Hz, 1H), 3.17-2.92 (m, 5H), 2.53 (ddd, *J* = 16.3, 3.8, 0.8 Hz, 1H), 2.28 (s, 3H), 2.24 (t, *J* = 0.7 Hz, 3H), 1.31 (t, *J* = 7.5 Hz, 3H).

^{13}C NMR (126 MHz, DMSO-d$_6$) δ ppm: 191.16, 179.57, 166.10, 138.10, 135.42, 135.06, 131.11, 129.29, 126.75, 125.65, 43.97, 36.36, 33.64, 27.05, 20.50, 18.89, 13.58.

2,5-Diphenyl-5,6-dihydrobenzo[*d*]thiazol-7(4*H*)-one **A3-4**

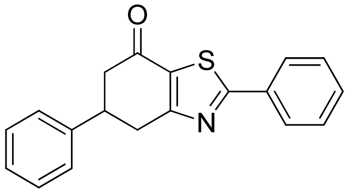

The compound was synthesized from 2-bromo-5-phenylcyclohexane-1,3-dione (**6a**) according to general procedure. Obtained: 127 mg of yellow oil (yield: 42%).

LC-MS: 100% (Rt = 3.67), ESI(+) m/z found: 306.38 $[M+H]^+$. Molecular Weight calc'd for $C_{19}H_{15}NOS$ = 305.40.

^{1}H NMR (500 MHz, DMSO-d$_6$) δ ppm: 8.09-8.02 (m, 2H), 7.63-7.52 (m, 3H), 7.46-7.39 (m, 2H), 7.40-7.32 (m, 2H), 7.27 (ddt, *J* = 7.9, 6.7, 1.3 Hz, 1H), 3.74-3.64 (m, 1H), 3.39-3.24 (m, 2H), 3.07 (dd, *J* = 16.3, 12.6 Hz, 1H), 2.73 (ddd, *J* = 16.2, 3.9, 1.0 Hz, 1H).

^{13}C NMR (126 MHz, DMSO-d$_6$) δ ppm: 191.03, 172.94, 166.93, 143.07, 132.17, 131.95, 129.86, 129.48(2C), 128.57(2C), 127.03(2C), 126.96(2C), 126.81, 44.39, 40.49, 34.27.

5-(2,4-Dimethylphenyl)-2-phenyl-5,6-dihydrobenzo[d]thiazol-7(4*H*)-one **A3-5**

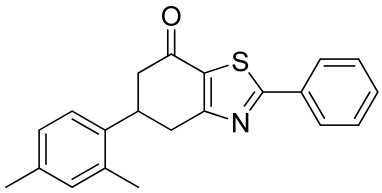

The compound was synthesized from 2-bromo-5-(2,4-dimethylphenyl)cyclohexane-1,3-dione (**6c**) according to the general procedure. Obtained: 119 mg of solid product (yield: 36%).

LC-MS: 92.7% (Rt = 4.16), ESI(+) m/z found: 334.11 $[M+H]^+$. Molecular Weight calc'd for $C_{21}H_{19}NOS$ = 333.45.

^{1}H NMR (500 MHz, DMSO-d_6) δ ppm: 8.09-8.02 (m, 2H), 7.63-7.52 (m, 3H), 7.35-7.29 (m, 1H), 7.04-6.98 (m, 2H), 3.83 (dddd, *J* = 13.0, 9.6, 6.3, 3.8 Hz, 1H), 3.27-3.15 (m, 2H), 3.04 (dd, *J* = 16.3, 12.8 Hz, 1H), 2.63-2.55 (m, 1H), 2.30 (s, 3H), 2.25 (s, 3H*). Traces of impurities visible on the spectrum*

^{13}C NMR (126 MHz, DMSO-d_6) δ ppm: 191.34, 172.84, 167.16, 138.09, 135.48, 135.14, 132.21, 131.94, 131.15, 129.82, 129.49(2C), 126.95(2C), 126.80, 125.71, 44.01, 36.23, 33.72, 20.52, 18.93.

1.2.5. Chemotype A4

General procedure for the synthesis of A4 derivatives

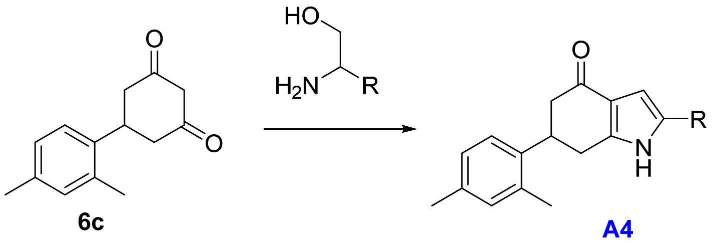

Reaction was run according to procedure described in patent EP1057812A1 [4]. A mixture of 5-(2,4-dimethylphenyl)cyclohexane-1,3-dione (**6c**) (1.2 g, 1 eq.), appropriate aminoalcohol (1,5 eq.), molecular sieves 4Å (7.2 g) and tetrahydrofuran (18 mL) was refluxed for 14 h and cooled, and insoluble materials were filtered off. The solvent was evaporated to give oil, which was dissolved in dimethylacetamide (30 mL). To the solution were added 2-bromomesitilene (1.1 eq.), tetrakistriphenylphosphine palladium (0.03 eq.), and K_2CO_3 (2.3 eq.), and the mixture was stirred at 150 °C for 10 h and concentrated under reduced pressure. The residue was dissolved in EtOAc, and the solution was washed with saturated brine, dried with K_2CO_3, and evaporated under reduced pressure to give desired *A4* derivatives.

6-(2,4-Dimethylphenyl)-2-ethyl-6,7-dihydro-1*H*-indol-4(5*H*)-one A4-1

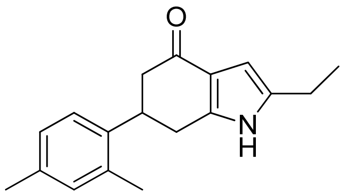

The compound was synthesized from compound **6c** and 2-aminobutan-1-ol according to the general procedure. The reaction mixture was purified with silica gel column chromatography (EtOAc) to give a colorless solid product (0.64 g, yield: 48%).

LC-MS: 100% (Rt = 2.96), ESI(+) m/z found: 267.95 $[M+H]^+$. Molecular Weight calc'd for $C_{18}H_{21}NO$ = 267.37.

^{1}H NMR (500 MHz, DMSO-d_6) δ ppm: 11.15 (s, 1H), 7.27 (d, *J* = 8.4 Hz, 1H), 7.01-6.94 (m, 2H), 5.99 (dt, *J* = 2.2, 1.0 Hz, 1H), 3.57 (dddd, *J* = 12.3, 10.9, 4.8, 3.7 Hz, 1H), 2.91 (dd, *J* = 16.0, 11.0 Hz, 1H), 2.83 (ddd, *J* = 15.9, 4.9, 1.1 Hz, 1H), 2.64 (dd, *J* = 16.1, 12.4 Hz, 1H), 2.55-2.47 (m, 2H), 2.31-2.21 (m, 7H), 1.16 (t, *J* = 7.5 Hz, 3H).

^{13}C NMR (126 MHz, DMSO-d_6) δ ppm: 191.50, 142.38, 138.96, 135.23, 135.09, 134.81, 131.01, 126.68, 125.64, 118.99, 100.25, 44.47, 37.50, 29.21, 20.49, 20.15, 18.90, 13.58.

6-(2,4-Dimethylphenyl)-2-phenyl-1,5,6,7-tetrahydro-4*H*-indol-4-one A4-2

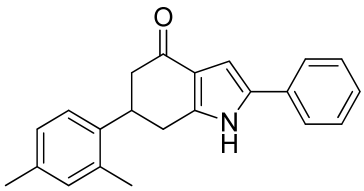

The compound was synthesized from 2-amino-2-phenylethan-1-ol according to the general procedure. The reaction mixture was purified with silica gel column chromatography (EtOAc) to give a colorless solid product (yield: 2.7%, maceration using 2-propanol/hexane mixture).

LC-MS: 100% (Rt = 3.29), ESI(+) m/z found: 316.14 $[M+H]^+$. Molecular Weight calc'd for $C_{22}H_{21}NO$ = 315.41.

^{1}H NMR (500 MHz, DMSO-d_6) δ ppm: 11.83 (s, 1H), 7.68 (d, *J* = 7.7 Hz, 2H), 7.39 (t, *J* = 7.6 Hz, 2H), 7.31 (d, *J* = 8.3 Hz, 1H), 7.22 (t, *J* = 7.4 Hz, 1H), 7.00 (d, *J* = 5.8 Hz, 2H), 6.77 (d, *J* = 2.2 Hz, 1H), 3.64 (td, *J* = 11.4, 5.6 Hz, 1H), 3.04 (dd, *J* = 16.1, 10.9 Hz, 1H), 2.96 (dd, *J* = 16.1, 4.9 Hz, 1H), 2.73 (dd, *J* = 16.1, 12.4 Hz, 1H), 2.35 (dd, *J* = 16.1, 3.7 Hz, 1H), 2.29 (s, 3H), 2.24 (s, 3H).

^{13}C NMR (126 MHz, DMSO-d_6) δ ppm: 191.88, 144.56, 138.81, 135.18, 134.87, 132.52, 131.81, 131.06, 128.80(2C), 126.74, 126.45, 125.68, 123.81(2C), 120.39, 101.57, 44.52, 37.23, 29.20, 20.51, 18.93.

2-Benzyl-6-(2,4-dimethylphenyl)-6,7-dihydro-1*H*-indol-4(5*H*)-one **A4-3**

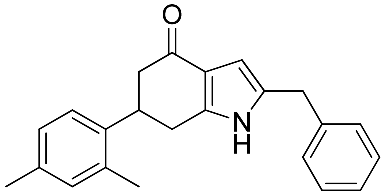

The compound was synthesized from compound **6c** and (2*R*)-2-amino-3-phenylpropan-1-ol according to the general procedure. The reaction mixture was purified with silica gel column chromatography (EtOAc) to give light cream solid product (yield: 7%, maceration using EtOAc/hexane mixture).

LC-MS: 100% (Rt = 3.34), ESI(+) m/z found: 330.21 [M+H]+. Molecular Weight calc'd for $C_{23}H_{23}NO$ = 329.43.

^{1}H NMR (500 MHz, DMSO-d_6) δ ppm: 11.24 (s, 1H), 7.32-7.19 (m 6H), 6.97 (br.s, 2H), 6.02 (d, *J* = 2.1 Hz, 1H), 3.85 (s, 2H), 3.60-3.53 (m, 1H), 2.86 (ddd, *J* = 20.7, 16.0, 7.9 Hz, 2H), 2.64 (dd, *J* = 16.1, 12.5 Hz, 1H), 2.28 (d, *J* = 3.4 Hz, 1H), 2.24 (d, *J* = 12.2 Hz, 6H).

^{13}C NMR (126 MHz, DMSO-d_6) δ ppm: 191.59, 142.97, 139.69, 138.93, 135.10, 134.82, 132.54, 131.02, 128.49(2C), 128.35(2C), 126.69, 126.14, 125.66, 119.17, 102.27, 44.48, 37.43, 33.20, 29.16, 20.49, 18.90.

1.2.6. Chemotype A5

General procedure for the synthesis of diphenyl-6,7-dihydrobenzofuran-4(5*H*)-one derivatives

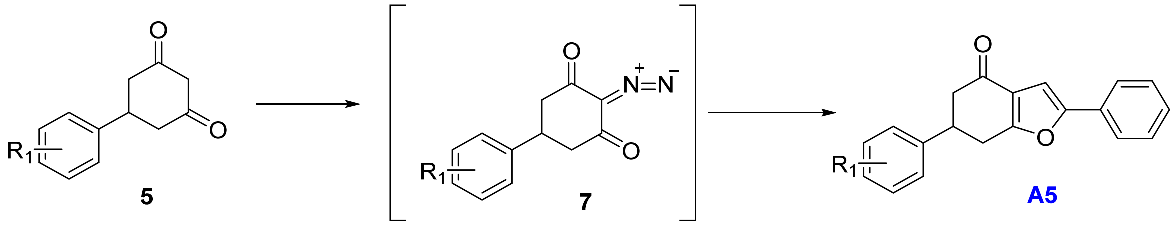

General procedure for synthesis of intermediates 7a-b

Reaction was run according to previously reported methods [5]. To the stirred solution of intermediate **5** (26 mmol) in MeCN (15 mL) was successively added tosyl azide (26 mmol) and K_2CO_3 (29 mmol) and stirred at RT for 13h. The mixture was then filtered through a pad of silica gel (rinsed out with CH_2Cl_2) and concentrated under vacuum to give the crude product. Next, the residue was purified *via* silica gel flash column chromatography eluting with hexane/EtOAc a ratio of 4:1 (v/v). The obtained products were stored at ca. –25 °C

General procedure for synthesis of A5 derivatives

Furan moiety was constructed *via* transition-metal-catalyzed [3+2] cycloaddition reactions between diazo compounds and acetylenes according to procedure reported by Xia et al. [6]. Tris(triphenylphosphine)ruthenium(II) dichloride ([Ru(PPh_3)$_3$$Cl_2$]; 0.02 mol, 2 mol-%) was added to a solution of a 2-diazo-5-arylcyclohexane-1,3-dione **7** (1.0 mmol, 1 eq.) and phenylacetylene (3.0 mmol, 3 equiv.) in toluene (2.0 mL) at room temperature. The reaction mixture was stirred at 70 °C for 3 days and then cooled to room temperature. Water (15 mL) was added, and the solution was extracted with EtOAc (3x15 mL). Evaporation of the solvent and purification by column chromatography on silica gel using hexane / EtOAc a ratio of 6:1 (v/v) as eluent gave the desired derivative *A5*.

2-diazo-5-phenylcyclohexane-1,3-dione (**7a**)

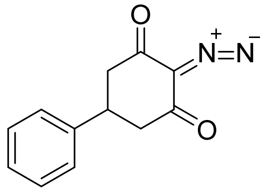

The compound was synthesized from intermediate **5a** according to the general procedure. There was obtained: 3.34 g of a yellow solid product (yield: 60%).

LC-MS: 92.4% (Rt = 2.42), ESI(+) m/z found: 215.10 $[M+H]^+$. Molecular Weight calc'd for $C_{12}H_{10}N_2O_2$ = 214.22.

2-diazo-5-(2,4-dimethylphenyl)cyclohexane-1,3-dione (**7b**)

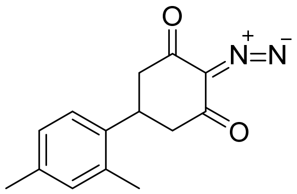

The compound was synthesized from intermediate 5c according to the general procedure. There was obtained: 3.90 g of a yellow solid product (yield: 62%).

LC-MS: 95.1% (Rt = 2.90), ESI(+) m/z found: 243.03 $[M+H]^+$. Molecular Weight calc'd for $C_{14}H_{14}N_2O_2$ = 242.28.

2,6-Diphenyl-6,7-dihydrobenzofuran-4(5*H*)-one **A5-1**

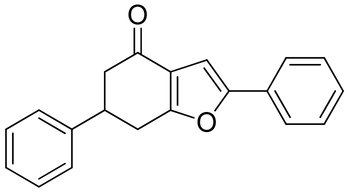

The compound was synthesized from 2-diazo-5-phenylcyclohexane-1,3-dione (2.33 mmol) and phenylacetylene (7.00 mmol) according to the general procedure. Obtained: 210 mg of a colorless solid product (yield: 31%, crystallization in 2-propanol/hexane).

LC-MS: 100% (Rt = 3.82), ESI(+) m/z found: 289.15 $[M+H]^+$. Molecular Weight calc'd for $C_{20}H_{16}O_2$ = 288.35.

^{1}H NMR (500 MHz, $CDCl_3$) δ ppm: 7.70 (dt, *J* = 2.9, 1.6 Hz, 2H), 7.47-7.38 (m, 4H), 7.37-7.30 (m, 4H), 6.96 (s, 1H), 3.67-3.59 (m, 1H), 3.29 (dd, *J* = 17.2, 5.1 Hz, 1H), 3.16 (dd, *J* = 17.2, 11.0 Hz, 1H), 2.86-2.81 (m, 2H).

^{13}C NMR (126 MHz, $CDCl_3$) δ ppm: 192.53, 165.30, 154.28, 141.96, 129.25, 128.44, 128.36(2C), 127.73, 126.81, 126.33(2C), 123.54(2C), 122.34, 100.38, 44.48, 40.78, 30.81.

6-(2,4-Dimethylphenyl)-2-phenyl-6,7-dihydrobenzofuran-4(5*H*)-one **A5-2**

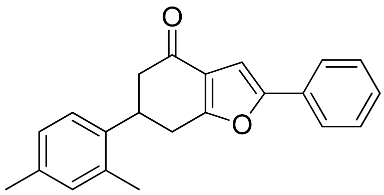

The compound was synthesized from 2-diazo-5-(2,4-dimethylphenyl)cyclohexane-1,3-dione (1.00 mmol) and phenylacetylene (3.00 mmol) according to the general procedure. Obtained: 60 mg of a yellow solid product (yield: 19%).

LC-MS: 100% (Rt = 4.02), ESI(+) m/z found: 317.21 $[M+H]^+$. Molecular Weight calc'd for $C_{22}H_{20}O_2$ = 316.40.

^{1}**H NMR** (500 MHz, DMSO-d$_6$) δ ppm: 7.76 (dt, *J* = 8.1, 1.5 Hz, 2H), 7.47-7.41 (m, 2H), 7.37-7.28 (m, 2H), 7.23 (s, 1H), 7.00 (d, *J* = 5.4 Hz, 2H), 3.81-3.74 (m, 1H), 3.20-3.09 (m, 2H), 2.86 (dd, *J* = 16.2, 12.5 Hz, 1H), 2.44 (dd, *J* = 16.2, 3.8 Hz, 1H), 2.30 (s, 3H), 2.24 (s, 3H).

^{13}**C NMR** (126 MHz, DMSO-d$_6$) δ ppm: δ 192.67, 166.59, 153.62, 137.99, 135.49, 135.12, 131.12, 129.33, 128.97, 128.16, 126.77, 125.75, 123.71, 122.03, 101.46, 44.06, 35.90, 29.75, 20.50, 18.91.

1.2.7. Chemotype A6

General procedure for the synthesis of **A6** derivatives

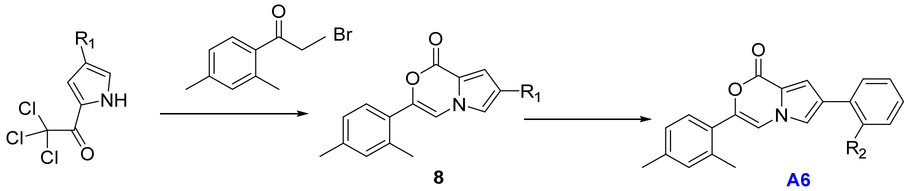

Procedure for synthesis of intermediate **8**

Compound **8** was synthesized according to the method reported in WO2009/130231[7]. To a solution of 2,2,2-trichloro-1-(1*H*-pyrrol-2-yl)ethan-1-one or 1-(4-bromo-1*H*-pyrrol-2-yl)-2,2,2-trichloroethan-1-one (1.7 mmol, 1 eq.) in DMF (6 mL), potassium carbonate (5.11 mmol, 3 eq.) and 2-bromo-1-(2,4-dimethylphenyl)ethan-1-one (2.56 mmol, 1.5 eq.) were added and the mixture was stirred at RT for 6 h. Cold water was added to the reaction mixture and the product was extracted with EtOAc. The organic phase was washed with brine, dried over $MgSO_4$ and the solvent was evaporated under vacuum to give desired compound (**8**).

Procedure for **A6** derivative synthesis

7-Bromo-3-(2,4-dimethylphenyl)-1*H*-pyrrolo[2,1-*c*][1,4]oxazin-1-one (**8**) (0.31 mmol, 1 eq.), appropriate phenylboronic acid (1.6 eq.), tetrakis(triphenylphosphine)palladium(0) (0.015 mmol, 0.05 eq.), 2M aqueous solution of potassium carbonate (0.31 mL, 2.0 eq.), toluene (2.5 mL) were sealed under argon. Reaction mixture was heated at 100 °C for 1.5 h in microwave oven (150 W), after cooling distilled water was added (50 mL) and reaction mixture was extracted with EtOAc (3x50 mL). The combined organic extract was washed with distilled water (50 mL), brine (30 mL) dried over anhydrous magnesium sulfate and evaporated under reduced pressure to give desired product (**A6**).

7-Bromo-3-(2,4-dimethylphenyl)-1*H*-pyrrolo[2,1-*c*][1,4]oxazin-1-one (**8**)

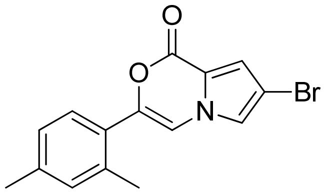

The 7-bromo-3-(2,4-dimethylphenyl)-1*H*-pyrrolo[2,1-*c*][1,4]oxazin-1-one was prepared from 1-(4-bromo-1*H*-pyrrol-2-yl)-2,2,2-trichloroethan-1-one according to the general procedure. The crude

product was purified by flash chromatography eluted with EtOAc/hexane a ratio of 1:9 (v/v) to give the title compound (720 mg, 65%) as light yellow solid.

LC-MS: 98.7% (Rt = 4.07), ESI(+) m/z found: 318.19 $[M+H]^+$. Molecular Weight calc'd for $C_{14}H_{12}BrNO_2$ = 318.165.

3-(2,4-Dimethylphenyl)-1*H*-pyrrolo[2,1-*c*][1,4]oxazin-1-one **A6-1**

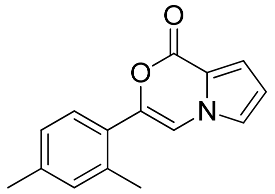

The 3-(2,4-dimethylphenyl)-1*H*-pyrrolo[2,1-*c*][1,4]oxazin-1-one was prepared from 2,2,2-trichloro-1-(1*H*-pyrrol-2-yl)ethan-1-one according to the general procedure. The crude product was purified by flash chromatography eluted with EtOAc/hexane a ratio of 1:9 (v/v) to give the title compound (610 mg, 45%) as light yellow solid.

LC-MS: 96.7% (Rt = 3.20), ESI(+) m/z found: 240.27 $[M+H]^+$. Molecular Weight calc'd for $C_{15}H_{13}NO_2$ = 239.27.

^{1}H NMR (500 MHz, DMSO-d_6) δ ppm: 7.79 (d, *J* = 0.8 Hz, 1H), 7.57 (dd, *J* = 2.5, 1.4 Hz, 1H), 7.37 (d, *J* = 7.8 Hz, 1H), 7.21 (ddd, *J* = 4.0, 1.5, 0.7 Hz, 1H), 7.17-7.06 (m, 2H), 6.65 (dd, *J* = 4.1, 2.5 Hz, 1H), 2.38 (s, 3H), 2.32 (s, 3H).

^{13}C NMR (126 MHz, DMSO-d_6) δ ppm: 154.36, 141.47, 139.17, 136.17, 131.53, 128.92, 127.65, 126.66, 122.87, 115.74, 114.69, 113.27, 107.81, 20.75, 20.07.

3-(2,4-Dimethylphenyl)-7-phenyl-1*H*-pyrrolo[2,1-*c*][1,4]oxazin-1-one **A6-2**

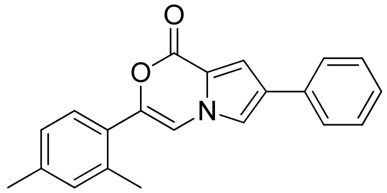

The 3-(2,4-dimethylphenyl)-7-phenyl-1*H*-pyrrolo[2,1-*c*][1,4]oxazin-1-one was prepared from 7-bromo-3-(2,4-dimethylphenyl)-1*H*-pyrrolo[2,1-*c*][1,4]oxazin-1-one (**8**) and phenylboronic acid (1.6 eq) according to the general procedure. The crude product was purified by centrifugal thin-layer chromatograph (Chromatotrone, Harrison Research; silica gel; eluent hexane/EtOAc a ratio of 9:1 (v/v)) to give colorless solid (101 mg, 37%).

LC-MS: 99.1% (Rt = 3.92), ESI(+) m/z found: 316.21 $[M+H]^+$. Molecular Weight calc'd for $C_{21}H_{17}NO_2$ = 315.37.

^{1}H NMR (500 MHz, DMSO-d_6) δ ppm: 8.01 (d, *J* = 1.7 Hz, 1H), 7.79-7.70 (m, 3H), 7.67 (dd, *J* = 1.8, 0.7 Hz, 1H), 7.46-7.37 (m, 3H), 7.32-7.23 (m, 1H), 7.19-7.10 (m, 2H), 2.40 (s, 3H), 2.33 (s, 3H).

^{13}C NMR (126 MHz, DMSO-d_6) δ ppm: 154.25, 141.87, 139.27, 136.22, 132.96, 131.58, 128.97, 128.95, 128.16, 127.60, 127.01, 126.70, 125.39, 119.32, 116.71, 111.61, 107.65, 20.79, 20.10.

7-(2-Chlorophenyl)-3-(2,4-dimethylphenyl)-1*H*-pyrrolo[2,1-*c*][1,4]oxazin-1-one **A6-3**

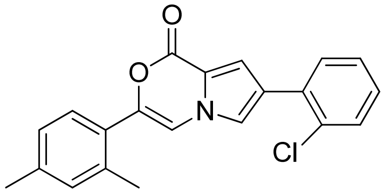

The compound was prepared from 7-bromo-3-(2,4-dimethylphenyl)-1*H*-pyrrolo[2,1-*c*][1,4]oxazin-1-one (**8**) and 2-chlorophenylboronic acid according to the general procedure 2. The crude product was purified by centrifugal thin-layer chromatograph (Chromatotrone, Harrison Research; silica gel; 9:1, hexane/EtOAc) to give colorless solid (24 mg, 17%).

LC-MS: 96.8% (Rt = 3.96), ESI(+) m/z found: 350.14 $[M+H]^+$. Molecular Weight calc'd for $C_{21}H_{16}ClNO_2$ = 349.81.

^{1}H NMR (500 MHz, DMSO-d_6) δ ppm: 8.02 (d, *J* = 1.7 Hz, 1H), 7.84 (d, *J* = 0.7 Hz, 1H), 7.68 (dd, *J* = 7.7, 1.7 Hz, 1H), 7.58-7.53 (m, 2H), 7.45-7.36 (m, 2H), 7.34 (td, *J* = 7.7, 1.7 Hz, 1H), 7.18-7.10 (m, 2H), 2.40 (s, 3H), 2.32 (s, 3H).

^{13}C NMR (126 MHz, DMSO-d_6) δ ppm: 154.21, 142.02, 139.29, 136.22, 131.80, 131.57, 130.84, 130.81, 130.32, 128.92, 128.73, 127.69, 127.56, 126.69, 124.93, 121.90, 115.89, 114.42, 107.55, 20.77, 20.10.

1.2.8. Chemotype A7

Procedure for the synthesis of **A7-1** derivative

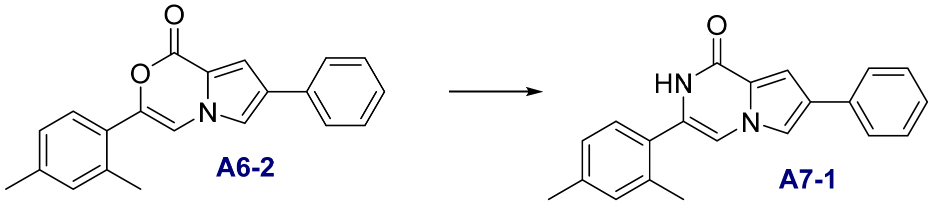

3-(2,4-Dimethylphenyl)-7-phenylpyrrolo[1,2-*a*]pyrazin-1(2*H*)-one **A7-1**

3-(2,4-dimethylphenyl)-7-phenyl-1*H*-pyrrolo[2,1-*c*][1,4]oxazin-1-one **A6-2** (100 mg, 0.317 mmol in acetic acid (3 mL), ammonium acetate (358 mg, 4.65 mmol) was added and the reaction mixture was heated at 140 °C for 1 h in microwave oven (300 W), after cooling distilled water was added (50 mL) and reaction mixture was extracted with EtOAc (3x50 mL). Combined organic extracts were washed with distilled water (50 mL), brine (30 mL) dried over anhydrous magnesium sulfate and evaporated under reduced pressure. Product was purified by flash chromatography (silica gel; 2:1, hexane/EtOAc) to give light brown solid (23 mg, 25%).

LC-MS: 95.8% (Rt = 3.45), ESI(+) m/z found: 315.28 $[M+H]^+$. Molecular Weight calc'd for $C_{21}H_{18}N_2O$ = 314.38.

^{1}**H NMR** (500 MHz, DMSO-d_6) δ ppm: 10.69 (d, *J* = 1.6 Hz, 1H), 7.87 (d, *J* = 1.7 Hz, 1H), 7.72-7.66 (m, 2H), 7.44-7.35 (m, 2H), 7.32 (dd, *J* = 1.8, 0.7 Hz, 1H), 7.28-7.19 (m, 3H), 7.16-7.04 (m, 2H), 2.31 (d, *J* = 10.2 Hz, 6H).

^{13}**C NMR** (126 MHz, DMSO-d_6) δ ppm: 155.80, 138.48, 136.61, 133.99, 130.97, 129.73, 129.53, 128.87, 127.03, 126.90, 126.47, 126.37, 125.27, 123.93, 115.94, 106.30, 105.87, 20.75, 19.61.

1.2.9. Chemotype A8

General procedure for the synthesis of A8 derivatives

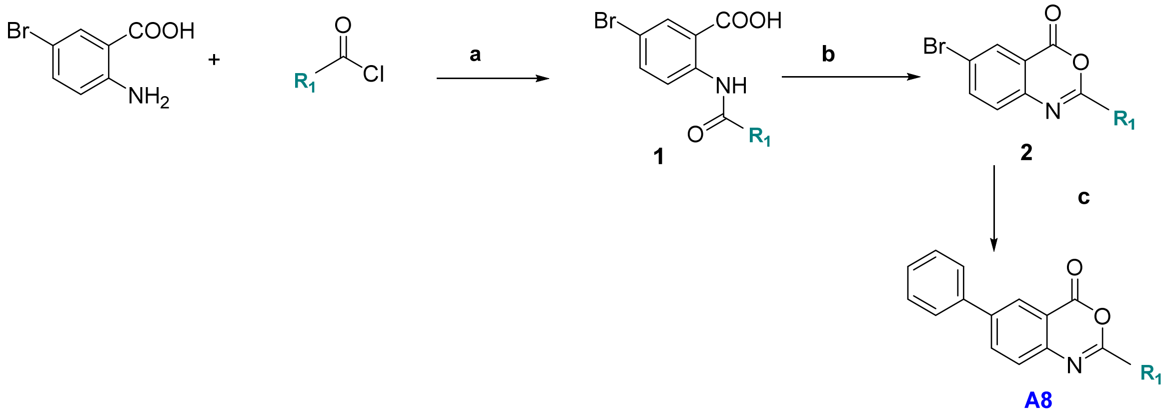

*Syntheses of intermediate **1** and **2** were described in section 1.2.1 (preparation of A9 derivative).*

Procedure for the synthesis of A8 derivatives

A mixture of compound **2** (0.66 mmol, 1.0 eq) and corresponding boronic acid (0.99 mmol, 1.5 eq.), K_2CO_3 (1.98 mmol, 3.0 eq.) was suspended in 1,4-dioxane (1 mL) and toluene (4 mL), degassed with argon. Then Pd(dppf)Cl_2 complex in DCM (0.033 mmol, 0.05 eq.) was added. Reaction was run in a sealed tube at 80 °C for 1-12 h. Then the mixture was cooled down to RT, quenched with water and extracted thrice with chloroform. The combined organic extracts were washed with brine, dried over $MgSO_4$ and evaporated on rotovap. Crude product was purified via silica gel column chromatography eluted with hexane/ethyl acetate a ratio of 3:1=>2:1 (v/v), followed by trituration with 2-PrOH/hexane a 1:3 (v/v) mixture to give desirable A8 derivative.

2,6-Diphenyl-4*H*-benzo[*d*][1,3]oxazin-4-one **A8-1**

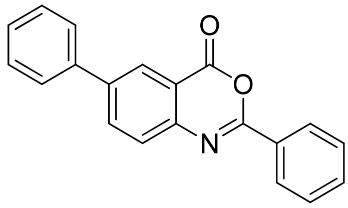

2,6-diphenyl-4*H*-benzo[*d*][1,3]oxazin-4-one **A8-1** was prepared according to general procedures. Yields: intermediates **1** (87.4%), **2** (95.4%) and 2,6-diphenyl-4H-benzo[d][1,3]oxazin-4-one **A8-1** (55.6%).

LC-MS: 95.0% (Rt = 4.07), ESI(+) m/z found: 300.12 $[M+H]^+$. Molecular Weight calc'd for $C_{20}H_{13}NO_2$ = 299.32.

^{1}**H NMR** (500 MHz, DMSO-d$_6$) δ ppm: 8.36-8.34 (m, 1H), 8.26 (dd, *J* = 8.4, 2.3 Hz, 1H), 8.24-8.19 (m, 2H), 7.83-7.79 (m, 3H), 7.70-7.65 (m, 1H), 7.64-7.58 (m, 2H), 7.56-7.50 (m, 2H), 7.47-7.43 (m, 1H).

^{13}**C NMR** (126 MHz, DMSO-d$_6$) δ ppm: 158.93, 156.40, 145.51, 140.12, 138.06, 135.06, 132.79, 130.03, 129.25(2C), 129.07(2C), 128.37, 127.84(2C), 127.66, 126.85(2C), 125.26, 117.50.

2-(2-Chlorophenyl)-6-phenyl-4*H*-benzo[*d*][1,3]oxazin-4-one **A8-2**

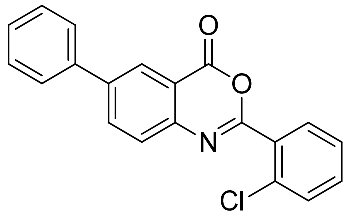

2-(2-Chlorophenyl)-6-phenyl-4*H*-benzo[*d*][1,3]oxazin-4-one **A8-2** was prepared according to general procedures. Yields: intermediates **1** (81.9%), **2** (75.4%) and 2-(2-chlorophenyl)-6-phenyl-4*H*-benzo[*d*][1,3]oxazin-4-one **A8-2** (77.9%).

LC-MS: 95.4% (Rt = 4.03), ESI(+) m/z found: 334.04 [M+H]$^+$. Molecular Weight calc'd for $C_{20}H_{12}ClNO_2$ = 333.77.

^{1}**H NMR** (500 MHz, DMSO-d$_6$) δ ppm: 8.36 (d, *J* = 2.0 Hz, 1H), 8.30-8.25 (m, 1H), 7.98-7.92 (m, 1H), 7.80 (ddd, *J* = 8.3, 2.9, 1.7 Hz, 3H), 7.65 (dtd, *J* = 8.1, 7.6, 1.5 Hz, 2H), 7.58-7.50 (m, 3H), 7.48-7.42 (m, 1H).

^{13}**C NMR** (126 MHz, DMSO-d$_6$) δ ppm: 158.75, 155.70, 144.99, 140.87, 137.95, 135.20, 132.92, 131.87, 131.75, 130.77, 130.06, 129.24(2C), 128.47, 127.83, 127.52, 126.93(2C), 125.25, 117.32.

2-(4-Methoxyphenyl)-6-phenyl-4*H*-benzo[*d*][1,3]oxazin-4-one **A8-3**

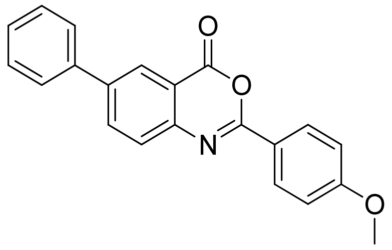

2-(4-methoxyphenyl)-6-phenyl-4*H*-benzo[*d*][1,3]oxazin-4-one **A8-3** was prepared according to general procedures. Yields: intermediates **1** (95.6%), **2** (85.3%), 2-(4-methoxyphenyl)-6-phenyl-4*H*-benzo[d][1,3]oxazin-4-one **A8-3** (91.9%).

LC-MS: 95.5% (Rt = 4.03), ESI(+) m/z found: 330.11 [M+H]$^+$. Molecular Weight calc'd for $C_{21}H_{15}NO_3$ = 329.35.

^{1}**H NMR** (500 MHz, $CDCl_3$) δ ppm: 8.43-8.41 (m, 1H), 8.28-8.24 (m, 2H), 8.03 (dd, *J* = 8.4, 2.2 Hz, 1H), 7.72-7.68 (m, 1H), 7.68-7.64 (m, 2H), 7.51-7.45 (m, 2H), 7.42-7.37 (m, 1H), 7.02-6.97 (m, 2H), 3.89 (s, 3H).

^{13}**C NMR** (126 MHz, DMSO-d$_6$) δ ppm: 163.28, 159.90, 157.00, 146.39, 140.67, 138.95, 135.16, 130.27(2C), 129.08(2C), 128.17, 127.39, 127.03(2C), 126.41, 122.53, 116.98, 114.16(2C), 55.51.

1.2.10. Chemotype A10

Procedure for the synthesis of A10-1 derivative

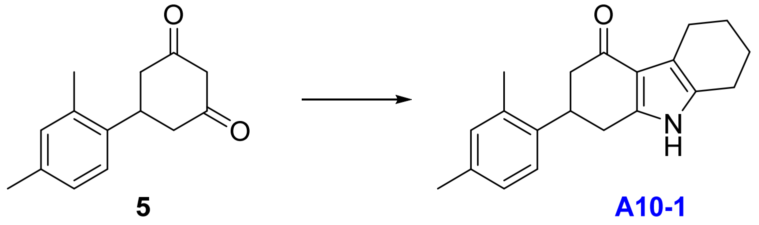

2-(2,4-Dimethylphenyl)-2,3,4,5,6,7,8,9-octahydro-1*H*-carbazol-4-one A10-1

A mixture of 5-(2,4-dimethyl phenyl)cyclohexane-1,3-dione (**5c**) (0.5 g, 1 eq.), 2-aminocyclohexan-1-ol (1,5 eq.), molecular sieves 4Å (3 g) and tetrahydrofuran (7.5 mL) was refluxed for 14 h and cooled, and insoluble material was filtered off. The solvent was evaporated to give oil, which was dissolved in dimethylacetamide (8 mL). To the solution were added 2-bromomesitilene (1.1 eq.), tetrakistriphenylphosphine palladium (0.03 eq.) and K_2CO_3 (2.3 eq.), and the mixture was stirred at 150°C for 10h and concentrated under reduced pressure. The residue was dissolved in EtOAc, and the solution was washed with saturated brine, dried with K_2CO_3, and concentrated under reduced pressure. Finally, the crude product was purified with silica gel column chromatography eluted with hexane/EtOAc a ratio of 4:1 (v/v) to give a solid product (yield: 3.4%).

LC-MS: 88.2% (Rt = 3.27), ESI(+) m/z found: 294.21 $[M+H]^+$. Molecular Weight calc'd for $C_{20}H_{23}NO$ = 293.41.

^{1}H NMR (500 MHz, DMSO-D6) δ ppm: 11.12 (s, 1H), 7.25 (d, *J* = 8.4 Hz, 1H), 6.98 (dd, *J* = 4.5, 2.5 Hz, 2H), 3.57-3.49 (m, 1H), 3.46-3.32 (m, 3H), 3.28 (dd, *J* = 10.8, 5.7 Hz, 1H), 2.86 (dd, *J* = 15.9, 10.9 Hz, 1H), 2.79 (dd, *J* = 15.9, 4.9 Hz, 1H), 2.65-2.54 (m, 3H), 2.44 (d, *J* = 6.5 Hz, 2H), 2.24 (d, *J* = 10.8 Hz, 7H).

^{13}C NMR (126 MHz, DMSO-D6) δ ppm: 192.23, 141.72, 138.98, 135.04, 134.78, 131.01, 127.36, 126.66, 125.54, 116.62, 114.40, 44.97, 37.57, 29.44, 22.97, 22.62, 22.42, 21.95, 20.49, 18.85.

1.2.11. Chemotypes A11 and A12

General procedure for the synthesis of A11 and A12 derivatives

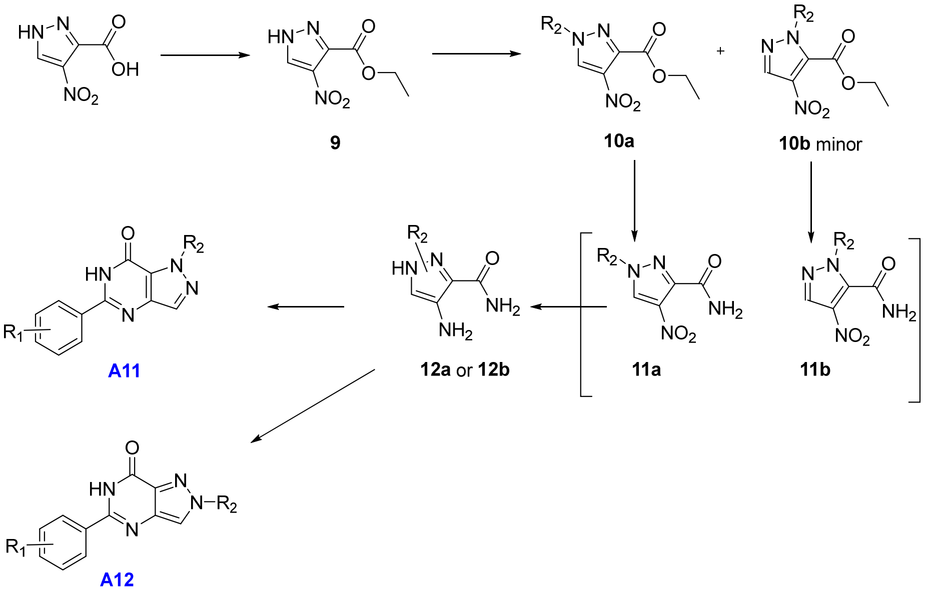

Procedure for the synthesis of ethyl 4-nitro-1*H*-pyrazole-3-carboxylate (**9**)

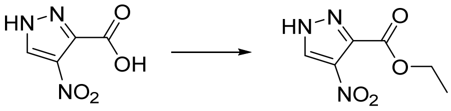

9

Compound **9** was synthesized according to US2007/00027166A1 [8]. 4-nitro-3-pyrazolecarboxylic acid (2 g, 12.73 mmol, 1.0 eq.) was suspended in EtOH (12 mL), treated with *p*-toluenesulfonic acid monohydrate (0.12 g, 064 mmol, 0.05 eq.) and heated under reflux for 3 h. Then the reaction mixture was stirred at RT/overnight. The reaction mixture was evaporated to dryness, diluted with 20 mL EtOAc and quenched with 20 mL of sat. $NaHCO_3$ solution. The mixture was stirred for 15 min and then product was extracted to EtOAc (5x). Combined organic layer was dried over MgSO4 and evaporated to dryness to give ethyl 4-nitro-1*H*-pyrazole-3-carboxylate (I) (2.08 g, 88.2%).

LC-MS: 100% (Rt = 1.76), ESI(+) m/z found: 186.74 $[M+H]^+$. Molecular Weight calc'd for $C_6H_7N_3O_4$ = 185.14.

Procedure for synthesis of ethyl 1-ethyl-4-nitro-1*H*-pyrazole-3-carboxylate intermediates **10a** and **10b**

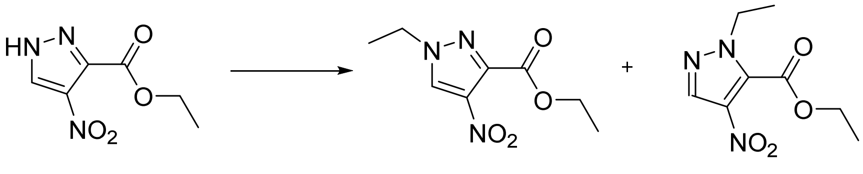

9 **10a** major **10b** minor

The iodoethane (842 mg, 5.4 mmol) was slowly dropped to a mixture of ethyl 4-nitro-1*H*-pyrazole-3-carboxylate **9** (500 mg, 2.7 mmol, 1.0 eq.) and potassium carbonate (746 mg, 5.4 mmol, 2.0 eq.) in acetone (10 mL). Then the reaction mixture was reflux for 1.5 h, cooled down to RT, diluted with EtOAc and quenched with water. Product was extracted thrice to EtOAc, dried over $MgSO_4$ and evaporated. The residue was purified *via* silica gel column chromatography eluted with hexane/EtOAc a ratio of 2:1=>1:1 (v/v) to give ethyl 1-ethyl-4-nitro-1*H*-pyrazole-3-carboxylate **10a** (320 mg, 55.5%) and ethyl 1-ethyl-4-nitro-1*H*-pyrazole-5-carboxylate **10b** (239 mg, 41.5%).

LC-MS (10a): 96.8% (Rt = 2.44), ESI(+) m/z found: 214.17 $[M+H]^+$. Molecular Weight calc'd for $C_8H_{11}N_3O_4$ = 213.19.

Procedure for synthesis of ethyl 1-benzyl-4-nitro-1*H*-pyrazole-3-carboxylate intermediates **10c** and **10d**

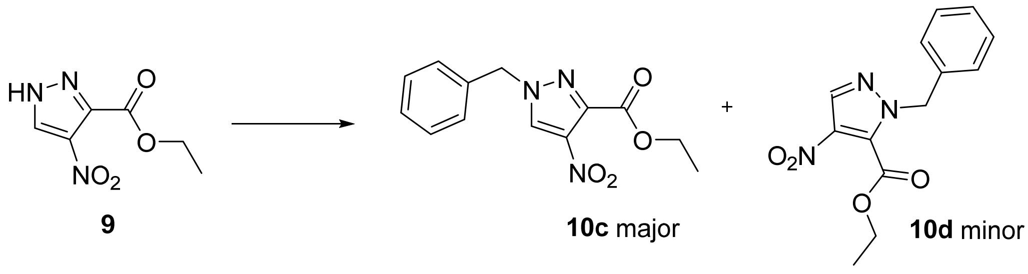

Compounds **10c** and **10d** were prepared from ethyl 4-nitro-1*H*-pyrazole-3-carboxylate according to the general procedure used for **10a.** The crude product was purified by flash chromatography eluted with chloroform: hexane a ratio of 1:2 (v/v) to give the title compound (175 mg, 59%) as a white solid.

LC-MS(10c): 95.4% (Rt = 3.09), ESI(+) m/z found: 276.02 $[M+H]^+$. Molecular Weight calc'd for $C_8H_{11}N_3O_4$ = 275.26.

Procedure for amination and reduction of nitro group

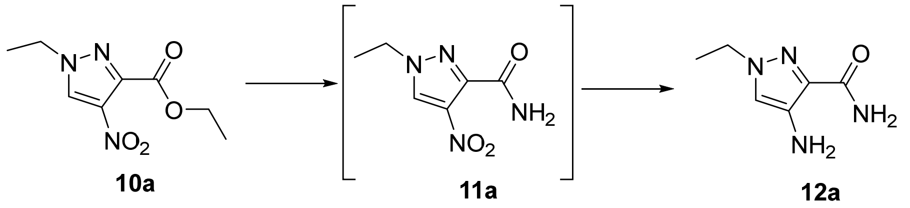

Starting ethyl 1-ethyl-4-nitro-1*H*-pyrazole-3-carboxylate (**10a**) (1.24 g, 5.8 mmol) was treated with 6 M NH_3 in MeOH (10 mL) and heated in a sealed tube (bath temp. 65 °C) for 6 h. The reaction mixture was evaporated to dryness and crude solid 1-ethyl-4-nitro-1*H*-pyrazole-3-carboxamide intermediate (**11a**) was dissolved in MeOH (25 mL), degassed with argon and Pd/C (10% wet) (0.124 g, 0.10 eq) was added. The reaction mixture was hydrogenated under hydrogen pressure (filled balloon) at RT/overnight. The catalyst was filtered off through the celite pad, washed with MeOH and the filtrate was evaporated. The crude product was purified by trituration with 2-PrOH/hexane a 1:3 (v/v) mixture to give 4-amino-1-ethyl-1*H*-pyrazole-3-carboxamide (0.85 g, 94%) **12a**. (Note: The same procedure was used for **12b** synthesis).

LC-MS: 100% (Rt =0.44), ESI(+) m/z found: 155.00 $[M+H]^+$. Molecular Weight calc'd for $C_6H_{10}N_4O$ = 154.17.

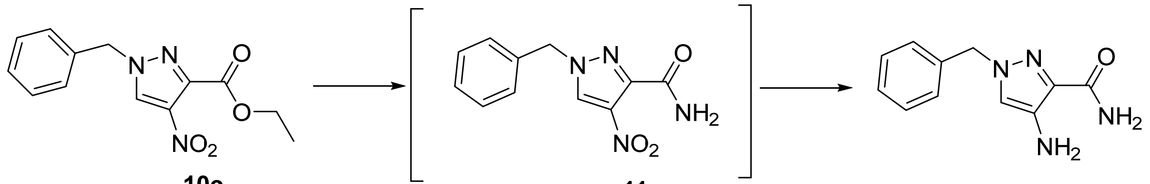

10c **11c** **12c**

Compounds **12c** was prepared from compound **10c** according to the general procedure used for **12a.** The crude product was purified by flash chromatography eluted with chloroform : hexane a ratio of 1:2 (v/v) to give the title compound (139 mg, 65.3%) as a white solid.

LC-MS (**12c**): 94.72% (Rt = 1.02), ESI(+) m/z found: 217.02 $[M+H]^+$. Molecular Weight calc'd for $C_8H_{11}N_3O_4$ = 216.24.

General procedure for preparation of A-11 or A12 compounds

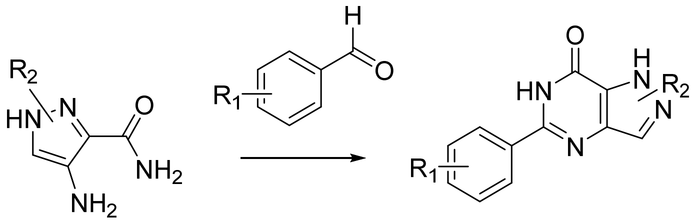

12 **A-11** or **A-12**

A11 and A12 were prepared according to *J. Med. Chem.* 2009, 52, 5013–5016 [9] The intermediates **12a** (or **12b**) (1.05 eq.), corresponding benzaldehyde (1.0 eq.) and potassium persulfate (3.0 eq.) were suspended in DMSO/water a 1:1 (v/v) mixture (25 ml per 1g of compound **12b**) and reacted under microwave irradiation at 100 °C (5 min, 250 Watts). The reaction mixture was cooled down, diluted with ethyl acetate and quenched with water. Product was extracted thrice with ethyl acetate. The combined organic phases were washed with brine, dried over $MgSO_4$ and evaporated. Crude product was purified via silica gel column chromatography to give desirable A11 (or A12 derivative).

5-(2,4-Dimethylphenyl)-1-ethyl-1*H*-pyrazolo[4,3-*d*]pyrimidin-7(6*H*)-one A11-1

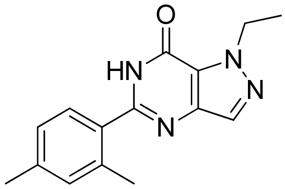

5-(2,4-Dimethylphenyl)-1-ethyl-1*H*-pyrazolo[4,3-*d*] pyrimidin-7(6*H*)-one A11-1 was synthesized from 4-amino-1-ethyl-1*H*-pyrazole-5-carboxamide (**12a**) (0.16 g, 0.86 mmol, 1.05 eq.) and 2,4-dimethylbenzaldehyde (0.11 g, 0.82 mmol, 1.0 eq.) according to general procedure. The crude product was purified *via* silica gel column chromatography eluted with hexane/EtOAc a ratio of 2:1 => 1:1 (v/v), followed by trituration with 2-PrOH/hexane a 1:2 (v/v) mixture to give 5-(2,4-dimethylphenyl)-1-ethyl-1H-pyrazolo[4,3-d] pyrimidin-7(6H)-one A11-1 (solid, 40 mg, 17%).

LC-MS: 99.1% (Rt = 2.55), ESI(+) m/z found: 269.29 $[M+H]^+$. Molecular Weight calc'd for $C_{15}H_{16}N_4O$ = 268.31.

^{1}H NMR (500 MHz, DMSO-d_6) δ ppm: 12.35 (s, 1H), 7.99 (s, 1H), 7.32 (d, *J* = 7.7 Hz, 1H), 7.15-7.08 (m, 2H), 4.59 (q, *J* = 7.2 Hz, 2H), 2.33 (s, 3H), 2.30 (s, 3H), 1.42 (t, *J* = 7.2 Hz, 3H).

^{13}C NMR (126 MHz, DMSO-d_6) δ ppm: 154.02, 152.62, 140.23, 139.17, 135.99, 132.60, 131.39, 131.07, 129.32, 126.17, 123.17, 46.13, 20.80, 19.54, 15.87.

1-Ethyl-5-phenyl-1*H*-pyrazolo[4,3-*d*]pyrimidin-7(6*H*)-one **A11-2**

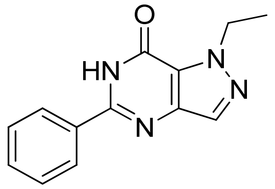

Compound was synthesized from 4-amino-1-ethyl-1*H*-pyrazole-5-carboxamide (**12a**) (0.153 g, 0.99 mmol, 1.05 eq.) and benzaldehyde (0.10 g, 0.94 mmol, 1.0 eq.) in the presence of potassium persulfate (0.764 g, 2.83 mmol, 3.0 eq.) according to general procedure. After evaporation crude product (136 mg) was tritueted with 2-PrOH/hexane (1:2) to give 1-ethyl-5-phenyl-1*H*-pyrazolo[4,3-*d*]pyrimidin-7(6*H*)-one (76 mg , 32%) **A11-2.**

LC-MS: 100% (Rt = 2.24), ESI(+) m/z found: 241.16 $[M+H]^+$. Molecular Weight calc'd for $C_{13}H_{12}N_4O$ = 240.26.

^{1}H NMR (500 MHz, DMSO-d_6) δ ppm: 12.49 (s, 1H), 8.08-8.02 (m, 3H), 7.57-7.48 (m, 3H), 4.60 (q, *J* = 7.2 Hz, 2H), 1.42 (t, *J* = 7.2 Hz, 3H). *Note: dimethyl sulfone presented on the spectrum 3.00 ppm*

^{13}C NMR (126 MHz, DMSO-d_6) δ ppm: 154.28, 151.09, 140.33, 132.86, 132.81, 130.81, 128.54(2C), 127.61(2C), 123.26, 46.15, 15.84.

1-Benzyl-5-phenyl-1*H*-pyrazolo[4,3-d]pyrimidin-7(6*H*)-one **A11-3**

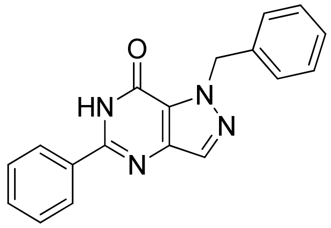

Compound was synthesized from 4-amino-1-benzyl-1*H*-pyrazole-5-carboxamide (**12a**) (0.139 g, 0.64 mmol, 1.05 eq.) and benzaldehyde (0.065 g, 0.61 mmol, 1.0 eq.) in the presence of potassium persulfate (0.52 g, 1.93 mmol, 3.0 eq.) according to general procedure. Crude product (188 mg) was purified *via* column chromatography eluted with $CHCl_3$:MeOH a ratio of 99:1 (v/v), followed by trituration with 2-PrOH/hexane a 1:2 (v/v) mixture to give 1-benzyl-5-phenyl-1*H*-pyrazolo[4,3-*d*]pyrimidin-7(6*H*)-one **A11-3** (72 mg, 37%).

LC-MS: 98.0% (Rt = 2.73), ESI(+) m/z found: 303.15 $[M+H]^+$. Molecular Weight calc'd for $C_{18}H_{14}N_4O$ = 302.33.

^{1}H NMR (500 MHz, DMSO-d$_6$) δ ppm: 12.56 (s, 1H), 8.11 (s, 1H), 8.08-8.05 (m, 2H), 7.57-7.49 (m, 3H), 7.36-7.31 (m, 2H), 7.30-7.26 (m, 3H), 5.79 (s, 2H). *Note: dimethyl sulfone presented on the spectrum 3.00 ppm.*

^{13}C NMR (126 MHz, DMSO-d$_6$) δ ppm: 154.38, 151.41, 140.51, 137.24, 133.77, 132.78, 130.86, 128.60(2C), 128.54(2C), 127.73, 127.67(2C), 127.58(2C), 123.73, 54.06.

2-Ethyl-5-phenyl-2*H*-pyrazolo[4,3-*d*]pyrimidin-7(6*H*)-one **A12-1**

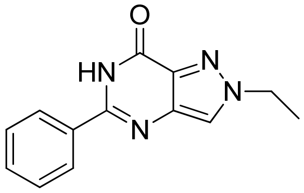

Compound was synthesized from 4-amino-1-ethyl-1*H*-pyrazole-3-carboxamide (**12b**) (0.214 g, 1.39 mmol, 1.05 eq.) and benzaldehyde (0.140 g, 1.32 mmol, 1.0 eq.) in the presence of potassium persulfate (1.123 g, 4.16 mmol, 3.0 eq.) according to general procedure. Crude product was purified *via* silica gel column chromatography eluted with DCM/MeOH a ratio of 99:1 (v/v), followed by trituration with 2-PrOH/hexane a 1:2 (v/v) mixture to give 2-ethyl-5-phenyl-2*H*-pyrazolo[4,3-*d*]pyrimidin-7(6*H*)-one **A12-1** (36 mg, 11%).

LC-MS: 100% (Rt = 1.86), ESI(+) m/z found: 241.23 [M+H]$^+$. Molecular Weight calc'd for $C_{13}H_{12}N_4O$ = 240.26.

^{1}H NMR (500 MHz, DMSO-d$_6$) δ ppm: 12.06 (s, 1H), 8.42 (s, 1H), 8.08-8.04 (m, 2H), 7.56-7.48 (m, 3H), 4.38 (q, *J* = 7.3 Hz, 2H), 1.49 (t, *J* = 7.3 Hz, 3H). *Note:* low intensity.

2-Benzyl-5-phenyl-2*H*-pyrazolo[4,3-*d*]pyrimidin-7(6*H*)-one **A12-2**

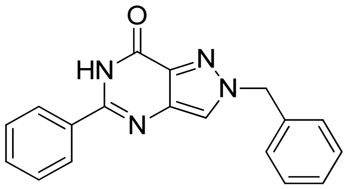

Compound was synthesized from 4-amino-1-benzyl-1*H*-pyrazole-3-carboxamide (**12b**) (0.214 g, 0.99 mmol, 1.05 eq.) and benzaldehyde (0.100 g, 0.94 mmol, 1.0 eq.) in the presence of potassium persulfate (0.802 g, 2.97 mmol, 3.0 eq.) according to general procedure. Red solid was filtered off from the reaction mixture and directly purified *via* silica column chromatography eluted with DCM/MeOH a ratio of 1:0 =>99:1=>49:1 (v/v)) to give 2-benzyl-5-phenyl-2*H*-pyrazolo[4,3-*d*]pyrimidin-7(6*H*)-one **A12-2** (38 mg, 13%).

LC-MS: 95.5% (Rt = 2.58), ESI(+) m/z found: 303.10 [M+H]$^+$. Molecular Weight calc'd for $C_{18}H_{14}N_4O$ = 302.33.

^{1}H NMR (500 MHz, DMSO-d$_6$) δ ppm: 12.10 (s, 1H), 8.55 (s, 1H), 8.09-8.00 (m, 2H), 7.58-7.44 (m, 3H), 7.42-7.26 (m, 5H), 5.58 (s, 2H). *Note: signal of dimethyl sulfone is present at 3.00 ppm.*

^{13}C NMR (126 MHz, DMSO-d_6) δ ppm: 157.29, 150.87, 138.00, 136.40, 135.16, 133.06, 130.74, 128.67(2C), 128.50(2C), 128.06, 127.95(2C), 127.50(2C), 125.74, 56.65. *Note: signal of dimethyl sulfone is present at 42.10 ppm.*

1.2.12. Chemotypes A13

General procedure for the synthesis of A13 derivatives

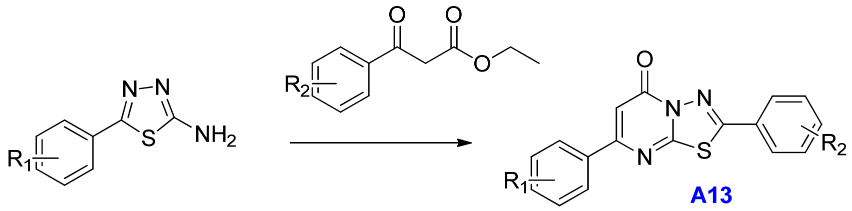

Corresponding 2-amino-5-phenylsubstituted-1,3,4-thiadiazole derivative (1.0 mmol, 1.0 eq.) was reacted with appropriate ethylbenzoyl acetate (1.3 mmol, 1.3 eq.) in the presence of polyphosphoric acid (≥83% phosphate as P_2O_5) (1.1 g). The reaction mixture was stirred in a sealed tube at 140 °C for 1 h, then cooled down, diluted with $CHCl_3$ and water. Product was extracted to $CHCl_3$ and next the organic phase was washed with brine, dried over $MgSO_4$ and evaporated. Crude product was pufiried *via* silica gel column chromatography eluted with $CHCl_3$/hexane a ratio of 1:1 (v/v) followed by trituration with EtOH/$CHCl_3$ a 1:2 (v/v) mixture to give desirable A13.

2,7-Diphenyl-5*H*-[1,3,4]thiadiazolo[3,2-*a*]pyrimidin-5-one A13-1

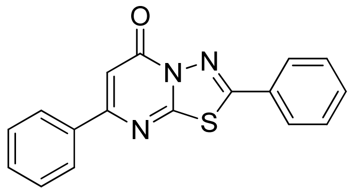

Compound was synthesized from commercially available 2-amino-5-phenyl-1,3,4-thiadiazole (0.18 g, 1.0 mmol, 1.0 eq.) and ethyl benzoylacetate (0.25 g, 1.3 mmol, 1.3 eq.) in the presence of polyphosphoric acid (≥83% phosphate as P_2O_5) (1.1 g) according to general procedure. Crude product was pufiried *via* silica gel column chromatography eluted with $CHCl_3$/hexane a ratio of 1:1 (v/v) and next *via* column chromatography on aluminum oxide (Al_2O_3) eluted with hexane/EtOAc a ratio of 2:1 (v/v), followed by trituration with EtOH/$CHCl_3$ a 1:2 (v/v) mixture. There was obtained: 47 mg of 2,7-diphenyl-5*H*-[1,3,4]thiadiazolo[3,2-*a*]pyrimidin-5-one A13-1 as an oily product.

LC-MS: 98.6% (Rt = 3.26), ESI(+) m/z found: 305.95 $[M+H]^+$. Molecular Weight calc'd for $C_{17}H_{11}N_3OS$ = 305.35.

^1H NMR (500 MHz, $CDCl_3$) δ ppm: 8.04-7.97 (m, 4H), 7.64-7.46 (m, 6H), 6.93 (s, 1H).

^{13}C NMR (126 MHz, $CDCl_3$) δ ppm: 161.45, 160.66, 159.12, 157.77, 135.84, 133.04, 131.07, 129.50(2C), 129.02(2C), 128.50, 127.84(2C), 127.36(2C), 104.56.

7-(2-Chlorophenyl)-2-phenyl-5*H*-[1,3,4]thiadiazolo[3,2-*a*]pyrimidin-5-one **A13-2**

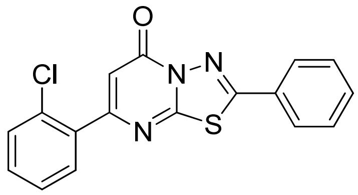

Compound was synthesized from 5-(2-chlorophenyl)-1,3,4-thiadiazol-2-amine (0.42 g, 1.0 mmol, 1.0 eq.) and ethyl (2-chlorobenzoyl)acetate (0.50 g, 1.3 mmol, 1.3 eq.) in the presence of polyphosphoric acid (≥83% phosphate as P_2O_5, 2.2 g) according to general procedure. Crude product was pufiried *via* silica gel column chromatography eluted with $CHCl_3$/hexane a ratio of 1:1 (v/v) and next *via* column chromatography on aluminum oxide (Al_2O_3) eluted with hexane/EtOAc a ratio of 2:1 (v/v), followed by slurring with EtOH. There was obtained: 126 mg of oily product **A13-2.**

LC-MS: 94.0% (Rt = 3.57), ESI(+) m/z found: 339.71 $[M+H]^+$. Molecular Weight calc'd for $C_{17}H_{10}ClN_3OS$ = 339.80.

^{1}H NMR (500 MHz, $CDCl_3$) δ ppm: 8.33 (ddd, *J* = 7.9, 1.7, 0.4 Hz, 1H), 8.04-7.97 (m, 2H), 7.59-7.42 (m, 6H), 6.93 (s, 1H).

^{13}C NMR (126 MHz, $CDCl_3$) δ ppm: 161.84, 160.90, 157.55, 155.94, 135.81, 133.27, 133.08, 131.33, 130.98, 130.89, 128.90(2C), 127.63, 127.30(2C), 127.24, 104.03.

2-(2-Chlorophenyl)-7-phenyl-5*H*-[1,3,4]thiadiazolo[3,2-*a*]pyrimidin-5-one **A13-3**

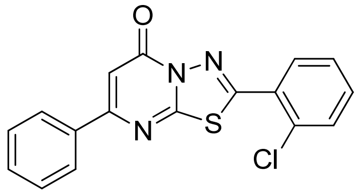

Compound was synthesized from 2-amino-5-phenyl-1,3,4-thiadiazole (0.27 g, 1.5 mmol, 1.0 eq.) and ethyl 3-(2-chlorophenyl)-3-oxopropanoate (0.34 g, 1.6 mmol, 1.06 eq.) in the presence of polyphosphoric acid (≥83% phosphate as P_2O_5) (1.6 g) according to general procedure. Crude product was pufiried *via* silica gel column chromatography eluted with $CHCl_3$/hexane a ratio of 1:1 (v/v) and next *via* column chromatography on aluminum oxide (Al_2O_3) eluted with hexane/EtOAc a ratio of 2:1 (v/v), followed by slurring with EtOH/$CHCl_3$ a 1:2 (v/v) mixture. There was obtained: 75 mg of oily product **A13-3.**

LC-MS: 97.6% (Rt = 3.29), ESI(+) m/z found: 341.94 $[M+H]^+$. Molecular Weight calc'd for $C_{17}H_{10}ClN_3OS$ = 339.80.

^{1}H NMR (500 MHz, $CDCl_3$) δ ppm: 8.02-7.97 (m, 2H), 7.65-7.46 (m, 5H), 7.42-7.35 (m, 2H), 6.84 (s, 1H).

^{13}C NMR (126 MHz, $CDCl_3$) δ ppm: 161.21, 160.02, 159.40, 157.03, 136.11, 133.05, 132.22, 130.82, 130.71, 130.59, 129.43(2C), 128.28, 127.79(2C), 127.05, 110.17.

1.2.13. Chemotypes A14

General procedure for the synthesis of A14 derivatives

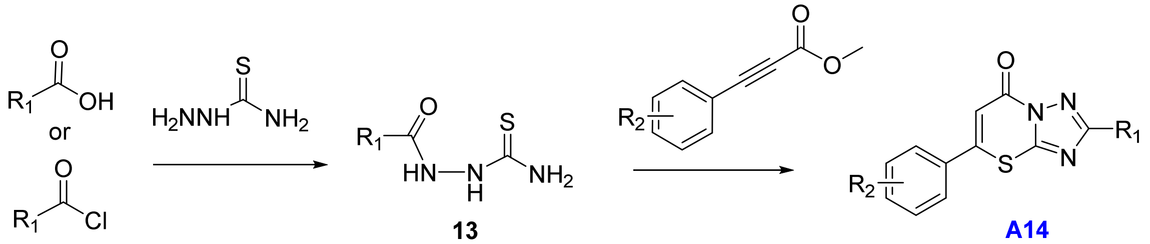

Procedures for synthesis of intermediate **13**

2-Propionylhydrazinecarbothioamide (**13a**)

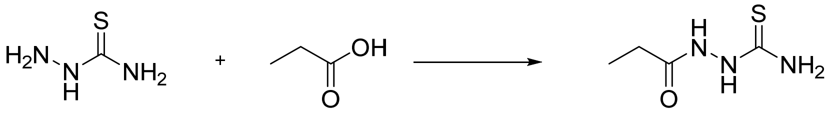

Compound **13a** was synthesized according to Faridoon *et.al Bioorg. Med. Chem. Lett.* 22 (2012) 380–386 [10], from hydrazinecarbothioamide (2.73 g, 30 mmol, 1.0 eq.) and propionic acid (13.86 g, 10 mL, 0.19 mol, 6.24 eq.). The reaction mixture was stirred in a sealed tube under reflux for 4 h, then cooled down to RT. Then the mixture was quenched with sat. $NaHCO_3$ and diluted with EtOAc. Product was extracted to EtOAc. The organic layer was washed with brine, dried over $MgSO_4$ and evaporated. There was obtained: 490 mg of semi-solid product (**13a**).

2-(2-Chlorobenzoyl)hydrazinecarbothioamide (**13b**)

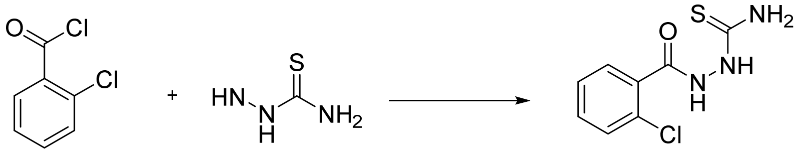

Compound **13b** was synthesized according to Faridoon *et.al Bioorg. Med. Chem. Lett.* 22 (2012) 380–386 [10]. 2-chlorobenzoyl chloride (1.72 g, 1.3 mL, 9.83 mmol, 1.0 eq.) was dissolved in THF, cooled down to 15 °C and next hydrazinecarbothioamide (1.82 g, 20 mmol, 2.03 eq.) was added in portions. The reaction mixture was stirred at RT overnight. Then the reaction mixture was quenched with sat. $NaHCO_3$, diluted with EtOAc. Product was extracted to EtOAc. The organic layer was washed with sat. $NaHCO_3$, brine and dried over $MgSO_4$ and evaporated to give: 1.6 g of crude product. The crude product was treated with 100 mL of EtOAc and undissolved solid was filtered off. The filtrate contained desired product was evaporated and crude residue was crystallized from EtOH to give 0.4 g of product (**13b**).

LC-MS : 97.1% (Rt = 0,79), ESI(+) m/z found: 230.03 $[M+H]^+$. Molecular Weight calc'd for $C_8H_8ClN_3OS$ = 229.69.

General procedure for synthesis of **A14**

Derivatives **A14** were synthesized from appropriate hydrazinecarbothioamide (**13**) (1.0 mmol, 1.0 eq.) and corresponding methyl phenylpropiolate (1.6 mmol, 1.06 eq.) in acetic acid (5 mL). The reaction mixture was stirred in a sealed tube under reflux for 18 h, then cooled down, diluted with $CHCl_3$ and water. Product was extracted to $CHCl_3$ and next the organic phase was washed with brine, dried over $MgSO_4$ and evaporated. Crude product was purified *via* silica gel column chromatography eluted with $CHCl_3$/hexane a ratio of 1:2 (v/v) and next *via* silica gel column chromatography eluted with $CHCl_3$/hexane a ratio of 1:1 (v/v) as an eluate, followed by trituration with 2-PrOH,to give desirable product **A14.**

2,5-Diphenyl-7*H*-[1,2,4]triazolo[5,1-*b*][1,3]thiazin-7-one **A14-1**

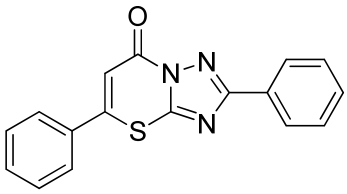

Compound was synthesized from 2-benzoylhydrazinecarbothioamide (0.20 g, 1.0 mmol, 1.0 eq.) and methyl 3-phenylpropiolate (0.16 g, 1.6 mmol, 1.06 eq.) in acetic acid (5 mL) according to general procedure. Crude product was purified *via* silica gel column chromatography eluted with $CHCl_3$/hexane a ratio of 1:2 (v/v) and next *via* silica gel column chromatography using $CHCl_3$/hexane a ratio of 1:1 (v/v), followed by trituration with 2-PrOH. There was obtained: 120 mg of beige solid product **A14-1**.

LC-MS: 98.4% (Rt = 3.42), ESI(+) m/z found: 306.08 $[M+H]^+$. Molecular Weight calc'd for $C_{17}H_{11}N_3OS$ = 305.35.

^{1}**H NMR** (500 MHz, $CDCl_3$) δ ppm: 8.33–8.28 (m, 2H), 7.69–7.64 (m, 2H), 7.62–7.53 (m, 3H), 7.52–7.46 (m, 3H), 7.04 (s, 1H).

^{13}**C NMR** (126 MHz, $CDCl_3$) δ ppm: 164.0, 155.8, 152.2, 150.8, 134.2, 132.1, 130.9, 129.8, 129.7, 128.8, 128.8(2C), 127.6(2C), 126.9(2C), 114.6.

2-Ethyl-5-phenyl-7*H*-[1,2,4]triazolo[5,1-*b*][1,3]thiazin-7-one **A14-2**

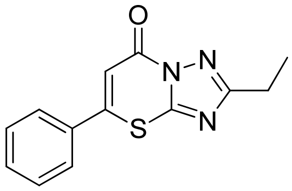

Compound was synthesized from 2-propionylhydrazinecarbothioamide (**13a**) (0.44 g, 3.0 mmol, 1.0 eq.) and methyl 3-phenylpropiolate (0.48 g, 3.0 mmol, 1.0 eq.) in acetic acid (15 mL) according to general procedure. Crude product was purified *via* silica gel column chromatography eluted with EtOAc/hexane a ratio of 1:3 (v/v), to give 140 mg of beige solid product **A14-2**.

LC-MS: 100% (Rt = 2.76), ESI(+) m/z found: 258.02 $[M+H]^+$. Molecular Weight calc'd for $C_{13}H_{11}N_3OS$ = 257.31.

^{1}H NMR (500 MHz, $CDCl_3$) δ ppm: 7.64–7.63 (m, 1H), 7.63–7.62 (m, 1H), 7.59–7.51 (m, 3H), 6.97 (s, 1H), 2.92 (q, J = 15.2, 7.6, 2H), 1.39 (t, J = 7.6, 3H).

^{13}C NMR (126 MHz, $CDCl_3$) δ ppm: 168.5, 155.9, 151.8, 151.0, 134.4, 132.2, 129.8(2C), 127.0(2C), 114.6, 22.2, 12.2.

2-(2-Chlorophenyl)-5-phenyl-7*H*-[1,2,4]triazolo[5,1-*b*][1,3]thiazin-7-one **A14-3**

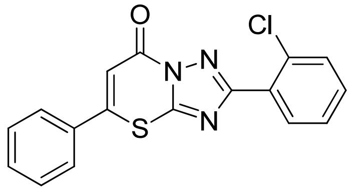

Compound was synthesized from 2-(2-chlorobenzoyl)hydrazinecarbothioamide (**13b**) (0.32 g, 1.4 mmol, 1.0 eq.) and methyl 3-phenylpropiolate (0.22 g, 1.4 mmol, 1.0 eq.) in acetic acid (7 mL) according to general procedure. Crude product was purified *via* silica gel column chromatography eluted with $CHCl_3$/hexane a ratio of 1:2 (v/v) and next using $CHCl_3$/hexane (gradient elution, 100% hexane => $CHCl_3$/hexane a ratio of 1:1 (v/v)), followed by trituration with hexane. There was obtained: 130 mg of beige solid of **A14-3** product.

LC-MS: 94.0% (Rt = 3.57), ESI(+) m/z found: 340.04 $[M+H]^+$. Molecular Weight calc'd for $C_{17}H_{10}ClN_3OS$ = 339.80.

^{1}H NMR (500 MHz, $CDCl_3$) δ ppm: 8.04–7.98 (m, 1H), 7.71–7.65 (m, 2H), 7.64–7.51 (m, 4H), 7.45–7.37 (m, 2H), 7.07 (s, 1H).

^{13}C NMR (126 MHz, $CDCl_3$) δ ppm: 162.84, 155.64, 151.69, 151.25, 134.23, 133.52, 132.18, 132.07, 131.41, 130.87, 129.80(2C), 128.18, 126.97(2C), 126.79, 114.57.

1.2.14. Chemotypes A15

General procedure for the synthesis of **A15** derivatives

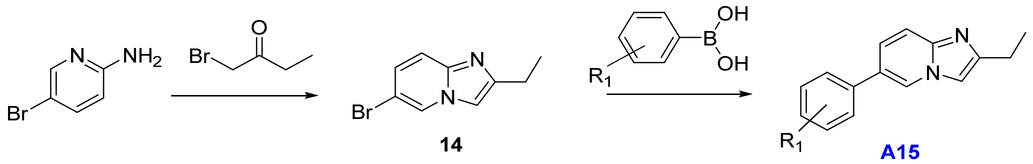

Procedure for the synthesis of 6-bromo-2-ethylimidazo[1,2-*a*]pyridine (**14**)

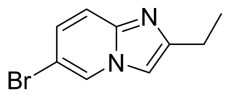

2-amino-5-bromopyridine (1.146 g, 6.62 mmol) was suspended in EtOH (16 mL) and treated with 1-bromo-2-butanone (1.0 g, 6.62 mmol). The reaction was run in a sealed tube at 85 °C overnight. Then the reaction mixture was cooled down and evaporated to dryness. Crude product was purified *via* column chromatography eluted with hexane/EtOAc a ratio of 1:0 => 1:1 => 0:1 (v/v) and then using 10% MeOH in EtOAc as an eluate, followed by trituration with hexane/EtOAc a 1:1 (v/v) mixture to give 6-bromo-2-ethylimidazo[1,2-*a*]pyridine **14** (1.48 g, quantitative).

LC-MS: 100% (Rt = 0.58), ESI(+) m/z found: 225.03 $[M+H]^+$. Molecular Weight calc'd for $C_9H_9BrN_2$ = 225.09.

Procedure for synthesis of A15 derivative

A mixture of 6-bromo-2-ethylimidazo[1,2-*a*] pyridine (**14**) (1.0 eq), corresponding arylboronic acid (1.2 eq), potassium carbonate (3.0 eq.) and 2N Na_2CO_3 aq solution (2.0 eq) in 1,4-dioxane (15 vol/1 g of I) was degassed with argon and Pd(dppf)Cl_2 complex in DCM (0.10 eq.) was added. Reaction was run in a sealed tube at 80 °C overnight. Then the mixture was cooled down to RT, quenched with water and extracted thrice with chloroform. The combined organic extracts were washed with brine, dried over $MgSO_4$ and evaporated on rotovap. Crude product was purified via silica gel column chromatography, followed by trituration with appropriate solvent to give desirable **A15** derivative.

6-(2,4-Dimethylphenyl)-2-ethylimidazo[1,2-*a*]pyridine **A15-1**

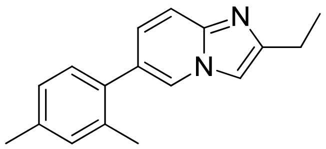

Compound **A15-1** was prepared from 6-bromo-2-ethylimidazo[1,2-*a*] pyridine (**14**) (200 mg, 0.89 mmol) and 2,4-dimethylbenzeneboronic acid (170 mg, 0.89 mmol) according to general procedure. The crude product was purified *via* silica gel column chromatography eluted with $CHCl_3$/MeOH a ratio of 99:1=>19:1 (v/v), followed by trituration with petroleum ether : EtOAc a 8:1 (v/v) mixture to yield 6-(2,4-dimethylphenyl)-2-ethylimidazo[1,2-a] pyridine **A15-1** as a solid product (43 mg, 19.4%).

LC-MS: 95.3% (Rt = 2.16), ESI(+) *m/z* found: 251.23 $[M+H]^+$. Molecular Weight calc'd for $C_{17}H_{18}N_2$ = 250.34.

^{1}**H NMR (**500 MHz, DMSO-d$_6$) δ ppm: 8.40 (s, 1H), 7.67 (s, 1H), 7.46 (d, *J* = 9.2 Hz, 1H), 7.20–7.09 (m, 3H), 7.07 (d, *J* = 7.6 Hz, 1H), 2.71 (q, *J* = 7.5 Hz, 2H), 2.31 (s, 3H), 2.22 (s, 3H), 1.26 (t, *J* = 7.6 Hz, 3H).

^{13}**C NMR** (126 MHz, DMSO-d$_6$) δ ppm: 148.82, 143.12, 136.96, 135.23, 134.55, 131.08, 129.72, 126.63, 126.32, 125.06, 125.00, 115.20, 109.11, 21.77, 20.63, 20.02, 13.57.

2-Ethyl-6-phenylimidazo[1,2-*a*]pyridine **A15-2**

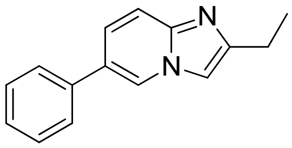

2-Ethyl-6-phenylimidazo[1,2-*a*] pyridine **A15-2** was prepared from 6-bromo-2-ethylimidazo[1,2-*a*] pyridine (**14**) (1 g, 4.44 mmol) and benzene boronic acid (700 mg, 5.78 mmol) according to general procedure. After extraction and evaporation, the crude product was purified *via* silica gel column chromatography eluted with hexane/EtOAc a ratio of 3:1=>1:1 (v/v) to yield 2-ethyl-6-phenylimidazo[1,2-*a*] pyridine **A15-2** as a solid product (43 mg, 19.4%).

LC-MS: 99.0% (Rt =1.57), ESI(+) *m/z* found: 223.10 $[M+H]^+$. Molecular Weight calc'd for $C_{15}H_{14}N_2$ = 222.29.

^{1}H NMR (500 MHz, DMSO-d_6) δ ppm: 9.32 (dd, *J* = 1.8, 0.9 Hz, 1H), 8.23 (dd, *J* = 9.4, 1.8 Hz, 1H), 8.15 (d, *J* = 0.7 Hz, 1H), 7.97 (dt, *J* = 9.4, 0.8 Hz, 1H), 7.78–7.75 (m, 2H), 7.57–7.52 (m, 2H), 7.50–7.46 (m, 1H), 2.91–2.83 (m, 2H), 1.34 (t, *J* = 7.6 Hz, 3H).

^{13}C NMR (126 MHz, DMSO-d_6) δ ppm: 139.32, 138.35, 134.75, 132.12, 129.33(2C), 129.26, 128.82, 126.92(2C), 125.92, 111.61, 111.56, 18.20, 12.55.

2-Ethyl-6-(3-methoxyphenyl)imidazo[1,2-*a*]pyridine **A15-3**

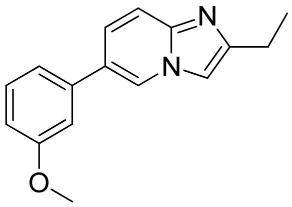

2-Ethyl-6-(3-methoxyphenyl) imidazo[1,2-*a*] pyridine **A15-3** was prepared from 6-bromo-2-ethylimidazo[1,2-*a*] pyridine (**14**) (1 g, 4.44 mmol) and 3-methoxyphenyl boronic acid (880 mg, 5.78 mmol) according to method general procedure. The crude product was purified *via* silica gel column chromatography eluted with $CHCl_3$/MeOH a ratio of 99:1=>19:1 (v/v), to give 747 mg of oily 2-ethyl-6-(3-methoxyphenyl) imidazo[1,2-*a*] pyridine, which was further re-purified using hexane/EtOAc a ratio of 3:1=>1:2 (v/v) to yield **A15-3** as a solid product (443 mg, 39.5%).

LC-MS: 98.5% (Rt =1.82), ESI(+) *m/z* found: 253.16 $[M+H]^+$. Molecular Weight calc'd for $C_{16}H_{16}N_2O$ = 252.31.

^{1}H NMR (500 MHz, DMSO-d_6) δ ppm: 9.34 (s, 1H), 8.24 (dd, *J* = 9.4, 1.8 Hz, 1H), 8.13 (s, 1H), 7.94 (d, *J* = 9.3 Hz, 1H), 7.46–7.42 (m, 1H), 7.33–7.30 (m, 2H), 7.03 (ddd, *J* = 8.3, 2.4, 1.0 Hz, 1H), 3.84 (s, 3H), 2.88 (qd, *J* = 7.5, 0.7 Hz, 2H), 1.33 (t, *J* = 7.6 Hz, 3H).

^{13}C NMR (126 MHz, DMSO-d_6) δ ppm: 159.93, 139.38, 138.41, 136.12, 132.12, 130.45, 129.05, 126.07, 119.08, 114.36, 112.44, 111.51, 111.49, 55.36, 18.21, 12.54.

2-Ethyl-6-(4-methoxyphenyl)imidazo[1,2-*a*]pyridine **A15-4**

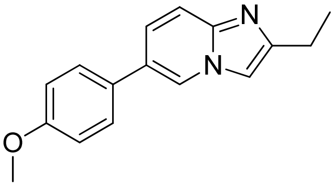

2-Ethyl-6-(4-methoxyphenyl)imidazo[1,2-*a*]pyridine was prepared from 6-bromo-2-ethylimidazo[1,2-*a*]pyridine (**14**) (1.10 g, 4.89 mmol) and 4-methoxyphenyl boronic acid (970 mg, 6.35 mmol) according to general procedure. After extraction crude product was purified by silica gel column chromatography eluted with hexane/EtOAc a ratio of 3:1 => 1:2 (v/v), followed by trituration with acetone to yield 2-ethyl-6-(4-methoxyphenyl)imidazo[1,2-*a*]pyridine **A15-4** as a solid product (474 mg, 38.5%).

LC-MS: 96.4% (Rt =1.66), ESI(+) *m/z* found: 253.16 $[M+H]^+$. Molecular Weight calc'd for $C_{16}H_{16}N_2O$ = 252.31.

^{1}H NMR (500 MHz, DMSO-d_6) δ ppm: 9.23 (s, 1H), 8.20 (dd, *J* = 9.4, 1.7 Hz, 1H), 8.10 (s, 1H), 7.93 (d, *J* = 9.3 Hz, 1H), 7.73–7.68 (m, 2H), 7.12–7.08 (m, 2H), 3.82 (s, 3H), 2.88 (q, *J* = 7.5 Hz, 2H), 1.33 (t, *J* = 7.6 Hz, 3H).

^{13}C NMR (126 MHz, DMSO-d_6) δ ppm: 159.85, 139.20, 138.06, 131.98, 129.07, 128.16(2C), 126.88, 125.00, 114.77 (2C), 111.47, 111.44, 55.35, 18.18, 12.54.

2-Ethyl-6-(pyridin-4-yl)imidazo[1,2-*a*]pyridine **A15-5**

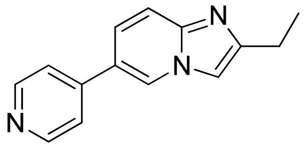

2-ethyl-6-(pyridin-4-yl)imidazo[1,2-*a*]pyridine was prepared from 6-bromo-2-ethylimidazo[1,2-*a*]pyridine (**14**) (1 g, 4.44 mmol) and pyridine-4-boronic acid (710 mg, 5.78 mmol) according to general procedure. The crude product was purified *via* silica gel column chromatography eluted with hexane/EtOAc a ratio of 3:1=>1:2 (v/v), to yield 2-ethyl-6-(pyridin-4-yl)imidazo[1,2-*a*]pyridine **A15-5** (492 mg, 49.8%).

LC-MS: 100% (Rt = 0.42), ESI(+) *m/z* found: 224.17 $[M+H]^+$. Molecular Weight calc'd for $C_{14}H_{13}N_3$ = 223.27.

^{1}H NMR (500 MHz, DMSO-d_6) δ ppm: 9.61 (s, 1H), 8.80 (d, *J* = 4.8 Hz, 2H), 8.33 (dd, *J* = 9.4, 1.2 Hz, 1H), 8.17 (s, 1H), 8.01 (d, *J* = 9.4 Hz, 1H), 7.97 (d, *J* = 5.2 Hz, 2H), 2.89 (q, *J* = 7.5 Hz, 2H), 1.33 (t, *J* = 7.6 Hz, 3H).

^{13}C NMR (126 MHz, DMSO-d_6) δ ppm: 148.87(2C), 144.01, 140.29, 139.20, 130.94, 127.65, 125.66, 121.84(2C), 112.20, 111.80, 18.39, 12.58.

1.2.15. Chemotypes A16

General procedure for the synthesis of **A16** derivatives

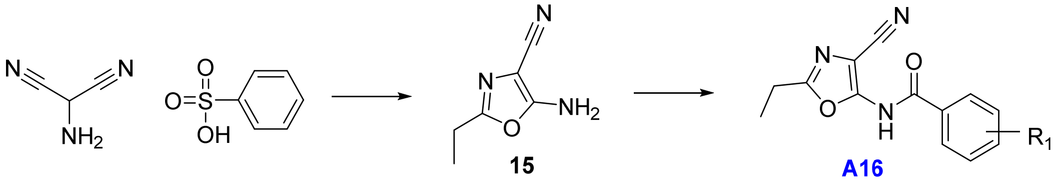

Procedure used for the synthesis of 5-amino-2-ethyloxazole-4-carbonitrile **15**

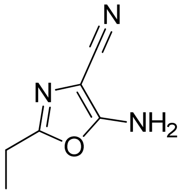

2-Aminomalonitrile toluene-4-sulphonate (1.0 g, 3.95 mmol) was dissolved in 1-methyl-2-pyrrolidione (NMP, 12 mL) at RT under argon atmosphere and treated with propionyl chloride (0.4 g, 4.34 mmol). The reaction mixture was stirred at RT/overnight. Then the mixture was diluted with ethyl acetate: diethyl ether a 1:1 (v/v) mixture (20 mL) and quenched with water (20 mL). The layers were separated

and product was extracted with EtOAc/diethyl ether a 1:1 (v/v) mixture three times. The combined organic layers were washed two times with saturated $NaHCO_3$ solution and then two times with water, dried over Na_2SO_4 and evaporated to give 613 mg of yellow solid product. The crude product was purified by trituration with hexane and then with hexane/EtOAc a ratio of 9:1, to give light yellow solid 5-amino-2-ethyloxazole-4-carbonitrile (**15**) (379 mg, 70.0%)

LC-MS: 97.4% (Rt = 0.94), ESI(+) *m/z* found: 138.06 $[M+H]^+$. Molecular Weight calc'd for $C_6H_7N_3O$ = 137.14.

Procedure for synthesis of **A16** derivative

5-amino-2-ethyloxazole-4-carbonitrile (**15**) (0.43 mmol, 1.0 eq) was suspended in DCM (30 mL per 1g of compound **15**), cooled down to 0 °C and treated with trimethylamine (0.66 mmol, 1.5 eq), next appropriate acid chloride (0.44 mol, 1.02 eq) was slowly dropped. The cooling bath was removed and next reaction was carried out at RT/overnight. The reaction mixture was diluted with $CHCl_3$ and quenched with water. The layers were separated and product was extracted with $CHCl_3$. The combined organic layer was dried over $MgSO_4$ and evaporated. The crude product was purified via silica gel column chromatography eluted with $CHCl_3$/MeOH a ratio of 99:1=>9:1 (v/v) to give **A16.**

N-(4-cyano-2-ethyloxazol-5-yl)-2,4-dimethylbenzamide **A16-1**

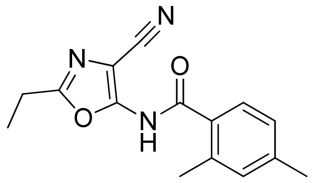

Compound **A16-1** was synthesized from 5-amino-2-ethyloxazole-4-carbonitrile (**15**) (60 mg, 0.43 mmol) and 2,4-dimethylbenzoyl chloride (74 mg, 0.44 mol) according to general procedure. The crude product was purified *via* silica gel column chromatography using $CHCl_3$/MeOH a ratio of 99:1=>9:1 (v/v) to give N-(4-cyano-2-ethyloxazol-5-yl)-2,4-dimethylbenzamide **A16-1** (26 mg, 22.0%).

LC-MS: 95.1% (Rt = 2.79), ESI(+) *m/z* found: 270.09 $[M+H]^+$. Molecular Weight calc'd for $C_{15}H_{15}N_3O_2$ = 269.30.

^{1}H NMR (500 MHz, DMSO-d$_6$) δ ppm: 11.93 (s, 1H), 7.48 (d, *J* = 7.8 Hz, 1H), 7.18–7.10 (m, 2H), 2.76 (q, *J* = 7.5 Hz, 2H), 2.39 (s, 3H), 2.33 (s, 3H), 1.26–1.20 (m, 3H*). Traces of impurities visible in the spectrum.*

^{13}C NMR (126 MHz, DMSO-d$_6$) δ ppm: 166.27, 159.76, 150.54, 141.06, 136.87, 131.69, 130.38, 128.20, 126.21, 113.34, 98.32, 20.87, 20.58, 19.61, 10.31.

N-(4-Cyano-2-ethyloxazol-5-yl)benzamide **A16-2**

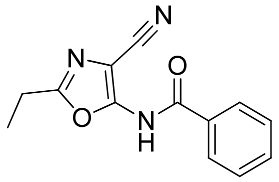

Compound **A16-2** was prepared from 5-amino-2-ethyloxazole-4-carbonitrile (**15**) (90 mg, 0.66 mmol) and benzoyl chloride (97 mg, 0.69 mol) according to general procedure. The crude product was purified

via silica gel column chromatography eluted with $CHCl_3$/MeOH a ratio of 99:1=>9:1 (v/v) to give *N*-(4-cyano-2-ethyloxazol-5-yl) benzamide **A16-2** (40 mg, 25.3%).

LC-MS: 97.9% (Rt = 2.32), ESI(+) *m/z* found: 242.16 $[M+H]^+$. Molecular Weight calc'd for $C_{13}H_{11}N_3O_2$ = 241.25.

^{1}H NMR (500 MHz, DMSO-d_6) δ ppm: 12.07 (s, 1H), 8.02 (ddd, *J* = 6.8, 3.2, 1.6 Hz, 2H), 7.70–7.64 (m, 1H), 7.59–7.54 (m, 2H), 2.78 (q, *J* = 7.5 Hz, 2H), 1.25 (t, *J* = 7.5 Hz, 3H).

^{13}C NMR (126 MHz, DMSO-d_6) δ ppm: 164.25, 159.87, 150.59, 133.03, 131.61, 128.70 (2C), 128.23(2C), 113.38, 98.70, 20.60, 10.30.

1.2.16. Chemotypes A17

General procedure for synthesis of **A17** derivative

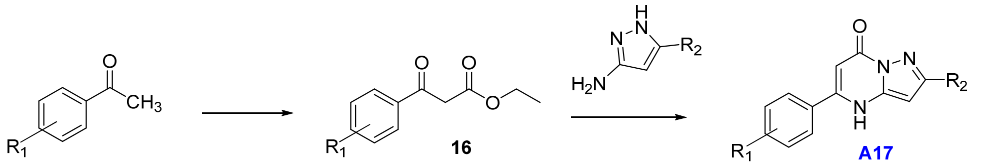

Procedure for the synthesis of ß-ketoesters **16**

Sodium hydride (60% suspension in paraffin oil, 3.0 eq.) was suspended in dry toluene (15 vol per 1 g of acetophenone) under argon atmosphere, cooled down to 0 °C with ice-bath and then diethyl carbonate (2.0 eq.) was slowly dropped. The suspension was stirred at 0 °C for 30 min. Next corresponding acetophenone derivative (1.0 eq.) was added and the cooling bath was removed. The reaction mixture was warmed to RT spontaneously and next refluxed for 1.5–2 h. After the reaction was complete, the mixture was cooled down to RT and poured into the ice. The product was extracted thrice with ethyl acetate, and the combined organic extracts were washed with brine, dried over Na_2SO_4 and evaporated. Crude product was absorbed on SiO_2 and purified via column chromatography eluted with $CHCl_3$/hexane (1:4 => 1:1) to give desirable ß-ketoester (**16**).

Procedure for the synthesis of **A17** derivatives

Compounds **A17** were synthesized according to method described in *J. Med. Chem.* 1981,24, 610-613 [11]. A mixture of corresponding 3-amino-1*H*-pyrazole (1.0 eq.) and ß-ketoester (**16**, 1.10–1.6 eq.) in acetic acid (15 vol per 5 g of amine) was heated in a sealed tube at temperature 90–130 °C for 6–12 h (*Note: at the beginning the reaction was run at 130 °C for 6–8 h, but if the reaction time was prolonged and stirring was continued during night the temperature was reduced to 90 °C*). After completing of the reaction, the mixture was cooled down to RT (*Note: in some experiments product precipitated directly from the RM after cooling*) and evaporated. The residue was diluted with chloroform and quenched with saturated sodium bicarbonate; product was extracted thrice with chloroform. The combined organic phases were washed with sat. $NaHCO_3$, brine, dried over $MgSO_4$ and evaporated. The crude product was purified via silica gel column chromatography preferably using $CHCl_3$/MeOH (99:1=>19:1) and then triturated with appropriate solvent (such as: Et_2O or Et_2O/EtOAc or 2-PrOH/hexane mixture) to give desired product **A17**.

Ethyl 3-(2,4-dimethylphenyl)-3-oxopropanoate (**16a**)

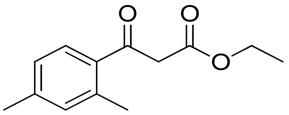

Compound **16a** was synthesized from 2,4-dimethylacetophenone (2.0 g, 13.49 mmol, 1.0eq), diethyl carbonate (3.19 g, 27.0 mmol, 2.0 eq) and sodium hydride (60% suspension in paraffin oil, 1.62 g, 40.49 mmol, 3.0 eq) in dry toluene (30 mL) according to general procedure. There was obtained: 1.61 g of oily product, 54.2% yield.

^{1}**H NMR** (400 MHz, $CDCl_3$) δ ppm: 7.54 (s, 2H), 7.22 (s, 1H), 4.21 (q, *J*=6.8 Hz, 2H), 3.96 (s, 2H), 2.37 (s, 6H), 1.26 (t, *J*=6.8 Hz, 3H).

Ethyl 3-(4-methoxyphenyl)-3-oxopropanoate (**16b**)

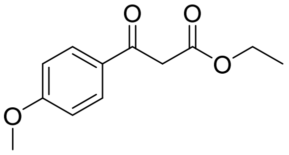

Compound **16b** was synthesized from 4-methoxyacetophenone (2.0 g, 13.32 mmol, 1.0eq), diethyl carbonate (3.15 g, 26.64 mmol, 2.0 eq) and sodium hydride (60% suspension in paraffin oil, 1.60 g, 39.95 mmol, 3.0 eq) in dry toluene (30 mL) according to general procedure. There was obtained: 1.98 g of oily product, 66.9% yield.

^{1}**H NMR** (400 MHz, $CDCl_3$) δ ppm: 7.95-7.92 (m, 2H), 6.97-6.94 (m,2H), 4.21 (q, *J* = 7.2 Hz, 2H), 3.95 (s, 2H), 3.88 (s, 3H), 1.26 (t, *J* = 7.2 Hz, 3H).

5-(2,4-Dimethylphenyl)-2-ethylpyrazolo[1,5-*a*] pyrimidin-7(4*H*)-one **A17-1**

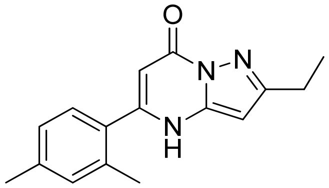

Compound **A17-1** was synthesized from 3-amino-5-ethyl-1*H*-pyrazole (0.20 g, 1.80 mmol) and ethyl (2,4-dimethylbenzolyl) acetate (**16a**) (0.44 g, 1.98 mmol) according to general procedure. The crude product after evaporation was purified *via* silica gel column chromatography eluted with $CHCl_3$/MeOH (100% $CHCl_3$ => a ratio of 19:1 (v/v), $CHCl_3$/MeOH). There was obtained: 102 mg of oily product which was precipitated from Et_2O/EtOAc a 2:1 (v/v) mixture. There was obtained: 64 mg of white solid product **A17-1** (yield: 13%).

LC-MS: 96.2% (Rt = 2.45), ESI(+) m/z found: 268.14 $[M+H]^+$. Molecular Weight calc'd for $C_{16}H_{17}N_3O$ = 267.33.

^{1}**H NMR** (500 MHz, DMSO-d_6) δ ppm: 12.30 (s, 1H), 7.31 (d, *J* = 7.7 Hz, 1H), 7.23–7.11 (m, 2H), 5.98 (s, 1H), 5.57 (s, 1H), 2.67 (q, *J* = 7.6 Hz, 2H), 2.34 (s, 3H), 2.29 (s, 3H), 1.23 (t, *J* = 7.6 Hz, 3H).

^{13}**C NMR** (126 MHz, DMSO-d$_6$) δ ppm: 157.62, 156.02, 150.17, 142.06, 139.65, 135.44, 131.19, 130.59, 128.90, 126.57, 96.06, 87.37, 21.65, 20.75, 19.33, 13.36.

2-Ethyl-5-phenylpyrazolo[1,5-*a*]pyrimidin-7(4*H*)-one **A17-2**

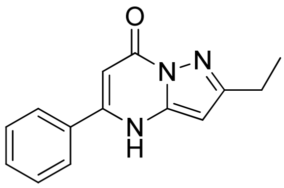

Prepared from 3-amino-5-ethyl-1*H*-pyrazole (0.20 g, 1.80 mmol) and commercially available ethyl benzoylacetate (0.38 g, 1.98 mmol) according to general procedure. Product was filtered directly from RM to give 2-ethyl-5-phenylpyrazolo[1,5-*a*]pyrimidin-7(4*H*)-one **A17-2** as a white solid (42 mg yield: 10%).

LC-MS: 100% (Rt = 1.98), ESI(+) m/z found: 240.16 [M+H]$^+$. Molecular Weight calc'd for $C_{14}H_{13}N_3O$ = 239.27.

^{1}**H NMR** (500 MHz, DMSO-d$_6$) δ ppm: 12.36 (s, 1H), 7.85–7.79 (m, 2H), 7.61–7.53 (m, 3H), 6.06 (s, 1H), 5.99 (s, 1H), 2.68 (q, *J* = 7.6 Hz, 2H), 1.24 (t, *J* = 7.6 Hz, 3H).

^{13}**C NMR** (126 MHz, DMSO-d$_6$) δ 157.75, 156.21, 149.24, 142.25, 132.37, 130.99, 129.05 (2C), 127.17(2C), 93.65, 87.87, 21.67, 13.34.

5-(2-Chlorophenyl)-2-ethylpyrazolo[1,5-*a*]pyrimidin-7(4*H*)-one **A17-3**

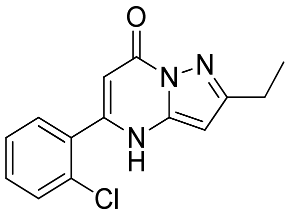

Compound **A17-3** was synthesized from 3-amino-5-ethyl-1*H*-pyrazole (0.20 g, 1.80 mmol) and commercially available ethyl (2-chlorobenzoyl)acetate (0.45 g, 1.98 mmol, 1.1 eq.) according to general procedure. The crude product after evaporation was purified *via* silica gel column chromatography eluting with $CHCl_3$/MeOH (a ratio of 19:1=>9:1 (v/v)), followed by trituration with 2-PrOH/hexane a 1:3 (v/v) mixture to give 5-(2-chlorophenyl)-2-ethylpyrazolo[1,5-*a*] pyrimidin-7(4*H*)-one **A17-3** as a solid (134 mg, 27.2%).

LC-MS: 96.6% (Rt = 2.16), ESI(+) m/z found: 274.09 [M+H]$^+$. Molecular Weight calc'd for $C_{14}H_{12}ClN_3O$ = 273.72.

^{1}**H NMR** (500 MHz, DMSO-d$_6$) δ ppm: 12.58 (s, 1H), 7.65 (ddd, *J* = 9.4, 7.8, 1.3 Hz, 2H), 7.61–7.56 (m, 1H), 7.52 (td, *J* = 7.5, 1.3 Hz, 1H), 6.03 (s, 1H), 5.69 (s, 1H), 2.68 (q, *J* = 7.6 Hz, 2H), 1.24 (t, *J* = 7.6 Hz, 3H).

^{13}**C NMR** (126 MHz, DMSO-d$_6$) δ ppm: 157.94, 155.88, 147.55, 141.94, 132.46, 131.91, 131.52, 131.10, 129.90, 127.57, 96.69, 87.62, 21.66, 13.36.

2-Ethyl-5-(4-methoxyphenyl)pyrazolo[1,5-*a*]pyrimidin-7(4*H*)-one **A17-4**

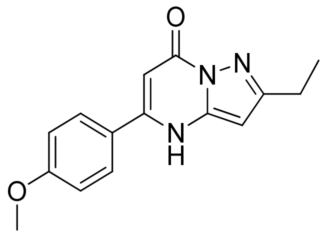

Compound was synthesized from 3-amino-5-ethyl-1*H*-pyrazole (0.20 g, 1.80 mmol) and commercially available ethyl (4-methoxybenzoyl)acetate (**16b**) (0.64 g, 2.88 mmol) according to general procedure. The beige solid product was filtered off from the reaction mixture and next triturating with diethyl ether to give 2-ethyl-5-(4-methoxyphenyl)pyrazolo[1,5-*a*]pyrimidin-7(4*H*)-one **A17-4** (116 mg, 24%).

LC-MS: 97.8% (Rt = 2.11), ESI(+) m/z found: 270.12 $[M+H]^+$. Molecular Weight calc'd for $C_{15}H_{15}N_3O_2$ = 269.30.

^{1}H NMR (500 MHz, DMSO-d_6) δ ppm: 12.21 (s, 1H), 7.81–7.76 (m, 2H), 7.14–7.08 (m, 2H), 6.03 (s, 1H), 5.94 (s, 1H), 3.84 (s, 3H), 2.67 (q, *J* = 7.6 Hz, 2H), 1.23 (t, *J* = 7.6 Hz, 3H).

^{13}C NMR (126 MHz, DMSO-d_6) δ ppm: 161.44, 157.58, 156.27, 148.90, 142.23, 128.68 (2C), 124.31, 114.44(2C), 92.56, 87.72, 55.48, 21.67, 13.35.

5-(2,4-Dimethylphenyl)-2-phenylpyrazolo[1,5-*a*]pyrimidin-7(4*H*)-one **A17-5**

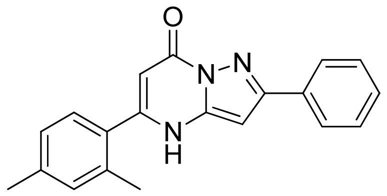

Compound was synthesized from 3-amino-5-phenylpyrazole (0.20 g, 1.26 mmol) and ethyl (2,4-dimethylbenzoyl)acetate (**16a**, 0.30 g, 1.38 mmol, 1.1 eq.) according to general procedure. The white solid product was filtered off directly from the reaction mixture to give 5-(2,4-dimethylphenyl)-2-phenylpyrazolo[1,5-*a*]pyrimidin-7(4*H*)-one **A17-5** (60 mg, 15%).

LC-MS: 98.2% (Rt = 2.81), ESI(+) m/z found: 316.21 $[M+H]^+$. Molecular Weight calc'd for $C_{20}H_{17}N_3O$ = 315.37.

^{1}H NMR (500 MHz, DMSO-d_6) δ ppm: 12.54 (s, 1H), 8.01–7.98 (m, 2H), 7.52–7.45 (m, 2H), 7.45–7.39 (m, 1H), 7.36 (d, *J* = 7.7 Hz, 1H), 7.23–7.20 (m, *J* = 1.0 Hz, 1H), 7.19–7.14 (m, 1H), 6.59 (s, 1H), 5.68 (s, 1H), 2.35 (s, 3H), 2.32 (s, 3H).

^{13}C NMR (126 MHz, DMSO-d_6) δ ppm: 156.07, 153.21, 150.67, 142.87, 139.76, 135.52, 132.41, 131.24, 130.51, 128.98, 128.94, 128.77(2C), 126.60, 126.20(2C), 96.52, 86.20, 20.79, 19.39.

2,5-Diphenylpyrazolo[1,5-*a*]pyrimidin-7(4*H*)-one **A17-6**

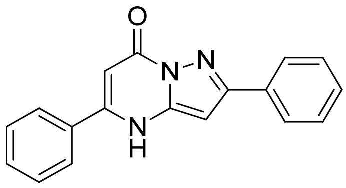

Compound was synthesized from 3-amino-5-phenylpyrazole (0.20 g, 1.26 mmol) and commercially available ethyl benzoylacetate (0.27 g, 1.38 mmol, 1.1 eq.) according to general procedure. The white solid product was filtered off directly from the reaction mixture to give 2,5-diphenylpyrazolo[1,5-*a*]pyrimidin-7(4*H*)-one **A17-6** (223 mg, 61%).

LC-MS: 100% (Rt = 2.45), ESI(+) m/z found: 288.15 $[M+H]^+$. Molecular Weight calc'd for $C_{18}H_{13}N_3O$ = 287.32.

^{1}H NMR (500 MHz, DMSO-d_6) δ ppm: δ 12.60 (s, 1H), 8.03–7.99 (m, 2H), 7.89–7.83 (m, 2H), 7.63–7.56 (m, 3H), 7.51–7.46 (m, 2H), 7.45–7.40 (m, 1H), 6.68 (s, 1H), 6.10 (s, 1H).

^{13}C NMR (126 MHz, DMSO-d_6) δ ppm: 156.26, 153.29, 149.72, 143.08, 132.38, 132.27, 131.11, 129.07 (2C), 128.96, 128.77(2C), 127.28(2C), 126.20(2C), 94.08, 86.63.

5-(2-Chlorophenyl)-2-phenylpyrazolo[1,5-*a*]pyrimidin-7(4*H*)-one **A17-7**

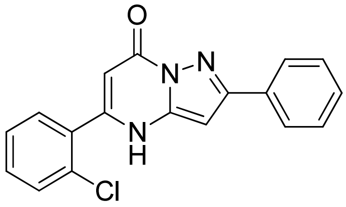

Compound was synthesized from 3-amino-5-phenylpyrazole (0.20 g, 1.26 mmol) and commercially available ethyl (2-chlorobenzoyl)acetate (0.31 g, 1.38 mmol, 1.1 eq.) according to general procedure. The white solid product was filtered off directly from the reaction mixture to give 5-(2-chlorophenyl)-2-phenylpyrazolo[1,5-*a*]pyrimidin-7(4*H*)-one **A17-7** (142 mg, 35%).

LC-MS: 99.3% (Rt = 2.49), ESI(+) m/z found: 322.14 $[M+H]^+$. Molecular Weight calc'd for $C_{18}H_{12}ClN_3O$ = 321.76.

^{1}H NMR (500 MHz, DMSO-d_6) δ ppm: 12.83 (s, 1H), 8.04–8.00 (m, 2H), 7.71–7.66 (m, 2H), 7.63–7.58 (m, 1H), 7.54 (td, *J* = 7.5, 1.3 Hz, 1H), 7.51–7.46 (m, 2H), 7.45–7.40 (m, 1H), 6.67 (s, 1H), 5.79 (s, 1H).

^{13}C NMR (126 MHz, DMSO-d_6) δ ppm: 155.93, 153.48, 148.04, 142.73, 132.37, 132.29, 132.00, 131.55, 131.16, 129.92, 129.04, 128.78(2C), 127.60, 126.26(2), 97.14, 86.45.

5-(4-Methoxyphenyl)-2-phenylpyrazolo[1,5-*a*]pyrimidin-7(4*H*)-one **A17-8**

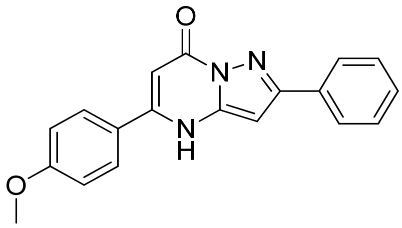

Compound was synthesized from 3-amino-5-phenylpyrazole (0.20 g, 1.26 mmol) and commercially available ethyl(4-methoxybenzoyl)acetate (**16b**) (0.31 g, 1.38 mmol, 1.1 eq.) according to general procedure. The solid product was filtered off directly from the reaction mixture and next triturating with diethyl ether to give 5-(4-methoxyphenyl)-2-phenylpyrazolo[1,5-*a*]pyrimidin-7(4*H*)-one **A17-8** (84 mg, 21%) poor soluble product.

LC-MS: 95.9% (Rt = 2.53), ESI(+) m/z found: 317.74 $[M+H]^+$. Molecular Weight calc'd for $C_{19}H_{15}N_3O_2$ = 317.34.

^{1}H NMR (500 MHz, DMSO-d_6) δ ppm: 12.46 (s, 1H), 8.01–7.98 (m, 2H), 7.87–7.82 (m, 2H), 7.52–7.46 (m, 2H), 7.45–7.39 (m, 1H), 7.17–7.09 (m, 2H), 6.63 (s, 1H), 6.05 (s, 1H), 3.86 (s, 3H). Low intensity, residual solvents presented.

1.2.17. Chemotypes A18

General procedure for the synthesis of **A18** derivatives

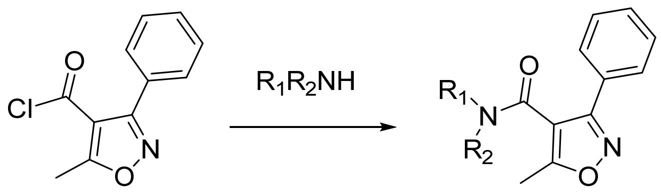

A18

5-Methyl-3-phenyl-1,2-oxazole-4-carbonyl chloride (1 mmol, 1.0 eq) was dissolved in DCM (10 mL) and treated with 2 eq. of appropriate benzylamine (2 mmol) and 2 eq. *N*,*N*-diisopropylethylamine (258 mg, 2 mmol). The mixture was stirred at RT overnight. The solvent was evaporated and crude product was purified with using centrifugal preparative thin layer chromatography (CPTLC, chromatotron, SiO_2) eluting with $CHCl_3$.

N-Benzyl-5-methyl-3-phenylisoxazole-4-carboxamide **A18-1**

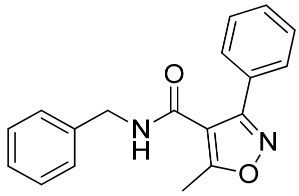

The compound was synthesized from 1-phenylmethanamine according to the general procedure. There was obtained: 140 mg of a light yellow oil product (yield: 70%).

LC-MS: 100% (Rt = 2.95), ESI(+) *m/z* found: 293.26 $[M+H]^+$. Molecular Weight calc'd for $C_{18}H_{16}N_2O_2$ = 292.34.

^{1}H NMR (500 MHz, DMSO-d_6) δ ppm: 8.92 (t, *J* = 6.0 Hz, 1H), 7.67–7.60 (m, 2H), 7.53–7.44 (m, 1H), 7.48–7.40 (m, 2H), 7.38–7.30 (m, 2H), 7.33–7.23 (m, 3H), 4.42 (d, *J* = 6.0 Hz, 2H), 2.51 (s, 3H).

^{13}C NMR (126 MHz, DMSO-d_6) δ ppm: 169.26, 161.43, 160.08, 138.87, 129.96, 128.67, 128.34, 128.15, 127.77, 127.50, 126.97, 112.90, 42.64, 11.79.

N-(4-Methoxybenzyl)-5-methyl-3-phenylisoxazole-4-carboxamide **A18-2**

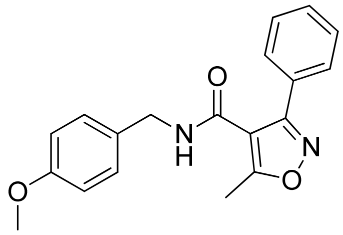

The compound was synthesized from 1-(4-methoxyphenyl)methanamine according to the general procedure. There was obtained: 150 mg of a white solid product (mp = 158–159°C, yield: 68%).

LC-MS: 100% (Rt = 2.90), ESI(+) *m/z* found: 323.06 $[M+H]^+$. Molecular Weight calc'd for $C_{19}H_{18}N_2O_3$ = 322.36.

^{1}**H NMR** (500 MHz, DMSO-d_6) δ ppm: 8.85 (t, *J* = 5.9 Hz, 1H), 7.66–7.60 (m, 2H), 7.53–7.46 (m, 1H), 7.48–7.40 (m, 2H), 7.26–7.19 (m, 2H), 6.94–6.87 (m, 2H), 4.35 (d, J = 5.9 Hz, 2H), 3.74 (s, 3H), 2.49 (s, 3H).

^{13}**C NMR** (126 MHz, DMSO-d_6) δ ppm: 169.20, 161.29, 160.04, 158.32, 130.83, 129.95, 128.86, 128.67, 128.16, 127.74, 113.71, 112.93, 55.07, 42.07, 11.77.

N-(4-Chlorobenzyl)-5-methyl-3-phenyl-1,2-oxazole-4-carboxamide **A18-3**

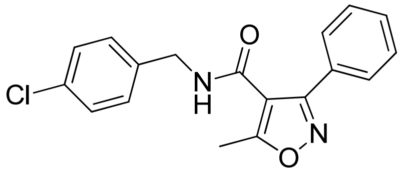

The compound was synthesized from 1-(4-chlorophenyl)methanamine according to the general procedure. There was obtained: 150 mg of a colorless solid product (mp = 160–161 °C, yield: 68%).

LC-MS: 98.8% (Rt = 3.17), ESI(+) *m/z* found: 327.26 $[M+H]^+$. Molecular Weight calc'd for $C_{18}H_{15}ClN_2O_2$ = 326.78.

^{1}**H NMR** (500 MHz, DMSO-d_6) δ ppm: 8.92 (t, J = 6.0 Hz, 1H), 7.65–7.58 (m, 2H), 7.55–7.46 (m, 1H), 7.47–7.39 (m, 4H), 7.37–7.28 (m, 2H), 4.40 (d, J = 6.0 Hz, 2H), 2.51 (s, 3H).

^{13}**C NMR** (126 MHz, DMSO-d_6) δ ppm: 169.37, 161.49, 160.11, 137.96, 131.54, 129.99, 129.40, 128.67, 128.28, 128.11, 127.78, 112.77, 41.98, 11.80.

5-Methyl-3-phenyl-*N*-(1-phenylethyl)-1,2-oxazole-4-carboxamide **A18-4**

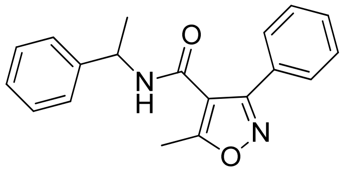

The compound was synthesized from 1-phenylethan-1-amine according to the general procedure. There was obtained: 150 mg of a colorless solid product (mp = 165–166 °C, yield: 72%).

LC-MS: 100% (Rt = 3.08), ESI(+) *m/z* found: 307.20 $[M+H]^+$. Molecular Weight calc'd for $C_{19}H_{18}N_2O_2$ = 306.36.

^{1}**H NMR** (500 MHz, DMSO-d_6) δ ppm: 8.90 (d, *J* = 8.1 Hz, 1H), 7.63–7.57 (m, 2H), 7.52–7.44 (m, 1H), 7.45–7.37 (m, 2H), 7.34 (d, *J* = 4.7 Hz, 4H), 7.31–7.22 (m, 1H), 5.15–5.05 (m, 1H), 2.49 (s, 3H), 1.41 (d, *J* = 7.0 Hz, 3H).

^{13}**C NMR** (126 MHz, DMSO-d_6) δ ppm: δ 169.07, 160.57, 159.96, 144.00, 129.95, 128.67, 128.29, 128.12, 127.64, 126.82, 126.08, 113.02, 48.46, 21.98, 11.71.

1.2.18. Chemotypes A19

General procedure for the synthesis of A19 derivatives

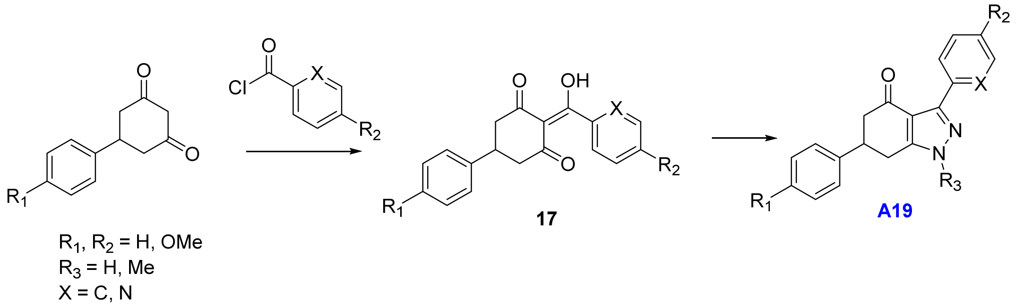

Procedure for synthesis of 2-[hydroxy(aryl)methylidene]-5-arylcyclohexane-1,3-diones **17**

A solution of benzoyl chloride or pyridine-2-carbonyl chloride (2.71 mmol, 1.02 eq.) in dry CH_3CN (1 mL) was added at 5–10 °C to a stirred solution of 5-arylcyclohexane-1,3-dione (2.66 mmol, 1 eq.) and triethylamine (0.37 mL) in dry MeCN (11 mL), and the mixture was stirred at ambient temperature for 2h. Triethylamine (1.12 mL) and acetone cyanohydrin (54 μL) were added successively to the solution and the mixture was stirred at ambient temperature for 24 h. Next the reaction mixture was evaporated, residue was dissolved in DCM and the resulting solution was washed successively with 2N hydrochloric acid (3.4 mL) and water (3x5 mL), dried over $MgSO_4$ evaporated under reduced pressure and crude product was purified by flash chromatography.

2-[Hydroxy(phenyl)methylidene]-5-phenylcyclohexane-1,3-dione (**17a**)

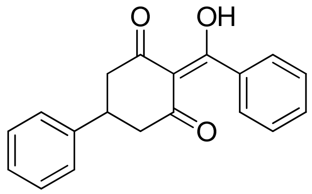

The compound **17a** was synthesized from 5-phenylcyclohexane-1,3-dione and benzoyl chloride according to the general procedure. The crude product was purified *via* silica gel column chromatography eluting with $CHCl_3$/MeOH (49:1). There was obtained: 460 mg of a white solid (yield: 59%).

LC-MS: 100% (Rt = 3.17), ESI(+) *m/z* found: 293.20 $[M+H]^+$. Molecular Weight calc'd for $C_{19}H_{16}O_3$ = 292.33.

2-[Hydroxy(4-methoxyphenyl)methylidene]-5-phenylcyclohexane-1,3-dione (**17b**)

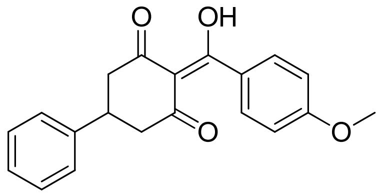

The compound was synthesized from 5-phenylcyclohexane-1,3-dione and 4-methoxybenzoyl chloride according to the general procedure. The crude product was purified *via* silica gel column chromatography eluting with $CHCl_3$/MeOH (49:1). There was obtained: 446 mg of a yellow solid (yield: 52%).

LC-MS: 77.8% (Rt = 3.13), ESI(+) *m/z* found: 293.26 $[M+H]^+$. Molecular Weight calc'd for $C_{18}H_{16}N_2O_2$ = 292.34.

2-[Hydroxy(pyridin-2-yl)methylidene]-5-(4-methoxyphenyl)cyclohexane-1,3-dione (**17c**)

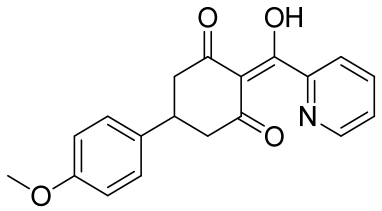

The compound was synthesized from 5-(4-methoxyphenyl)cyclohexane-1,3-dione and pyridine-2-carbonyl chloride according to the general procedure. The crude product was purified *via* silica gel column chromatography eluting with $CHCl_3$/MeOH (49:1). There was obtained: 441 mg of a white solid (yield: 51%).

LC-MS: 90.6% (Rt = 2.06), ESI(+) *m/z* found: 324.25 $[M+H]^+$. Molecular Weight calc'd for $C_{18}H_{16}N_2O_2$ = 323.348.

Procedure for synthesis of 3,6-diaryl-4,5,6,7-tetrahydro-1*H*-indazol-4-ones **A19**

A solution of 2-[hydroxy(aryl)methylidene]-5-arylcyclohexane-1,3-dione (0.74 mmol, 1 eq.) and hydrazine hydrate or methylhydrazine (2.21 mmol, 3 eq.) in EtOH (20 mL) and 0.2 mL DMSO-d_6 was refluxed for 8 h. The solvent was evaporated and crude product was purified by flash chromatography.

1-Methyl-3,6-diphenyl-6,7-dihydro-1*H*-indazol-4(5*H*)-one **A19-1**

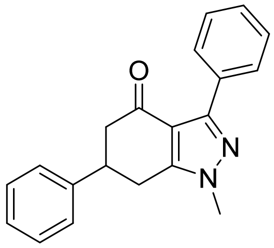

The compound was synthesized from 2-[hydroxy(phenyl)methylidene]-5-phenylcyclohexane-1,3-dione **17a** and methylhydrazine according to the general procedure. The crude product was purified *via* silica gel column chromatography eluting with $CHCl_3$. There was obtained: 336 mg of a colorless solid product (mp.=179–181 °C, yield: 65%).

LC-MS: 96.0% (Rt = 3.18), ESI(+) m/z found: 303.26 $[M+H]^+$. Molecular Weight calc'd for $C_{20}H_{18}N_2O$ = 302.38.

^{1}**H NMR** (500 MHz, DMSO-d_6) δ ppm: δ 8.10–8.06 (m, 2H), 7.44–7.34 (m, 7H), 7.29–7.25 (m, 1H), 3.84 (s, 3H), 3.57–3.50 (m, 1H), 3.25 (ddd, *J* = 16.2, 4.7, 0.9 Hz, 1H), 3.06 (dd, *J* = 16.3, 11.4 Hz, 1H), 2.90 (dd, *J* = 16.0, 12.8 Hz, 1H), 2.54 (ddd, *J* = 16.0, 3.7, 1.0 Hz, 1H).

^{13}**C NMR** (126 MHz, DMSO-d_6) δ ppm: 190.90, 151.48, 148.78, 143.23, 132.24, 128.54(2C), 128.42, 128.18(2C), 127.93(2C), 127.00(2C), 126.82, 114.63, 45.82, 40.33, 36.16, 28.64.

6-(4-Methoxyphenyl)-1-methyl-3-phenyl-6,7-dihydro-1*H*-indazol-4(5*H*)-one **A19-2**

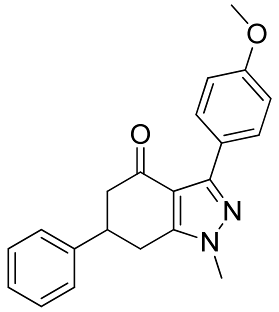

The compound was synthesized from 2-[hydroxy(pyridin-2-yl)methylidene]-5-(4-methoxyphenyl)cyclohexane-1,3-dione **17b** and methylhydrazine according to the general procedure. The crude product was purified *via* silica gel column chromatography eluting with $CHCl_3$. There was obtained: 150 mg of a light beige solid product (mp.=142-144 °C, yield: 61%).

LC-MS: 98.7% (Rt = 3.15), ESI(+) m/z found: 333.26 $[M+H]^+$. Molecular Weight calc'd for $C_{21}H_{20}N_2O_2$ = 332.40.

^{1}**H NMR** (500 MHz, DMSO-d_6) δ ppm: 8.08–8.05 (m, 2H), 7.43–7.30 (m, 5H), 6.95–6.88 (m, 2H), 3.83 (s, 3H), 3.74 (s, 3H), 3.47 (ddd, *J* = 16.1, 8.3, 4.1 Hz, 1H), 3.21 (dd, *J* = 16.3, 4.3 Hz, 1H), 3.01 (dd, *J* = 16.3, 11.3 Hz, 1H), 2.85 (dd, *J* = 16.0, 12.7 Hz, 1H), 2.53 (d, *J* = 3.1 Hz, 1H).

^{13}**C NMR** (126 MHz, DMSO-d_6) δ ppm: 191.04, 158.08, 151.55, 148.74, 135.22, 132.25, 128.40, 128.17(2C), 127.98(2C), 127.92(2C), 114.64, 113.88(2C), 55.06, 46.11, 39.57, 36.14, 28.88.

6-(4-Methoxyphenyl)-1-methyl-3-(pyridin-4-yl)-4,5,6,7-tetrahydro-1*H*-indazol-4-one **A19-3**

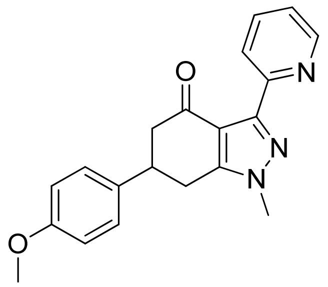

The compound was synthesized from 2-[hydroxy(pyridin-2-yl)methylidene]-5-(4-methoxyphenyl) cyclohexane-1,3-dione **17c** and methylhydrazine according to the general procedure. The crude product was purified *via* silica gel column chromatography eluting with EtOAc/MeOH a ratio of 19:1 (v/v). There was obtained: 27 mg of a colorless solid product (mp.=142–144 °C, yield: 5.24%).

LC-MS: 100% (Rt = 1.91), ESI(+) m/z found: 334.14 $[M+H]^+$. Molecular Weight calc'd for $C_{20}H_{19}N_3O_2$ = 333.39.

^{1}**H NMR** (500 MHz, DMSO-d$_6$) δ ppm: 8.63 (dd, *J* = 4.5, 1.6 Hz, 2H), 8.10 (dd, *J* = 4.5, 1.6 Hz, 2H), 7.35–7.32 (m, 2H), 6.94–6.90 (m, 2H), 3.88 (s, 3H), 3.76–3.72 (m, 3H), 3.51 (tt, *J* = 12.4, 4.1 Hz, 1H), 3.25 (dd, *J* = 16.7, 4.2 Hz, 1H), 3.06 (dd, *J* = 16.3, 11.3 Hz, 1H), 2.90 (dd, *J* = 16.1, 12.7 Hz, 1H), 2.55 (dd, *J* = 15.6, 4.1 Hz, 1H).

^{13}**C NMR** (126 MHz, DMSO-d$_6$) δ ppm: 191.34, 158.10, 152.29, 149.40, 146.10, 139.26, 135.05, 128.00(2C), 122.09, 115.47, 113.91(2C), 55.08, 45.98, 36.41, 28.80.

3,6-Diphenyl-6,7-dihydro-1*H*-indazol-4(5*H*)-one **A19-4**

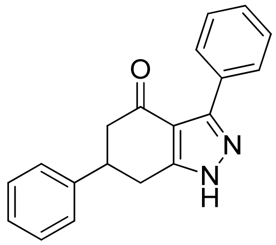

A solution of 2-[hydroxy(phenyl)methylidene]-5-phenylcyclohexane-1,3-dione **17a** (1.06 mmol, 0.31 g) and hydrazine hydrate (0.14 g) in EtOH (20 mL) was refluxed for 8 h. The solvent was evaporated and crude product was purified by silica gel column chromatography eluted with $CHCl_3$/MeOH a ratio of 49:1 (v/v) to provide yellow solid (mp.=255–257 °C, 46 mg, 15% yield).

LC-MS: 100% (Rt = 2.93), ESI(+) m/z found: 289.04 $[M+H]^+$. Molecular Weight calc’d for $C_{19}H_{16}N_2O$= 288.35.

^{1}**H NMR** (500 MHz, DMSO-d$_6$) ppm: 13.51 (d, *J* = 127.2 Hz, 1H), 8.07 (t, *J* = 31.0 Hz, 2H), 7.54–7.31 (m, 7H), 7.25 (t, *J* = 7.2 Hz, 1H), 3.51 (dd, *J* = 55.7, 23.7 Hz, 1H), 3.12 (t, *J* = 22.4 Hz, 2H), 2.91 (dd, *J* = 16.1, 12.3 Hz, 1H), 2.58 (dd, *J* = 16.1, 3.1 Hz, 1H).

^{13}**C NMR** (126 MHz, DMSO-d$_6$) δ ppm: 191.09, 151.93, 149.65, 143.29, 132.70, 128.53(2C), 128.32, 128.19(2C), 127.91, 126.96(2C), 126.75, 114.04, 46.34, 40.74, 28.46.

1.2.19. Chemotypes A20

General procedure for the synthesis of **A20** derivatives

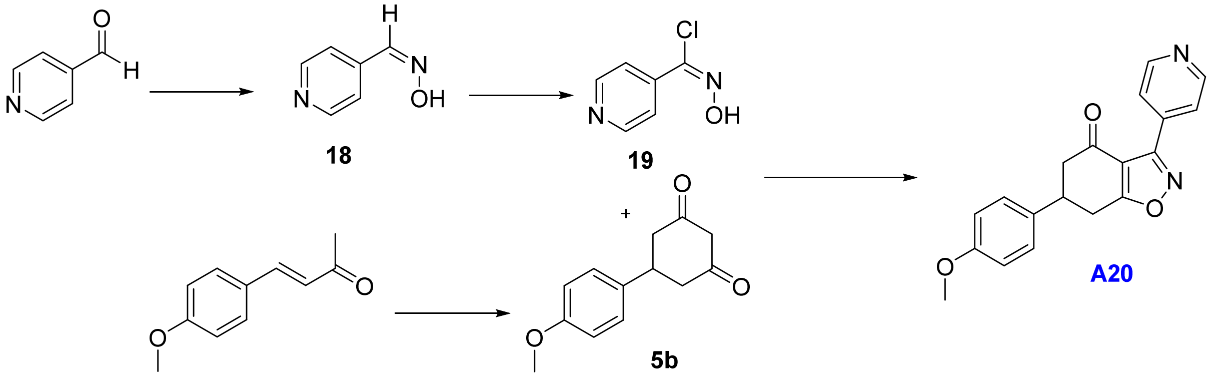

Procedure for synthesis of oximes **18**

The mixture of the benzaldehyde or pyridine-4-carbaldehyde (0.21 mol) and hydroxylamine hydrochloride (0.23 mmol) in MeOH (150 mL) was stirred at 50 °C for 0.5 h and left overnight at RT. The precipitated solid was filtered and washed with MeOH and diethyl ether. The obtained oximes were used in the next step without further purification.

Procedure for synthesis of chlorooximes **19**

To a solution of the oxime (2.75 mmol) in $CHCl_3$ (10 mL), pyridine was added (20 µL, 0.27 mmol). The reaction mixture was heated at 40 °C, and *N*-chlorosuccinimide (405 mg, 3.03 mmol) was added portionwise. After the reaction was complete (3 h, monitored by TLC), the mixture was diluted with DCM (30 mL) and washed with brine (3×10 mL). The organic phase was dried (Na_2SO_4), filtered, and concentrated in vacuo to give the chlorooximes, which were used in the cycloaddition step without further purification.

Procedure for synthesis of diketones **5**

Synthesis described in section 1.2.3 (**A2** derivative).

Procedure for synthesis of isoxazoles **A20**

To a solution of sodium isoproproxide, prepared from 0.135 g of Na in 30 mL of isopropyl alcohol, was added diketone (5.9 mmol) and chlorooxime (2.8 mmol) successively. After stirring 6 h at RT, the reaction mixture was poured into water and extracted with EtOAc (3x45 mL). The combined organic layer was washed with brine, dried over Na_2SO_4, and concentrated under reduced pressure. The product was synthesized according to previously reported methods [12,13].

3,6-Diphenyl-6,7-dihydrobenzo[*d*]isoxazol-4(5*H*)-one **A20-1**

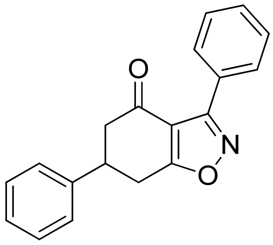

The compound was synthesized from 5-phenylcyclohexane-1,3-dione (**5a**) and *N*-hydroxybenzene-carbonimidoyl chloride according to the general procedure. The crude product was purified *via* silica gel column chromatography eluting with $CHCl_3$/MeOH a ratio of 49:1 (v/v). There was obtained: 280 mg of a light yellow solid product (mp.=161–163 °C, yield: 31%).

LC-MS: 88.2% (Rt = 3.27), ESI(+) m/z found: 294.21 $[M+H]^+$. Molecular Weight calc'd for $C_{19}H_{15}NO_2$ = 293.41.

^{1}**H NMR** (500 MHz, DMSO-d_6) δ ppm: 8.02–7.98 (m, 2H), 7.58–7.51 (m, 3H), 7.44–7.35 (m, 4H), 7.31–7.26 (m, 1H), 3.76–3.68 (m, 1H), 3.45–3.32 (m, 2H), 3.02 (dd, *J* = 16.2, 12.5 Hz, 1H), 2.66 (ddd, *J* = 16.2, 3.8, 0.7 Hz, 1H).

^{13}**C NMR** (126 MHz, DMSO-d_6) δ ppm: 190.97, 182.91, 159.10, 142.25, 130.68, 128.85(2C), 128.65(2C), 128.53(2C), 127.13, 127.04(2C), 127.00, 113.33, 45.51, 39.41, 29.92.

6-(4-Methoxyphenyl)-3-(pyridin-4-yl)-6,7-dihydrobenzo[*d*]isoxazol-4(5*H*)-one **A20-2**

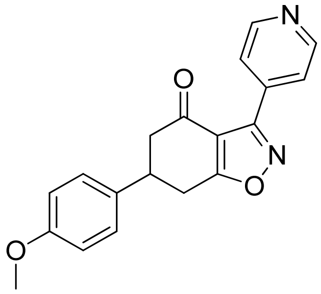

The compound was synthesized from 5-(4-methoxyphenyl)cyclohexane-1,3-dione (**5b**) and *N*-hydroxypyridine-4-carbonimidoyl chloride according to the general procedure. The crude product was purified *via* silica gel column chromatography eluting with $CHCl_3$/MeOH a ratio of 49:1 (v/v). There was obtained: 280 mg of a light yellow solid product (mp.=161–163°C, yield: 31%).

LC-MS: 97.6% (Rt = 2.56), ESI(+) m/z found: 321.01 $[M+H]^+$. Molecular Weight calc'd for $C_{19}H_{16}N_2O_3$ = 320.34.

^{1}**H NMR** (500 MHz, DMSO-d_6) δ ppm: 8.85–8.75 (m, 2H), 8.01–7.95 (m, 2H), 7.36–7.29 (m, 2H), 6.96–6.89 (m, 2H), 3.74 (s, 3H), 3.67 (dddd, *J* = 12.4, 10.7, 4.8, 3.7 Hz, 1H), 3.46–3.30 (m, 2H), 3.00 (dd, *J* = 16.3, 12.5 Hz, 1H), 2.65 (ddd, *J* = 16.2, 3.8, 1.0 Hz, 1H).

^{13}**C NMR** (126 MHz, DMSO-d_6) δ ppm: 191.08, 183.46, 158.24, 157.40, 150.21, 134.61, 134.03, 128.01, 122.83, 114.00, 113.52, 55.07, 45.65, 38.67, 30.11.

1.2.20. Chemotypes A21

General procedure for the synthesis of 21 derivatives

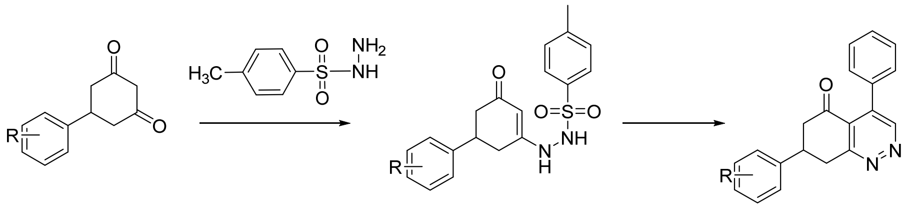

5 20 A21

Procedure for the synthesis of intermediates **20**

A mixture of diketone (**5**) (2 mmol, 1.0 eq) and corresponding benzenesulfonhydrazide (2 mmol, 1.0 eq), in EtOH (20 mL) was refluxed for 1 h and left for overnight stirring at RT. The beige solid product was filtered to give desired product.

Procedure for synthesis of A21 derivatives

A mixture of **20** (1 mmol, 1.0 eq), K_2CO_3 (2.5 mmol, 2.5 eq), 2-bromoacetophenone (1.3 mmol, 1.3 eq) in 1,2-dimethoxyethane (20 mL) was stirred under reflux for 3 h. Next the reaction mixture was cooled down and evaporated. Crude product was purified *via* column chromatography eluting with EtOAc/hexane a ratio of 1:2 (v/v) to give desired A21 derivative.

Synthesis of intermediate **20a**

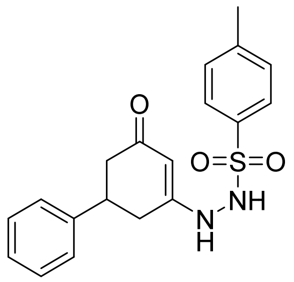

The compound **20a** was synthesized from compound **5a** according to the general procedure. There was obtained: 0.93 g of a colorless solid product (yield: 65.5%), mp: 223-224 °C.

LC-MS: 98.89% (Rt = 2.59), ESI(+) m/z found: 356.90 $[M+H]^+$. Molecular Weight calc'd for $C_{19}H_{20}N_2O_3S$ = 356.44

Synthesis of intermediate **20b**

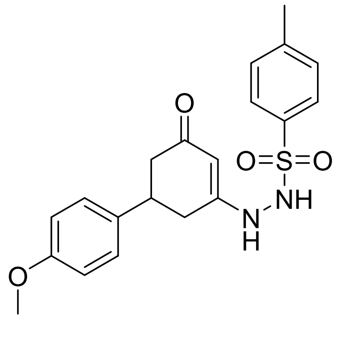

The compound **20b** was synthesized from compound **5b** according to the general procedure. There was obtained: 0.81 g of a colorless solid product (yield: 69.0%), mp: 209-210 °C.

LC-MS: 100% (Rt = 2.63), ESI(+) m/z found: 387.37 $[M+H]^+$. Molecular Weight calc'd for $C_{20}H_{22}N_2O_4S$ = 386.47

Synthesis of intermediate **20c**

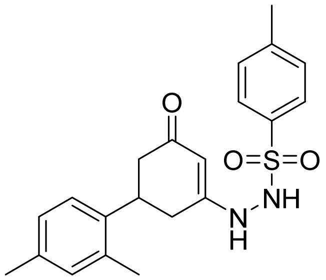

The compound **20b** was synthesized from compound **5b** according to the general procedure. There was obtained: 2.31 g of a colorless solid product (yield: 75%), mp: 198-200 °C.

LC-MS: 95.34% (Rt = 2.88), ESI(+) m/z found: 385.26 $[M+H]^+$. Molecular Weight calc'd for $C_{21}H_{24}N_2O_3S$ = 384.49

Synthesis of intermediate **20d**

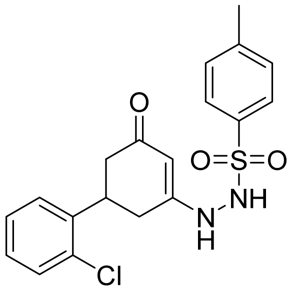

The compound **20d** was synthesized from compound **5d** according to the general procedure. There was obtained:1.69 g of a colorless solid product (yield: 62%), mp: 218-220 °C.

LC-MS: 91.24% (Rt = 2.76), ESI(+) m/z found: 391.06 $[M+H]^+$. Molecular Weight calc'd for $C_{19}H_{19}ClN_2O_3S$ = 390.88

4,7-Diphenyl-7,8-dihydrocinnolin-5(6*H*)-one **A21-1**

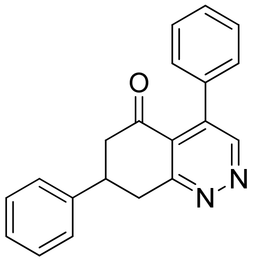

4,7-diphenyl-7,8-dihydrocinnolin-5(6*H*)-one **A21-1** was prepared from a mixture of 4-methyl-*N*'-(5-oxo-1,2,5,6-tetrahydro-[1,1'-biphenyl]-3-yl)benzenesulfonohydrazide (**20a**) (0.36 g, 1 mmol), K_2CO_3 (0.35 g, 2.5 mmol), 2-bromoacetophenone (0.26 g, 1.3 mmol) in 1,2-dimethoxyethane (20 mL) according to general procedure. Crude product was purified *via* column chromatography eluting with EtOAc/hexane a ratio of 1:2 (v/v) to give 0.13 g 4,7-diphenyl-7,8-dihydrocinnolin-5(6*H*)-one **A21-1.**

LC-MS: 100% (Rt = 3.04), ESI(+) m/z found: 301.36 $[M+H]^+$. Molecular Weight calc'd for $C_{20}H_{16}N_2O$ = 300.13.

^{1}H NMR (500 MHz, $CDCl_3$) δ ppm: 9.18 (s, 1H), 7.52–7.44 (m, 3H), 7.40 (dd, *J* = 10.3, 4.7 Hz, 2H), 7.32 (ddd, *J* = 9.2, 6.1, 2.2 Hz, 5H), 3.90–3.81 (m, 1H), 3.66–3.51 (m, 2H), 3.07–2.92 (m, 2H).

^{13}C NMR (126 MHz, $CDCl_3$) δ ppm: 196.90, 196.79, 161.29, 153.42, 141.78, 138.68, 135.00, 129.16, 129.05, 128.47(2C), 128.45(2C), 127.45, 126.61(2C), 125.11, 47.07, 38.93, 38.24.

7-(4-Methoxyphenyl)-4-phenyl-7,8-dihydrocinnolin-5(6*H*)-one **A21-2**

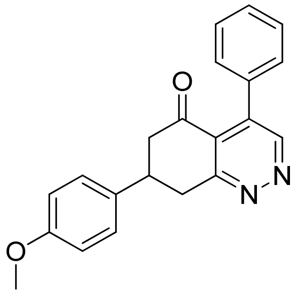

7-(4-methoxyphenyl)-4-phenyl-7,8-dihydrocinnolin-5(6*H*)-one was synthesized from a mixture of *N*'-(4'-methoxy-5-oxo-1,2,5,6-tetrahydro-[1,1'-biphenyl]-3-yl)benzenesulfonohydrazide (**20b**, 0.58 g, 1.5 mmol), K_2CO_3 (0.52 g, 3.75 mmol), 2-bromoacetophenone (0.39 g, 1.95 mmol) in 1,2-dimethoxyethane (20 mL) according to general procedure. Crude product was purified *via* column chromatography eluting with EtOAc/hexane a ratio of 1:2 (v/v) to give 0.048 g 7-(4-methoxyphenyl)-4-phenyl-7,8-dihydrocinnolin-5(6*H*)-one **A21-2**.

LC-MS: 100% (Rt = 3.01), ESI(+) m/z found: 331.27 $[M+H]^+$. Molecular Weight calc'd for $C_{21}H_{18}N_2O_2$ = 330.38.

^{1}H NMR (500 MHz, $CDCl_3$) δ ppm: 9.17 (s, 1H), 7.50–7.45 (m, 3H), 7.34–7.31 (m, 2H), 7.25–7.23 (m, 2H), 6.97–6.89 (m, 2H), 3.89–3.84 (m, 1H), 3.82 (s, 3H), 3.60–3.49 (m, 2H), 3.05–2.90 (m, 2H).

^{13}C NMR (126 MHz, $CDCl_3$) δ ppm: 196.70, 161.38, 158.84, 153.40, 134.87, 133.76, 129.30, 128.52(2C), 128.48(2C), 127.62(2C), 114.40(2C), 55.35, 47.33, 38.29, 38.12.

7-(2,4-Dimethylphenyl)-4-phenyl-7,8-dihydrocinnolin-5(6*H*)-one **A21-3**

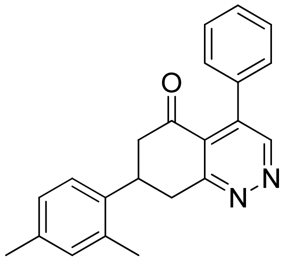

7-(2,4-dimethylphenyl)-4-phenyl-7,8-dihydrocinnolin-5(6*H*)-one was synthesized from a mixture of *N*'-(2',4'-dimethyl-5-oxo-1,2,5,6-tetrahydro-[1,1'-biphenyl]-3-yl)-4-methylbenzenesulfonohydrazide (**20c**, 0.58 g, 1.5 mmol), K_2CO_3 (0.52 g, 3.75 mmol), 2-bromoacetophenone (0.39 g, 1.95 mmol) in 1,2-dimethoxyethane (20 mL) according to general procedure. Crude product was purified *via* column chromatography eluted with EtOAc/hexane a ratio of 1:2 (v/v) to give 0.100 g of 7-(2,4-dimethylphenyl)-4-phenyl-7,8-dihydrocinnolin-5(6*H*)-one **A21-3.**

LC-MS: 100% (Rt = 3.32), ESI(+) m/z found: 329.13 $[M+H]^+$. Molecular Weight calc'd for $C_{22}H_{20}N_2O$ = 328.41.

^{1}H NMR (500 MHz, $CDCl_3$) δ ppm: 9.17 (s, 1H), 7.51–7.45 (m, 3H), 7.36–7.30 (m, 2H), 7.19 (d, *J* = 7.8 Hz, 1H), 7.11–7.03 (m, 2H), 3.81–3.70 (m, 2H), 3.48 (dd, *J* = 17.9, 13.0 Hz, 1H), 2.99–2.88 (m, 2H), 2.34 (d, *J* = 14.5 Hz, 6H).

^{13}C NMR (126 MHz, $CDCl_3$) δ ppm: 197.18, 161.62, 153.41, 138.77, 136.98, 136.81, 135.31, 135.05, 131.85, 129.15, 128.47, 128.46(2C), 127.34, 125.15, 124.86, 46.78, 37.84, 34.78, 29.30, 20.92, 19.30.

7-(2,4-Dimethylphenyl)-4-(pyridin-4-yl)-7,8-dihydrocinnolin-5(6*H*)-one **A21-4**

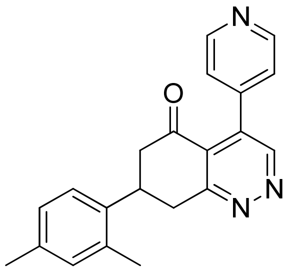

7-(2,4-dimethylphenyl)-4-(pyridin-4-yl)-7,8-dihydrocinnolin-5(6*H*)-one was synthesized from a mixture of *N*'-(2',4'-dimethyl-5-oxo-1,2,5,6-tetrahydro-[1,1'-biphenyl]-3-yl)-4-methylbenzenesulfono hydrazide (**20c**, 0.58 g, 1.5 mmol), K_2CO_3 (0.52 g, 3.75 mmol), 2-bromo-1-(4-pyridinyl)-1-ethanone hydrobromide (0.55 g, 1.95 mmol) in 1,2-dimethoxyethane (40 mL) according to general procedure. Crude product was purified *via* column chromatography eluted with EtOAc/hexane a ratio of 1:2 (v/v) to give 0.180 g of 7-(2,4-dimethylphenyl)-4-(pyridin-4-yl)-7,8-dihydrocinnolin-5(6*H*)-one **A21-4.**

LC-MS: 97.3% (Rt = 2.93), ESI(+) m/z found: 330.20 $[M+H]^+$. Molecular Weight calc'd for $C_{21}H_{19}N_3O$ = 329.40.

^{1}H NMR (500 MHz, DMSO-d_6) δ ppm: 9.37 (s, 1H), 9.08 (t, *J* = 6.7 Hz, 2H), 8.26 (dd, *J* = 32.2, 5.9 Hz, 2H), 7.30 (d, *J* = 7.8 Hz, 1H), 7.10–7.00 (m, 2H), 3.90 (t, *J* = 12.5 Hz, 1H), 3.63–3.46 (m, 2H), 3.09 (dd, *J* = 16.6, 13.6 Hz, 1H), 2.70 (d, *J* = 16.6 Hz, 1H), 2.38–2.18 (m, 6H).

^{13}C NMR (126 MHz, DMSO-d_6) δ ppm: 197.22, 161.73, 154.31, 150.90, 141.22, 137.82, 135.68, 135.15, 132.47, 131.21, 127.03, 125.38, 124.75, 45.21, 36.72, 33.41, 20.55, 18.93.

7-(2-chlorophenyl)-4-(pyridin-4-yl)-7,8-dihydrocinnolin-5(6*H*)-one **A21-5**

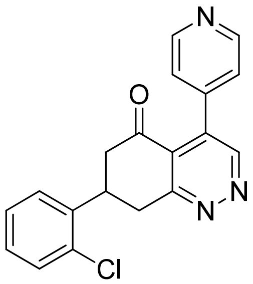

7-(2-chlorophenyl)-4-(pyridin-4-yl)-7,8-dihydrocinnolin-5(6*H*)-one was synthesized from a mixture of *N*'-(2'-chloro-5-oxo-1,2,5,6-tetrahydro-[1,1'-biphenyl]-3-yl)-4-methylbenzenesulfonohydrazide (**20d**, 0.59 g, 1.5 mmol), K_2CO_3 (0.52 g, 3.75 mmol), 2-bromo-1-(4-pyridinyl)-1-ethanone hydrobromide (0.55 g, 1.95 mmol) in 1,2-Dimethoxyethane (40 mL) according to general procedure. Crude product was purified *via* column chromatography eluted with EtOAc/hexane a ratio of 1:2 (v/v) to give 0.07 g of 7-(2-chlorophenyl)-4-(pyridin-4-yl)-7,8-dihydrocinnolin-5(6*H*)-one **A21-5**.

LC-MS: 100% (Rt = 2.12), ESI(+) m/z found: 335.70 $[M+H]^+$. Molecular Weight calc'd for $C_{19}H_{14}ClN_3O$ = 336.0.

^{1}H NMR (500 MHz, $CDCl_3$) δ ppm: 9.15 (s, 1H), 8.74 (d, *J* = 6.0 Hz, 2H), 7.47–7.42 (m, 1H), 7.33 (dd, *J* = 10.7, 2.4 Hz, 2H), 7.31–7.23 (m, 3H), 4.13–4.04 (m, 1H), 3.96–3.87 (m, 1H), 3.56 (dd, *J* = 17.0, 11.9 Hz, 1H), 3.10–3.02 (m, 1H), 2.92 (dd, *J* = 16.8, 12.9 Hz, 1H).

^{13}C NMR (126 MHz, $CDCl_3$) δ ppm: 196.15, 161.40, 152.16, 149.42 (2C), 143.97, 138.62, 135.58, 133.71, 130.35, 128.76, 127.58, 126.70, 124.40, 122.96, 45.39, 36.63, 35.44, 30.91.

1.2.21. Chemotypes A22

General procedure for the synthesis of A22 derivatives

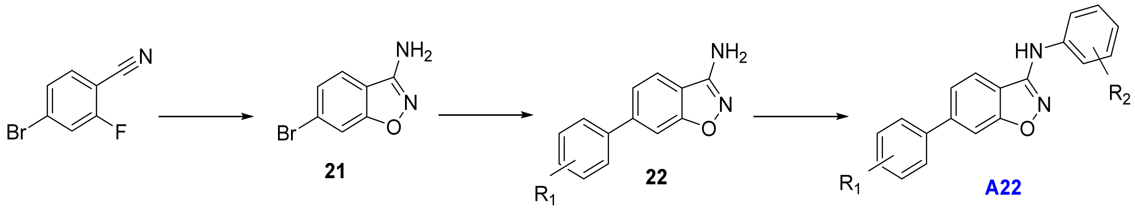

Procedure for synthesis of 6-bromobenzo[*d*]isoxazol-3-amine (**21**)

N-hydroxyacetamide (206 mg, 2.75 mmol, 1.1 eq.) and potassium tert-butoxide (309 mg, 2.75 mmol, 1.1 eq.) were suspended in DMF (5 mL), and aged at RT for 30 min. Then 4-bromo-2-fluorobenzonitrile (500 mg, 2.5 mmol, 1.0 eq.) was added and the reaction mixture was held at 50 °C for another 3 h. Then the mixture was left stirring at RT/overnight. The reaction mixture was diluted with ethyl acetate, quenched with water and extracted thrice with ethyl acetate. The combined organic layers were washed with brine, dried over $MgSO_4$ and evaporated. The crude product was purified *via* silica gel column chromatography eluted with hexane/EtOAc a ratio of 2:1 =>1:1 (v/v), followed by trituration with 2-PrOH/hexane a 1:2 (v/v) mixture to give 6-bromobenzo[*d*]isoxazol-3-amine (**21**, 118 mg, 22.3%).

LC-MS: 100% (Rt = 2.37), ESI(+) m/z found: 214.0 $[M+H]^+$. Molecular Weight calc'd for $C_7H_5BrN_2O$ = 213.03.

Procedure for synthesis of 6-(2-chlorophenyl)benzo[*d*]isoxazol-3-amine (**22**)

A mixture of 6-bromobenzo[*d*]isoxazol-3-amine (**21**) (1.0 eq), corresponding arylboronic acid (1.2eq), potassium carbonate (3.0 eq.) and 2N Na_2CO_3 aq solution (2.0 eq) in 1,4-dioxane (15 vol/1 g of 10) was degassed with argon and next Pd(dppf)Cl_2 complex in DCM (0.10 eq.) was added. The reaction mixture was stirred in a sealed tube at 80 °C overnight. Then the mixture was cooled down to RT, diluted with chloroform, quenched with water and thrice extracted with chloroform. The combined organic layers were washed with brine, dried over $MgSO_4$ and evaporated. Crude product was purified by silica gel column chromatography, followed by trituration with appropriate solvent to give desirable **22**.

Procedure for synthesis of **A22** derivatives (Buchwald–Hartwig amination)

Corresponding amine (**22**) (1.5 eq), appropriate aryl halides (1.0eq) and cesium carbonate (2.0 eq.) were suspended in dry 1,4-dioxane/toluene a 1:2 (v/v) mixture, and degassed with argon. Next tris(dibenzylideneacetone)dipalladium (0) (0.03 eq.) and (±)-2,2′-bis(diphenylphosphino)-1,1′-binaphthalen BINAP (0.05 eq.) were added. The reaction mixture was stirred at 80 °C in a sealed tube overnight. Then the mixture was cooled down, diluted with dichloromethane and filtered through the celit pad. The filtrate was quenched with water and product was extracted twice to DCM. The combined organic layers were dried over Na_2SO_4 and evaporated to dryness. Crude product was purified *via* silica gel column chromatography eluted with hexane/EtOAc a ratio of 2:1 => 1:2 (v/v), followed by trituration with $CHCl_3$/hexane a 2:1 (v/v) mixture to give desirable **A22** derivative

Procedure for synthesis of **A22** derivatives (Chan-Lam coupling)

Starting amine (**22**) (1.0 eq.), corresponding arylboronic acid (1.0 eq.) and potassium carbonate (1.2 eq.) were suspended in EtOAc (20 vol/1 g of amine **22**). The copper (II) acetate (0.2 eq.) was added and reaction was stirred at 85 °C for 4 days under reflux condenser. Then the reaction mixture was cooled down, diluted with ethyl acetate, quenched with water and extracted twice to ethyl acetate. The combined organic layers were dried over $MgSO_4$, evaporated to dryness. The crude product was purified *via* silica gel column chromatography to give desirable **A22** derivative.

Synthesis of 6-(2-chlorophenyl) benzo[*d*]isoxazol-3-amine (**22a**)

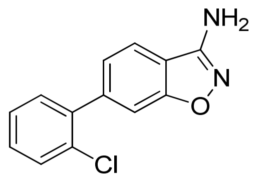

Compound **22a** was prepared from 6-bromobenzo[*d*]isoxazol-3-amine **21** (1.0 g, 4.69 mmol) and 2-chlorobenzeneboronic acid (880 mg, 5.6 mmol) according to general procedure. The crude product was purified *via* silica gel column chromatography eluted with hexane/EtOAc a ratio of 2:1 => 1:2 (v/v), followed by trituration with 2-PrOH/hexane a 1:3 (v/v) mixture to give desirable product as a solid (480 mg, 41.74%).

LC-MS: 100% (Rt = 2.29), ESI (+) m/z found: 245.0 $[M+H^+]$. Molecular Weight calc'd for $C_{13}H_9ClN_2O$ = 244.68.

Synthesis of 6-(4-methoxyphenyl) benzo[*d*]isoxazol-3-amine (**22b**)

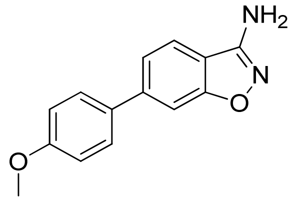

Compound **22b** was prepared from 6-bromobenzo[*d*]isoxazol-3-amine (1.0 g, 4.69 mmol) and 4-methoxyphenylboronic acid (930 mg, 6.10 mmol) according to general procedure. Crude product was purified *via* silica gel column chromatography eluted with hexane/EtOAc a ratio of 2:1 => 1:2), to give desirable product as a solid (465 mg, 41.15%).

LC-MS: 94% (Rt = 2.87), ESI (+) m/z found: 241.08 [M+H+]. Molecular Weight calc'd for $C_{14}H_{12}N_2O_2$ = 240.25.

6-(4-Methoxyphenyl)-*N*-(pyridin-4-yl)benzo[*d*]isoxazol-3-amine **A22-1**

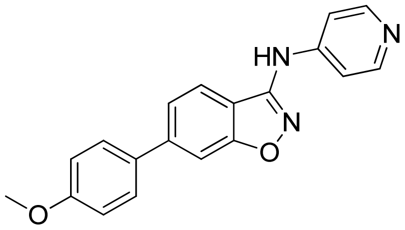

Compound **A22-1** was prepared from 4-iodopyridine (90 mg, 4.4 mmol, 1.0 eq.) and 6-(4-methoxyphenyl)benzo[*d*]isoxazol-3-amine (**22b**) (158 mg, 6.6 mmol, 1.5 eq.) according general procedure of Buchwald–Hartwig amination. Crude product was purified *via* silica gel column chromatography eluted with DCM/MeOH (100% DCM => a ratio of 8:2 (v/v)), followed by trituration with $CHCl_3$/hexane a 1:2 (v/v) mixture to give desirable 6-(4-methoxyphenyl)-*N*-(pyridin-4-yl)benzo[*d*]isoxazol-3-amine **A22-1** (15 mg solid product, 11%).

LC-MS: 98.0% (Rt =), ESI2.32 (+) *m/z* found: 318.10 $[M+H]^+$ Molecular Weight calc'd for $C_{19}H_{15}N_3O_2$ =317.34.

^{1}H NMR (500 MHz, DMSO-d_6) δ ppm: 10.13 (s, 1H), 8.46 (s br, 2H), 8.16 (d, *J* = 8.3 Hz, 1H), 7.89 (s, 1H), 7.80–7.75 (m, 2H), 7.72 (dd, *J* = 8.4, 1.3 Hz, 1H), 7.65 (d, *J* = 5.8 Hz, 2H), 7.13–7.00 (m, 2H), 3.82 (s, 3H).

^{13}C NMR (126 MHz, DMSO-d_6) δ 162.27, 159.61, 154.67, 150.28(2C), 146.95, 142.85, 131.32, 128.59(2C), 122.10, 121.79, 114.75, 114.50(2C), 112.00(2C), 106.64, 55.27.

6-(4-Methoxyphenyl)-*N*-phenylbenzo[*d*]isoxazol-3-amine **A22-2**

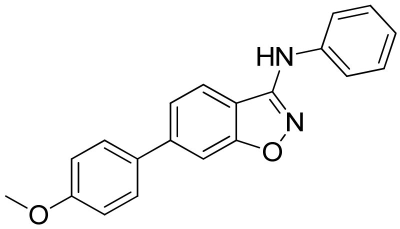

Compound A22-2 was prepared from 6-(4-methoxyphenyl) benzo[*d*]isoxazol-3-amine (**22b**) (0.200 g, 8.3 mmol, 1.0 eq.) and benzeneboronic acid (0.101 g, 8.3 mmol, 1.0 eq.) according to Chan-Lam coupling conditions. Crude product was purified *via* silica gel column chromatography eluted with DCM/MeOH (a ratio of 99:1 => 49:1 (v/v)) to give 6-(4-methoxyphenyl)-*N*-phenylbenzo[*d*]isoxazol-3-amine A22-2 (6 mg of white product, 2%).

LC-MS: 95.0% (Rt = 3.68), ESI(+) *m/z* found: 317.14 $[M+H]^+$. Molecular Weight calc'd for $C_{20}H_{16}N_2O_2$ = 316.35.

^{1}H NMR (500 MHz, DMSO-d$_6$) δ ppm: 9.57 (s, 1H), 8.17 (d, *J* = 8.3 Hz, 1H), 7.82 (s, 1H), 7.79–7.74 (m, 2H), 7.71 (dd, *J* = 8.6, 0.9 Hz, 2H), 7.67 (dd, *J* = 8.4, 1.4 Hz, 1H), 7.41–7.35 (m, 2H), 7.10–7.04 (m, 2H), 7.01–6.96 (m, 1H), 3.82 (s, 3H).

^{13}C NMR (126 MHz, DMSO-d$_6$) δ ppm: 161.99, 159.53, 154.97, 142.51, 140.78, 131.50, 129.00(2C), 128.54(2C), 121.85, 121.63, 121.04, 117.45(2C), 115.27, 114.48(2C), 106.52, 55.26.

6-Phenyl-*N*-(pyridin-4-yl)benzo[*d*]isoxazol-3-amine A22-3

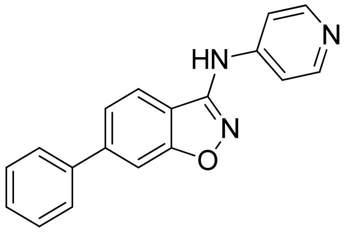

Compound A22-3 was prepared from 4-iodopyridine (140 mg, 6.8 mmol, 1.0 eq.) and 6-phenylbenzo[*d*]isoxazol-3-amine (**22a**) (251 mg, 1.2 mmol, 1.75 eq.) according to Buchwald–Hartwig amination conditions. The reaction was stirred in a sealed tube at 85 °C for 3 days. Crude product was purified *via* silica gel column chromatography eluted with hexane/EtOAc a ratio of 2:1 => 1:2 (v/v). There was obtained: 53 mg of oily product which was next precipitated from $CHCl_3$/hexane a 2:1 (v/v) mixture to give 6-phenyl-*N*-(pyridin-4-yl) benzo[*d*]isoxazol-3-amine A22-3 (11 mg of solid product, 5.5%).

LC-MS: 93.4% (Rt = 2.30), ESI(+) *m/z* found: 288.15 $[M+H]^+$. Molecular Weight calc'd for $C_{18}H_{13}N_3O$ = 287.32.

^{1}H NMR (500 MHz, DMSO-d$_6$) δ ppm: 10.17 (s, 1H), 8.46 (s, 2H), 8.21 (dd, *J* = 8.3, 0.4 Hz, 1H), 7.95 (dd, *J* = 1.3, 0.6 Hz, 1H), 7.81 (dt, *J* = 8.3, 1.7 Hz, 2H), 7.76 (dd, *J* = 8.4, 1.4 Hz, 1H), 7.66 (d, *J* = 6.0 Hz, 2H), 7.55–7.50 (m, 2H), 7.47–7.42 (m, 1H).

^{13}C NMR (126 MHz, DMSO-d$_6$) δ ppm: 162.14, 154.69, 150.27(2C), 146.94, 143.19, 139.13, 129.09(2C), 128.37, 127.43(2C), 122.58, 121.94, 115.38, 112.02(2C), 107.46.

6-(2-chlorophenyl)-*N*-(pyridin-4-yl)benzo[*d*]isoxazol-3-amine, A22-4

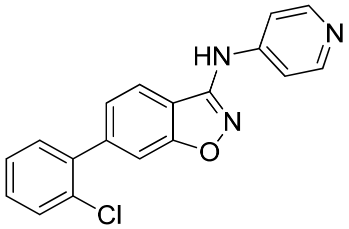

The product was synthesized from 4-iodopyridine (0.110 g, 0.54 mmol, 1.0 eq.) and 6-(2-chlorophenyl) benzo [*d*]isoxazol-3-amine **22b** (0.197 g, 0.80 mmol, 1.50 eq.) using Buchwald–Hartwig amination conditions. The reaction mixture was stirred in a sealed tube at 85 °C for 30 h. The crude product was purified *via* silica gel column chromatography eluted with DCM/MeOH a ratio of 1:0 => 9:1 (v/v). There was obtained: 55 mg of oily product which was next precipitated from $CHCl_3$/hexane a 2:1 (v/v) mixture to 6-(2-chlorophenyl)-*N*-(pyridin-4-yl) benzo[*d*]isoxazol-3-amine **A22-4** (29 mg, 17%).

LC-MS: 94.6% (Rt = 2.37), ESI(+) m/z found: 322.01 $[M+H]^+$. Molecular Weight calc'd for $C_{18}H_{12}N_3O$ = 321.76.

^{1}H NMR (500 MHz, DMSO-d_6) δ ppm: 10.19 (s, 1H), 8.47 (dd, *J* = 4.8, 1.5 Hz, 2H), 8.24–8.19 (m, 1H), 7.72 (dd, *J* = 1.2, 0.6 Hz, 1H), 7.66 (dd, *J* = 4.8, 1.6 Hz, 2H), 7.64–7.59 (m, 1H), 7.54–7.49 (m, 1H), 7.49–7.44 (m, 3H).

^{13}C NMR (126 MHz, DMSO-d_6) δ ppm: 161.22, 154.75, 150.33(2C), 146.89, 141.45, 138.89, 131.73, 131.37, 129.95, 129.86, 127.61, 124.93, 121.30, 115.65, 112.06(2C), 110.41.

1.3. Crystal structure determination

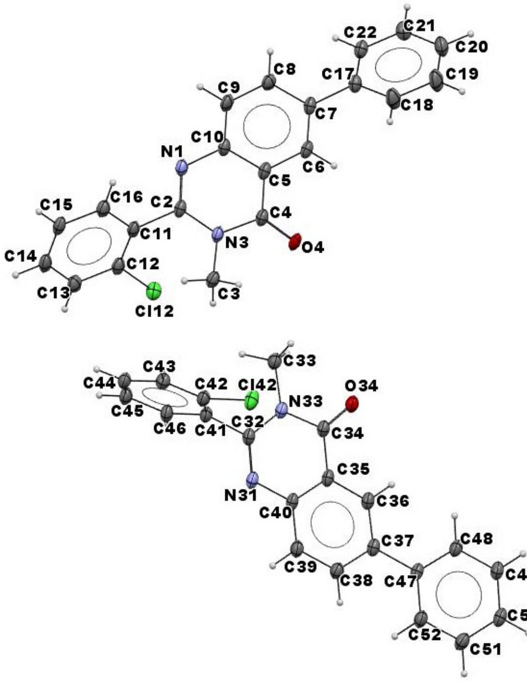

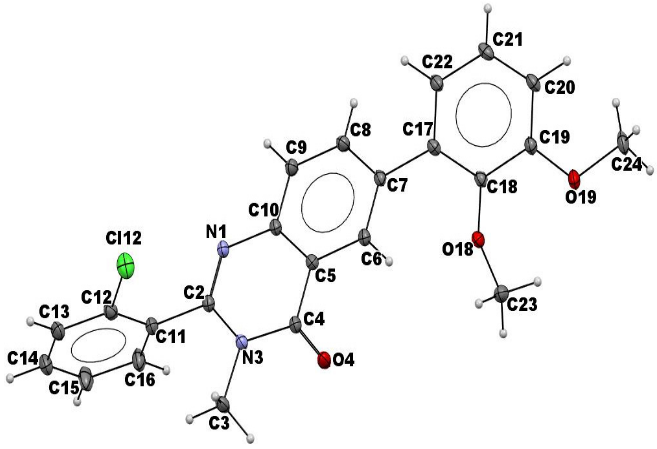

ALX-065 ALX-171

Figure S1. Molecular geometry observed in the asymmetric units of crystal structures of **ALX-065** and **ALX-171**, presenting the numbering scheme. The asymmetric unit of **ALX-065** consists of two independent molecules representing two alternative conformations in the crystal environment. Displacement ellipsoids of non-hydrogen atoms are drawn at the 30% probability level. H atoms are presented as small spheres with an arbitrary radius.

Table S3 Crystal data and structure refinement results

	ALX-065	ALX-171
Empirical moiety formula	2 × $C_{21}H_{15}ClN_2O$	$C_{23}H_{19}ClN_2O_3$
Formula weight [g/mol]	693.60	406.85
Crystal system	Triclinic	Triclinic
Space group	$P\bar{1}$	$P\bar{1}$
Unite cell dimensions	a = 9.4902(4) Å<br>b = 10.2208(5) Å<br>c = 18.0336(9) Å<br>α = 89.591(4)°<br>β = 86.575(4)°<br>γ = 69.191(4)°	a = 7.1641(2) Å<br>b = 10.6034(3) Å<br>c = 14.2601(4) Å<br>α = 74.706(3)°<br>β = 77.131(2)°<br>γ = 70.713(3)°
Volume [Å^3]	1632.01(14)	975.25(5)
Z	2	2
Dcalc [Mg/m^3]	1.411	1.385
μ [mm^{-1}]	2.154	1.964
F(000)	720	424

Crystal size [mm^3]	0.2 × 0.15 × 0.1	0.3 × 0.2 × 0.07
Θ range	2.455° to 75.290°	3.249° to 80.552°
Index ranges	-10 ≤ h ≤ 11<br>-12 ≤ k ≤ 12<br>-22 ≤ l ≤ 22	-9 ≤ h ≤ 9<br>-13 ≤ k ≤ 12<br>-17 ≤ l ≤ 18
Refl. collected	17776	11597
Independent reflections	6478<br>[R(int) = 0.0583]	4172<br>[R(int) = 0.0351]
Completeness [%] to<br>Θ = 67.68°	99.4	99.9
Absorption correction	Multi-scan	Multi-scan
Tmin. and Tmax.	0.500 and 1.000	0.877 and 1.000
Data/ restraints/parameters	6478 / 0 / 453	4172 / 0 / 265
GooF on F_2	1.046	1.084
Final R indices [I>2sigma(I)]	R_1= 0.0847<br>wR_2= 0.2274	R_1= 0.0433<br>wR_2= 0.1186
R indices (all data)	R_1 = 0.0920<br>wR_2 = 0.2343	R_1 = 0.0467<br>wR_2 = 0.1216
Δϱmax, Δϱmin [e·Å^{-3}]	1.317 and -0.566	0.403 and -0.401

2. In vitro pharmacology

2.1. NAM mGlu$_7$ receptor activity on T-Rex 293 cell line

The activity of compounds was examined on T-REx 293 cell line expressing recombinant human mGlu$_7$ receptor by detecting the level of cyclic AMP in the presence of 5 μM forskolin [14,15]. The cell line was verified using reference negative allosteric modulators ADX71743 and MMPIP. The substances were incubated with 5 μM of LSP4-2022 (EC_{80}). Both ADX71743 and MMPIP dose-dependently antagonized LSP4-2022 in presence of forskolin, with IC_{50} 0.58 μM (±0.27, n=5) and 0.54 μM (±0.470, n=3), respectively (**Figure S2**)

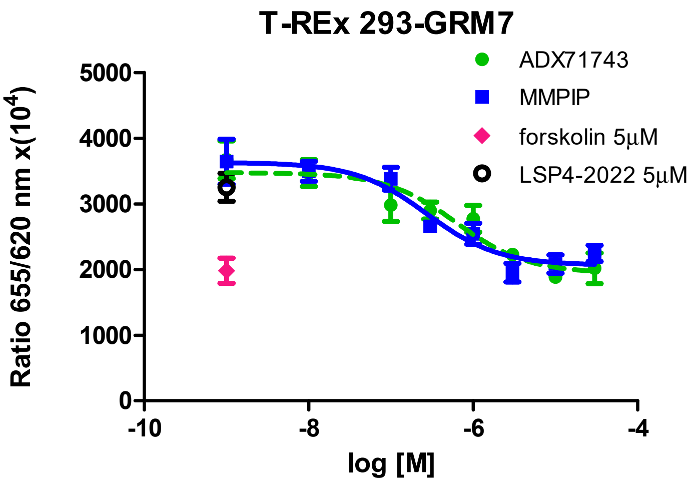

Figure S2. Representative results demonstrating activity of NAMs ADX71743 and MMPIP in T-REx 293 cell line with inducible expression of human mGlu7 receptor. Both compounds inhibit activity of the receptor in dose dependent manner in presence of unselective agonist LSP4-2022 in concentration corresponding to its EC_{80} (5 μM) and 5 μM of forskolin, an activator of adenylate cyclase.

ALX-063

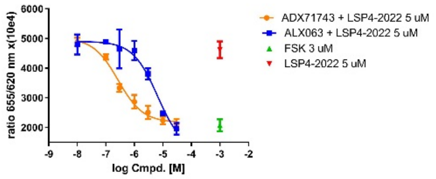

IC_{50}: 6.50 ± 0.14

% inhibition: 42.55 ± 36.5

ALX-065 hit compound

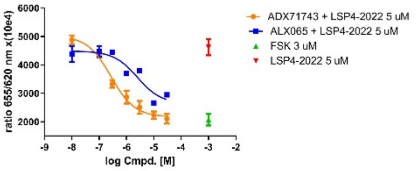

IC_{50}: 4.65 ± 3.46

% inhibition: 26.5 ± 11.8

ALX-171 early lead

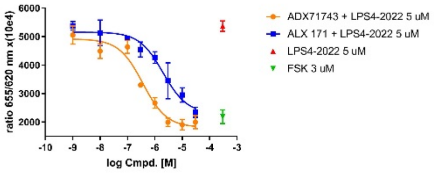

IC_{50}: 6.14 ± 3.42

% inhibition:30.77 ± 6.44

Figure S3. Representative results demonstrating activity of NAMs **ALX-063**, **ALX-065**, **ALX-171** in T-REx 293 cell line with inducible expression of human mGlu7 receptor All compounds inhibit activity of the receptor in dose dependent manner in presence of unselective agonist LSP4-2022 in concentration corresponding to its EC_{80} (5 μM) and 5 μM of forskolin, an activator of adenylate cyclase.

2.2. NAM mGlu7 receptor activity on CHO-K1 cell line according to EuroscreenFast protocol

ALX-065 and **ALX-171** were testing for negative allosteric modulator (NAM) activity on alternative mGlu7 cell line (recombinant human mGlu7 receptor (FAST-0154C) using cAMP HTRF functional assay) by EuroscreenFast company according to the provided methodology.

Cell line

Receptor	Accession Number	Cell line	Reference agonist	Reference antagonist
mGlu7	NP_000835.1	CHO-K1	L-AP4	MMPIP

Compound Testing

Compounds were tested for NAM activity at the human mGlu7 receptor (FAST-0154C) in dose response and in duplicates (range concentration: 1 nM – 25 μM)

Testing Protocol

cAMP HTRF assay for Gi coupled receptor

CHO-K1 cells expressing recombinant human mGlu7 receptor (FAST-0154C) grown prior to the test inmedia without antibiotic are detached by gentle flushing with PBS-EDTA (5 mM EDTA), recovered by centrifugation and resuspended in assay buffer (KRH: 5 mM KCl, 1.25 mM $MgSO_4$, 124 mM NaCl, 25 mM HEPES, 13.3 mM Glucose, 1.25 mM KH_2PO_4, 1.45 mM $CaCl_2$, 0.5 g/L BSA, supplemented with 1 mM IBMX).

Dose response curves are performed in parallel with the reference compounds.

For antagonist/NAM test (96 well): 12 µl of cells are mixed with 6 µl of the test compound at increasing concentrations and then incubated 10 min. Thereafter 6 µl of the mix of the forskolin and reference agonist is added at a final concentration corresponding to the historical EC_{80}. The plates are then incubated for 30 min at room temperature. After addition of the lysis buffer and 1 hour incubation, cAMP concentrations are estimated, according to the manufacturer specification, with the HTRF kit.

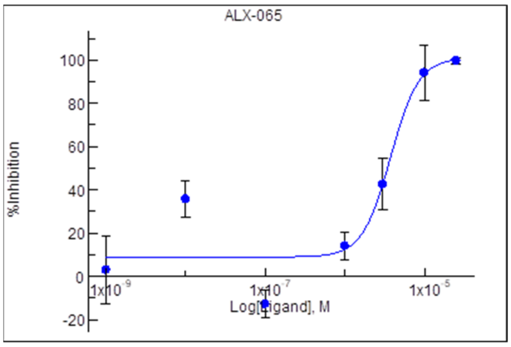

IC_{50}: 3.74 µmol
% inhibition at maximal concentration: 99.62%

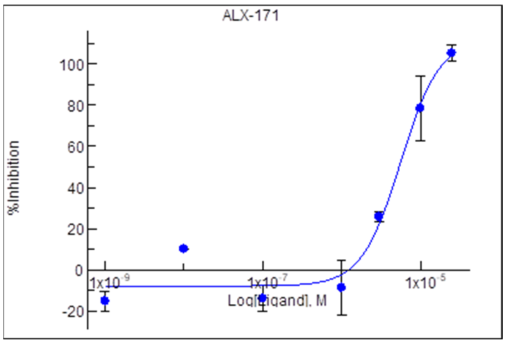

IC_{50}: 5.60 µmol
% inhibition at maximal concentration: 105.21%

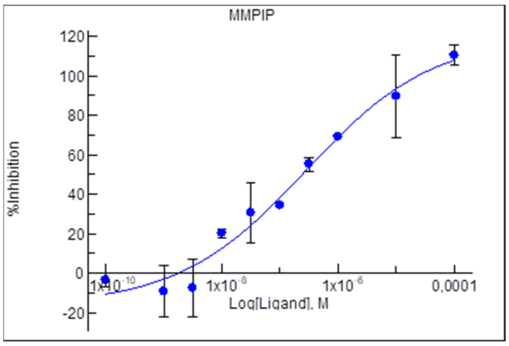

IC50: 0.27 µmol
% inhibition at maximal concentration 100%

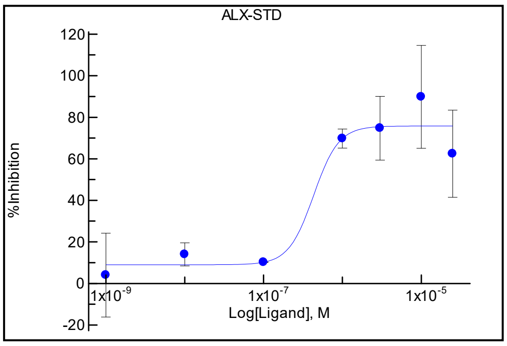

IC50: 0.43 µmol
% inhibition at maximal concentration 62.42%

Figure S4. Results demonstrating activity of NAMs **ALX-065** and **ALX-171**, MMPIP, ALX-STD (ADX71743) in CHO-K1 cells line verified by cAMP accumulation with inducible expression of human $mGlu_7$ receptor. All compounds inhibit activity of the receptor in dose dependent manner in presence of agonist L-AP4. Agonist activity of test compounds is expressed as a percentage of the activity of the reference agonist at its EC_{100} concentration. Antagonist activity of test compound is expressed as a percentage of the inhibition of reference agonist activity at its EC_{80} concentration.

2.3. $mGlu_4$ and $mGlu_8$ receptor selectivity

All active compounds were screened at 10 µM using three cell line with expression of $mGlu_4$ and $mGlu_8$ receptors to evaluate selectivity. For $mGlu_4$ and $mGlu_8$ receptors glutamic acid was used as agonist and has good potency. As reference compounds for $mGlu_4$ and $mGlu_8$ receptors were used VU0155041 and AZ12216052 respectively. Parameter described as "% of inhibition" was introduced to compare bioactivity of new compounds to reference NAM in 10 µM concentration in presence 5 µM of LSP4-2022 (0%) and 3 µM of forskolin (100%). For ADX71743 % of inhibition was 42.61% (±7.56; n=10). For compounds that passed screening procedure dose-response curve in presence of LSP4-2022 in 5 µM was evaluated compare to reference NAM ADX71743. Only **ALX-063**, **ALX-065** and **ALX-171** compounds meet our conditions regarding bioactivity. All of them were further investigated to determinate EC_{50} and receptor selectivity.

ALX-065

Off-target (T-Rex screening)

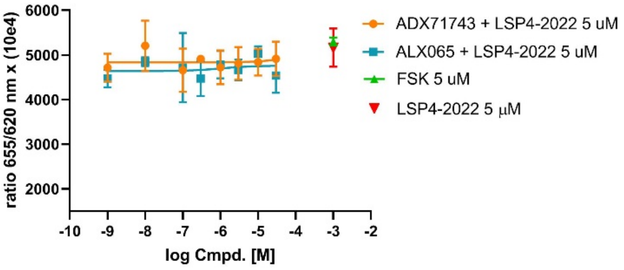

ALX-171

Off-target (T-Rex screening)

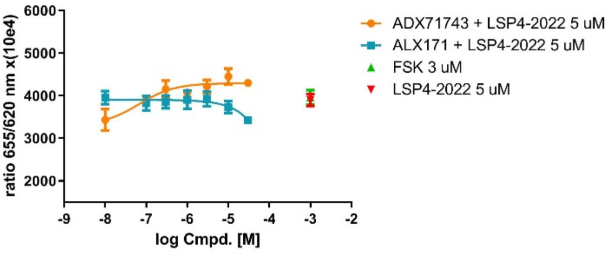

Figure S5. Activity of compounds **ALX-065 and ALX-171** in forskolin-induced cAMP accumulation in untransfected cells of the T-REx 293 line. Similar to reference mGlu$_7$ receptor ADX71743, no effects of the tested 1,2,4-oxadiazole derivatives were observed in the cAMP assay in the mock T-REx 293 cell line.

ALX-065

mGlu$_4$

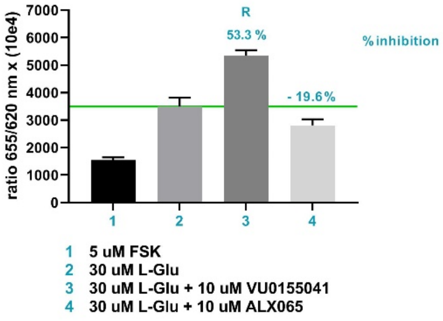

ALX-065

mGlu$_8$

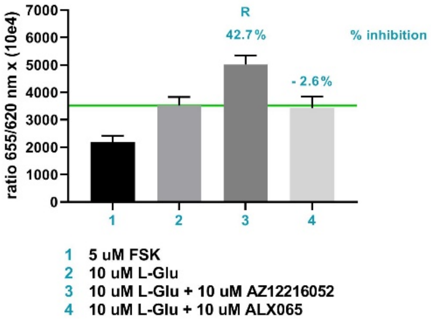

ALX-171

mGlu$_4$

ALX-171

mGlu$_8$

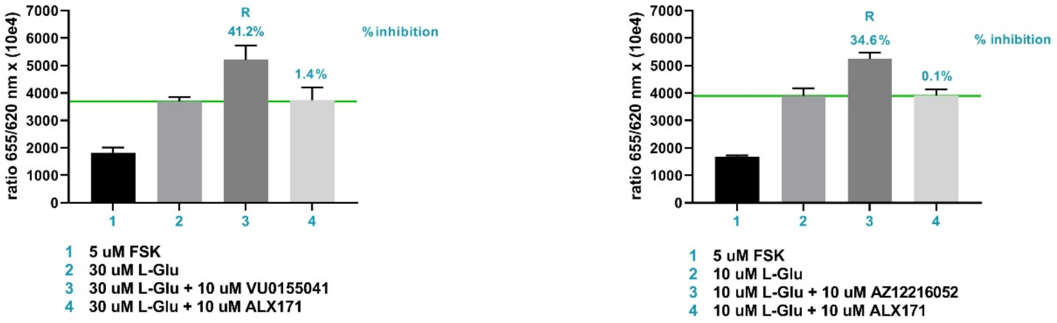

Figure S6. Activity of compounds **ALX-065** and **ALX-171** references: VU0155041(positive allosteric modulator/allosteric agonist at $mGlu_4$ receptors), AZ12216052 (positive allosteric modulator of $mGlu_8$ receptos) at a concentration of 10 μM verified by cAMP accumulation assay in cells expressing human $mGlu_4$ or $mGlu_8$ receptors.

3. References

1. Kesten, S.J.; Degnan, M.J.; Hung, J.; McNamara, D.J.; Ortwine, D.F.; Uhlendorf, S.E.; Werbel, L.M. Synthesis and Antimalarial Properties of 1-Imino Derivatives of 7-chloro-3-substituted-3,4-dihydro-1,9(2H,10H)-acridinediones and Related Structures. *J. Med. Chem.* **1992**, *35*, 3429–3447, doi:10.1021/jm00097a001.

2. Gudkov, A. US20200123127A1 - Removal of senescence-associated macrophages 2020.

3. Alexander, R.; Balasundaram, A.; Batchelor, M.; Brookings, D.; Crépy, K.; Crabbe, T.; Deltent, M.F.; Driessens, F.; Gill, A.; Harris, S.; et al. 4-(1,3-Thiazol-2-yl)morpholine derivatives as inhibitors of phosphoinositide 3-kinase. *Bioorganic Med. Chem. Lett.* **2008**, *18*, 4316–4320, doi:10.1016/j.bmcl.2008.06.076.

4. Shiraishi, M. EP1057812A1 - Aminoguanidine hydrazone derivatives, process for producing the same and drugs thereof 2000.

5. Presset, M.; Mailhol, D.; Coquerel, Y.; Rodriguez, J. Diazo-transfer reactions to 1,3-dicarbonyl compounds with tosyl azide. *Synthesis (Stuttg).* **2011**, 2549–2552, doi:10.1055/s-0030-1260107.

6. Xia, L.; Lee, Y.R. Regioselective synthesis of highly functionalized furans through the RuII-catalyzed [3+2] cycloaddition of diazodicarbonyl compounds. *European J. Org. Chem.* **2014**, *2014*, 3430–3442, doi:10.1002/ejoc.201402067.

7. Di Fabio, R. WO2009130231A1 - Pyrrolo [1, 2-a] pyrazine derivatives as vasopressin vib receptor antagonists 2009.

8. Johan D. Oslob, C.H.Y. 20070027166 A1 2007, *1*.

9. Zask, A.; Verheijen, J.C.; Curran, K.; Kaplan, J.; Richard, D.J.; Nowak, P.; Malwitz, D.J.; Brooijmans, N.; Bard, J.; Svenson, K.; et al. ATP-competitive inhibitors of the mammalian target of rapamycin: Design and synthesis of highly potent and selective pyrazolopyrimidines. *J. Med. Chem.* **2009**, *52*, 5013–5016, doi:10.1021/jm900851f.

10. Faridoon; Hussein, W.M.; Vella, P.; Islam, N.U.; Ollis, D.L.; Schenk, G.; McGeary, R.P. 3-Mercapto-1,2,4-triazoles and N-acylated thiosemicarbazides as metallo-β-lactamase inhibitors. *Bioorganic Med. Chem. Lett.* **2012**, *22*, 380–386, doi:10.1016/j.bmcl.2011.10.116.

11. Senga, K.; Novinson, T.; Wilson, H.R.; Robins, R.K. Synthesis and Antischistosomal Activity of Certain Pyrazolo[1,5-a]pyrimidines. *J. Med. Chem* **1981**, *408*, 610–613.

12. Castellano, S.; Kuck, D.; Viviano, M.; Yoo, J.; López-Vallejo, F.; Conti, P.; Tamborini, L.; Pinto, A.; Medina-Franco, J.L.; Sbardella, G. Synthesis and biochemical evaluation of δ 2-isoxazoline derivatives as DNA methyltransferase 1 inhibitors. *J. Med. Chem.* **2011**, *54*, 7663–7677, doi:10.1021/jm2010404.

13. Bode, J.W.; Hachisu, Y.; Matsuura, T.; Suzuki, K. Facile construction and divergent transformation of polycyclic isoxazoles: Direct access to polyketide architectures. *Org. Lett.* **2003**, *5*, 391–394, doi:10.1021/ol027283f.

14. Cieślik, P.; Woźniak, M.; Kaczorowska, K.; Brański, P.; Burnat, G.; Chocyk, A.; Bobula, B.; Gruca, P.; Litwa, E.; Pałucha-Poniewiera, A.; et al. Negative Allosteric Modulators of mGlu7 Receptor as Putative Antipsychotic Drugs. *Front. Mol. Neurosci.* **2018**, *11*, 1–14, doi:10.3389/fnmol.2018.00316.

15. Chruścicka, B.; Burnat, G.; Brański, P.; Chorobik, P.; Lenda, T.; Marciniak, M.; Pilc, A. Tetracycline-based system for controlled inducible expression of group III metabotropic glutamate receptors. *J. Biomol. Screen.* **2015**, *20*, 350–358, doi:10.1177/1087057114559183.
